# Whole-genome analysis showed the promotion of genetic diversity and coevolution in *Staphylococcus aureus* lytic bacteriophages and their hosts mediated by prophages *via* worldwide recombination events

**DOI:** 10.3389/fmicb.2023.1088125

**Published:** 2023-03-10

**Authors:** Wenyuan Zhou, Yajie Li, Xuechao Xu, Shengqi Rao, Hua Wen, Yeiling Han, Aiping Deng, Zhenwen Zhang, Zhenquan Yang, Guoqiang Zhu

**Affiliations:** ^1^College of Food Science and Engineering, Yangzhou University, Yangzhou, Jiangsu, China; ^2^College of Veterinary Medicine, Yangzhou University, Yangzhou, Jiangsu, China; ^3^Medical College, Yangzhou University, Yangzhou, Jiangsu, China

**Keywords:** prophages, *Staphylococcus*, bacteriophage, genomes, evolution

## Abstract

Prophages as a part of *Staphylococcus aureus* genome contribute to the genetic diversity as well as survival strategies of their host. Some *S. aureus* prophages also have an imminent risk of host cell lysis and become a lytic phage. Nonetheless, interactions among *S. aureus* prophages, lytic phages, and their hosts, as well as the genetic diversity of *S. aureus* prophages, remain unclear. We identified 579 intact and 1,389 incomplete prophages in the genomes of 493 *S. aureus* isolates obtained from the NCBI database. The structural diversity and gene content of intact and incomplete prophages were investigated and compared with 188 lytic phages. Mosaic structure comparison, ortholog group clustering, phylogenetic analysis, and recombination network analysis were performed to estimate genetic relatedness among *S. aureus* intact prophages, incomplete prophages, and lytic phages. The intact and incomplete prophages harbored 148 and 522 distinct mosaic structures, respectively. The major difference between lytic phages and prophages was the lack of functional modules and genes. Compared to the lytic phages, both the *S. aureus* intact and incomplete prophages harbored multiple antimicrobial resistance (AMR) and virulence factor (VF) genes. Several functional modules of lytic phages 3_AJ_2017 and 23MRA shared more than 99% nucleotide sequence identity with *S. aureus* intact (ST20130943_p1 and UTSW_ MRSA_55_ip3) and incomplete prophages (SA3_LAU_ip3 and MRSA_FKTN_ip4); other modules showed little nucleotide sequence similarity. Ortholog and phylogenetic analyses revealed a common gene pool shared between the prophages and lytic Siphoviridae phages. Moreover, most shared sequences existed within intact (43428/137294, 31.6%) and incomplete prophages (41248/137294, 30.0%). Therefore, the maintenance or loss of functional modules in intact and incomplete prophages is key to balance the costs and benefits of large prophages harboring various AMR and VF genes in the bacterial host. The shared identical functional modules between *S. aureus* lytic phages and prophages are likely to result in the exchange, acquisition, and loss of functional modules, and therefore contribute to their genetic diversity. Moreover, constant recombination events within prophages globally were responsible for the coevolution of lytic phages and their bacterial hosts.

## Introduction

The *Staphylococcus aureus* pathogen causes nosocomial and community-acquired infections in humans and animals owing to several immune resistance and evasion factors, toxins, and invasive mechanisms (Zhou et al., 2018; Guerillot et al., 2019). The threat of *S. aureus* can be attributed to a highly variable genome associated with phenotypic diversity and varying epidemiologic factors in different strains (Moon et al., 2015), with prophage genetic material accounting for 10–20% of the host genome (Johnson et al., 2022). Moreover, *S. aureus* can rapidly acquire antimicrobial resistance (AMR) and virulence factor (VF) genes *via* prophage transduction (Kondo et al., 2021). Studies on *S. aureus* prophage diversity at the nucleotide, structural, and genomic levels are necessary to understand the genomic plasticity and potential threats of *S. aureus* isolates.

Prophages originate from temperate bacteriophages (phages) that undergo a lysogenic cycle and integrate into the host chromosome, acting as a genetic reservoir and promoting diversity of their bacterial host (Ramisetty and Sudhakari, 2019; Johnson et al., 2022). A previous study indicated that distinct distribution of AMR and VF genes has been observed in intact and incomplete prophages in the genomes of seven pathogens, including 424 *S. aureus* isolates, derived from a public database (Kondo et al., 2021). Intact prophages can be characterized as genetic elements that can transmit VF genes and other genetic traits to *S. aureus* isolates (Moon et al., 2016). Despite the deletion of most phage genes, incomplete prophages or prophage-like elements occur stably in the genome of *Lactococcus lactis* IL1403 (Aucouturier et al., 2018). Nonetheless, the genetic relationship among lytic phages, intact prophages and incomplete prophages that is vital for elucidating the evolutionary relationships between *S. aureus* prophages and their bacterial host remains unclear.

Previously, the association of *S. aureus* prophages with increased bacterial pathogenicity and fitness has been examined (Kondo et al., 2021). The presence of different prophage-encoded VF and AMR genes among methicillin-resistant *S. aureus* (MRSA) strains enables them to produce a broad range of diseases (Dini et al., 2019). Furthermore, as genetic reservoirs, prophages promote diversity in hosts *via* enhanced recombination (Nadeem and Wahl, 2017). Besides, prophages mediate active horizontal gene transfer (HGT) *via* transduction, resulting in a large common gene pool shared by lytic phage genomes (Ramisetty and Sudhakari, 2019; Dion et al., 2020). Therefore, *S. aureus* prophages can remarkably influence the diversity and evolution of lytic phages and their hosts.

In this study, we identified 1968 prophages in 493 *S. aureus* isolate genomes and compared their sequences to those of 188 previously published lytic phages to (i) better elucidate structural diversity and gene content of *S. aureus* prophages, (ii) explore the genetic relationship between *S. aureus* intact and incomplete prophages, and (iii) understand the coevolutionary strategy of lytic phages and their hosts involving interaction with prophages.

## Materials and methods

### Collection of *Staphylococcus aureus* prophage metadata

The genomic sequences of 500 *S. aureus* isolates including 83 complete genome sequences and 417 incomplete genomes generated by high-throughput sequencing technologies were downloaded from the NCBI Genome database (April 2022). PATRIC was used to access the genome quality, as previously described (Johnson et al., 2022). In the assessment, 493 sequences derived from 58 countries globally (North America, South America, Asia, Europe, Africa, and Oceania) were selected (Supplementary Table S1). These strains were isolated from various samples (humans, *n* = 346; bovine, *n* = 39; porcine, *n* = 20; poultry, *n* = 12; sheep, *n* = 5; canine, *n* = 5; other origins, *n* = 27; unknown origin, *n* = 36). The cohort comprised genomes of 188 methicillin-sensitive *S. aureus* and 302 MRSA isolates. Phage sequences were predicted using the PHASTER software (Arndt et al., 2016) and confirmed by CheckV (Nayfach et al., 2021). The prophage regions were classified as intact (Supplementary Table S2) or incomplete (Supplementary Table S3) using the PHASTER criteria.

### Prediction and annotation of *Staphylococcus aureus* isolates and prophages

The genomes of 493 *S. aureus* isolates, 1968 predicted prophages (Supplementary Tables S2, S3), and 188 lytic phages (previously published) (Zhou et al., 2022) were analyzed using FGENESB (Liu et al., 2016), Glimmer v3.02 (Delcher et al., 2007), and GeneMarkS (Borodovsky and Lomsadze, 2014) to predict open reading frames (ORFs). ORFs were annotated using the ‘NCBI non-redundant (nr) protein database’ (Tatusova et al., 2016), ‘Clusters of Orthologous Groups’ (Galperin et al., 2019), ‘InterProScan’ (Jones et al., 2014), and ‘eggNOG’ functions (Cantalapiedra et al., 2021). AMR and VF genes were predicted using the Comprehensive Antibiotic Resistance Database (mcmaster.ca) (CARD, version 4.0.0) (Alcock et al., 2020) and virulence factor database (VFDB) (Liu et al., 2019), respectively. The latest data involving all AMR and VF genes were downloaded from CARD^1^ and VFDB^2^ websites. BLAST was used for local analysis *via* blastn with a threshold of >80% identity and coverage. CRISPR arrays in *S. aureus* isolate, prophage, and lytic phage genomes, were detected and classified using CRISPRCasFinder (version 1.6.4) with default settings (Russel et al., 2020). Genes encoding tRNAs in prophages were screened using tRNAScan-SE (Chan and Lowe, 2019).

### Phylogenetic analysis

Phylogenetic trees were constructed based on single-nucleotide polymorphisms (SNPs) to assess evolutionary relationships of *S. aureus* intact and incomplete prophages, respectively. Another phylogenetic analysis was performed to estimate the genetic relationship among the representative 148 intact prophages (Supplementary Table S4), 522 incomplete prophages (Supplementary Table S5), and 188 previously published lytic phages. Phylogenetic trees of *S. aureus* prophage and lytic phage genomes were generated using the *Erwinia* phage phiEa2809 as an outgroup. SNPs were determined for all genomes using the kSNP3 (version 3.1) software package (Gardner et al., 2015). The k-mer size estimated using the Kchooser software was set to 15 (Gardner et al., 2015). The SNP-based phylogenetic trees were constructed based on the Maximum Likelihood methods by RAxML (v 8.2.12) with 1,000 bootstraps and the GTRCAT nucleotide substitution model (Stamatakis, 2014). The phylogenetic trees were rooted using the outgroup and annotated using iTOL (Letunic and Bork, 2021). The mosaic structure of the *S. aureus* prophages was aligned using progressive MAUVE (Zhou et al., 2022).

### Orthogroup clustering

The proteomes of 188 lytic phages, 579 intact prophages, and 1,389 incomplete prophages were used for orthogroup clustering (Emms and Kelly, 2019). In total, 93,038 protein sequences (lytic phages, 20,293; intact prophages, 38,809; incomplete prophages, 33,936) were clustered using OrthoFinder v2 with the default parameters. The resulting orthogroups were annotated using the ‘NCBI nr protein database’, ‘Clusters of Orthologous Groups’, ‘InterProScan’, ‘eggNOG’ functions, CARD, and VFDB.

### Recombination network

The recombination events between the 188 lytic phages and 1968 prophages were estimated as previously described (Belcaid et al., 2010). Each sequence was queried against each other in the database using local BLAST. Homologous results with a nucleotide sequences identity of >95% were considered as the shared segments (Owen et al., 2017). The shared segments among intact prophages, incomplete prophages and lytic phages from different geographic were used to establish a worldwide recombination network as previously described (Göller et al., 2021).

### Statistical analyses

Pearson’s chi-squared test (two-tailed) was performed to analyze the differences in the distribution of genes encoding for phage morphogenesis, DNA metabolism, host cell lysis, DNA packaging, lysogeny, AMR genes and VF among intact, incomplete, and lytic phages using SPSS software (version 26).

## Results

### *Staphylococcus aureus* intact and incomplete prophages exhibit distinct mosaic structures

In total, 493 *S. aureus* sequences deposited in GenBank were analyzed in this study (Supplementary Table S1). Except for eight isolates without any prophages, *S. aureus* genomes had 4.0 ± 0.2 prophages. A total of 1968 prophages, comprising 579 intact (Supplementary Table S2) and 1,389 incomplete prophage sequences (Supplementary Table S3), were identified. Except for 20 prophages harboring 4.5 ± 2.2 tRNA genes, 1948 prophages had no tRNA genes. The genome size of intact prophages was 48.8 ± 1.5 kb with 67 ± 2 ORFs (Supplementary Table S2). The G + C content of the intact prophages was 33.5 ± 0.2%. The genome size of incomplete prophages was 18.5 ± 0.6 kb with 24 ± 1 ORFs (Supplementary Table S3). The G + C content of incomplete prophages was 31.0 ± 0.2%.

We determined two phylogenies for 579 *S. aureus* intact and 1,389 incomplete prophages, respectively. Except for 8 singletons, the phylogenetic tree based on the 49,189 SNPs from genome sequences of 579 *S. aureus* intact prophages and the Erwinia phage phiEa2809 revealed 2 major genetic lineages (I–II; Supplementary Figure S1). Lineage I consisted of 35 intact prophages and lineage II concluded 536 intact prophages. However, statistical analyses based on the Pearson’s chi-square test showed no significant differences in the distribution of intact prophages from distinct geographic origin, host and isolation source between the two lineages (*p* > 0.05). Another phylogenetic tree based on the 292,450 SNPs from genome sequences of 1,389 *S. aureus* incomplete prophages and the Erwinia phage phiEa2809 revealed 51 singletons and 2 main lineages (I–II, Supplementary Figure S2). Lineage I consisted of 46 incomplete prophages and lineage II conclude 1,292 incomplete prophages. Consistently, there was no significant differences in the distribution of incomplete prophages from distinct geographic origin, host and isolation source between the two lineages (*p* > 0.05). The mosaic structure and gene content were compared across phylogenetic groups. Colinary and MARVE analyses revealed 148 mosaic structures among intact *S. aureus* prophages (Supplementary Table S4). Five mosaic structures (types CP1–CP5) were found in 5.9% (34/579), 4.7% (27/579), 4.7% (27/579), 4.1% (24/579), and 3.6% (21/579) of the intact prophages, respectively (Supplementary Table S4 and Supplementary Figures S3–S7). Among incomplete *S. aureus* prophages, 522 mosaic structures were identified (Supplementary Table S5). Five mosaic structures (IP1–IP5) were found in 3.4% (47/1389), 3.2% (45/1389), 3.2% (44/1389), 2.7% (38/1389), and 2.5% (35/1389) of incomplete prophages, respectively (Supplementary Table S5 and Supplementary Figures S8–S12).

**Figure 1:**
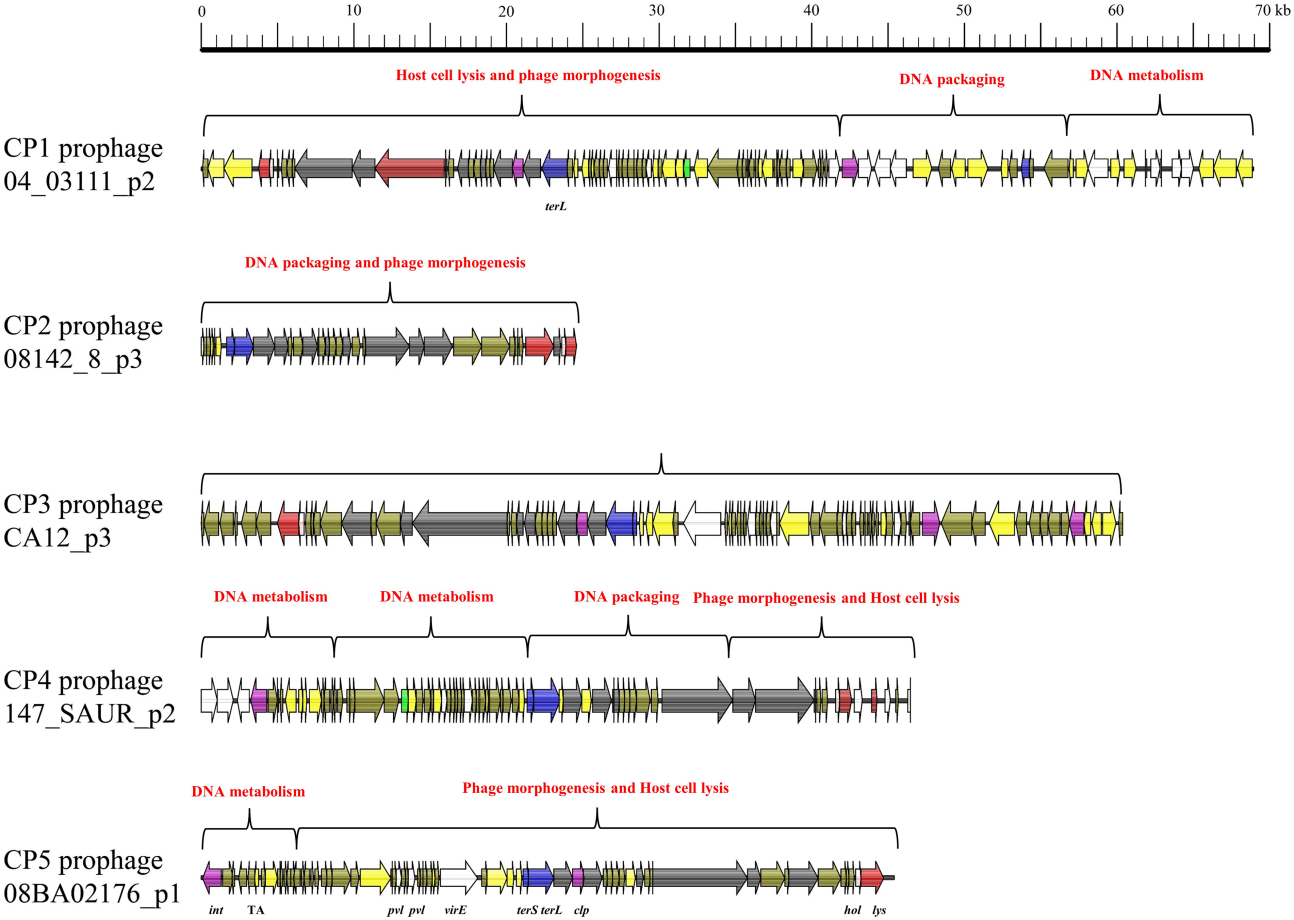
Mosaic pattern of the primary structure of intact prophages CP1–5. Functional modules are annotated with different colors. ORFs are represented by arrows indicating the transcription direction, and the colors of the arrows represent different genes. Gene color: virulence determinants, white; holin-encoding gene, pink; lysin-encoding gene, red; genes associated with lysogeny, purple; *bla*, green; DNA packaging genes, blue; DNA metabolism genes, yellow; and hypothetical protein genes, brown.

**Figure 2:**
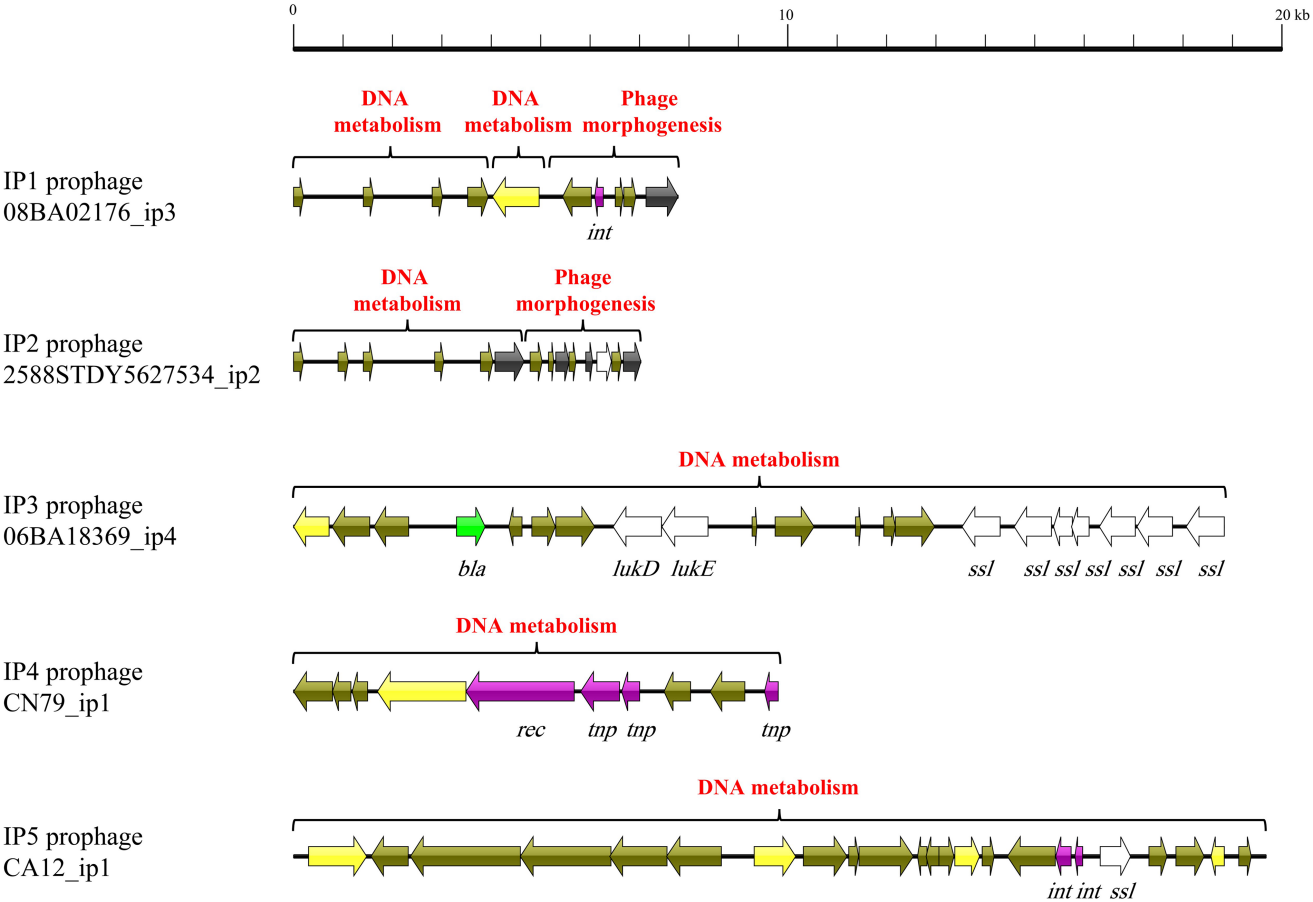
Mosaic structure of the main structure of incomplete prophages IP1–5.

All five intact prophages (CP1–CP5) typically consisted of genes associated with four functional modules: phage morphogenesis, host cell lysis, DNA packaging, and DNA metabolism (Figure 1). CP1 was consist of three functional regions: host cell lysis and phage morphogenesis, DNA packaging, and DNA metabolism. Despite CP2 and CP3 comprised only one module, this module included genes associated all four functions. CP4 comprised four functional regions including 2 DNA metabolism modules, 1 DNA packaging module, and 1 phage morphogenesis and host cell lysis module. CP5 comprised two functional regions: DNA metabolism, and phage morphogenesis and host cell lysis. However, incomplete prophages IP1–IP5 comprised only genes associated with phage morphogenesis, DNA metabolism, lysogeny, and virulence, but genes associated with host cell lysis and DNA packaging (Figure 2).

### Functional gene loss contributes to a major difference between intact and incomplete prophages

**Figure 3:**
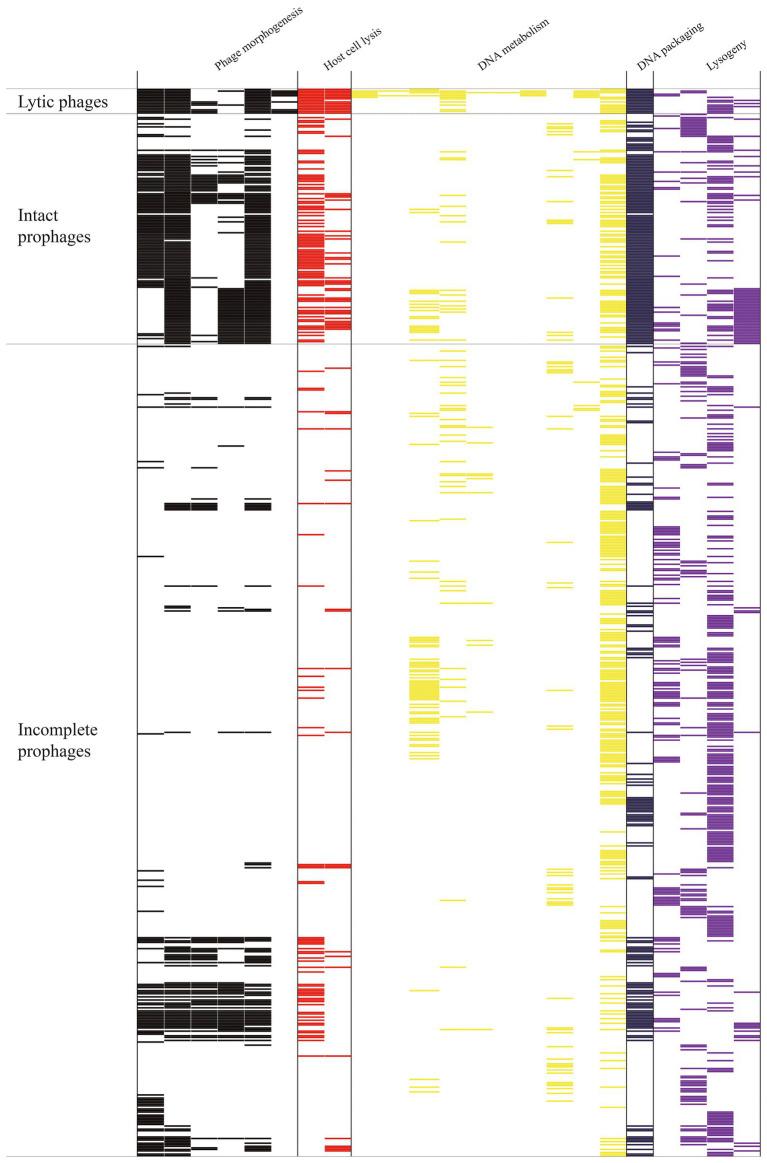
Heat map showing gene distribution associated with phage morphogenesis, host cell lysis, DNA metabolism, DNA packaging, and lysogeny of *S. aureus* 148 intact prophages, 522 incomplete prophages, and 16 lytic phages. Gene presence (colored blocks) or absence (white) is shown.

We compared the typical mosaic structures (Supplementary Tables S4, S5) of 148 intact and 522 incomplete prophages of *S. aureus* with those of 16 *S. aureus* lytic phages (Zhou et al., 2022). Genes encoding head and tail morphogenetic and portal proteins were found in all lytic phages (16/16; 100%; *p* ≤ 0.001; Figure 3). Such genes were found in 92/148 (62.2%), 125/148 (84.5%), and 121/148 (81.8%) intact prophages and 74/522 (14.2%), 72/522 (13.8%), and 62/522 (11.9%) incomplete prophages, respectively. Moreover, the prevalence of genes encoding the head-tail connector protein was much higher in lytic phages (37.5%, 6/16) than that in intact (18.9%, 28/148) and incomplete prophages (10.7%, 56/522; *p* ≤ 0.001). Genes encoding baseplate proteins were found in 50.0% (8/16) of lytic phages and 0.7% (1/148, *p* ≤ 0.001) of intact prophages. However, genes encoding tail tube proteins were more prevalent in intact prophages (37.2%, 55/148) than those in lytic phages (12.5%, 2/16) and incomplete prophages (8.2%, 43/522; *p* ≤ 0.001). As for host cell lysis module genes, *lys* and *hol* were more prevalent in lytic phages (lysin, 16/16, 100%; holin, 15/16, 93.8%) than those in intact prophages (lysin, 84/148, 56.8%; holin, 35/148, 23.6%) and incomplete prophages (lysin, 55/522, 10.5%; holin, 23/522, 4.4%; *p* ≤ 0.001). Genes encoding DNA metabolism and synthesis proteins, RNA polymerase, and type III restriction enzymes were only found in 5/16 (31.3%), 2/16 (12.5%), and 4/16 (25.0%) lytic phages, respectively. Genes encoding DNA helicase were considerably more prevalent in lytic phages (10/16, 62.5%) when compared with those in intact prophages (15/148, 10.1%) and incomplete prophages (34/522, 6.5%; *p* ≤ 0.001). Genes encoding DNA primase were only found in 11 genomes, including 31.3% (5/16), 1.4% (2/148), and 0.8% (4/522) of lytic phages, intact prophages, and incomplete prophages, respectively (*p* ≤ 0.001). The DNA packaging gene was more prevalent in lytic phages (16/16, 100%) than that in intact (15/148, 10.1%) and incomplete prophages (34/522, 22.0%; *p* ≤ 0.001). The lysogeny genes (*rec*, *tnp* and *int*) encoding for recombinase, transposase, and integrase were observed at the following rates: 16.8% (115/686), 17.3% (119/686), and 42.6% (292/686). The genes encoding Clp protease were found in 65 phages (lytic phages, 18.8%, 3/16; intact prophages, 30.4%, 45/148; and incomplete prophages, 3.3%, 17/522) (*p* ≤ 0.001).

### *Staphylococcus aureus* intact and incomplete prophages harbored multiple AMR and VF genes

AMR gene distribution was compared in 579 intact prophages, 1,389 incomplete prophages and their host genomes, respectively. Conferring resistance against 19 types of antimicrobials, 85 AMR genes were identified in these *S. aureus* isolates (Supplementary Table S6). Moreover, a total of 28 (32.9%, 28/85) and 26 (30.6%, 26/85) AMR genes were found in the genomes of intact and incomplete prophages, respectively. All of the AMR genes observed in the prophages were also found in their host genome. Notably, both intact and incomplete prophages harbored 20 genes associated with resistance against beta-lactamases, aminoglycosides, fluoroquinolones, trimethoprim, tetracycline, and multidrug use. Besides, the distribution of AMR genes among intact prophages (*n* = 148), incomplete prophages (*n* = 522), and lytic phages (*n* = 16) were also compared in this study (Supplementary Table S7). Two AMR genes were found in intact prophages (*ermC*, 2/148, 1.4%; *mepA*, 2/148, 1.4%), but absent in incomplete prophages and lytic phages (*p* ≤ 0.05). Moreover, 12 AMR genes were more frequently observed in *tnp-*carrying phages (*blaZ*, 4/119, 3.4%; *aac6_Ie_aph2_Ia*, 21/119, 17.6%; *ant*(6)*-Ia*, 12/119, 10.1%; *ant*(9)*-Ia*, 3/119, 2.5%; *aph*(2″)*-Ia*, 1/119, 0.8%; *dfrC*, 6/119, 5.0%; *cat-TC*, 1/119, 0.8%; *tet*(K), 2/119, 1.7%; *mphC*, 1/119, 0.8%; *ermA*, 3/119, 2.5%; *ermC*, 2/119, 1.7%; *msrA*, 1/119, 0.8%) than those in phages without *tnp* (*blaZ*, 3/567, 0.5%; *aac6_Ie_aph2_Ia*, 4/567, 0.7%; *ant*(6)*-Ia*, 3/567, 0.5%; *ant*(9)*-Ia*, 3/567, 0.5%; *aph*(2″)*-Ia*, 0/567, 0%; *dfrC*, 6/567, 1.1%; *cat-TC*, 0/567, 0%; *tet*(K), 1/567, 0.2%; *mphC*, 0/567, 0%; *ermA*, 3/567, 0.5%; *ermC*, 0/567, 0%; *msrA*, 0/567, 0%; *p* ≤ 0.05).

A total of 493 *S. aureus* isolates were screened for VF genes (Supplementary Table S6), and 146 VF genes associated with adherence (13.7%, 20/146), immune modulation (17.1%, 25/146), exoenzymes (12.3%, 18/146), effector delivery system (8.9%, 13/146), nutritional/metabolic factors (11.6%, 17/146), exotoxins (30.1%, 44/146), biofilms (4.8%, 7/146), stress survival (0.7%, 1/146), and antimicrobial activity/competitive advantage (0.7%, 1/146) were observed. Notably, 59.6% (87/146) and 60.3% (88/146) of VF genes were found in the genomes of intact and incomplete prophages, respectively. Both intact and incomplete prophages harbored 20 VF genes associated with adherence, immune modulation, exoenzymes, nutritional/metabolic factors, exotoxins, and biofilms. As shown in Supplementary Table S7, some VF genes were more prevalent in intact prophages (*ebp*, 6/148, 4.1%; *fnbA*, 5/148, 3.4%; *sdrD*, 8/148, 5.4%; *sea*, 6/148, 4.1%; *seh*, 2/148, 1.4%; *set8*, 5/148, 3.4%; *set10*, 2/148, 1.4%) than those in incomplete prophages (*ebp*, 2/522, 0.4%; *fnbA*, 3/522, 0.6%; *sdrD*, 8/522, 1.5%; *sea*, 4/522, 0.8%; *seh*, 0/522, 0%; *set8*, 3/522, 0.6%; *set10*, 0/522, 0%), but absent in lytic phages (*p* ≤ 0.05). However, gene encoding for staphylococcal enterotoxin A precursor (*sea*) was more prevalent in incomplete prophages (23/522, 4.4%) than that in intact prophages (1/148, 0.7%) and lytic phages (0/16, 0%; *p* ≤ 0.05).

Besides, 13 VF genes were more frequently detected in *rec*-harboring phages (*ebp*, 4/115, 3.5%; *map*, 1/115, 0.9%; *sbi*, 1/115, 0.9%; *adsA*, 2/115, 1.7%; *isdA*, 1/115, 0.9%; *isdF*, 1/115, 0.9%; *isdG*, 1/115, 0.9%; *sfaD*, 1/115, 0.9%; *srtB*, 1/115, 0.9%; *hlgA*, 1/115, 0.9%; *sea*, 4/115, 3.5%; *selp*, 2/115, 1.7%; *set9*, 1/115, 0.9%) than those in phages without gene encoding for recombinase (*ebp*, 4/571, 0.7%; *map*, 0/571, 0%; *sbi*, 0/571, 0%; *adsA*, 1/571, 0.2%; *isdA*, 0/571, 0%; *isdF*, 0/571, 0%; *isdG*, 0/571, 0%; *sfaD*, 0/571, 0%; *srtB*, 0/571, 0%; *hlgA*, 0/571, 0%; *sea*, 6/571, 1.1%; *selp*, 1/571, 0.2%; *set9*, 0/571, 0%; *p* ≤ 0.05). Moreover, 24 VF genes were more frequently detected in in *tnp*-containing phages (*map*, 1/119, 0.8%; *fnbA*, 8/119, 6.7%; *fnbB*, 4/119, 3.4%; *atl*, 1/119, 0.8%; *sasA*, 1/119, 0.8%; *sdrC*, 4/119, 3.4%; *sdrD*, 8/119, 6.7%; *adsA*, 2/119, 1.7%; *splD*, 10/119, 8.4%; *splE*, 9/119, 7.6%; *splF*, 11/119, 9.2%; *sspA*, 1/119, 0.8%; *sspB*, 1/119, 0.8%; *esaG*, 1/119, 0.8%; *hla*, 5/119, 4.2%; *seg*, 11/119, 9.2%; *sei*, 10/119, 8.4%; *seln*, 1/119, 0.8%; *set4*, 1/119, 0.8%; *set5*, 1/119, 0.8%; *set7*, 5/119, 4.2%; *set8*, 5/119, 4.2%; *set9*, 1/119, 0.8%; *set10*, 2/119, 1.7%) than those in the phages without *tnp* (*map*, 0/567, 0%; *fnbA*, 0/567, 0%; *fnbB*, 2/567, 0.4%; *atl*, 0/567, 0%; *sasA*, 0/567, 0%; *sdrC*, 5/567, 0.9%; *sdrD*, 8/567, 1.4%; *adsA*, 1/567, 0.2%; *splD*, 17/567, 3.0%; *splE*, 13/567, 2.3%; *splF*, 13/567, 2.3%; *sspA*, 0/567, 0%; *sspB*, 0/567, 0%; *esaG*, 0/567, 0%; *hla*, 4/567, 0.7%; *seg*, 14/567, 2.5%; *sei*, 14/567, 2.5%; *seln*, 0/567, 0%; *set4*, 0/567, 0%; *set5*, 0/567, 0%; *set7*, 3/567, 0.5%; *set8*, 3/567, 0.5%; *set9*, 0/567, 0%; *set10*, 0/567, 0%; *p* ≤ 0.05). Furthermore, 10 VF genes were more prevalent in *int-* harboring phages (*eno*, 8/292, 2.7%; *geh*, 12/292, 4.1%; *hlb*, 54/292, 18.5%; *lukS-PV*, 7/292, 2.4%; *lukF-PV*, 7/292, 2.4%; *lukG*, 53/292, 18.2%; *lukH*, 53/292, 18.2%; *sec*, 9/292, 3.1%; *sell*, 9/292, 3.1%; *tsst-1*, 8/292, 2.7%) than those in phages without gene encoding for integrase (*eno*, 3/394, 0.8%; *geh*, 1/394, 0.3%; *hlb*, 29/394, 7.4%; *lukS-PV*, 1/394, 0.3%; *lukF-PV*, 1/394, 0.3%; *lukG*, 5/394, 1.3%; *lukH*, 5/394, 1.3%; *sec*, 1/394, 0.3%; *sell*, 1/394, 0.3%; *tsst-1*, 1/394, 0.3%; *p* ≤ 0.05).

### *Staphylococcus aureus* lytic phages and prophages shared identical functional modules

**Figure 4:**
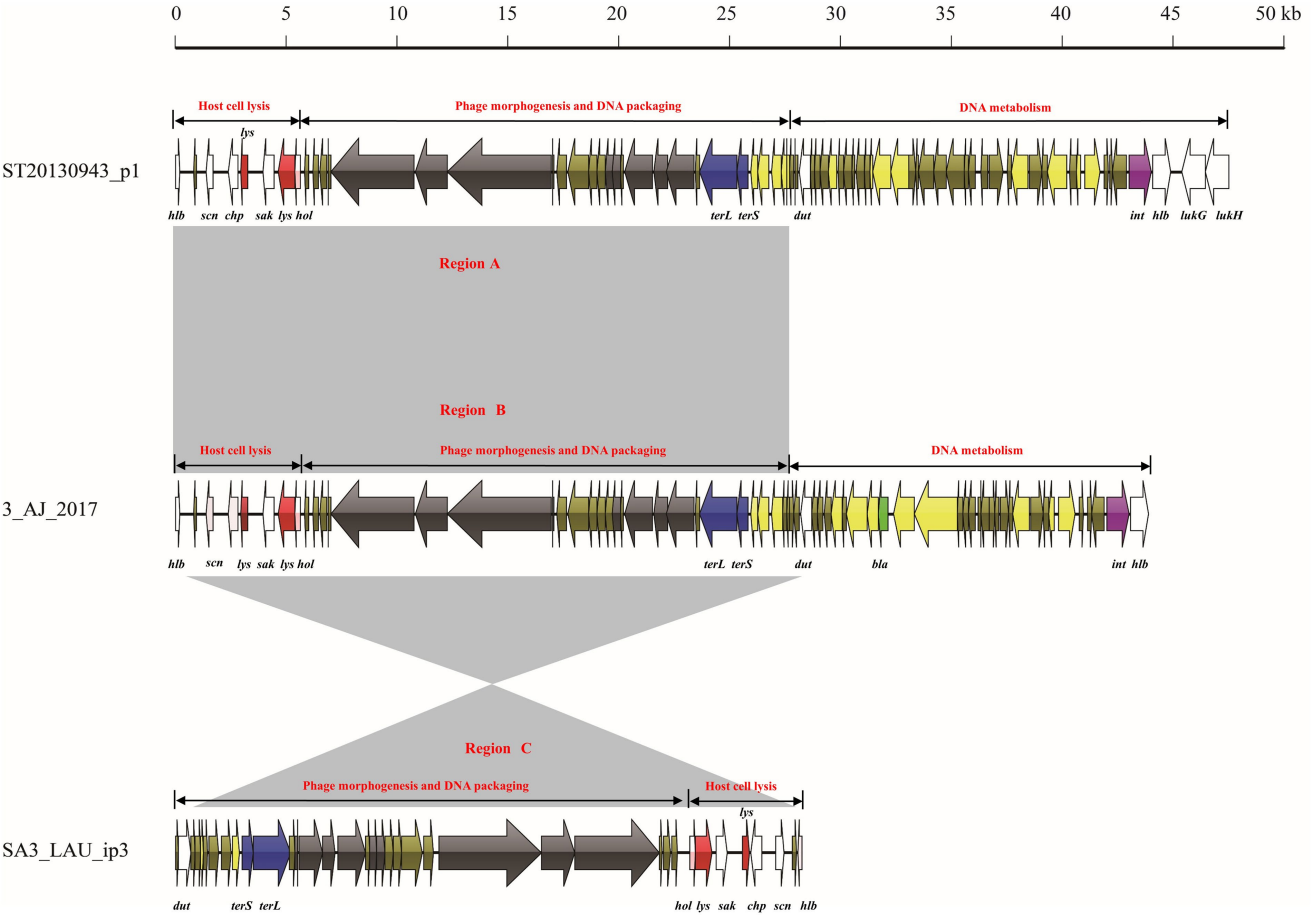
Comparative structural analyses of lytic phage-23MRA with intact prophage UTSW_MRSA_55_p3 and incomplete prophage MRSA_FKTN_ip4.

The mosaic structures of intact prophage ST20130943_p1, lytic phage 3_AJ_2017, and incomplete prophage SA3_LAU_ip3 were analyzed (Figure 4). The prophage ST20130943_p1 genome comprised 47,507 bp and three functional modules (host cell lysis module, phage morphogenesis and DNA packaging module, and DNA metabolism module). The host cell lysis module comprised eight ORFs with four virulence genes (*hlb*, *scn*, *chp*, and *sak*) and three host cell lysis genes (*lys* and *hol*). The phage morphogenesis and DNA packaging module comprised eight genes encoding phage morphogenesis proteins and two DNA packaging genes. The DNA metabolism module comprised four virulence genes (*dut*, *hlb*, *lukG*, and *lukH*) and one lysogeny gene (*int*). The lytic phage-3_AJ_2017 genome comprised 43,922 bp and three functional modules (host cell lysis module, phage morphogenesis and DNA packaging module, and DNA metabolism module). The DNA metabolism module comprised one lysogeny (*int*), one AMR gene (*bla*), and two virulence (*dut* and *hlb*) genes. The prophage SA3_LAU_ip3 genome comprised only 28,267 bp and two functional modules (phage morphogenesis and DNA packaging module, and host cell lysis module). Notably, region A of the intact prophage ST20130943_p1 comprised 27,929 bp and 35 ORFs, including two functional modules (host cell lysis module, and phage morphogenesis and DNA packaging module). This region showed more than 99.0% nucleotide sequence identity with region B in the lytic phage 3_AJ_2017 and region C in the incomplete prophage SA3_LAU_ip3.

**Figure 5:**
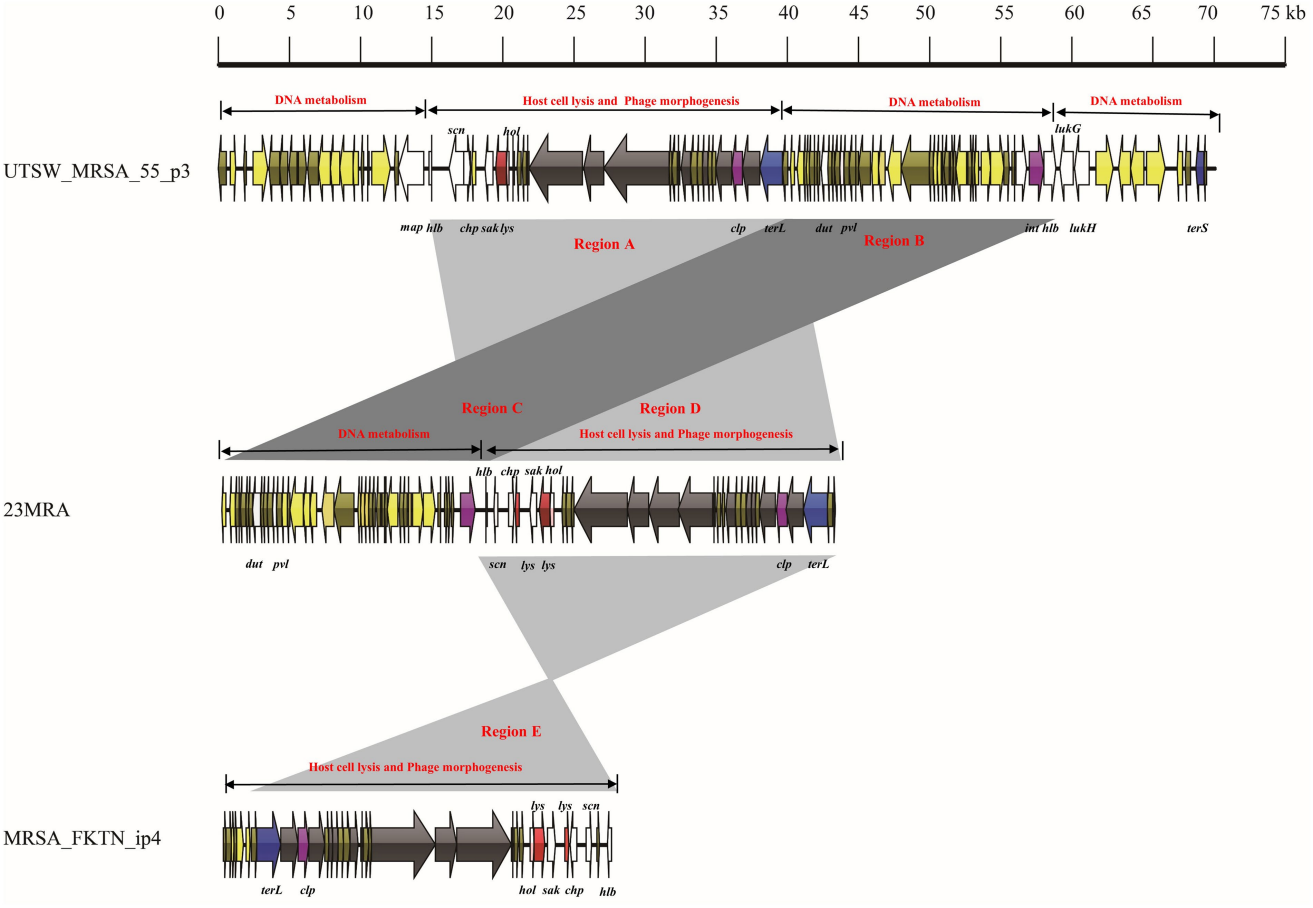
Comparative structural analyses of lytic phage-3_AJ_2017 with intact prophage ST20130943_p1 and incomplete prophage SA3_LAU_ip3.

The mosaic structures of intact prophage UTSW_MRSA_55_p3, lytic phage 23MRA, and incomplete prophage MRSA_FKTN_ip4 were also compared (Figure 5). The incomplete prophage-UTSW_MRSA_55_p3 comprised 70,093 bp and four functional modules including three DNA metabolism modules, and one host cell lysis and phage morphogenesis module. The first DNA metabolism module comprised one virulence gene (*map*). The host cell lysis and phage morphogenesis module comprised one lysogeny gene (*clp*), two host cell lysis genes (*lys* and *hol*), seven phage morphogenesis genes, four virulence genes (*hlb*, *scn*, *chp*, and *sak*) and one DNA packaging gene (*terL*). The second DNA metabolism module comprised three virulence genes (*dut*, *pvl* and *hlb*), and one lysogeny gene (*int*). The third DNA metabolism module comprised two virulence genes (*lukG,* and *lukH*), and one DNA packaging gene (*terS*). The lytic phage 23MRA genome comprised two modules (DNA metabolism module, and host cell lysis and phage morphogenesis module). The DNA metabolism module comprised two virulence genes (*dut*, and *pvl*), and one lysogeny gene (*int*). The host cell lysis and phage morphogenesis module comprised two lysin genes (*lys*), one holin gene (*hol*), one lysogeny gene (*clp*), four virulence genes (*hlb*, *scn*, *chp*, and *sak*) and one DNA packaging gene (*terL*). The incomplete prophage-MRSA_FKTN_ip4 genome was 27,240 bp in size and comprised one DNA packaging gene (*terL*), one lysogeny gene (*clp*), two host cell lysis genes (*hol* and *lys*), and four virulence genes (*sak*, *chp*, *scn*, and *hlb*). Notably, region A in the UTSW_MRSA_55_p3 genome comprised one functional module (host cell lysis and phage morphogenesis module), which showed more than 99.0% nucleotide sequence identity with region D of the lytic phage 23MRA and region E of the incomplete prophage MRSA_FKTN_ip4. Moreover, region B in the UTSW_MRSA_55_p3 genome comprised another functional module (DNA metabolism module) and showed more than 99.0% nucleotide sequence identity with region C of the lytic phage 23MRA.

### High prevalence of recombination events among *Staphylococcus aureus* prophages promotes coevolution of the *Staphylococcus aureus* lytic phage and its host

**Figure 6:**
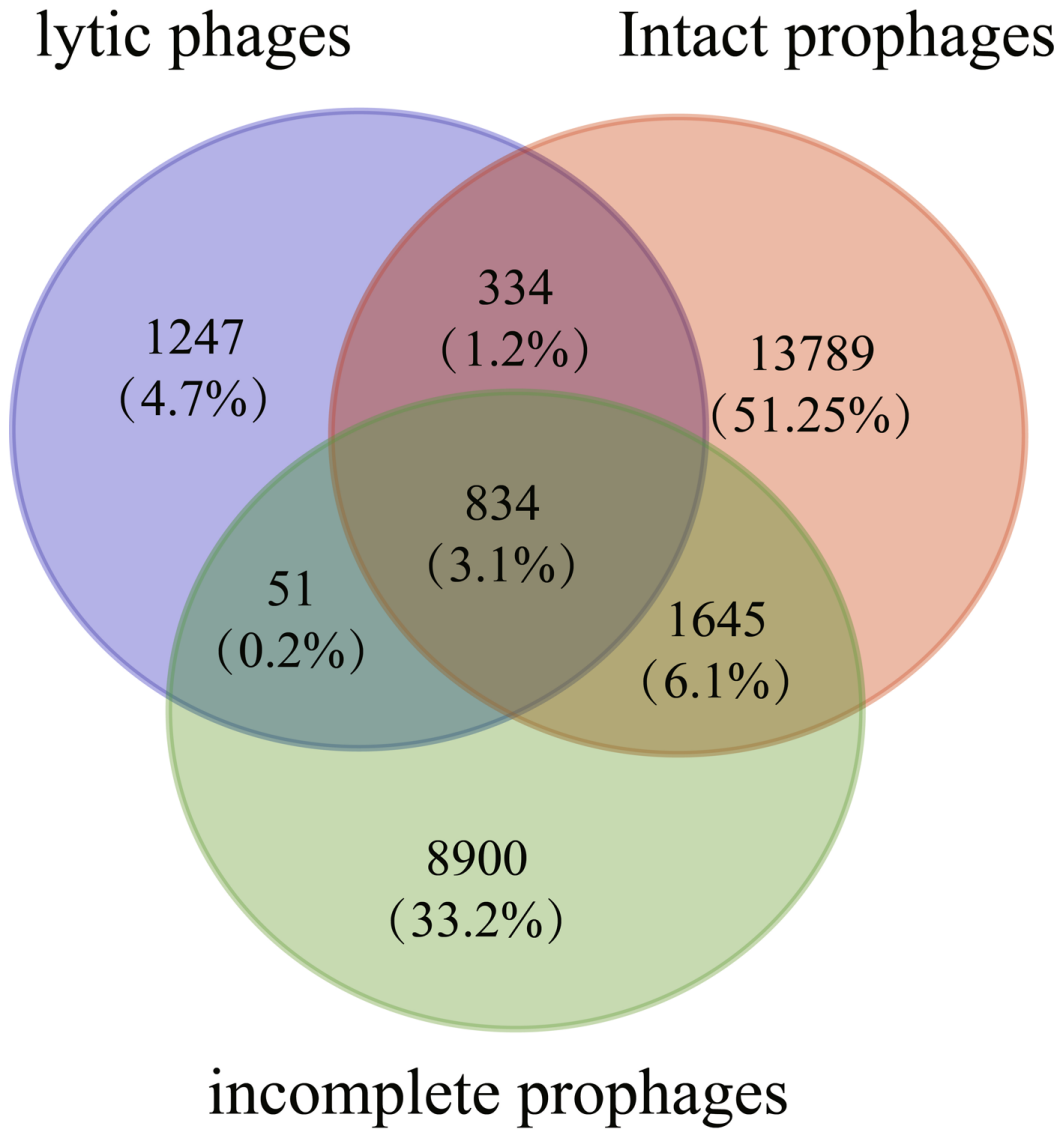
Venn diagrams showing the number of orthologous groups among 579 intact prophage sequences, 1,389 incomplete prophages, and 188 lytic phages.

Ortholog clusters were analyzed among 188 lytic phages, 579 intact prophages, and 1,389 incomplete prophages (Figure 6). Orthofinder revealed 26,800 orthogroups in these sequences. Notably, 2,466 orthogroups were found in *S. aureus* lytic phages including 1,219 orthogroups, which were also found in prophage genomes. Moreover, 834 orthogroups were found among all lytic phages and prophages, including genes encoding phage morphogenesis proteins (head proteins, 9; capsid proteins, 5; portal proteins, 5; head-tail joint proteins, 5; tail proteins, 21), host cell lysis (lysins, 2; holins, 8), DNA metabolism (DNA polymerases, 3; DNA-binding proteins, 15; DNA helicases, 2; type III restriction enzyme, 1), DNA packaging proteins, lysogeny proteins (recombinase, 1; transposases, 2; integrases, 12; Clp proteases, 2), and virulence factors (Staphylokinase, 2; beta-hemolysin, 2; Panton-Valentine leukocidin chain S precursor, 2; chemotaxis-inhibiting protein CHIPS, 1; complement inhibitor SCIN, 1; staphylococcal enterotoxin A precursor, 1; staphylococcal enterotoxin type P, 1; glycerol ester hydrolase, 1).

**Figure 7:**
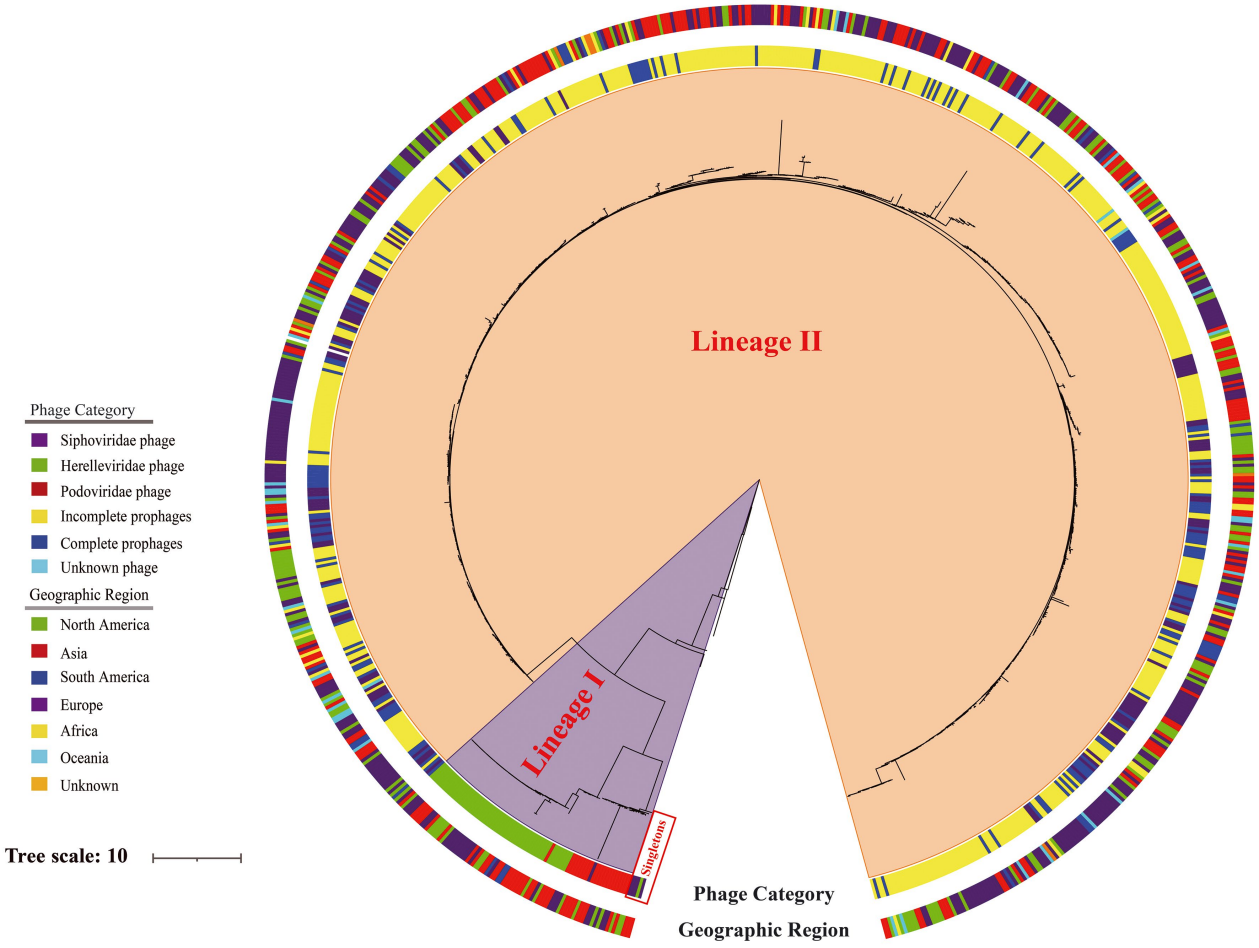
Phylogeny of 188 lytic phages, 148 representative intact prophages and 522 representative incomplete prophages analyzed using *Erwinia* phage phiEa2809 as the outgroup based on 439,973 single-nucleotide polymorphisms. The inner ring is colored according to type of the sequence and the outer ring according to the geographic region.

Phylogenetic analysis was based on 439,973 SNPs from the genome sequences of 188 lytic phages, 148 representative intact prophages (Supplementary Table S4), 522 representative incomplete prophages (Supplementary Table S5), and the *Erwinia* phage phiEa2809. Except for five singletons, this tree revealed two major genetic lineages (I–II; Figure 7). The most ancestral sequences (singletons) comprised five lytic phages, including four Siphoviridae and one Herelleviridae phage. Lineage I comprised exclusively 79 lytic phages, including 19 and 60 phages belonging to Podoviridae and Herelleviridae, respectively. However, lineage II comprised the genomes of 148 intact prophages, 522 incomplete prophages, 101 Siphoviridae lytic phages, and three unclassified lytic phage genomes.

**Figure 8:**
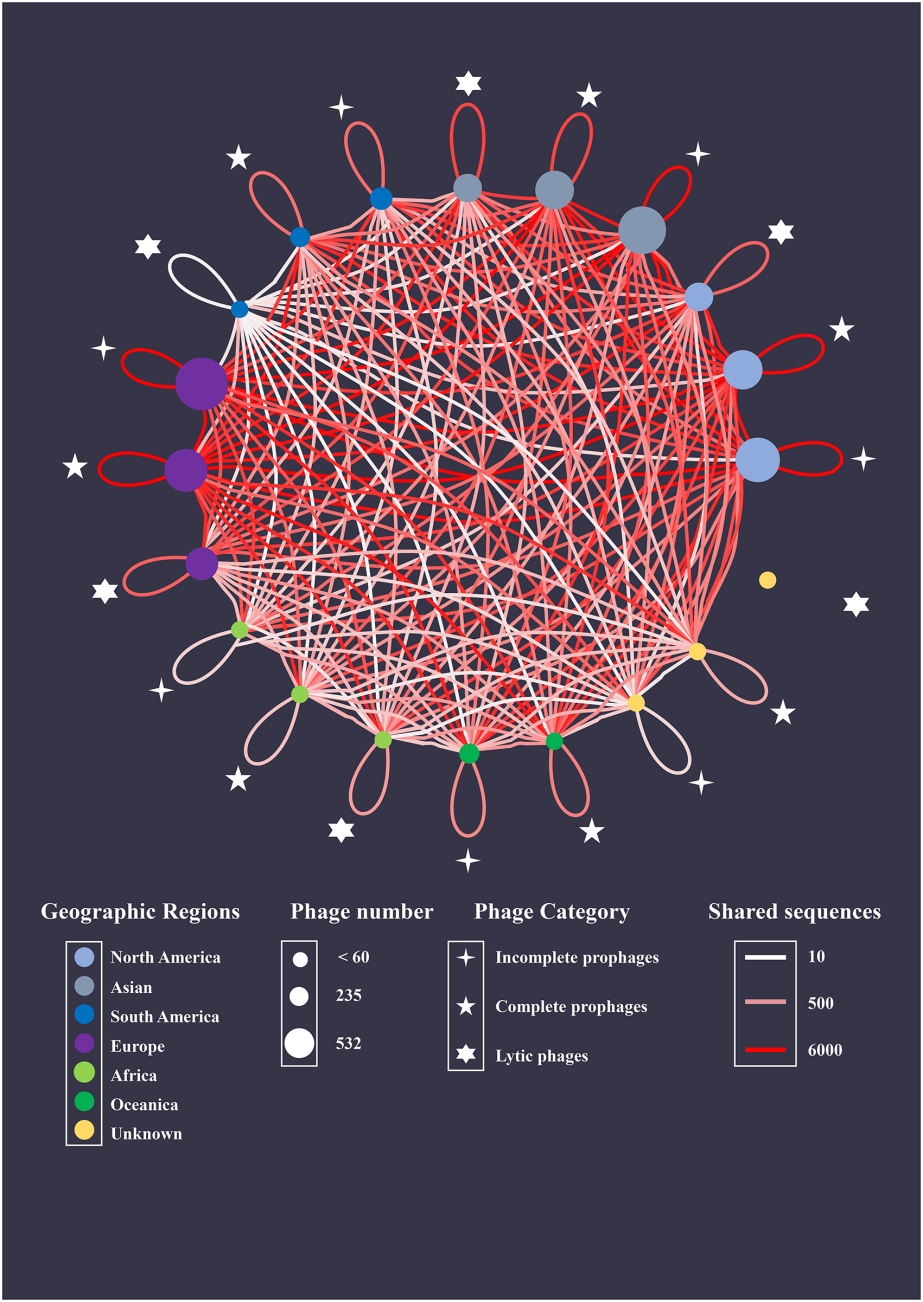
Genetic relationship network with shared sequences as coupling links. *S. aureus* intact prophage, incomplete prophages, and lytic phages isolated from different geographic regions are represented as nodes. The area of each node directly correlates with the average number of shared sequences connected to a species. The number of shared sequences between nodes is represented by colored edges.

The recombination events were analyzed among intact prophages, incomplete prophages, and lytic phages (Figure 8). Phages and prophages from different geographic regions were represented as nodes, and the edges between the two nodes were colored based on the number of shared sequences. The BLAST algorithm revealed 137,294 shared segments with a query length of 5,003–86,192 bp among the 2,156 sequences. Most shared sequences existed within intact (43428/137294, 31.6%) and incomplete prophages (41248/137294, 30.0%). The predominant interplays occurred in 235 intact prophages (11347/137294, 8.3%) and 532 incomplete prophages (10099/137294, 7.4%) from Europe. Notably, 11.6% (15879/137294) of the shared sequences existed between lytic phages and prophages (intact prophages, 11571/137294, 8.4%; incomplete prophages, 4308/137294, 3.1%). Furthermore, predominant interplay occurred between the lytic phages from North America and intact prophages from Europe (1545/137294, 1.1%), and the lytic phages from Asia and intact prophages from Europe (1178/137294, 0.9%).

Besides, we assessed the distribution of genes associated with CRISPR-Cas in 493 *S. aureus* isolates, 1968 prophages, and 188 lytic phages. Notably, only 7 *S. aureus* isolates (08BA02167, APS210, APS211, JS395, M1169, M1216, and ST1358) harbored type III-A CRISPR-Cas proteins and operons. However, none of the CRISPR-Cas proteins were predicted in the prophages and lytic phages.

## Discussion

Prophages play potent roles in the pathogenicity, fitness and diversity of their *S. aureus* hosts *via* HGT. However, the genetic diversity of prophages at the nucleotide, structural, and genomic levels remains unknown. The both phylogenies for *S. aureus* intact and incomplete prophages revealed that prophages from distinct geographic origin and host were clustered together, indicating the genetic diversity of the *S. aureus* prophages. Besides, our previous study analyzed the genetic diversity of 188 *S. aureus* lytic phages and observed 16 mosaic structures (Zhou et al., 2022), while in this study, we identified as many as 670 distinct mosaic structures (148 intact and 522 incomplete prophages) in the genomes of prophages, indicating the extraordinary structural diversity of *S. aureus* prophages. The transduction of temperate bacteriophage phiSaBov is accompanied by mobilization of the prophages vSaα, vSaβ, and vSaγ (Moon et al., 2015, 2016). Therefore, distinct mosaic structures of intact and incomplete prophages of *S. aureus* promote genomic plasticity in hosts and genetic diversity in *S. aureus* lytic phages. Intact and incomplete prophages exhibited distinct mosaic structures, which was consistent with those of a previous study indicating that mosaicism of phage genomes is perhaps one of the few common features (Dion et al., 2020). Our previous study revealed that the mosaic structure that harbored the most VF genes among the 16 main structures of *S. aureus* lytic phages was the clade IIIa (*hlg*, *pvl*, *dut*, and *virE*), clade IIIb (*dut*, *pvl*, *scn*, and *sak*) and clade IIIc (*hlb*, *sak*, and *dut*) phages (Zhou et al., 2022). However, higher prevalence of the VF genes was observed in the genomes of CP1 (*dut*, *hlb*, *lukG*, *lukH*, and *hld*), CP3 (*virE*, *dut*, *lukH*, *lukG*, *hlb*, *scn* and *sak*), CP4 (*lukH*, *lukG*, *hlb*, *scn*, *sak*, and *dut*), and IP3 (*splA*, *splB*, *splC*, *lukD*, *lukE*, *seg*, *seln*, *yent1*, *yent2*, *sei*, *selm* and *selo*). Therefore, the *S. aureus* prophages probably contributes to the transmission of VF genes in their hosts.

The major difference of the major mosaic structures between lytic phages, and the intact and incomplete prophages was the functional modules as well as genes. The 16 major mosaic structures of lytic phages normally harbored four functional modules and other genes associated with the VF and lysogeny (Zhou et al., 2022). However, the intact and incomplete prophages were lack of functional genes associated with typical phage modules including phage morphogenesis, host cell lysis, DNA metabolism, and DNA packaging. These results were inconsistent with those of previous studies, suggesting that large-scale deletions occurred in the accessory genes of prophages such as integrase and cargo genes, rather than conserved genes involved in lytic gene repression, replication, expression of capsid proteins, and packaging (Ramisetty and Sudhakari, 2019). *S. aureus* isolates harboring large prophages and related genes impose an extra metabolic burden but may promote bacterial adaptation and habitat colonization (Fillol-Salom et al., 2019; Ramisetty and Sudhakari, 2019). Therefore, we hypothesized that the maintenance or loss of functional genes is key to balancing the costs and benefits of large prophages, thus expediting bacterial genome evolution. Besides, phage-induced lysis involves the destruction of the inner membrane, peptidoglycan layer, and outer membrane of the host cell through the phage encoded holin and lysin (Young, 2014). Double-stranded DNA bacteriophages package their genome at high pressure inside a procapsid through the DNA packaging proteins (Cuervo et al., 2019). These results indicated the loss of functional modules in prophages is the possible mechanism to ground the phages and, thus protect the *S. aureus* isolates from the lysis cycle of phages, which is consistent with a previous study (Bernheim and Sorek, 2020).

AMR in *S. aureus* is a serious growing problem associated with enormous human and medical costs, and resistance transmission is promoted by mobile genetic elements (Allen et al., 2010). Furthermore, *S. aureus* can survive robust activation of the host’s innate immune system as a result of the expression of a wide array of VFs, disabling the acquired immune response (Goldmann and Medina, 2018). Compared to the only 1 AMR gene (*bla*) and 5 VF genes observed in the genomes of 188 lytic phages (Zhou et al., 2022), this study revealed various AMR and VF genes identified in the genomes of *S. aureus* prophages. These results indicates that prophages act as a genetic reservoir of AMR and VF for *S. aureus* and that transduction is the major means of HGT for AMR and VF, consistent with the results of a recent study (Kondo et al., 2021). Over the past 75 years, beta-lactams have been the most important antibiotics used in treating *S. aureus* infections (Vestergaard et al., 2019). A previous study surveyed global antibiotic consumption from 2000 to 2010 and suggested that antimicrobial drugs, such as aminoglycosides, fluoroquinolones, trimethoprim, and tetracycline, were the most abundantly consumed antibiotics globally (Van Boeckel et al., 2014). *S. aureus* strains that secrete exotoxins and exoenzymes can disrupt endothelial and epithelial barriers *via* cell lysis and junction protein cleavage (Tam and Torres, 2019). Adhesins allow *S. aureus* to attach to host cells and the extracellular matrix during the early colonization phase (Negron et al., 2022). Nutritional/metabolic factors are critical for the fitness of *S. aureus* and its adaptation to nutritionally diverse environments (Balasubramanian et al., 2017). The formation of *S. aureus* biofilms *in vivo* renders it highly resistant to chemotherapeutics, leading to chronic diseases (Paharik and Horswill, 2016). Hence, intact and incomplete prophages of *S. aureus* contribute to adaptation to the host environment and the severity of *S. aureus* infections.

In addition, our analysis revealed that the prevalence of several AMR and VF genes in the intact prophages were significantly higher than those in incomplete prophages and lytic phages. These results suggested the easily spreading of AMR and virulence *via* prophage transduction, which was consistent with previous studies (Moon et al., 2015, 2016). Besides, the numbers of some AMR and VF genes in lysogeny genes (*rec*, *tnp*, and *int*) harboring phages were significantly higher than those in phages without lysogeny genes, which is inconsistent with a previous study revealing that prophages containing VF-encoding genes are not likely to possess recombination-related genes near to VF genes (Kondo et al., 2021). These results indicated that the AMR genes and VF in the prophages may be acquired by various mechanism as well as phage transduction.

Phage 3-AJ-2017, isolated in Colombia, belongs to the Siphoviridae family. The host cell lysis module, and phage morphogenesis and DNA packaging module of phage 3-AJ-2017 shared high nucleotide sequence identity with an intact prophage in *S. aureus* strain ST20130943 isolated from humans in Brazil and an incomplete prophage in *S. aureus* strain SA3_LAU isolated from humans in Lebanon. However, the DNA metabolism module of phage-3-AJ-2017 shared little nucleotide sequence identity with ST20130943_p1. Consistently, the host cell lysis and phage morphogenesis module of phage 23MRA were similar to those of an intact prophage in *S. aureus* strain UTSW_MRSA_55 and an incomplete prophage in *S. aureus* strain MRSA_FKTN isolated from humans in the United States. The DNA metabolism module of phage 23MRA shared high nucleotide sequence identity with UTSW_MRSA_55_p3. However, the genome of UTSW_MRSA_55_p3 harbored other genes associated with DNA metabolism. These results revealed the identical functional modules between the lytic phages and prophages, which is likely to result in the easily homologous recombination events between the lytic phages and prophages (Dion et al., 2020). A previous study indicated that extensive mosaicism with genes organized into functional modules that are frequently exchanged between phages because they coexist in a common host (Deghorain and Van Melderen, 2012). Therefore, exchange, acquisition, and loss of functional modules resulting from homologous recombination may augment the genetic diversity of the lytic phages, prophages and their hosts. However, a limitation of this study can be attributed to the numbers of *S. aureus* lytic phage genomes analyzed, therefore a more accurate determination of the genetic relationship between the prophage and lytic phage is required.

Consistently, ortholog and phylogenetic analyses indicated that prophages and lytic phages shared a common core gene pool and therefore could easily exchange, acquire, and lose genetic material in the host genomes. In the phylogenetic tree, the prophages were exclusively clustered with the *S. aureus* lytic Siphoviridae phages, indicating that the Siphoviridae lytic phage and prophages shared a common ancestor. These results suggest that a broad recombination events probably occurred between prophages and *S. aureus* Siphoviridae phages, consistent with the previous study (Deghorain and Van Melderen, 2012). The phylogenetic tree obtained *via* whole-genome phylogenetic analysis revealed that the *S. aureus* prophages and lytic Siphoviridae phages from different regions belonged to the same lineage, indicating that the recombination events between *S. aureus* prophages and lytic Siphoviridae phages occurred globally. However, future research should elucidate the taxonomic classification of these prophages and their evolutionary relationship with Siphoviridae phages based on their genetic features.

The worldwide genome interaction network revealed the majority of shared sequences in *S. aureus* prophages, representing the complex recombination events may easily occur in these prophages (Belcaid et al., 2010). Recombination events, called HGT, are major mediators of phage evolution and render the understanding of evolutionary relationships difficult (Dion et al., 2020). Therefore, frequent recombination events within *S. aureus* prophages resulting from homologous recombination promoted the genetic diversity of *S. aureus* isolates as well as prophages. Consistent with these results, region A in the genome of 09_01244_p4 comprised 43,514 bp and shared 99.0% nucleotide sequence identity with region B in the *S. aureus* prophage BSAR202_p1 (Supplementary Figure S13). Moreover, region B in the MAL9_ip4 genome comprised 29,477 bp and shared 98.0% nucleotide sequence identity with region D in the prophage MRGR3_ip9 (Supplementary Figure S14). Incomplete prophages that were considered genetic junks confer advantageous phenotypes to bacterial hosts with respect to virulence, stress resistance, or mutation rate (Ramisetty and Sudhakari, 2019). The present study illustrates that incomplete prophages can also be reservoirs of genetic material for lytic phages. These results identified that the constant recombination between *S. aureus* prophages in host chromosomes and lytic phages tightly links their evolution and indirectly accelerates bacterial evolution. However, future studies should elucidate the exact mechanism underlying recombination events among *S. aureus* intact prophages, incomplete prophages and lytic phages.

The CRISPR-Cas system is an important constraint for HGT, and ensuring the maintenance or loss of prophages is key to the environmental adaption of this pathogen (Wheatley and MacLean, 2021). The non-specific DNase activity of the staphylococcal type III-A CRISPR-Cas system contributes to mutation rates in bacteria and AMR in *S. aureus* and *S. epidermidis* (Mo et al., 2021). Therefore, our study suggested that type III-A CRISPR-Cas proteins may mediate the genetic diversity of *S. aureus* prophages as well as their hosts. Considering the low detection rate of CRISPR-Cas genes in *S. aureus* genomes, recombination within *S. aureus* prophages *via* HGT was responsible for the genetic diversity and coevolution in *S. aureus* prophages, lytic phages, and their hosts.

## Conclusion

In summary, our analysis suggests that intact and incomplete *S. aureus* prophages exhibited distinct mosaic structures, and the major difference between lytic phages, and intact and incomplete prophages was the loss of functional modules and genes. Compared to the lytic phages, both the *S. aureus* intact and incomplete prophages harbored multiple AMR and VF genes and contributed to the environmental adaption of its hosts and infection severity. Hence, the maintenance or loss of functional modules in intact and incomplete prophages is key to balance the costs and benefits of large prophages harboring various AMR and VF genes in the bacterial host. Besides, exchange, acquisition, and loss of functional modules between lytic phages and prophages resulting from homologous recombination contribute to the genetic diversity of *S. aureus* prophages. Constant recombination within prophages globally were responsible for the genetic diversity of *S. aureus* lytic phages and their host. Through these evolutionary strategies, the *S. aureus* prophage accelerates the evolution itself and the coevolution of lytic phages and their bacterial hosts.

## Data availability statement

The datasets presented in this study can be found in online repositories. The names of the repository/repositories and accession number(s) can be found in the article/Supplementary material.

## Author contributions

WZ and ZY generated the research and critically reviewed the final version of manuscript. WZ and GZ designed and conceptualized the research. WZ and YL performed the research. HW, YH, and AD analyzed the data. XX, SR, and ZZ supervised and edited the manuscript. All authors contributed to the article and approved the submitted version.

## Funding

This research was supported by the National Natural Science Foundation of China (grant numbers: 32102100 and 32001661), the China Postdoctoral Science Foundation (grant number: 2022M712695), Natural Science Foundation of Jiangsu Province, China (grant number: BK20190890), Jiangsu Key Research & Development Program (grant number: BE2019436-5), Yangzhou University food quality and safety talents project (grant number: YZUJX2020-A3), and Yangzhou city talents project.

## Conflict of interest

The authors declare that the research was conducted in the absence of any commercial or financial relationships that could be construed as a potential conflict of interest.

## Publisher’s note

All claims expressed in this article are solely those of the authors and do not necessarily represent those of their affiliated organizations, or those of the publisher, the editors and the reviewers. Any product that may be evaluated in this article, or claim that may be made by its manufacturer, is not guaranteed or endorsed by the publisher.

## Supplementary material

The Supplementary material for this article can be found online at: https://www.frontiersin.org/articles/10.3389/fmicb.2023.1088125/full#supplementary-material

## Footnotes

^1^ https://card.mcmaster.ca/download

^2^ http://www.mgc.ac.cn/cgi-bin/VFs/v5/main.cgi
