# Supplementary material for: Whole-genome analysis showed the promotion of genetic diversity and coevolution in Staphylococcus aureus lytic bacteriophages and their hosts mediated by prophages via worldwide recombination events
Source: Front Microbiol. 2023 Mar 10;14:1088125. doi: 10.3389/fmicb.2023.1088125 (PMC10036374; doi:10.3389/fmicb.2023.1088125)
Supplement: Supplementary file 1 [file Data_Sheet_1.docx]

**Supplementary data**

Whole-genome analysis showed the promotion of genetic diversity and coevolution in *Staphylococcus aureus* lytic bacteriophages and their hosts mediated by prophages via worldwide recombination events

Wenyuan Zhou^1,2^, Yajie Li^1^, Xuechao Xu^1^, Shengqi Rao^1^, Hua Wen^1^, Yeiling Han^1^, Aiping Deng^1^, Zhenwen Zhang^3^, Zhenquan Yang^1*^, Guoqiang Zhu^2*^

^1^ College of Food Science and Engineering, Yangzhou University, Yangzhou, Jiangsu 225127, China

^2^ College of Veterinary Medicine, Yangzhou University, Yangzhou, Jiangsu 225009, China

^3^ Medical College, Yangzhou University, Yangzhou, Jiangsu 225009, China


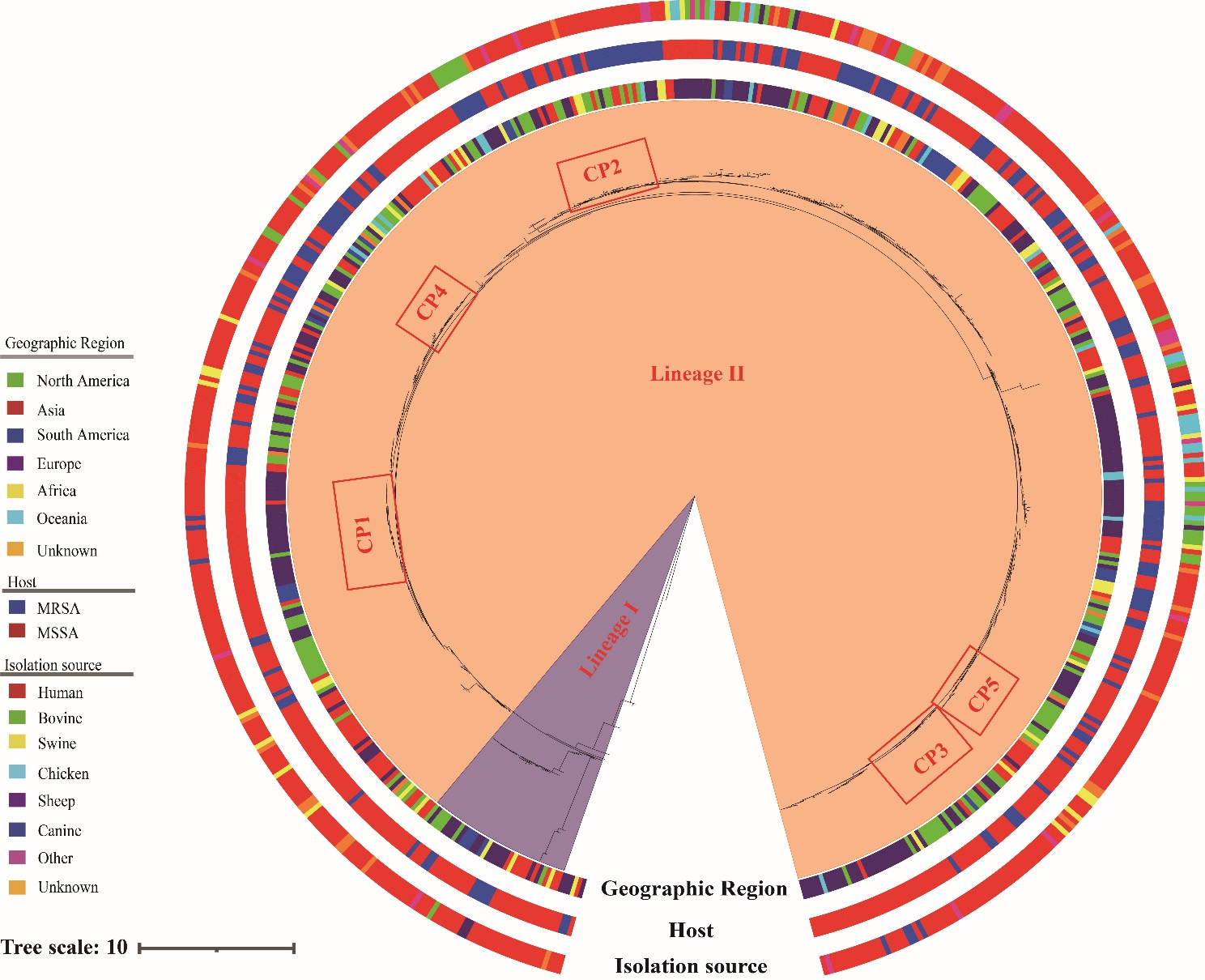


Figure S1. Phylogeny of 579 *S. aureus* intact prophages and the Erwinia phage, phiEa2809, based on 49,189 single-nucleotide polymorphisms. The inner ring is colored according to the geographic region; the middle ring, according to the host; and the outer ring, according to the isolation source.


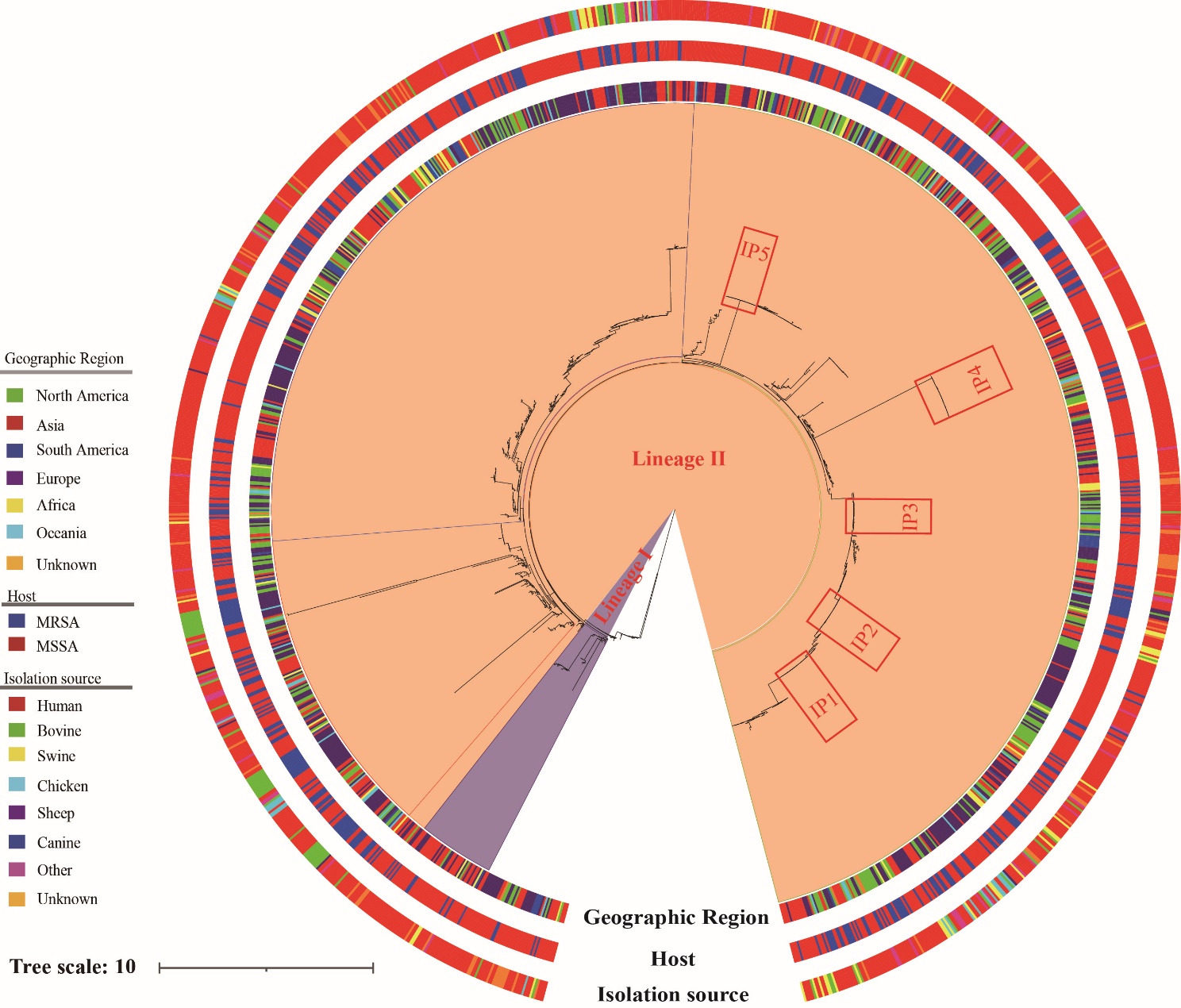


Figure S2. Phylogeny of 1389 *S. aureus* incomplete prophages and the Erwinia phage, phiEa2809, based on 292,450 single-nucleotide polymorphisms. The inner ring is colored according to the geographic region; the middle ring, according to the host; and the outer ring, according to the isolation source.


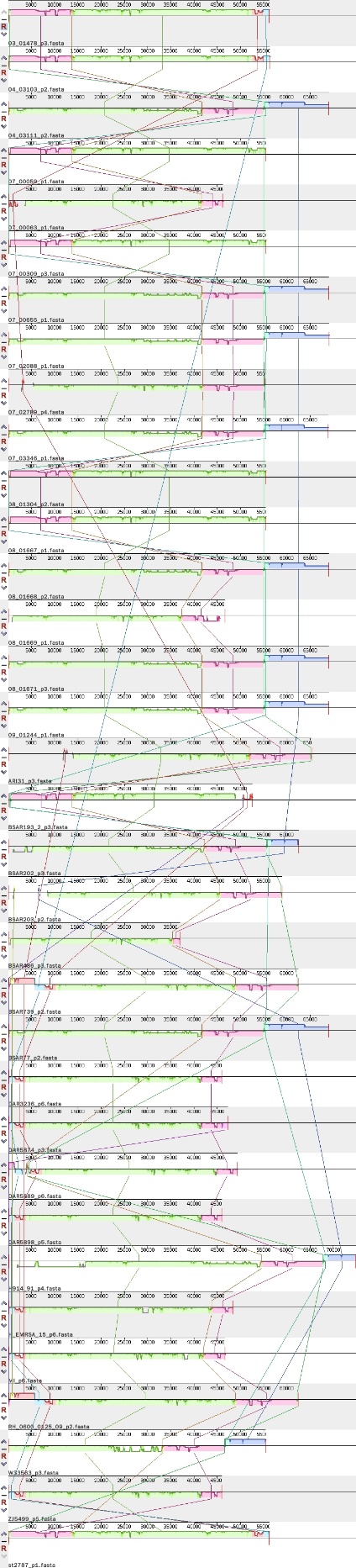


Figure S3. MARVE analysis of genomes of 34 intact prophages in group 1 (CP1).


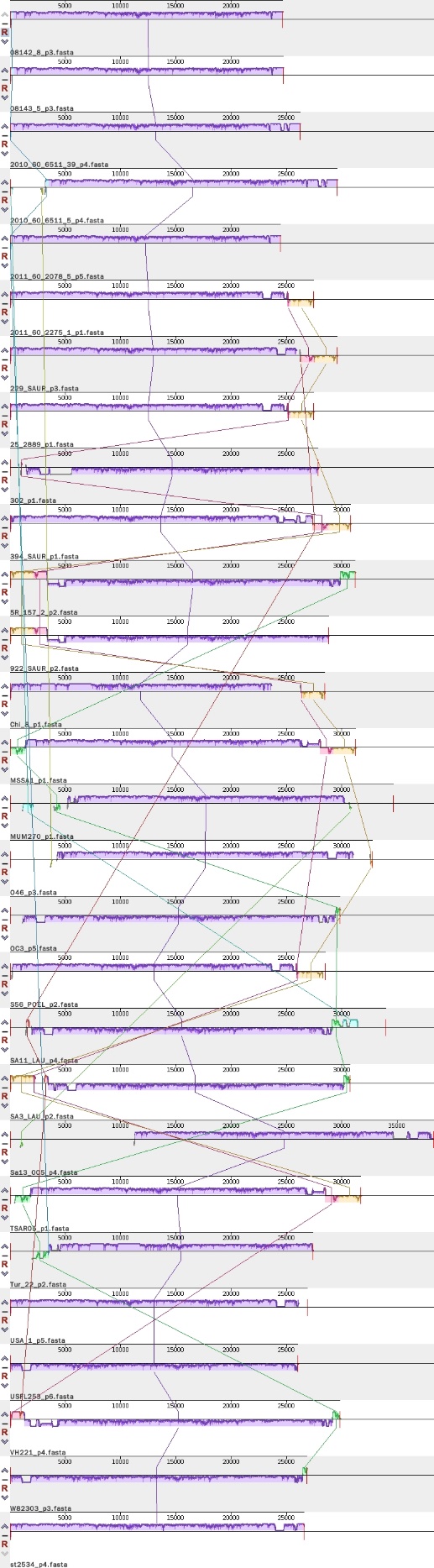


Figure S4. MARVE analysis of genomes of 27 intact prophages in group 2 (CP2).


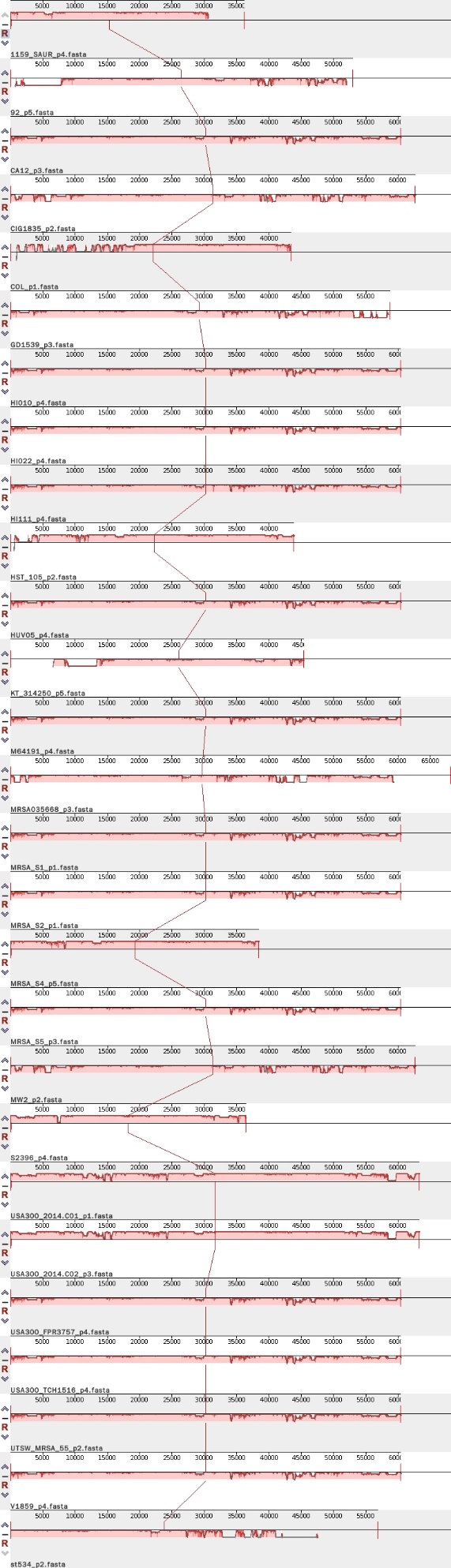


Figure S5. MARVE analysis of genomes of 27 intact prophages in group 3 (CP3).


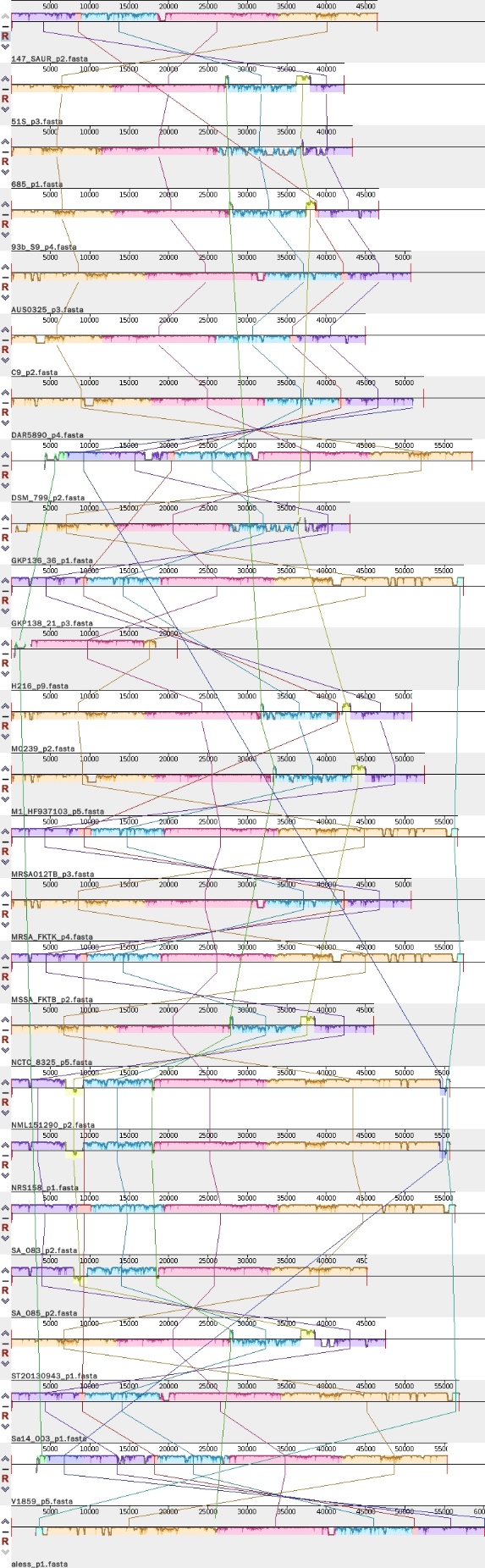


Figure S6. MARVE analysis of genomes of 24 intact prophages in group 4 (CP4).


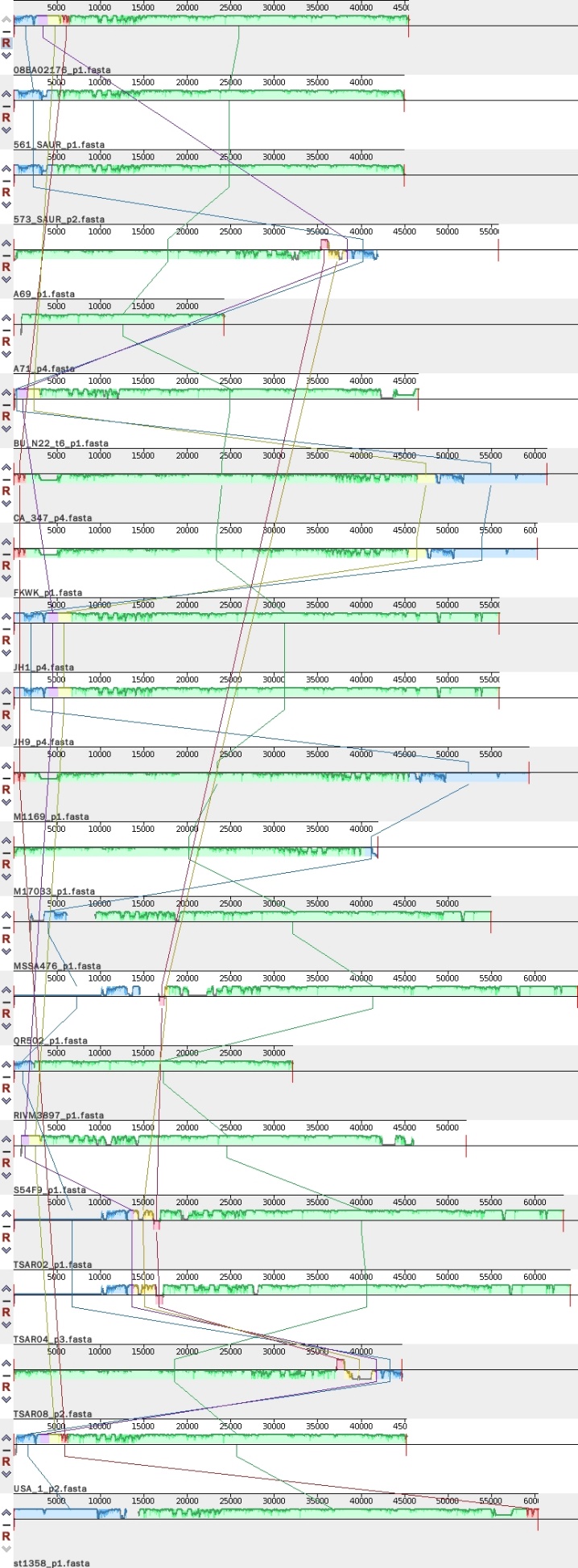


Figure S7. MARVE analysis of genomes of 21 intact prophages in group 5 (CP5).


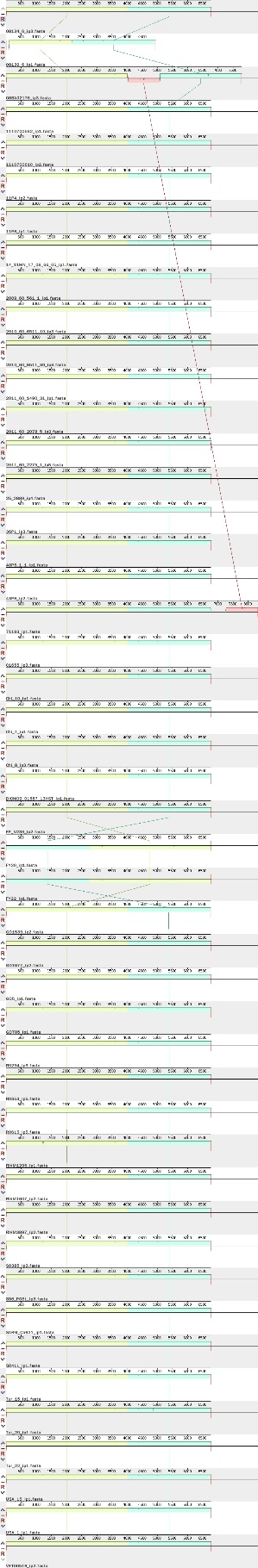


Figure S8. MARVE analysis of genomes of 47 incomplete prophages in group 1 (IP1).


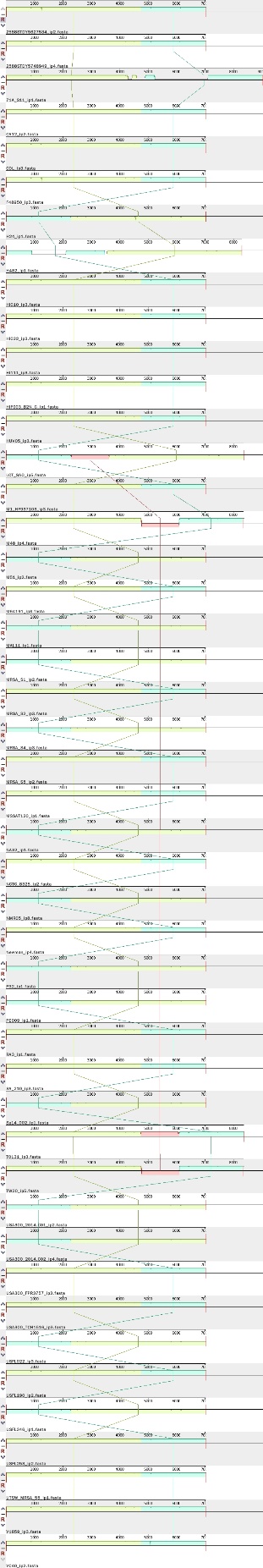


Figure S9. MARVE analysis of genomes of 44 incomplete prophages in group 2 (IP2).


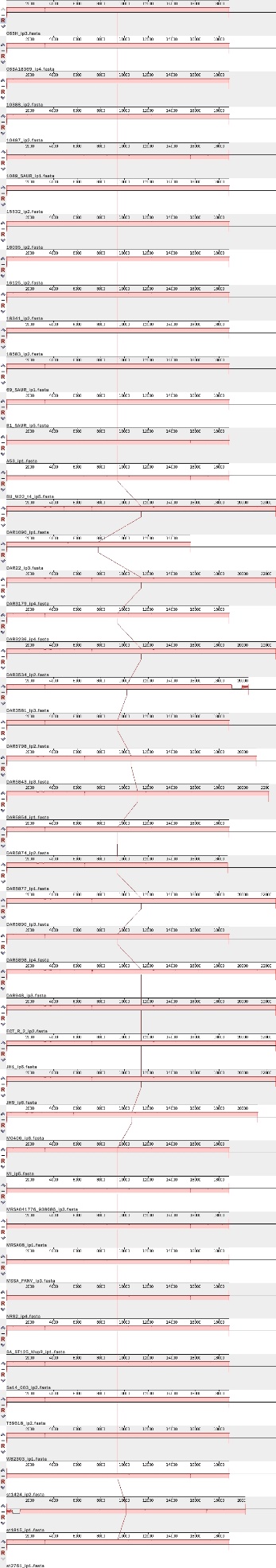


Figure S10. MARVE analysis of genomes of 44 incomplete prophages in group 3 (IP3).


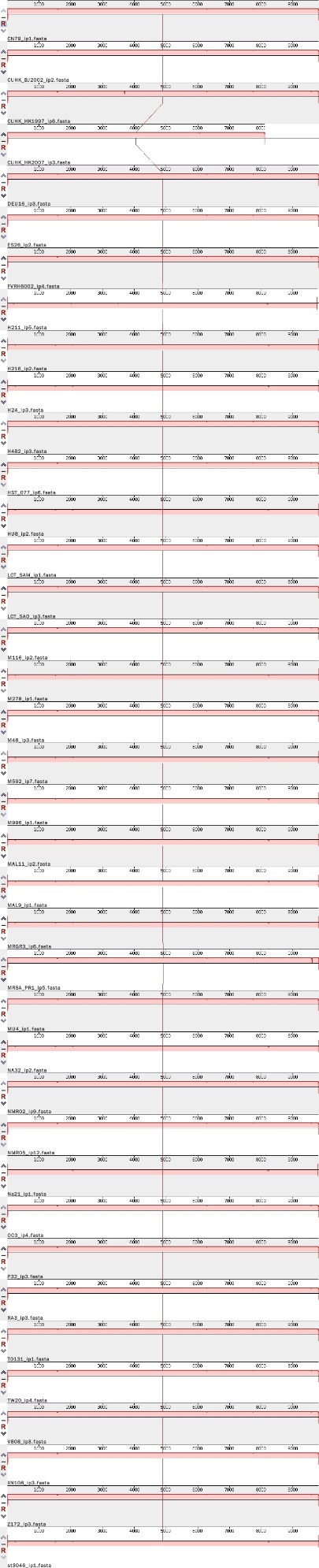


Figure S11. MARVE analysis of genomes of 38 incomplete prophages in group 4 (IP4).


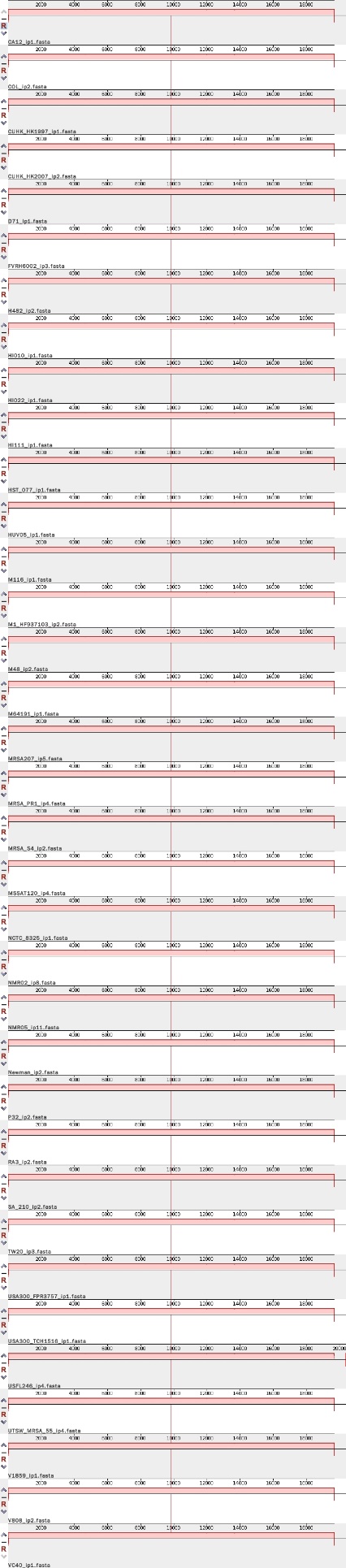


Figure S12. MARVE analysis of genomes of 35 incomplete prophages in group 5 (IP5).


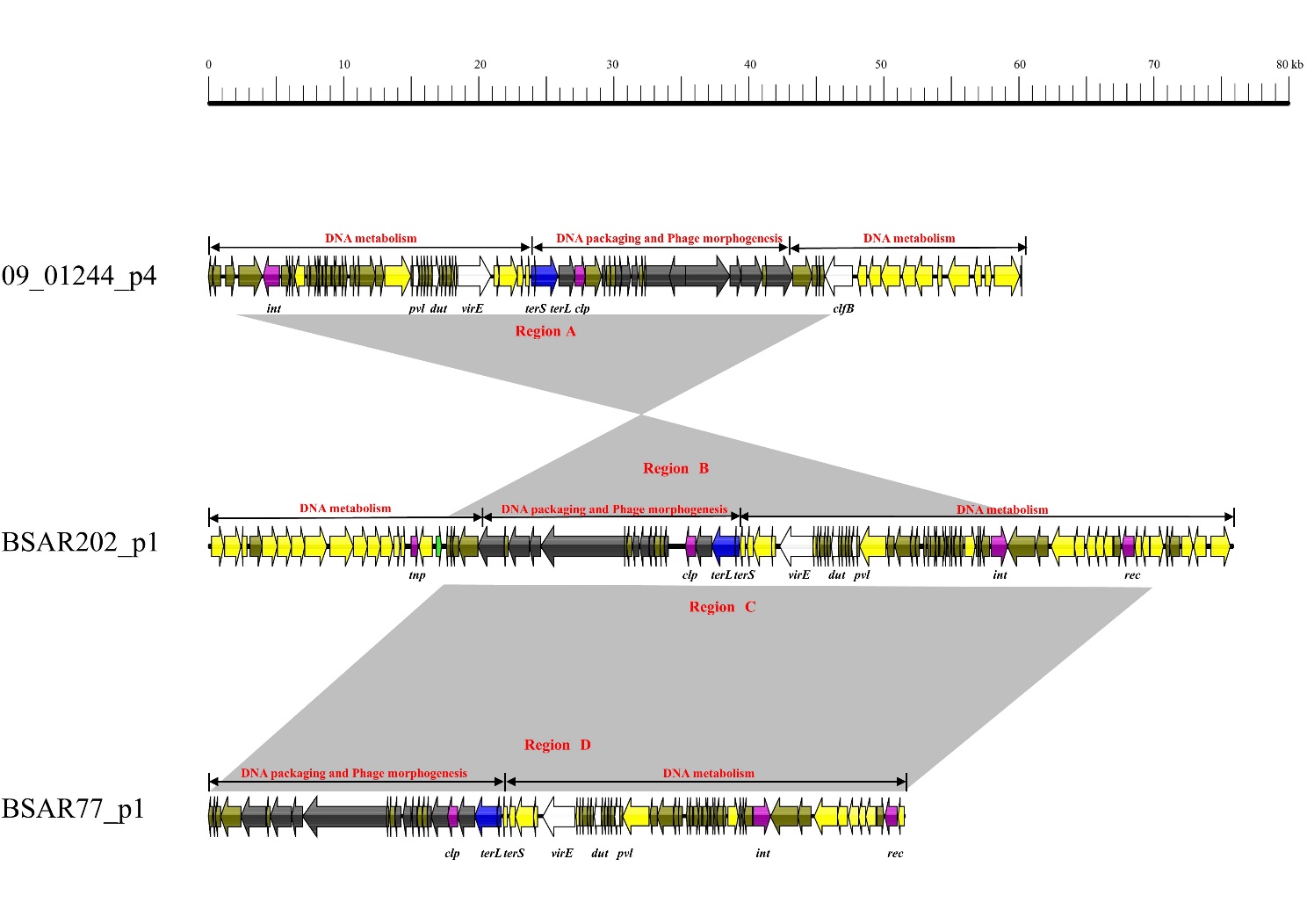


Figure S13. Comparative structural analysis of intact prophage 09_01244_p4 against intact prophages BSAR202_p1 and BSAR77_p1 of the *S. aureus*. Areas shaded in gray represent regions with >99% nucleotide sequence identity.


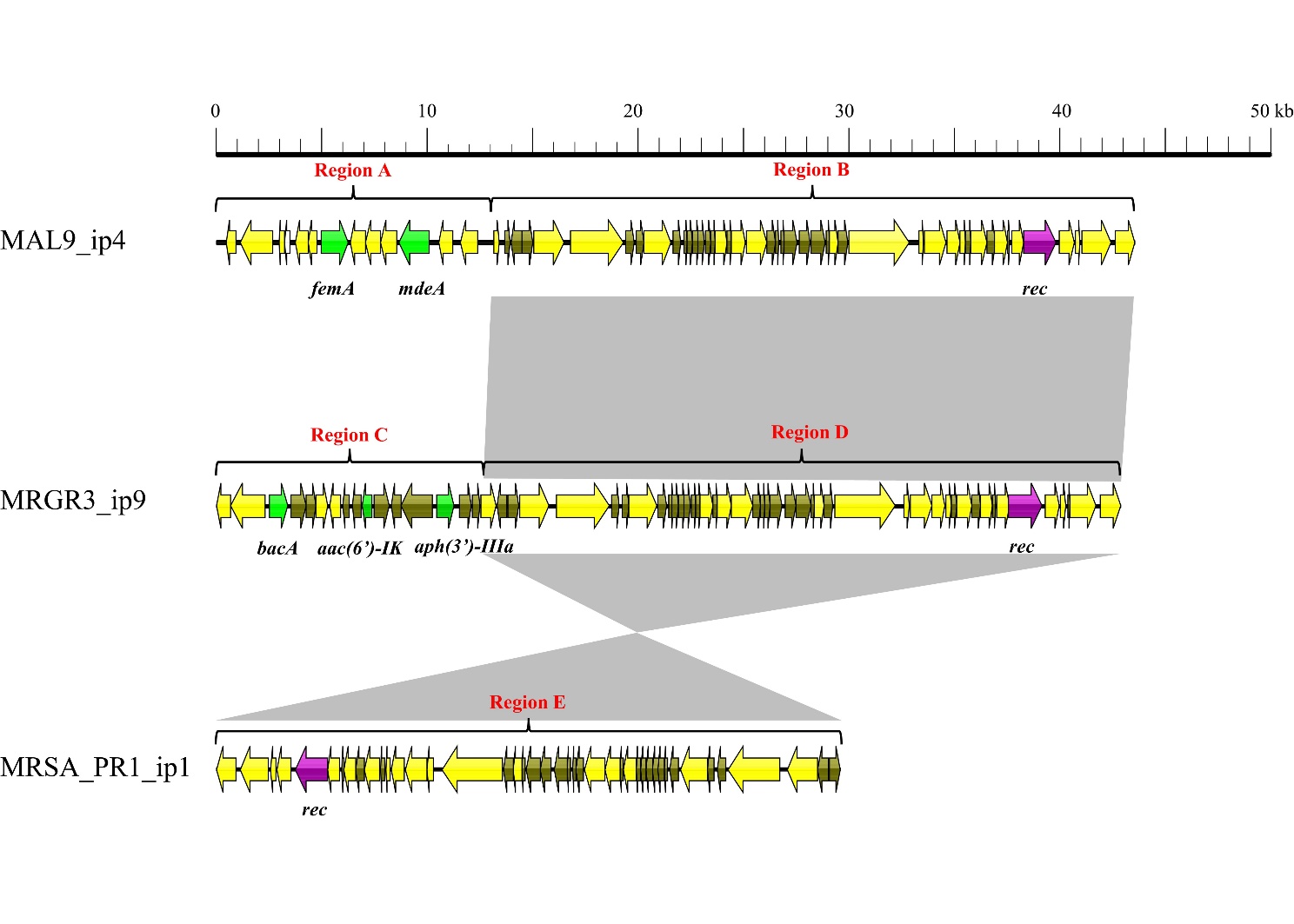


Figure S14. Comparative structural analysis of incomplete prophage MAL9_ip4 against intact prophages MRGR3_ip9 and MRSA_PR1_ip1 of the *S. aureus*. Areas shaded in gray represent regions with >98% nucleotide sequence identity.

Table S1 **Metadata for** **494 isolates publicly available on NCBI.** 494 isolates comprises two data sets (493 previously published genome sequences of *S. aureus* isolates, and *Staphylococcus simiae* CCM 7213). These 493 sequences include 349 *S. aureus* isolates from human origin; 39 *S. aureus* isolates from bovine origin; 20 *S. aureus* isolates from swine origin; 12 *S. aureus* isolate from poultry origin; 5 *S. aureus* isolates from sheep origin; 5 *S. aureus* isolates from canine origin; 27 *S. aureus* isolates from other origins, and 36 *S. aureus* isolates from unknown origin. The isolates are isolated from the North America, South America, Asia, Europe, Africa, Oceania, Space Station and unknown location.

| **Strain** | **Geographic region** | **MRSA or MSSA** | **Host** | **Intact Phages** | **Incomplete Phages** | **GenBank Accession** | **Database** |
| --- | --- | --- | --- | --- | --- | --- | --- |
| IF7SW_P3 | Space Station | MSSA | Other | 1 | 3 | NZ_MIZM00000000 | NCBI |
| SA_120 | Switzerland | MSSA | Other | 1 | 3 | NZ_JXIG00000000 | NCBI |
| GKP136_64 | United Kingdom | MRSA | Bovine | 1 | 1 | NZ_FMRT00000000 | NCBI |
| GKP136_74 | United Kingdom | MRSA | Bovine | 0 | 3 | NZ_FMPZ00000000 | NCBI |
| MRSA207 | Tanzania | MRSA | Unknown | 2 | 2 | NZ_FMOP00000000 | NCBI |
| 08_02300 | Germany | MSSA | Human | 1 | 2 | NZ_CP015646 | NCBI |
| 08_02119 | Germany | MSSA | Human | 2 | 2 | NZ_CP015645 | NCBI |
| MCRF184 | United State | MSSA | Human | 0 | 2 | NZ_CP014791 | NCBI |
| RIVM1295 | Netherlands | MRSA | Human | 1 | 1 | NZ_CP013616 | NCBI |
| ST20130943 | Brazil | MSSA | Human | 1 | 0 | NZ_CP012974 | NCBI |
| HUV05 | United State | MRSA | Human | 2 | 3 | NZ_CP007676 | NCBI |
| CA12 | United State | MRSA | Human | 2 | 2 | NZ_CP007672 | NCBI |
| H_EMRSA_15 | Belgium | MRSA | Human | 2 | 4 | NZ_CP007659 | NCBI |
| V2200 | Venezuela | MRSA | Human | 1 | 4 | NZ_CP007657 | NCBI |
| S2398 | France | MSSA | Human | 1 | 5 | NZ_CAVW000000000 | NCBI |
| MI | United State | MRSA | Human | 3 | 3 | NZ_AP017320 | NCBI |
| 08BA02176 | Canada | MRSA | Human | 1 | 2 | NC_018608 | NCBI |
| NCTC_8325 | Unknown | MSSA | Unknown | 3 | 2 | NC_007795 | NCBI |
| IF6SW_P2_RA | Space Station | MSSA | Other | 1 | 2 | MIZK00000000 | NCBI |
| IF6SW_P2 | Space Station | MSSA | Other | 2 | 2 | MIZI00000000 | NCBI |
| NML151290 | Canada | MSSA | Human | 2 | 0 | MEGZ00000000 | NCBI |
| 5R_157_2 | Mexico | MSSA | Human | 1 | 2 | MCFM00000000 | NCBI |
| Sa14_004 | Australia | MSSA | Bovine | 1 | 2 | MAQR00000000 | NCBI |
| Sa14_003 | Australia | MSSA | Bovine | 1 | 2 | MAQQ00000000 | NCBI |
| Sa14_002 | Australia | MSSA | Bovine | 1 | 2 | MAQP00000000 | NCBI |
| Sa13_006 | Australian | MSSA | Bovine | 2 | 3 | MAQN00000000 | NCBI |
| Sa12_002 | Australia | MSSA | Bovine | 0 | 4 | MAQH00000000 | NCBI |
| Sa12_001 | Australia | MSSA | Bovine | 1 | 3 | MAQG00000000 | NCBI |
| FP_SA_ST25 | Unknown | MSSA | Other | 0 | 0 | LXFD00000000 | NCBI |
| VB26276 | India | MSSA | Human | 1 | 3 | LWMF00000000 | NCBI |
| NMR05 | India | MSSA | Human | 1 | 11 | LWAL00000000 | NCBI |
| NMR02 | India | MSSA | Human | 0 | 9 | LWAK00000000 | NCBI |
| A53 | Brazil | MSSA | Other | 0 | 1 | LVID00000000 | NCBI |
| AUS0325 | Australia | MSSA | Human | 2 | 1 | LT615218 | NCBI |
| HUK16 | France | MSSA | Other | 0 | 3 | LSMV00000000 | NCBI |
| MS4_T60 | China | MRSA | Unknown | 1 | 2 | LSBE00000000 | NCBI |
| MS4_T10 | China | MRSA | Unknown | 1 | 0 | LSBD00000000 | NCBI |
| 685 | Italy | MSSA | Bovine | 1 | 0 | LRNB00000000 | NCBI |
| 120 | Belgium | MSSA | Bovine | 1 | 1 | LRMZ00000000 | NCBI |
| 299 | Belgium | MSSA | Bovine | 1 | 2 | LRMY00000000 | NCBI |
| 302 | Brazil | MSSA | Bovine | 1 | 3 | LNOR00000000 | NCBI |
| 170 | Brazil | MSSA | Bovine | 1 | 5 | LNOQ00000000 | NCBI |
| 1364 | Brazil | MSSA | Bovine | 0 | 1 | LNOP00000000 | NCBI |
| 1269 | Brazil | MSSA | Bovine | 0 | 0 | LNOO00000000 | NCBI |
| BB155 | Mali | MSSA | Human | 2 | 0 | LN854556 | NCBI |
| HOU1445_VS | Brazil | MSSA | Human | 0 | 5 | LJOC00000000 | NCBI |
| BA01611 | China | MRSA | Bovine | 2 | 4 | LIRC00000000 | NCBI |
| S54F9 | Denmark | MSSA | Swine | 1 | 2 | LIPH00000000 | NCBI |
| CC169 | Brazil | MRSA | Human | 0 | 3 | LIGU00000000 | NCBI |
| CC022 | Brazil | MRSA | Human | 1 | 3 | LIGS00000000 | NCBI |
| ATCC_29213 | Unknown | MSSA | Unknown | 1 | 3 | LHUS00000000 | NCBI |
| MRSA_S5 | Switzerland | MRSA | Human | 2 | 3 | LFUX00000000 | NCBI |
| MRSA_S4 | Switzerland | MRSA | Human | 2 | 4 | LFUW00000000 | NCBI |
| MRSA_S2 | Switzerland | MRSA | Human | 2 | 3 | LFUV00000000 | NCBI |
| MRSA_S1 | Switzerland | MRSA | Human | 2 | 3 | LFUU00000000 | NCBI |
| BU_W12_t13 | Ghana | MSSA | Human | 1 | 4 | LFTT00000000 | NCBI |
| BU_G1074_t4 | Ghana | MSSA | Human | 2 | 0 | LFOH00000000 | NCBI |
| BU_G0301_t8 | Ghana | MSSA | Human | 2 | 0 | LFOG00000000 | NCBI |
| BU_N22_t6 | Ghana | MSSA | Human | 3 | 0 | LFNS00000000 | NCBI |
| BU_W22_t4 | Ghana | MSSA | Human | 0 | 5 | LFNK00000000 | NCBI |
| BU_G0201_t8 | Ghana | MRSA | Human | 2 | 4 | LFMG00000000 | NCBI |
| V808 | Korea | MRSA | Human | 1 | 7 | LFED00000000 | NCBI |
| V040 | Korea | MRSA | Human | 0 | 0 | LFEC00000000 | NCBI |
| V1142 | Korea | MRSA | Human | 1 | 1 | LFEB00000000 | NCBI |
| V1127 | Korea | MRSA | Human | 1 | 7 | LFEA00000000 | NCBI |
| C9 | Australia | MSSA | Human | 1 | 1 | LDVH00000000 | NCBI |
| A5 | Australia | MSSA | Human | 1 | 4 | LDVF00000000 | NCBI |
| PE009 | United State | MRSA | Human | 0 | 3 | LAMO00000000 | NCBI |
| V1859 | Venezuela | MRSA | Human | 2 | 3 | LALU00000000 | NCBI |
| Mq2T | India | MSSA | Human | 1 | 2 | LAKI00000000 | NCBI |
| 112 | Finland | MSSA | Bovine | 0 | 3 | JZIQ00000000 | NCBI |
| 110 | Finland | MSSA | Bovine | 0 | 4 | JZIP00000000 | NCBI |
| 75 | Finland | MSSA | Bovine | 0 | 3 | JZIO00000000 | NCBI |
| 9 | Finland | MSSA | Bovine | 0 | 1 | JZIN00000000 | NCBI |
| P151 | Malaysia | MRSA | Human | 1 | 7 | JYFO00000000 | NCBI |
| SCCmec1 | Denmark | MRSA | Human | 3 | 3 | JYAW00000000 | NCBI |
| NRS2 | Japan | MRSA | Human | 3 | 4 | JYAO00000000 | NCBI |
| NRS158 | France | MSSA | Human | 2 | 1 | JYAN00000000 | NCBI |
| APS211 | Australia | MRSA | Human | 0 | 6 | JXUV00000000 | NCBI |
| APS210 | Australia | MRSA | Human | 1 | 7 | JXUD00000000 | NCBI |
| aless | Brazil | MSSA | Human | 1 | 2 | JXOB00000000 | NCBI |
| PB1_1 | United Kingdom | MRSA | Human | 0 | 3 | JXOA00000000 | NCBI |
| SA_210 | Switzerland | MSSA | Other | 1 | 3 | JXIH00000000 | NCBI |
| SA_085 | Switzerland | MSSA | Other | 2 | 1 | JXIF00000000 | NCBI |
| SA_083 | Switzerland | MSSA | Other | 2 | 2 | JXIE00000000 | NCBI |
| SA_067 | Switzerland | MSSA | Other | 1 | 2 | JXID00000000 | NCBI |
| SA_260 | Switzerland | MSSA | Other | 1 | 5 | JXIB00000000 | NCBI |
| FRI137 | United State | MSSA | Human | 1 | 4 | JXHX00000000 | NCBI |
| DSM_799 | United State | MSSA | Human | 1 | 3 | JXHV00000000 | NCBI |
| A900624 | France | MSSA | Human | 0 | 2 | JXHT00000000 | NCBI |
| 1089_SAUR | United State | MSSA | Human | 1 | 3 | JWDJ00000000 | NCBI |
| 1158_SAUR | United State | MSSA | Human | 1 | 1 | JWAY00000000 | NCBI |
| 1159_SAUR | United State | MSSA | Human | 1 | 5 | JWAX00000000 | NCBI |
| 1322_2_SAUR | United State | MSSA | Human | 1 | 1 | JVTT00000000 | NCBI |
| 147_SAUR | United State | MSSA | Human | 2 | 0 | JVSK00000000 | NCBI |
| 169_SAUR | United State | MSSA | Human | 1 | 1 | JVRM00000000 | NCBI |
| 229_SAUR | United State | MSSA | Human | 1 | 3 | JVPF00000000 | NCBI |
| 394_SAUR | United State | MSSA | Human | 1 | 3 | JVIV00000000 | NCBI |
| 511_SAUR | United State | MSSA | Human | 1 | 1 | JVEF00000000 | NCBI |
| 561_SAUR | United State | MSSA | Human | 1 | 0 | JVCB00000000 | NCBI |
| 573_SAUR | United State | MSSA | Human | 1 | 2 | JVBM00000000 | NCBI |
| 69_SAUR | United State | MSSA | Human | 1 | 2 | JUWZ00000000 | NCBI |
| 81_SAUR | United State | MRSA | Human | 0 | 3 | JURZ00000000 | NCBI |
| 82_SAUR | United State | MSSA | Human | 1 | 0 | JURO00000000 | NCBI |
| 922_SAUR | United State | MSSA | Human | 1 | 4 | JUNM00000000 | NCBI |
| 975_SAUR | United State | MRSA | Human | 0 | 3 | JULK00000000 | NCBI |
| UAMS_1 | United State | MSSA | Human | 1 | 2 | JTJK00000000 | NCBI |
| 42S | Pakistan | MRSA | Human | 1 | 1 | JTFJ00000000 | NCBI |
| 51S | Pakistan | MRSA | Human | 1 | 2 | JSAJ00000000 | NCBI |
| 337 | Malaysia | MSSA | Human | 1 | 2 | JQIZ00000000 | NCBI |
| NGS_ED_1006 | United Kingdom | MSSA | Human | 1 | 2 | JPWO00000000 | NCBI |
| 08134_6 | Belgium | MRSA | Other | 1 | 2 | JJEQ00000000 | NCBI |
| 08139_6 | Netherlands | MSSA | Other | 0 | 2 | JJEP00000000 | NCBI |
| 08142_8 | Netherlands | MRSA | Other | 1 | 2 | JJEO00000000 | NCBI |
| 08143_5 | Netherlands | MRSA | Other | 1 | 2 | JJEN00000000 | NCBI |
| 1110700562 | Netherlands | MSSA | Human | 1 | 1 | JJED00000000 | NCBI |
| 1110700610 | Netherlands | MRSA | Human | 1 | 1 | JJEC00000000 | NCBI |
| 11P4 | Netherlands | MRSA | Swine | 1 | 1 | JJDM00000000 | NCBI |
| 11P8 | Netherlands | MRSA | Swine | 0 | 2 | JJDL00000000 | NCBI |
| 14_11MN_17_08_66_05 | Netherlands | MRSA | Human | 0 | 1 | JJCT00000000 | NCBI |
| 18754_2 | Italy | MRSA | Swine | 1 | 5 | JJCN00000000 | NCBI |
| 2009_60_561_1 | Germany | MRSA | Chicken | 1 | 2 | JJCK00000000 | NCBI |
| 2010_60_6511_10 | Netherlands | MRSA | Bovine | 2 | 3 | JJCG00000000 | NCBI |
| 2010_60_6511_39 | Germany | MRSA | Chicken | 2 | 2 | JJCF00000000 | NCBI |
| 2010_60_6511_5 | Netherlands | MRSA | Bovine | 1 | 3 | JJCE00000000 | NCBI |
| 2011_60_1490_31 | Germany | MRSA | Chicken | 1 | 2 | JJCC00000000 | NCBI |
| 2011_60_2078_5 | Netherlands | MRSA | Bovine | 2 | 3 | JJCB00000000 | NCBI |
| 2011_60_2275_1 | Germany | MRSA | Chicken | 2 | 4 | JJBZ00000000 | NCBI |
| 25_2889 | Netherlands | MSSA | Human | 2 | 4 | JJBL00000000 | NCBI |
| 36P1 | Netherlands | MSSA | Swine | 1 | 2 | JJBH00000000 | NCBI |
| 40P5_1_1 | Netherlands | MSSA | Swine | 0 | 2 | JJBD00000000 | NCBI |
| 43P8 | Netherlands | MSSA | Swine | 1 | 2 | JJBB00000000 | NCBI |
| C1655 | Spain | MRSA | Swine | 1 | 2 | JIZQ00000000 | NCBI |
| Chi_10 | Austria | MRSA | Chicken | 1 | 2 | JIYV00000000 | NCBI |
| Chi_4 | Germany | MRSA | Chicken | 1 | 2 | JIYU00000000 | NCBI |
| Chi_8 | Germany | MRSA | Chicken | 2 | 3 | JIYT00000000 | NCBI |
| DICM09_01587_13HST | Spain | MRSA | Sheep | 1 | 2 | JIYR00000000 | NCBI |
| FP_N239 | Netherlands | MRSA | Bovine | 1 | 1 | JIYQ00000000 | NCBI |
| R0294 | China | MSSA | Other | 2 | 6 | JIXH00000000 | NCBI |
| R0353 | China | MSSA | Other | 2 | 5 | JIXG00000000 | NCBI |
| R0615 | China | MSSA | Other | 0 | 6 | JIXB00000000 | NCBI |
| Rd_3 | Germany | MRSA | Bovine | 1 | 1 | JIWZ00000000 | NCBI |
| S56_POEL | Belgium | MRSA | Chicken | 2 | 5 | JIWS00000000 | NCBI |
| SARM_C5621 | Spain | MRSA | Human | 1 | 2 | JIWM00000000 | NCBI |
| Tur_15 | Austria | MRSA | Chicken | 1 | 3 | JIWB00000000 | NCBI |
| Tur_20 | Germany | MRSA | Chicken | 1 | 1 | JIVZ00000000 | NCBI |
| Tur_22 | Italy | MRSA | Chicken | 1 | 2 | JIVY00000000 | NCBI |
| USA_1 | United State | MSSA | Human | 2 | 3 | JIVS00000000 | NCBI |
| USA_15 | United State | MSSA | Human | 2 | 1 | JIVP00000000 | NCBI |
| VET0051R | Netherlands | MRSA | Human | 1 | 1 | JIVF00000000 | NCBI |
| VET0889S | Netherlands | MSSA | Human | 1 | 2 | JIJO00000000 | NCBI |
| VET1518S | Netherlands | MSSA | Human | 1 | 2 | JIIM00000000 | NCBI |
| SA11_LAU | Jordan | MSSA | Human | 1 | 4 | JHEC00000000 | NCBI |
| SA8_LAU | Jordan | MSSA | Human | 1 | 4 | JHDZ00000000 | NCBI |
| SA3_LAU | Lebanon | MSSA | Human | 1 | 5 | JHDV00000000 | NCBI |
| DAR5889 | Peru | MRSA | Human | 5 | 2 | JGUJ00000000 | NCBI |
| DAR5890 | Venezuela | MRSA | Human | 4 | 3 | JGUI00000000 | NCBI |
| DAR3236 | Argentina | MRSA | Human | 3 | 3 | JGUH00000000 | NCBI |
| DAR3179 | Argentina | MRSA | Human | 1 | 5 | JGUE00000000 | NCBI |
| DAR1090 | United State | MSSA | Human | 0 | 2 | JGRI00000000 | NCBI |
| DAR5874 | Colombia | MRSA | Human | 3 | 2 | JGQY00000000 | NCBI |
| DAR5877 | Peru | MRSA | Human | 1 | 4 | JGQW00000000 | NCBI |
| DAR22 | Belgium | MRSA | Human | 2 | 2 | JGQU00000000 | NCBI |
| DAR3581 | United State | MRSA | Human | 1 | 2 | JGOF00000000 | NCBI |
| DAR3534 | United State | MRSA | Human | 1 | 2 | JGNU00000000 | NCBI |
| T59618 | United State | MRSA | Human | 1 | 1 | JGKS00000000 | NCBI |
| M17033 | United State | MRSA | Human | 1 | 0 | JGJV00000000 | NCBI |
| F48950 | United State | MRSA | Human | 2 | 2 | JGJC00000000 | NCBI |
| M35954 | United State | MRSA | Human | 1 | 1 | JGIM00000000 | NCBI |
| W25799 | United State | MRSA | Human | 1 | 0 | JGIF00000000 | NCBI |
| W45755_111412 | United State | MRSA | Human | 1 | 12 | JGFP00000000 | NCBI |
| F19490 | United State | MRSA | Unknown | 0 | 2 | JGFB00000000 | NCBI |
| F26051 | United State | MRSA | Human | 1 | 1 | JFUO00000000 | NCBI |
| DAR5867 | Argentina | MSSA | Human | 1 | 3 | JFRK00000000 | NCBI |
| DAR5854 | Ecuador | MRSA | Human | 1 | 3 | JFRA00000000 | NCBI |
| DAR5843 | Peru | MRSA | Human | 2 | 1 | JFQR00000000 | NCBI |
| DAR5798 | Colombia | MRSA | Human | 0 | 3 | JFPC00000000 | NCBI |
| M1169 | United State | MSSA | Human | 1 | 2 | JEKD00000000 | NCBI |
| M1242 | United State | MRSA | Human | 1 | 1 | JEIN00000000 | NCBI |
| T44444 | United State | MRSA | Human | 1 | 2 | JEAR00000000 | NCBI |
| W33563 | United State | MRSA | Human | 2 | 2 | JDZZ00000000 | NCBI |
| W82303 | United State | MRSA | Human | 1 | 3 | JDNE00000000 | NCBI |
| F70893 | United State | MRSA | Human | 0 | 0 | JDLM00000000 | NCBI |
| M64191 | United State | MRSA | Human | 2 | 3 | JDJD00000000 | NCBI |
| M0821 | United State | MSSA | Human | 1 | 3 | JCPQ00000000 | NCBI |
| M0426 | United State | MRSA | Human | 1 | 6 | JCFL00000000 | NCBI |
| M0406 | United State | MRSA | Human | 2 | 6 | JCEY00000000 | NCBI |
| M0359 | United State | MSSA | Human | 0 | 4 | JCDZ00000000 | NCBI |
| DAR5898 | Venezuela | MRSA | Human | 3 | 4 | JBTA00000000 | NCBI |
| DAR948 | United State | MRSA | Human | 2 | 3 | JBSS00000000 | NCBI |
| FVRH6002 | United State | MRSA | Human | 1 | 6 | JBSL00000000 | NCBI |
| OCMM6035 | United State | MRSA | Human | 1 | 5 | JBNA00000000 | NCBI |
| KINW6056 | United State | MRSA | Human | 2 | 6 | JBFH00000000 | NCBI |
| M0684 | United State | MSSA | Human | 1 | 3 | JBCT00000000 | NCBI |
| M0690 | United State | MRSA | Human | 0 | 5 | JBCP00000000 | NCBI |
| M0715 | United State | MRSA | Human | 1 | 4 | JBBX00000000 | NCBI |
| M0745 | United State | MRSA | Human | 1 | 3 | JAYT00000000 | NCBI |
| M0896 | United State | MRSA | Human | 1 | 4 | JAYI00000000 | NCBI |
| MRSA_CVM43477 | United State | MRSA | Other | 0 | 1 | JANQ00000000 | NCBI |
| M1_HF937103 | Denmark | MRSA | Human | 3 | 2 | HF937103 | NCBI |
| 18583 | Switzerland | MRSA | Unknown | 0 | 2 | HE579073 | NCBI |
| 18412 | Switzerland | MRSA | Unknown | 2 | 0 | HE579071 | NCBI |
| 18341 | Switzerland | MRSA | Unknown | 0 | 2 | HE579069 | NCBI |
| 16125 | Switzerland | MRSA | Unknown | 0 | 2 | HE579067 | NCBI |
| 16035 | Switzerland | MRSA | Unknown | 0 | 2 | HE579065 | NCBI |
| 15532 | Switzerland | MRSA | Unknown | 0 | 2 | HE579063 | NCBI |
| 10497 | Switzerland | MRSA | Unknown | 0 | 2 | HE579061 | NCBI |
| 10388 | Switzerland | MRSA | Unknown | 0 | 2 | HE579059 | NCBI |
| LGA251 | United Kingdom | MRSA | Bovine | 1 | 0 | FR821779 | NCBI |
| ECT_R_2 | Sweden | MSSA | Human | 1 | 2 | FR714927 | NCBI |
| TW20 | United Kingdom | MRSA | Unknown | 3 | 5 | FN433596 | NCBI |
| MRSA012TB | Tanzania | MRSA | Unknown | 2 | 1 | FMSG00000000 | NCBI |
| MRSA012TA | Tanzania | MRSA | Unknown | 1 | 3 | FMPB00000000 | NCBI |
| GKP138_17 | United Kingdom | MSSA | Bovine | 2 | 5 | FMSB00000000 | NCBI |
| GKP136_68 | United Kingdom | MSSA | Bovine | 1 | 3 | FMSA00000000 | NCBI |
| GKP138_71 | United Kingdom | MSSA | Bovine | 1 | 4 | FMRI00000000 | NCBI |
| GKP136_12 | United Kingdom | MSSA | Bovine | 0 | 4 | FMQU00000000 | NCBI |
| GKP136_8 | United Kingdom | MSSA | Bovine | 2 | 1 | FMQS00000000 | NCBI |
| GKP136_67 | United Kingdom | MSSA | Bovine | 1 | 1 | FMQM00000000 | NCBI |
| GKP136_36 | United Kingdom | MSSA | Bovine | 1 | 2 | FMQK00000000 | NCBI |
| GKP136_21 | United Kingdom | MSSA | Bovine | 1 | 1 | FMQI00000000 | NCBI |
| GKP136_19 | United Kingdom | MSSA | Bovine | 1 | 5 | FMQF00000000 | NCBI |
| GKP138_21 | United Kingdom | MSSA | Bovine | 1 | 2 | FMQE00000000 | NCBI |
| GKP138_41 | United Kingdom | MSSA | Bovine | 1 | 0 | FMQC00000000 | NCBI |
| GKP136_11 | United Kingdom | MRSA | Bovine | 3 | 2 | FMPJ00000000 | NCBI |
| MRSA079A | Tanzania | MRSA | Unknown | 1 | 1 | FMPE00000000 | NCBI |
| MSSAT120 | Tanzania | MSSA | Unknown | 0 | 3 | FMPD00000000 | NCBI |
| MRSA_041T_B | Tanzania | MRSA | Unknown | 1 | 1 | FMOZ00000000 | NCBI |
| MSSA_T188 | Tanzania | MSSA | Unknown | 0 | 2 | FMOV00000000 | NCBI |
| MRSA08 | Tanzania | MRSA | Unknown | 2 | 2 | FMOS00000000 | NCBI |
| MRSA140 | Tanzania | MRSA | Unknown | 0 | 4 | FMOO00000000 | NCBI |
| MRSA071 | Tanzania | MRSA | Unknown | 1 | 2 | FMON00000000 | NCBI |
| 043H | Tanzania | MSSA | Unknown | 1 | 4 | FMOI00000000 | NCBI |
| MRSA041776_938686 | Tanzania | MRSA | Unknown | 0 | 4 | FMNU00000000 | NCBI |
| 04Hi | Tanzania | MSSA | Unknown | 2 | 0 | FMNR00000000 | NCBI |
| 065H | Tanzania | MSSA | Unknown | 1 | 4 | FMNL00000000 | NCBI |
| MRSA035668 | Tanzania | MRSA | Unknown | 1 | 2 | FMMQ00000000 | NCBI |
| MRSA_FKYJ | United State | MRSA | Human | 1 | 3 | FKYJ00000000 | NCBI |
| MRSA_FKWK | United State | MRSA | Human | 3 | 4 | FKWK00000000 | NCBI |
| MRSA_FKWH | United State | MRSA | Human | 1 | 2 | FKWH00000000 | NCBI |
| MRSA_FKWA | United State | MRSA | Human | 0 | 2 | FKWA00000000 | NCBI |
| MRSA_FKVP | United State | MRSA | Human | 1 | 3 | FKVP00000000 | NCBI |
| MRSA_FKUV | United State | MRSA | Human | 1 | 2 | FKUV00000000 | NCBI |
| MSSA_FKTY | United State | MSSA | Human | 0 | 1 | FKTY00000000 | NCBI |
| MRSA_FKTN | United State | MRSA | Human | 1 | 4 | FKTN00000000 | NCBI |
| MRSA_FKTK | United State | MRSA | Human | 1 | 2 | FKTK00000000 | NCBI |
| MSSA_FKTB | United State | MSSA | Human | 1 | 1 | FKTB00000000 | NCBI |
| MRSA_FKSE | United State | MRSA | Human | 2 | 2 | FKSE00000000 | NCBI |
| MSSA1 | United State | MSSA | Human | 1 | 3 | FKPS00000000 | NCBI |
| MRSA_FKPC | United State | MRSA | Human | 2 | 3 | FKPC00000000 | NCBI |
| MSSA_FKOF | United State | MSSA | Human | 1 | 2 | FKOF00000000 | NCBI |
| MSSA_FKNV | United State | MSSA | Human | 2 | 2 | FKNV00000000 | NCBI |
| 1943STDY5698363 | United Kingdom | MSSA | Other | 1 | 3 | FJNV00000000 | NCBI |
| 1943STDY5573748 | United Kingdom | MSSA | Unknown | 1 | 0 | FJNT00000000 | NCBI |
| 1943STDY5573749 | United Kingdom | MSSA | Unknown | 1 | 2 | FJNN00000000 | NCBI |
| st3047 | United Kingdom | MRSA | Human | 2 | 1 | FHER00000000 | NCBI |
| st1815 | Ireland | MRSA | Human | 1 | 3 | FHCI00000000 | NCBI |
| st3046 | United Kingdom | MRSA | Human | 0 | 9 | FGZX00000000 | NCBI |
| st2787 | United Kingdom | MRSA | Human | 1 | 2 | FGYQ00000000 | NCBI |
| st2761 | United Kingdom | MRSA | Human | 0 | 2 | FGYL00000000 | NCBI |
| st2543 | United Kingdom | MRSA | Human | 2 | 2 | FGXM00000000 | NCBI |
| st2436 | United Kingdom | MRSA | Human | 1 | 4 | FGWU00000000 | NCBI |
| st1831 | Ireland | MRSA | Human | 0 | 5 | FGTG00000000 | NCBI |
| st1358 | Ireland | MRSA | Human | 3 | 0 | FGQY00000000 | NCBI |
| st1335 | United Kingdom | MRSA | Human | 1 | 4 | FGQX00000000 | NCBI |
| st1332 | United Kingdom | MSSA | Human | 0 | 2 | FGQW00000000 | NCBI |
| st424 | United Kingdom | MSSA | Human | 0 | 2 | FGOS00000000 | NCBI |
| st1624 | United Kingdom | MSSA | Human | 0 | 1 | FGNT00000000 | NCBI |
| st1607 | Ireland | MRSA | Human | 2 | 3 | FGNQ00000000 | NCBI |
| st1520 | United Kingdom | MSSA | Human | 1 | 3 | FGMR00000000 | NCBI |
| st1470 | United Kingdom | MSSA | Human | 1 | 0 | FGMD00000000 | NCBI |
| st1424 | United Kingdom | MSSA | Human | 1 | 1 | FGLO00000000 | NCBI |
| st534 | Ireland | MSSA | Human | 1 | 2 | FGAA00000000 | NCBI |
| st436 | United Kingdom | MRSA | Human | 1 | 4 | FFZB00000000 | NCBI |
| st2534 | United Kingdom | MRSA | Human | 2 | 6 | FFOY00000000 | NCBI |
| 2588STDY5748949 | United State | MRSA | Human | 0 | 5 | CYOL00000000 | NCBI |
| 2588STDY5627534 | United State | MSSA | Unknown | 0 | 3 | CYJS00000000 | NCBI |
| RH_0600_0125_09 | United Kingdom | MRSA | Human | 1 | 2 | CUCD00000000 | NCBI |
| ARI29 | United Kingdom | MRSA | Human | 1 | 5 | CUBZ00000000 | NCBI |
| H914_91 | United Kingdom | MRSA | Human | 3 | 3 | CUAK00000000 | NCBI |
| HU8 | Turkey | MRSA | Human | 1 | 7 | CTZP00000000 | NCBI |
| ARI31 | United Kingdom | MRSA | Human | 2 | 4 | CTZL00000000 | NCBI |
| DEU16 | Turkey | MRSA | Human | 1 | 5 | CTYV00000000 | NCBI |
| DEU23 | Turkey | MRSA | Human | 0 | 0 | CTYS00000000 | NCBI |
| DEU10 | Turkey | MRSA | Human | 0 | 0 | CTYJ00000000 | NCBI |
| MU4 | Turkey | MRSA | Human | 2 | 7 | CTYE00000000 | NCBI |
| H202 | Thailand | MRSA | Human | 1 | 9 | CTYD00000000 | NCBI |
| H216 | Denmark | MRSA | Human | 2 | 11 | CTXZ00000000 | NCBI |
| RA3 | Argentina | MRSA | Human | 2 | 6 | CTXW00000000 | NCBI |
| P32 | Poland | MRSA | Human | 1 | 7 | CTXV00000000 | NCBI |
| M592 | Syria | MRSA | Human | 2 | 10 | CTXT00000000 | NCBI |
| M418 | India | MRSA | Human | 0 | 0 | CTXS00000000 | NCBI |
| M116 | Viet Nam | MRSA | Human | 3 | 9 | CTXQ00000000 | NCBI |
| M278 | Portugal | MRSA | Human | 1 | 4 | CTXP00000000 | NCBI |
| Na21 | Sri Lanka | MRSA | Human | 1 | 13 | CTXN00000000 | NCBI |
| MAL11 | Malaysia | MRSA | Human | 2 | 7 | CTXM00000000 | NCBI |
| H482 | Romania | MRSA | Human | 1 | 7 | CTXK00000000 | NCBI |
| ES26 | Spain | MRSA | Human | 3 | 7 | CTXI00000000 | NCBI |
| D71 | Germany | MRSA | Human | 1 | 7 | CTXH00000000 | NCBI |
| MAL9 | Malaysia | MRSA | Human | 2 | 7 | CTWZ00000000 | NCBI |
| H211 | Denmark | MRSA | Human | 1 | 13 | CTWW00000000 | NCBI |
| H24 | Egypt | MRSA | Human | 3 | 5 | CTWS00000000 | NCBI |
| NA32 | Denmark | MRSA | Human | 2 | 7 | CTWR00000000 | NCBI |
| 04_03103 | Germany | MRSA | Human | 2 | 1 | CTVX00000000 | NCBI |
| 04_02314_1 | United Kingdom | MRSA | Human | 1 | 4 | CTVT00000000 | NCBI |
| 04_00608 | Germany | MRSA | Human | 2 | 2 | CTVS00000000 | NCBI |
| 03_01478 | Germany | MRSA | Human | 2 | 1 | CTVR00000000 | NCBI |
| 04_03111 | Germany | MRSA | Human | 2 | 1 | CTVQ00000000 | NCBI |
| 06_01900 | Germany | MRSA | Human | 1 | 4 | CTVN00000000 | NCBI |
| 07_00309 | Germany | MRSA | Human | 2 | 2 | CTVC00000000 | NCBI |
| 06_02400 | Germany | MRSA | Human | 0 | 2 | CTVB00000000 | NCBI |
| 07_00063 | Germany | MRSA | Human | 1 | 0 | CTVA00000000 | NCBI |
| 07_00058 | Germany | MRSA | Human | 0 | 3 | CTUW00000000 | NCBI |
| 07_00059 | Germany | MRSA | Human | 2 | 2 | CTUT00000000 | NCBI |
| 07_00655 | Germany | MRSA | Human | 1 | 3 | CTUR00000000 | NCBI |
| 07_02088 | Germany | MRSA | Human | 2 | 2 | CTUQ00000000 | NCBI |
| 07_02789 | Germany | MRSA | Human | 2 | 2 | CTUM00000000 | NCBI |
| 07_01497 | Germany | MRSA | Human | 0 | 1 | CTUK00000000 | NCBI |
| 07_02997 | Australia | MRSA | Human | 1 | 3 | CTTW00000000 | NCBI |
| 07_03339 | Czech Republic | MRSA | Human | 0 | 2 | CTTU00000000 | NCBI |
| 07_03345 | Czech Republic | MRSA | Human | 1 | 1 | CTTT00000000 | NCBI |
| 07_03346 | Czech Republic | MRSA | Human | 2 | 2 | CTTS00000000 | NCBI |
| 07_03349 | Denmark | MRSA | Human | 0 | 2 | CTTP00000000 | NCBI |
| 07_03350 | Denmark | MRSA | Human | 0 | 0 | CTTK00000000 | NCBI |
| 08_01669 | Denmark | MRSA | Human | 1 | 3 | CTTI00000000 | NCBI |
| 08_01483 | Portugal | MRSA | Human | 0 | 2 | CTTC00000000 | NCBI |
| 08_01671 | Germany | MRSA | Human | 2 | 2 | CTTA00000000 | NCBI |
| 08_01486 | Portugal | MRSA | Human | 0 | 4 | CTSV00000000 | NCBI |
| 08_01666 | Germany | MRSA | Human | 0 | 0 | CTSS00000000 | NCBI |
| 08_01667 | Germany | MRSA | Human | 1 | 2 | CTSP00000000 | NCBI |
| 09_01244 | Singapore | MRSA | Human | 2 | 2 | CTSM00000000 | NCBI |
| 09_01245 | Singapore | MRSA | Human | 0 | 6 | CTSI00000000 | NCBI |
| 08_01668 | Germany | MRSA | Human | 1 | 4 | CTSC00000000 | NCBI |
| 07_03354_2 | Denmark | MRSA | Human | 1 | 1 | CTSA00000000 | NCBI |
| 08_01304 | Germany | MRSA | Human | 1 | 2 | CTRY00000000 | NCBI |
| 08_01672 | Germany | MRSA | Human | 0 | 2 | CTRW00000000 | NCBI |
| BSAR858 | United Kingdom | MRSA | canine | 1 | 1 | CSSX00000000 | NCBI |
| BSAR203 | United Kingdom | MRSA | canine | 2 | 0 | CSSQ00000000 | NCBI |
| BSAR748 | United Kingdom | MRSA | Human | 1 | 3 | CSRY00000000 | NCBI |
| BSAR208 | United Kingdom | MRSA | Human | 2 | 3 | CSRC00000000 | NCBI |
| BSAR486 | United Kingdom | MRSA | Human | 3 | 2 | CSQK00000000 | NCBI |
| BSAR734 | United Kingdom | MRSA | Human | 0 | 4 | CSQJ00000000 | NCBI |
| BSAR739 | United Kingdom | MRSA | Human | 2 | 2 | CSQH00000000 | NCBI |
| BSAR176_2 | United Kingdom | MRSA | canine | 0 | 3 | CSPO00000000 | NCBI |
| BSAR193_2 | United Kingdom | MRSA | canine | 2 | 2 | CSPH00000000 | NCBI |
| BSAR865 | United Kingdom | MRSA | Other | 2 | 5 | CSOQ00000000 | NCBI |
| BSAR205 | United Kingdom | MRSA | Other | 1 | 2 | CSOM00000000 | NCBI |
| BSAR77 | United Kingdom | MRSA | Human | 2 | 2 | CSNI00000000 | NCBI |
| BSAR729 | United Kingdom | MRSA | Human | 1 | 5 | CSMU00000000 | NCBI |
| USFL253 | United State | MSSA | Human | 1 | 4 | CSJT00000000 | NCBI |
| USFL190 | United State | MSSA | Human | 1 | 1 | CSHX00000000 | NCBI |
| USFL246 | United State | MSSA | Human | 2 | 3 | CSHA00000000 | NCBI |
| BSAR111_2 | Denmark | MSSA | sheep | 2 | 1 | CSBB00000000 | NCBI |
| BSAR57 | Denmark | MSSA | Human | 3 | 0 | CSAX00000000 | NCBI |
| RIVM3897 | Netherlands | MRSA | Human | 3 | 1 | CP013621 | NCBI |
| RIVM1607 | Netherlands | MRSA | Human | 0 | 2 | CP013619 | NCBI |
| UTSW_MRSA_55 | United State | MRSA | Human | 2 | 3 | CP013231 | NCBI |
| JS395 | Switzerland | MRSA | Human | 0 | 5 | CP012756 | NCBI |
| Tager_104 | United State | MSSA | Human | 1 | 1 | CP012409 | NCBI |
| USA300_2014.C02 | United State | MRSA | Human | 2 | 2 | CP012120 | NCBI |
| USA300_2014.C01 | United State | MRSA | Human | 3 | 3 | CP012119 | NCBI |
| FORC_012 | Korea | MRSA | Human | 1 | 0 | CP010998 | NCBI |
| 93b_S9 | South Africa | MRSA | Human | 2 | 0 | CP010952 | NCBI |
| 79_S10 | South Africa | MRSA | Human | 1 | 1 | CP010944 | NCBI |
| 71A_S11 | South Africa | MRSA | Human | 0 | 4 | CP010940 | NCBI |
| DAR4145 | India | MRSA | Human | 2 | 3 | CP010526 | NCBI |
| FORC_001 | Korea | MSSA | Other | 1 | 0 | CP009554 | NCBI |
| ATCC_25923 | United State | MSSA | Human | 2 | 2 | CP009361 | NCBI |
| 6850 | Germany | MSSA | Human | 0 | 2 | CP006706 | NCBI |
| CA_347 | United State | MRSA | Human | 2 | 4 | CP006044 | NCBI |
| CN1 | Korea | MRSA | Unknown | 0 | 1 | CP003979 | NCBI |
| 71193 | United State | MSSA | Human | 1 | 1 | CP003045 | NCBI |
| VC40 | Germany | MSSA | Other | 0 | 2 | CP003033 | NCBI |
| TCH60 | United State | MRSA | Human | 1 | 2 | CP002110 | NCBI |
| 04_02981 | Germany | MRSA | Human | 2 | 3 | CP001844 | NCBI |
| ED98 | Ireland | MSSA | Chicken | 2 | 1 | CP001781 | NCBI |
| JH1 | Unknown | MRSA | Unknown | 3 | 3 | CP000736 | NCBI |
| USA300_TCH1516 | United State | MRSA | Human | 2 | 3 | CP000730 | NCBI |
| JH9 | United State | MRSA | Unknown | 3 | 3 | CP000703 | NCBI |
| USA300_FPR3757 | United State | MRSA | Human | 2 | 3 | CP000255 | NCBI |
| COL | United Kingdom | MRSA | Other | 1 | 2 | CP000046 | NCBI |
| USFL022 | United State | MRSA | Human | 2 | 4 | CIAQ00000000 | NCBI |
| BSAR58 | Denmark | MSSA | Human | 3 | 0 | CHEJ00000000 | NCBI |
| BSAR202 | United Kingdom | MRSA | canine | 2 | 2 | CGHI00000000 | NCBI |
| S2396 | France | MSSA | Human | 2 | 9 | CAWA000000000 | NCBI |
| S2397 | France | MSSA | Human | 2 | 5 | CAVV000000000 | NCBI |
| MSSA476 | Unknown | MSSA | Human | 1 | 0 | BX571857 | NCBI |
| MRSA252 | United Kingdom | MRSA | Human | 3 | 2 | BX571856 | NCBI |
| OC3 | Russia | MRSA | Human | 2 | 4 | BBKC00000000 | NCBI |
| Mu50 | Japan | MRSA | Unknown | 1 | 5 | BA000017 | NCBI |
| HST_105 | Lebanon | MRSA | Human | 1 | 1 | AZTH00000000 | NCBI |
| MUM475 | India | MRSA | Human | 2 | 1 | AZSG00000000 | NCBI |
| MUM270 | India | MRSA | Human | 2 | 2 | AZSF00000000 | NCBI |
| ST772_MRSA_V | Australia | MRSA | Human | 1 | 1 | AZBT00000000 | NCBI |
| 10S | Pakistan | MRSA | Human | 1 | 3 | AYXU00000000 | NCBI |
| HST_077 | Lebanon | MRSA | Human | 0 | 7 | AVPR00000000 | NCBI |
| SA_ST125_MupR | Spain | MRSA | Human | 0 | 4 | ASTH00000000 | NCBI |
| 06BA18369 | Canada | MSSA | Human | 1 | 7 | ARXY00000000 | NCBI |
| KLT6 | Switzerland | MSSA | Other | 0 | 3 | APFH00000000 | NCBI |
| Newman | Unknown | MSSA | Unknown | 4 | 2 | AP009351 | NCBI |
| Mu3 | Japan | MRSA | Human | 1 | 5 | AP009324 | NCBI |
| VH221 | India | MSSA | Human | 1 | 4 | AOFX00000000 | NCBI |
| LVP5 | India | MSSA | Human | 1 | 5 | AOFW00000000 | NCBI |
| LVP2 | India | MRSA | Human | 3 | 4 | AOFV00000000 | NCBI |
| 3957 | India | MRSA | Human | 2 | 3 | AOFU00000000 | NCBI |
| KT_Y21 | Malaysia | MRSA | Human | 1 | 1 | AOCQ00000000 | NCBI |
| KT_314250 | Malaysia | MSSA | Human | 2 | 4 | AOCP00000000 | NCBI |
| MRSA_PR1 | Malaysia | MRSA | Human | 0 | 6 | ANPO00000000 | NCBI |
| S0385 | Australia | MRSA | Human | 2 | 2 | AM990992 | NCBI |
| 333 | India | MRSA | Human | 1 | 1 | ALWF00000000 | NCBI |
| 118 | India | MRSA | Human | 1 | 1 | AJGE00000000 | NCBI |
| M0513 | United State | MRSA | Human | 3 | 4 | AJDA00000000 | NCBI |
| M0396 | United State | MSSA | Human | 1 | 2 | AJCV00000000 | NCBI |
| RF122 | Ireland | MSSA | Bovine | 0 | 3 | AJ938182 | NCBI |
| M1311 | United State | MRSA | Human | 0 | 5 | AIZE00000000 | NCBI |
| M1216 | United State | MRSA | Human | 0 | 4 | AIYW00000000 | NCBI |
| M0239 | United State | MSSA | Human | 1 | 1 | AIWE00000000 | NCBI |
| HI111 | United State | MRSA | Human | 2 | 3 | AIVO00000000 | NCBI |
| HI022 | United State | MSSA | Human | 2 | 3 | AIVL00000000 | NCBI |
| HIF003_B2N_C | United State | MSSA | Human | 1 | 2 | AIVK00000000 | NCBI |
| HI010 | United State | MRSA | Human | 2 | 3 | AIVH00000000 | NCBI |
| CIG1835 | United State | MRSA | Human | 1 | 1 | AIEQ00000000 | NCBI |
| CIG1242 | United State | MRSA | Human | 4 | 3 | AIEO00000000 | NCBI |
| MRGR3 | Switzerland | MRSA | Human | 1 | 12 | AHZL00000000 | NCBI |
| 122051 | Switzerland | MRSA | Human | 1 | 2 | AHZJ00000000 | NCBI |
| 91751 | Switzerland | MRSA | Human | 2 | 7 | AHZH00000000 | NCBI |
| CIG1524 | United State | MRSA | Human | 0 | 2 | AHVI00000000 | NCBI |
| O46 | France | MSSA | Sheep | 2 | 4 | AEUR00000000 | NCBI |
| O11 | France | MSSA | Sheep | 1 | 4 | AEUQ00000000 | NCBI |
| MR1 | Poland | MRSA | Human | 1 | 3 | ACZQ00000000 | NCBI |
| USA300_TCH959 | United State | MSSA | Human | 2 | 2 | AASB00000000 | NCBI |
| RKI4 | Germeny | MSSA | Human | 0 | 2 | NZ_CP011528 | NCBI |
| Sa13_005 | Australian | MSSA | Sheep | 1 | 3 | MAQM00000000 | NCBI |
| FDA209P | Unknown | MSSA | Unknown | 1 | 3 | AP014942 | NCBI |
| A69 | China | MRSA | Swine | 1 | 2 | JJOP01000000 | NCBI |
| A71 | China | MRSA | Swine | 1 | 3 | JJOO01000000 | NCBI |
| N315 | Japan | MRSA | Human | 1 | 4 | BA000018 | NCBI |
| MW2 | United State | MRSA | Human | 1 | 1 | BA000033 | NCBI |
| M3 | China | MRSA | Swine | 0 | 2 | MWRY00000000 | NCBI |
| M6 | China | MRSA | Swine | 0 | 2 | NCQK00000000 | NCBI |
| M48 | China | MRSA | Swine | 4 | 4 | NZ_CP030138.1 | NCBI |
| M51 | China | MRSA | Swine | 2 | 2 | NZ_CP030137.1 | NCBI |
| 57 | China | MRSA | Swine | 1 | 2 | - | Processing in NCBI |
| 92 | China | MRSA | Swine | 2 | 4 | - | Processing in NCBI |
| 107 | China | MRSA | Swine | 1 | 3 | - | Processing in NCBI |
| 125 | China | MRSA | Swine | 1 | 3 | - | Processing in NCBI |
| SAHPchr | China | MRSA | Swine | 1 | 5 | - | Processing in NCBI |
| MS4 | China | MRSA | Human | 1 | 3 | CP009828.1 | NCBI |
| CUHK_HK188 | China | MRSA | Human | 1 | 4 | JFFV00000000 | NCBI |
| LCT_SAO | China | MRSA | Human | 0 | 6 | JANM00000000 | NCBI |
| M996 | China | MRSA | Human | 1 | 5 | CTXU00000000 | NCBI |
| XN108 | China | MRSA | Human | 3 | 5 | CP007447 | NCBI |
| Z172 | China | MRSA | Human | 2 | 5 | CP006838 | NCBI |
| SA268 | China | MRSA | Human | 1 | 3 | CP006630 | NCBI |
| SA40 | China | MRSA | Human | 0 | 3 | CP003604 | NCBI |
| SA957 | China | MRSA | Human | 1 | 3 | CP003603 | NCBI |
| M013 | China | MRSA | Human | 1 | 3 | CP003166 | NCBI |
| T0131 | China | MRSA | Human | 1 | 4 | CP002643 | NCBI |
| CUHK_HK2007 | China | MRSA | Human | 1 | 7 | AZMZ00000000 | NCBI |
| CUHK_BJ2002 | China | MRSA | Human | 1 | 1 | AZMY00000000 | NCBI |
| CUHK_HK1997 | China | MRSA | Human | 1 | 10 | AZJQ00000000 | NCBI |
| CN79 | China | MRSA | Human | 2 | 3 | ANCJ00000000 | NCBI |
| ZJ5499 | China | MRSA | Human | 2 | 4 | CP011685 | NCBI |
| XQ | China | MSSA | Human | 3 | 0 | CP013137.1 | NCBI |
| 4126_2 | China | MRSA | Human | 0 | 4 | MBTE00000000.1 | NCBI |
| 4126_1 | China | MRSA | Human | 4 | 0 | MBTD00000000.1 | NCBI |
| 3503 | China | MRSA | Human | 0 | 4 | MBTB00000000.1 | NCBI |
| 3503VR10 | China | MSSA | Human | 0 | 2 | MBTC00000000.1 | NCBI |
| SA40TW | China | MRSA | Human | 0 | 1 | CP013182.1 | NCBI |
| GD1677 | China | MSSA | Human | 1 | 1 | CP019595.1 | NCBI |
| GD1539 | China | MSSA | Human | 3 | 1 | CP019594.1 | NCBI |
| GD705 | China | MSSA | Human | 1 | 1 | CP019593.1 | NCBI |
| GD5 | China | MRSA | Human | 1 | 1 | CP019592.1 | NCBI |
| HZW450 | China | MRSA | Human | 1 | 3 | NZ_CP020741.1 | NCBI |
| FY22 | China | MRSA | Human | 0 | 1 | NZ_NXFU00000000.1 | NCBI |
| FY20 | China | MRSA | Human | 0 | 1 | NZ_NTMC00000000.1 | NCBI |
| A187 | China | MSSA | Swine | 0 | 1 | NZ_JJON00000000.1 | NCBI |
| LCT_SAM | China | MRSA | Human | 0 | 2 | NZ_JANO00000000.1 | NCBI |
| LCT_SAS | China | MSSA | Human | 0 | 1 | JANP00000000.1 | NCBI |
| LCT_SAG | China | MRSA | Human | 0 | 1 | JANN00000000.1 | NCBI |
| SR434 | China | MRSA | Human | 1 | 3 | CP019563.1 | NCBI |
| SR389 | China | MRSA | Human | 0 | 0 | NZ_PDFA00000000.1 | NCBI |
| SR411 | China | MRSA | Human | 0 | 1 | NZ_PDFB00000000.1 | NCBI |
| MS4_LZD100 | China | MRSA | Human | 1 | 1 | NZ_JXMJ00000000.1 | NCBI |
| TSAR05 | China | MRSA | Human | 2 | 5 | NZ_NADE00000000.1 | NCBI |
| TSAR02 | China | MRSA | Human | 1 | 2 | NZ_NADB00000000.1 | NCBI |
| TSAR07 | China | MRSA | Human | 0 | 2 | NZ_NADH00000000.1 | NCBI |
| TSAR08 | China | MRSA | Human | 1 | 1 | NZ_NADG00000000.1 | NCBI |
| TSAR06 | China | MRSA | Human | 0 | 1 | NZ_NADF00000000.1 | NCBI |
| QR502 | China | MRSA | Human | 1 | 3 | NZ_NADI00000000.1 | NCBI |
| TSAR04 | China | MRSA | Human | 1 | 2 | NZ_NADD00000000.1 | NCBI |
| TSAR01 | China | MRSA | Human | 1 | 3 | NZ_NADA00000000.1 | NCBI |
| TSAR03 | China | MRSA | Human | 0 | 1 | NZ_NADC00000000.1 | NCBI |
| **Outgroup** |  |  |  |  |  |  |  |
| *Staphylococcus simiae* CCM7213 | |  |  | 1 | 2 | NZ_AEUN00000000 | NCBI |

**Table S2 Metadata for** **579 intact phages predicted in the genomes of *S. aureus* and outgroup phage Erwinia_phage_phiEa2809 (NC_027340.1).**

| **Intact Phages** | | **Genome length (bp)** | | **GC content (%)** | | **ORFs** | | **tRNA** | | **Geographic region** | | **Isolation source** | |
| --- | --- | --- | --- | --- | --- | --- | --- | --- | --- | --- | --- | --- | --- |
| 03_01478_p1 | | 51569 | | 33.1 | | 70 | | 0 | | Germany | | Human | |
| 03_01478_p3 | | 56195 | | 32.3 | | 80 | | 0 | | Germany | | Human | |
| 04_00608_p1 | | 37271 | | 33.4 | | 51 | | 0 | | Germany | | Human | |
| 04_00608_p4 | | 44937 | | 32.1 | | 71 | | 0 | | Germany | | Human | |
| 04_02314_1_p4 | | 91983 | | 32.5 | | 137 | | 0 | | United Kingdom | | Human | |
| 04_02981_p2 | | 69455 | | 33.7 | | 99 | | 0 | | Germany | | Human | |
| 04_02981_p5 | | 47584 | | 32.6 | | 68 | | 0 | | Germany | | Human | |
| 04_03111_p1 | | 69081 | | 32.9 | | 87 | | 0 | | Germany | | Human | |
| 04_03111_p2 | | 68987 | | 32.4 | | 91 | | 0 | | Germany | | Human | |
| 5R_157_2_p2 | | 31202 | | 35.2 | | 38 | | 0 | | Mexico | | Human | |
| 06_01900_p4 | | 48105 | | 33.1 | | 53 | | 0 | | Germany | | Human | |
| 06BA18369_p8 | | 21504 | | 34.5 | | 36 | | 0 | | Canada | | Human | |
| 07_00059_p1 | | 55408 | | 32.6 | | 79 | | 0 | | Germany | | Human | |
| 07_00059_p3 | | 50881 | | 32.9 | | 67 | | 0 | | Germany | | Human | |
| 07_00063_p1 | | 46166 | | 33.3 | | 68 | | 0 | | Germany | | Human | |
| 07_00655_p1 | | 68975 | | 32.4 | | 91 | | 0 | | Germany | | Human | |
| 07_02088_p1 | | 69020 | | 32.4 | | 91 | | 0 | | Germany | | Human | |
| 07_02088_p2 | | 65815 | | 32.8 | | 84 | | 0 | | Germany | | Human | |
| 07_02789_p3 | | 37488 | | 33.3 | | 46 | | 0 | | Germany | | Human | |
| 07_02789_p4 | | 55318 | | 32.7 | | 76 | | 0 | | Germany | | Human | |
| 07_02997_p4 | | 50036 | | 33.7 | | 53 | | 0 | | Australia | | Human | |
| 07_03345_p2 | | 27764 | | 33.4 | | 33 | | 0 | | Czech Republic | | Human | |
| 07_03346_p1 | | 68909 | | 32.4 | | 91 | | 0 | | Czech Republic | | Human | |
| 07_03346_p3 | | 38463 | | 32.1 | | 46 | | 0 | | Czech Republic | | Human | |
| 08_01304_p2 | | 55375 | | 32.6 | | 79 | | 0 | | Germany | | Human | |
| 08_01667_p1 | | 55375 | | 32.6 | | 79 | | 0 | | Germany | | Human | |
| 08_01668_p2 | | 68987 | | 32.4 | | 91 | | 0 | | Germany | | Human | |
| 08_01669_p1 | | 46637 | | 33.3 | | 68 | | 0 | | Denmark | | Human | |
| 08_01671_p1 | | 67288 | | 32.6 | | 86 | | 0 | | Germany | | Human | |
| 08_01671_p3 | | 68987 | | 32.4 | | 91 | | 0 | | Germany | | Human | |
| 08_02119_p2 | | 67048 | | 33.1 | | 82 | | 0 | | Germany | | Human | |
| 08_02119_p3 | | 47131 | | 34.7 | | 70 | | 0 | | Germany | | Human | |
| 08BA02176_p1 | | 45431 | | 33.5 | | 64 | | 0 | | Canada | | Human | |
| 09_01244_p1 | | 68987 | | 32.4 | | 91 | | 0 | | Singapore | | Human | |
| 09_01244_p4 | | 60210 | | 33.0 | | 75 | | 0 | | Singapore | | Human | |
| 11P4_p1 | | 72437 | | 32.9 | | 91 | | 0 | | Netherlands | | Swine | |
| 25_2889_p1 | | 27426 | | 36.2 | | 31 | | 0 | | Netherlands | | Human | |
| 25_2889_p3 | | 36300 | | 33.6 | | 39 | | 0 | | Netherlands | | Human | |
| 36P1_p1 | | 71337 | | 33.0 | | 102 | | 0 | | Netherlands | | Swine | |
| 42S_p1 | | 48384 | | 33.1 | | 72 | | 0 | | Pakistan | | Human | |
| 043H_p4 | | 36306 | | 34.2 | | 45 | | 0 | | Tanzania | | Unknown | |
| 43P8_p3 | | 50261 | | 33.7 | | 70 | | 0 | | Netherlands | | Swine | |
| 51S_p3 | | 42264 | | 33.5 | | 69 | | 0 | | Pakistan | | Human | |
| 57_p2 | | 42903 | | 33.1 | | 63 | | 0 | | China | | Swine | |
| 065H_p1 | | 67277 | | 33.9 | | 97 | | 0 | | Tanzania | | Unknown | |
| 69_SAUR_p3 | | 57663 | | 32.4 | | 91 | | 0 | | United State | | Human | |
| 79_S10_p2 | | 56920 | | 32.9 | | 84 | | 0 | | South Africa | | Human | |
| 04_03103_p2 | | 56228 | | 32.3 | | 80 | | 0 | | Germany | | Human | |
| 04_03103_p3 | | 39331 | | 32.0 | | 46 | | 0 | | Germany | | Human | |
| 82_SAUR_p1 | | 56640 | | 33.0 | | 81 | | 0 | | United State | | Human | |
| 92_p5 | | 52923 | | 33.1 | | 71 | | 2 | | China | | Swine | |
| 92_p6 | | 56571 | | 32.2 | | 84 | | 0 | | China | | Swine | |
| 93b_S9_p2 | | 41545 | | 32.3 | | 52 | | 0 | | South Africa | | Human | |
| 93b_S9_p4 | | 46676 | | 33.5 | | 74 | | 0 | | South Africa | | Human | |
| 107_p3 | | 56801 | | 32.3 | | 80 | | 0 | | China | | Swine | |
| 118_p2 | | 59366 | | 32.8 | | 89 | | 0 | | India | | Human | |
| 120_p1 | | 40060 | | 34.7 | | 62 | | 0 | | Belgium | | Bovine | |
| 125_p3 | | 59974 | | 32.2 | | 84 | | 0 | | China | | Swine | |
| 147_SAUR_p1 | | 60382 | | 33.4 | | 89 | | 0 | | United State | | Human | |
| 147_SAUR_p2 | | 46488 | | 33.1 | | 69 | | 0 | | United State | | Human | |
| 169_SAUR_p1 | | 55825 | | 32.3 | | 80 | | 0 | | United State | | Human | |
| 170_p2 | | 19565 | | 36.1 | | 23 | | 0 | | Brazil | | Bovine | |
| 229_SAUR_p3 | | 29545 | | 35.3 | | 34 | | 0 | | United State | | Human | |
| 299_p1 | | 42526 | | 34.9 | | 63 | | 0 | | Belgium | | Bovine | |
| 302_p1 | | 27858 | | 36.3 | | 31 | | 0 | | Brazil | | Bovine | |
| 333_p2 | | 48384 | | 33.1 | | 72 | | 0 | | India | | Human | |
| 337_p1 | | 59338 | | 32.3 | | 74 | | 0 | | Malaysia | | Human | |
| 394_SAUR_p1 | | 30797 | | 35.1 | | 37 | | 0 | | United State | | Human | |
| 511_SAUR_p1 | | 63326 | | 33.1 | | 81 | | 0 | | United State | | Human | |
| 561_SAUR_p1 | | 44965 | | 33.4 | | 64 | | 0 | | United State | | Human | |
| 04Hi_p1 | | 52000 | | 33.2 | | 70 | | 0 | | Tanzania | | Unknown | |
| 04Hi_p2 | | 44602 | | 33.3 | | 65 | | 0 | | Tanzania | | Unknown | |
| 573_SAUR_p2 | | 44965 | | 33.4 | | 64 | | 0 | | United State | | Human | |
| 685_p1 | | 43311 | | 34.1 | | 73 | | 0 | | Italy | | Bovine | |
| 922_SAUR_p2 | | 28814 | | 35.4 | | 32 | | 0 | | United State | | Human | |
| 1089_SAUR_p2 | | 23391 | | 34.8 | | 35 | | 0 | | United State | | Human | |
| 1158_SAUR_p1 | | 42185 | | 35.1 | | 65 | | 0 | | United State | | Human | |
| 1159_SAUR_p4 | | 36209 | | 33.2 | | 39 | | 0 | | United State | | Human | |
| 1322_2_SAUR_p2 | | 43726 | | 33.1 | | 63 | | 0 | | United State | | Human | |
| 1943STDY5573748_p2 | | 51187 | | 32.6 | | 70 | | 0 | | United Kingdom | | Unknown | |
| 1943STDY5573749_p2 | | 63321 | | 33.0 | | 88 | | 0 | | United Kingdom | | Unknown | |
| 1943STDY5698363_p4 | | 39210 | | 35.3 | | 55 | | 0 | | United Kingdom | | Other | |
| 2009_60_561_1_p2 | | 73204 | | 33.1 | | 96 | | 0 | | Germany | | Chicken | |
| 2010_60_6511_5_p4 | | 29592 | | 35.6 | | 45 | | 0 | | Netherlands | | Bovine | |
| 2010_60_6511_10_p1 | | 46743 | | 34.7 | | 73 | | 0 | | Netherlands | | Bovine | |
| 2010_60_6511_10_p4 | | 31883 | | 33.9 | | 39 | | 0 | | Netherlands | | Bovine | |
| 2010_60_6511_39_p1 | | 45077 | | 33.9 | | 64 | | 0 | | Germany | | Chicken | |
| 2010_60_6511_39_p4 | | 26210 | | 35.8 | | 35 | | 0 | | Germany | | Chicken | |
| 2011_60_1490_31_p2 | | 72998 | | 33.1 | | 94 | | 0 | | Germany | | Chicken | |
| 2011_60_2078_5_p2 | | 30946 | | 33.6 | | 38 | | 0 | | Netherlands | | Bovine | |
| 2011_60_2078_5_p5 | | 24450 | | 36.0 | | 32 | | 0 | | Netherlands | | Bovine | |
| 2011_60_2275_1_p1 | | 27426 | | 36.2 | | 31 | | 0 | | Germany | | Chicken | |
| 2011_60_2275_1_p4 | | 36300 | | 33.6 | | 39 | | 0 | | Germany | | Chicken | |
| 3957_p4 | | 40395 | | 34.3 | | 57 | | 0 | | India | | Human | |
| 3957_p5 | | 30165 | | 33.9 | | 43 | | 0 | | India | | Human | |
| 4126_1_p1 | | 18830 | | 27.7 | | 21 | | 0 | | China: Guangzhou | | Human | |
| 4126_1_p3 | | 19972 | | 30.9 | | 26 | | 0 | | China: Guangzhou | | Human | |
| 4126_1_p4 | | 10039 | | 32.0 | | 9 | | 0 | | China: Guangzhou | | Human | |
| 4126_1_p5 | | 34429 | | 34.2 | | 49 | | 0 | | China: Guangzhou | | Human | |
| 08134_6_p1 | | 70409 | | 33.0 | | 93 | | 0 | | Belgium | | Other | |
| 08142_8_p3 | | 24630 | | 36.0 | | 33 | | 0 | | Netherlands | | Other | |
| 08143_5_p3 | | 24675 | | 36.0 | | 33 | | 0 | | Netherlands | | Other | |
| 18412_p1 | | 6104 | | 28.6 | | 7 | | 0 | | Switzerland | | Unknown | |
| 18412_p2 | | 18826 | | 27.7 | | 21 | | 0 | | Switzerland | | Unknown | |
| 18754_2_p3 | | 49091 | | 34.9 | | 72 | | 0 | | Italy | | Swine | |
| 71193_p2 | | 45142 | | 33.0 | | 65 | | 0 | | United State | | Human | |
| 91751_p1 | | 36315 | | 34.2 | | 45 | | 0 | | Switzerland | | Human | |
| 91751_p8 | | 86251 | | 31.5 | | 117 | | 0 | | Switzerland | | Human | |
| 122051_p2 | | 57827 | | 33.2 | | 74 | | 0 | | Switzerland | | Human | |
| 1110700562_p2 | | 36674 | | 33.4 | | 55 | | 0 | | Netherlands | | Human | |
| 1110700610_p1 | | 70981 | | 32.9 | | 100 | | 0 | | Netherlands | | Human | |
| A5_p3 | | 38677 | | 34.8 | | 45 | | 0 | | Australia | | Human | |
| A69_p1 | | 55817 | | 33.2 | | 86 | | 0 | | China | | Swine | |
| A71_p4 | | 24235 | | 33.5 | | 31 | | 0 | | China | | Swine | |
| aless_p1 | | 60179 | | 32.3 | | 87 | | 0 | | Brazil | | Human | |
| APS210_p5 | | 26705 | | 36.6 | | 32 | | 0 | | Australia | | Human | |
| ARI29_p6 | | 40529 | | 34.9 | | 44 | | 0 | | United Kingdom | | Human | |
| ARI31_p3 | | 65299 | | 32.5 | | 91 | | 0 | | United Kingdom | | Human | |
| ARI31_p4 | | 30533 | | 33.4 | | 34 | | 0 | | United Kingdom | | Human | |
| ATCC_25923_p1 | | 33641 | | 32.5 | | 42 | | 0 | | United State | | Human | |
| ATCC_25923_p2 | | 54183 | | 33.0 | | 82 | | 0 | | United State | | Human | |
| ATCC_29213_p1 | | 27509 | | 34.7 | | 38 | | 0 | | Unknown | | Unknown | |
| AUS0325_p1 | | 68615 | | 33.4 | | 98 | | 0 | | Australia | | Human | |
| AUS0325_p3 | | 50735 | | 32.7 | | 75 | | 0 | | Australia | | Human | |
| BA01611_p2 | | 53915 | | 34.1 | | 79 | | 0 | | China | | Bovine | |
| BA01611_p6 | | 53557 | | 33.6 | | 80 | | 0 | | China | | Bovine | |
| BB155_p1 | | 71487 | | 32.9 | | 91 | | 0 | | Mali | | Human | |
| BB155_p2 | | 57004 | | 34.0 | | 84 | | 0 | | Mali | | Human | |
| BSAR57_p1 | | 37079 | | 32.5 | | 49 | | 0 | | Denmark | | Human | |
| BSAR57_p2 | | 66448 | | 33.5 | | 91 | | 0 | | Denmark | | Human | |
| BSAR57_p3 | | 29491 | | 34.6 | | 40 | | 0 | | Denmark | | Human | |
| BSAR58_p1 | | 37079 | | 32.5 | | 49 | | 0 | | Denmark | | Human | |
| BSAR58_p2 | | 63412 | | 33.4 | | 89 | | 0 | | Denmark | | Human | |
| BSAR58_p3 | | 47593 | | 33.6 | | 53 | | 0 | | Denmark | | Human | |
| BSAR77_p1 | | 51569 | | 33.1 | | 69 | | 0 | | United Kingdom | | Human | |
| BSAR77_p2 | | 68987 | | 32.4 | | 91 | | 0 | | United Kingdom | | Human | |
| BSAR111_2_p1 | | 42187 | | 32.6 | | 57 | | 0 | | Denmark | | sheep | |
| BSAR111_2_p2 | | 47580 | | 33.8 | | 65 | | 0 | | Denmark | | sheep | |
| BSAR193_2_p1 | | 63048 | | 32.8 | | 85 | | 0 | | United Kingdom | | canine | |
| BSAR193_2_p3 | | 52520 | | 32.9 | | 73 | | 0 | | United Kingdom | | canine | |
| BSAR202_p1 | | 75885 | | 32.9 | | 93 | | 0 | | United Kingdom | | canine | |
| BSAR202_p3 | | 62499 | | 32.5 | | 84 | | 0 | | United Kingdom | | canine | |
| BSAR203_p1 | | 71174 | | 33.0 | | 86 | | 0 | | United Kingdom | | canine | |
| BSAR203_p2 | | 58922 | | 32.8 | | 79 | | 0 | | United Kingdom | | canine | |
| BSAR205_p1 | | 72946 | | 32.8 | | 93 | | 0 | | United Kingdom | | Other | |
| BSAR208_p3 | | 36641 | | 33.1 | | 42 | | 0 | | United Kingdom | | Human | |
| BSAR208_p4 | | 29358 | | 35.6 | | 31 | | 0 | | United Kingdom | | Human | |
| BSAR486_p1 | | 71171 | | 33.0 | | 87 | | 0 | | United Kingdom | | Human | |
| BSAR486_p3 | | 36954 | | 33.6 | | 57 | | 0 | | United Kingdom | | Human | |
| BSAR486_p5 | | 10621 | | 30.5 | | 16 | | 0 | | United Kingdom | | Human | |
| BSAR729_p3 | | 82307 | | 32.0 | | 97 | | 0 | | United Kingdom | | Human | |
| BSAR739_p1 | | 51570 | | 33.1 | | 69 | | 0 | | United Kingdom | | Human | |
| BSAR739_p2 | | 62386 | | 32.1 | | 86 | | 0 | | United Kingdom | | Human | |
| BSAR748_p1 | | 47055 | | 33.5 | | 63 | | 0 | | United Kingdom | | Human | |
| BSAR858_p1 | | 48871 | | 33.0 | | 66 | | 0 | | United Kingdom | | canine | |
| BSAR865_p1 | | 20172 | | 31.2 | | 28 | | 0 | | United Kingdom | | Other | |
| BSAR865_p3 | | 35893 | | 32.6 | | 40 | | 0 | | United Kingdom | | Other | |
| BU_G0201_t8_p1 | | 54914 | | 33.3 | | 72 | | 0 | | Ghana | | Human | |
| BU_G0201_t8_p3 | | 51144 | | 34.9 | | 74 | | 0 | | Ghana | | Human | |
| 07_00309_p1 | | 59221 | | 32.9 | | 80 | | 0 | | Germany | | Human | |
| 07_00309_p3 | | 55374 | | 32.6 | | 79 | | 0 | | Germany | | Human | |
| BU_G0301_t8_p1 | | 43725 | | 33.3 | | 61 | | 0 | | Ghana | | Human | |
| BU_G0301_t8_p2 | | 34114 | | 35.9 | | 53 | | 7 | | Ghana | | Human | |
| BU_G1074_t4_p1 | | 42437 | | 35.5 | | 64 | | 0 | | Ghana | | Human | |
| BU_G1074_t4_p2 | | 43106 | | 33.3 | | 63 | | 0 | | Ghana | | Human | |
| BU_N22_t6_p1 | | 46585 | | 33.6 | | 66 | | 0 | | Ghana | | Human | |
| BU_N22_t6_p2 | | 37595 | | 31.2 | | 55 | | 0 | | Ghana | | Human | |
| BU_N22_t6_p3 | | 40072 | | 33.5 | | 64 | | 0 | | Ghana | | Human | |
| BU_W12_t13_p3 | | 27750 | | 35.1 | | 34 | | 0 | | Ghana | | Human | |
| C9_p2 | | 44981 | | 33.3 | | 63 | | 0 | | Australia | | Human | |
| C1655_p1 | | 74200 | | 33.1 | | 97 | | 0 | | Spain | | Swine | |
| CA12_p3 | | 60406 | | 32.7 | | 80 | | 0 | | United State | | Human | |
| CA12_p4 | | 46375 | | 32.7 | | 68 | | 0 | | United State | | Human | |
| CA_347_p4 | | 61361 | | 32.9 | | 81 | | 0 | | United State | | Human | |
| CA_347_p7 | | 59606 | | 34.1 | | 88 | | 0 | | United State | | Human | |
| CC022_p3 | | 55367 | | 33.3 | | 81 | | 0 | | Brazil | | Human | |
| Chi_4_p2 | | 72998 | | 33.1 | | 94 | | 0 | | Germany | | Chicken | |
| Chi_8_p1 | | 28435 | | 35.4 | | 33 | | 0 | | Germany | | Chicken | |
| Chi_8_p4 | | 32030 | | 34.0 | | 35 | | 0 | | Germany | | Chicken | |
| Chi_10_p2 | | 30913 | | 34.1 | | 33 | | 0 | | Austria | | Chicken | |
| CIG1242_p1 | | 33118 | | 29.9 | | 39 | | 0 | | United State | | Human | |
| CIG1242_p3 | | 33850 | | 32.6 | | 42 | | 0 | | United State | | Human | |
| CIG1242_p4 | | 58086 | | 33.1 | | 82 | | 0 | | United State | | Human | |
| CIG1242_p6 | | 50347 | | 32.6 | | 73 | | 0 | | United State | | Human | |
| CIG1835_p2 | | 62658 | | 32.6 | | 82 | | 0 | | United State | | Human | |
| CN79_p2 | | 45328 | | 34.2 | | 69 | | 0 | | China | | Human | |
| CN79_p3 | | 70977 | | 31.8 | | 93 | | 0 | | China | | Human | |
| COL_p1 | | 43358 | | 33.9 | | 71 | | 0 | | United Kingdom | | Other | |
| CUHK_BJ2002_p1 | | 50352 | | 33.7 | | 76 | | 0 | | China | | Human | |
| CUHK_HK188_p3 | | 36785 | | 34.2 | | 45 | | 0 | | China | | Human | |
| CUHK_HK1997_p5 | | 39352 | | 34.5 | | 47 | | 0 | | China | | Human | |
| CUHK_HK2007_p1 | | 31366 | | 36.3 | | 37 | | 0 | | China | | Human | |
| D71_p5 | | 58265 | | 34.5 | | 61 | | 0 | | Germany | | Human | |
| DAR22_p1 | | 47219 | | 34.3 | | 71 | | 0 | | Belgium | | Human | |
| DAR22_p4 | | 47570 | | 32.6 | | 69 | | 0 | | Belgium | | Human | |
| DAR948_p2 | | 80821 | | 34.1 | | 102 | | 0 | | United State | | Human | |
| DAR948_p4 | | 45482 | | 33.2 | | 69 | | 0 | | United State | | Human | |
| DAR3179_p1 | | 73357 | | 34.0 | | 104 | | 0 | | Argentina | | Human | |
| DAR3236_p2 | | 20313 | | 31.1 | | 28 | | 0 | | Argentina | | Human | |
| DAR3236_p5 | | 53464 | | 34.0 | | 79 | | 0 | | Argentina | | Human | |
| DAR3236_p6 | | 45962 | | 32.9 | | 68 | | 0 | | Argentina | | Human | |
| DAR3534_p3 | | 47578 | | 32.6 | | 68 | | 0 | | United State | | Human | |
| DAR3581_p2 | | 62692 | | 33.0 | | 87 | | 0 | | United State | | Human | |
| DAR4145_p1 | | 62340 | | 33.4 | | 92 | | 0 | | India | | Human | |
| DAR4145_p5 | | 56919 | | 32.9 | | 82 | | 0 | | India | | Human | |
| DAR5843_p1 | | 30159 | | 33.9 | | 32 | | 0 | | Peru | | Human | |
| DAR5843_p2 | | 22442 | | 34.8 | | 24 | | 0 | | Peru | | Human | |
| DAR5854_p3 | | 27873 | | 35.0 | | 33 | | 0 | | Ecuador | | Human | |
| DAR5867_p2 | | 28102 | | 34.8 | | 33 | | 0 | | Argentina | | Human | |
| DAR5874_p3 | | 47297 | | 33.0 | | 69 | | 0 | | Colombia | | Human | |
| DAR5874_p4 | | 43168 | | 33.8 | | 64 | | 0 | | Colombia | | Human | |
| DAR5874_p5 | | 42060 | | 34.5 | | 69 | | 0 | | Colombia | | Human | |
| DAR5877_p2 | | 17381 | | 36.1 | | 21 | | 0 | | Peru | | Human | |
| DAR5889_p2 | | 73299 | | 33.1 | | 96 | | 0 | | Peru | | Human | |
| DAR5889_p3 | | 51430 | | 34.5 | | 71 | | 0 | | Peru | | Human | |
| DAR5889_p5 | | 34802 | | 32.4 | | 42 | | 0 | | Peru | | Human | |
| DAR5889_p6 | | 49349 | | 33.0 | | 70 | | 0 | | Peru | | Human | |
| DAR5889_p7 | | 53289 | | 34.0 | | 78 | | 0 | | Peru | | Human | |
| DAR5890_p4 | | 52377 | | 32.7 | | 72 | | 0 | | Venezuela | | Human | |
| DAR5890_p5 | | 35893 | | 32.1 | | 46 | | 0 | | Venezuela | | Human | |
| DAR5890_p6 | | 53464 | | 34.0 | | 79 | | 0 | | Venezuela | | Human | |
| DAR5890_p7 | | 45658 | | 33.6 | | 67 | | 0 | | Venezuela | | Human | |
| DAR5898_p5 | | 45962 | | 32.9 | | 68 | | 0 | | Venezuela | | Human | |
| DAR5898_p6 | | 36170 | | 31.9 | | 51 | | 0 | | Venezuela | | Human | |
| DAR5898_p7 | | 53464 | | 34.0 | | 79 | | 0 | | Venezuela | | Human | |
| DEU16_p6 | | 40005 | | 34.2 | | 60 | | 0 | | Turkey | | Human | |
| DICM09_01587_13HST_p2 | | 71197 | | 33.1 | | 96 | | 0 | | Spain | | Sheep | |
| DSM_799_p2 | | 58582 | | 32.6 | | 82 | | 0 | | United State | | Human | |
| ECT_R_2_p3 | | 47582 | | 32.6 | | 68 | | 0 | | Sweden | | Human | |
| ED98_p1 | | 89031 | | 33.0 | | 129 | | 0 | | Ireland | | Chicken | |
| ED98_p3 | | 46402 | | 33.1 | | 65 | | 0 | | Ireland | | Chicken | |
| ES26_p10 | | 36507 | | 33.2 | | 63 | | 0 | | Spain | | Human | |
| ES26_p4 | | 32034 | | 33.3 | | 34 | | 0 | | Spain | | Human | |
| ES26_p7 | | 14482 | | 36.1 | | 20 | | 0 | | Spain | | Human | |
| F26051_p2 | | 77184 | | 32.0 | | 108 | | 0 | | United State | | Human | |
| F48950_p2 | | 47642 | | 33.3 | | 61 | | 0 | | United State | | Human | |
| F48950_p4 | | 40927 | | 33.2 | | 60 | | 0 | | United State | | Human | |
| FDA209P_p4 | | 74287 | | 31.9 | | 98 | | 0 | | Unknown | | Unknown | |
| FKWK_p1 | | 60286 | | 32.9 | | 81 | | 0 | | United State | | Human | |
| FKWK_p5 | | 18472 | | 34.6 | | 26 | | 0 | | United State | | Human | |
| FKWK_p6 | | 16359 | | 36.0 | | 22 | | 0 | | United State | | Human | |
| FORC_001_p1 | | 72706 | | 33.4 | | 102 | | 0 | | Korea | | Other | |
| FORC_012_p1 | | 72706 | | 33.4 | | 102 | | 0 | | Korea | | Human | |
| FP_N239_p1 | | 46457 | | 33.6 | | 63 | | 0 | | Netherlands | | Bovine | |
| FRI137_p1 | | 31049 | | 33.9 | | 46 | | 0 | | United State | | Human | |
| FVRH6002_p2 | | 81916 | | 33.7 | | 106 | | 0 | | United State | | Human | |
| GD5_p2 | | 48335 | | 32.9 | | 69 | | 0 | | China | | Human | |
| GD705_p2 | | 85299 | | 32.7 | | 109 | | 0 | | China | | Human | |
| GD1539_p1 | | 61393 | | 34.3 | | 83 | | 0 | | China | | Human | |
| GD1539_p3 | | 58776 | | 33.0 | | 95 | | 0 | | China | | Human | |
| GD1539_p4 | | 49394 | | 32.9 | | 69 | | 0 | | China | | Human | |
| GD1677_p1 | | 44747 | | 33.9 | | 65 | | 0 | | China | | Human | |
| GKP136_8_p2 | | 25525 | | 33.5 | | 31 | | 0 | | United Kingdom | | Bovine | |
| GKP136_8_p3 | | 26925 | | 35.2 | | 32 | | 0 | | United Kingdom | | Bovine | |
| GKP136_11_p1 | | 12622 | | 31.3 | | 20 | | 0 | | United Kingdom | | Bovine | |
| GKP136_11_p3 | | 45821 | | 32.8 | | 53 | | 0 | | United Kingdom | | Bovine | |
| GKP136_11_p4 | | 24434 | | 36.4 | | 26 | | 0 | | United Kingdom | | Bovine | |
| GKP136_19_p5 | | 50916 | | 33.1 | | 67 | | 0 | | United Kingdom | | Bovine | |
| GKP136_21_p1 | | 55579 | | 33.8 | | 84 | | 0 | | United Kingdom | | Bovine | |
| GKP136_36_p1 | | 43041 | | 34.0 | | 65 | | 0 | | United Kingdom | | Bovine | |
| GKP136_64_p2 | | 47085 | | 32.9 | | 67 | | 0 | | United Kingdom | | Bovine | |
| GKP136_67_p1 | | 50451 | | 34.6 | | 70 | | 0 | | United Kingdom | | Bovine | |
| GKP136_68_p1 | | 69783 | | 33.5 | | 91 | | 0 | | United Kingdom | | Bovine | |
| GKP138_17_p4 | | 25452 | | 33.5 | | 30 | | 0 | | United Kingdom | | Bovine | |
| GKP138_17_p5 | | 23067 | | 35.1 | | 32 | | 0 | | United Kingdom | | Bovine | |
| GKP138_21_p3 | | 57461 | | 32.3 | | 81 | | 0 | | United Kingdom | | Bovine | |
| GKP138_41_p1 | | 59738 | | 33.3 | | 83 | | 0 | | United Kingdom | | Bovine | |
| GKP138_71_p2 | | 44285 | | 34.1 | | 56 | | 0 | | United Kingdom | | Bovine | |
| H_EMRSA_15_p3 | | 74629 | | 32.6 | | 95 | | 0 | | Belgium | | Human | |
| H_EMRSA_15_p6 | | 48357 | | 32.9 | | 73 | | 0 | | Belgium | | Human | |
| H24_p2 | | 13973 | | 31.4 | | 24 | | 0 | | Egypt | | Human | |
| H24_p5 | | 15932 | | 37.0 | | 20 | | 0 | | Egypt | | Human | |
| H24_p8 | | 50070 | | 32.9 | | 84 | | 0 | | Egypt | | Human | |
| H202_p3 | | 22198 | | 34.6 | | 44 | | 0 | | Thailand | | Human | |
| H211_p6 | | 41414 | | 34.4 | | 44 | | 0 | | Denmark | | Human | |
| H216_p7 | | 31267 | | 35.7 | | 37 | | 0 | | Denmark | | Human | |
| H216_p9 | | 21088 | | 34.1 | | 26 | | 0 | | Denmark | | Human | |
| H482_p8 | | 90138 | | 33.0 | | 137 | | 0 | | Romania | | Human | |
| H914_91_p3 | | 29830 | | 35.5 | | 32 | | 0 | | United Kingdom | | Human | |
| H914_91_p4 | | 74862 | | 32.2 | | 95 | | 0 | | United Kingdom | | Human | |
| H914_91_p5 | | 67094 | | 33.5 | | 85 | | 0 | | United Kingdom | | Human | |
| HI010_p4 | | 60405 | | 32.7 | | 81 | | 0 | | United State | | Human | |
| HI010_p5 | | 46375 | | 32.7 | | 68 | | 0 | | United State | | Human | |
| HI022_p4 | | 60405 | | 32.7 | | 81 | | 0 | | United State | | Human | |
| HI022_p5 | | 46375 | | 32.7 | | 68 | | 0 | | United State | | Human | |
| HI111_p4 | | 60353 | | 32.7 | | 81 | | 0 | | United State | | Human | |
| HI111_p5 | | 46212 | | 32.7 | | 68 | | 0 | | United State | | Human | |
| HIF003_B2N_C_p2 | | 74918 | | 31.7 | | 90 | | 0 | | United State | | Human | |
| HST_105_p2 | | 43875 | | 33.5 | | 62 | | 0 | | Lebanon | | Human | |
| HU8_p3 | | 28299 | | 36.4 | | 34 | | 0 | | Turkey | | Human | |
| HUV05_p4 | | 60407 | | 32.7 | | 80 | | 0 | | United State | | Human | |
| HUV05_p5 | | 46375 | | 32.7 | | 68 | | 0 | | United State | | Human | |
| HZW450_p3 | | 68800 | | 32.8 | | 102 | | 0 | | China | | Human | |
| IF6SW_P2_p2 | | 43300 | | 34.2 | | 70 | | 0 | | Space Station | | Other | |
| IF6SW_P2_p3 | | 50289 | | 32.1 | | 77 | | 0 | | Space Station | | Other | |
| IF6SW_P2_RA_p2 | | 43300 | | 34.2 | | 70 | | 0 | | Space Station | | Other | |
| IF7SW_P3_p1 | | 33739 | | 33.4 | | 39 | | 0 | | Space Station | | Other | |
| JH1_p2 | | 42888 | | 35.4 | | 65 | | 0 | | Unknown | | Unknown | |
| JH1_p3 | | 69498 | | 33.7 | | 99 | | 0 | | Unknown | | Unknown | |
| JH1_p4 | | 55836 | | 33.2 | | 71 | | 0 | | Unknown | | Unknown | |
| JH9_p2 | | 42888 | | 35.4 | | 65 | | 0 | | United State | | Unknown | |
| JH9_p3 | | 69498 | | 33.7 | | 99 | | 0 | | United State | | Unknown | |
| JH9_p4 | | 55836 | | 33.2 | | 71 | | 0 | | United State | | Unknown | |
| KINW6056_p3 | | 33850 | | 32.6 | | 42 | | 0 | | United State | | Human | |
| KINW6056_p7 | | 20304 | | 36.0 | | 25 | | 0 | | United State | | Human | |
| KT_314250_p5 | | 45393 | | 33.1 | | 50 | | 0 | | Malaysia | | Human | |
| KT_314250_p6 | | 28911 | | 34.3 | | 35 | | 0 | | Malaysia | | Human | |
| KT_Y21_p1 | | 56920 | | 32.9 | | 84 | | 0 | | Malaysia | | Human | |
| 07_03354_2_p1 | | 57969 | | 32.3 | | 78 | | 0 | | Denmark | | Human | |
| LGA251_p1 | | 65471 | | 34.6 | | 84 | | 0 | | United Kingdom | | Bovine | |
| LVP2_p3 | | 33453 | | 34.0 | | 51 | | 0 | | India | | Human | |
| LVP2_p5 | | 16620 | | 34.3 | | 21 | | 0 | | India | | Human | |
| LVP2_p7 | | 15706 | | 36.0 | | 20 | | 0 | | India | | Human | |
| LVP5_p3 | | 42451 | | 34.6 | | 53 | | 0 | | India | | Human | |
| M1_HF937103_p1 | | 67110 | | 31.3 | | 72 | | 0 | | Denmark | | Human | |
| M1_HF937103_p4 | | 60394 | | 32.8 | | 83 | | 0 | | Denmark | | Human | |
| M1_HF937103_p5 | | 52506 | | 32.8 | | 80 | | 0 | | Denmark | | Human | |
| M013_p3 | | 66879 | | 32.9 | | 99 | | 0 | | China | | Human | |
| M48_p1 | | 43097 | | 35.1 | | 68 | | 0 | | China | | Swine | |
| M48_p5 | | 63445 | | 33.9 | | 92 | | 0 | | China | | Swine | |
| M48_p6 | | 47943 | | 32.9 | | 71 | | 0 | | China | | Swine | |
| M48_p7 | | 134273 | | 30.9 | | 165 | | 0 | | China | | Swine | |
| M51_p1 | | 60085 | | 32.4 | | 81 | | 0 | | China | | Swine | |
| M51_p3 | | 47770 | | 35.1 | | 72 | | 0 | | China | | Swine | |
| M116_p12 | | 45964 | | 32.2 | | 85 | | 0 | | Viet Nam | | Human | |
| M116_p6 | | 49224 | | 33.5 | | 49 | | 0 | | Viet Nam | | Human | |
| M116_p8 | | 24905 | | 36.2 | | 28 | | 0 | | Viet Nam | | Human | |
| M0239_p2 | | 50856 | | 33.1 | | 80 | | 0 | | United State | | Human | |
| M278_p3 | | 40898 | | 33.5 | | 46 | | 0 | | Portugal | | Human | |
| M0396_p2 | | 62859 | | 32.9 | | 83 | | 0 | | United State | | Human | |
| M0406_p6 | | 32466 | | 34.3 | | 43 | | 0 | | United State | | Human | |
| M0406_p7 | | 31406 | | 33.5 | | 36 | | 0 | | United State | | Human | |
| M0426_p4 | | 21334 | | 35.8 | | 28 | | 0 | | United State | | Human | |
| M0513_p1 | | 61943 | | 32.9 | | 86 | | 0 | | United State | | Human | |
| M0513_p3 | | 33850 | | 32.6 | | 42 | | 0 | | United State | | Human | |
| M0513_p6 | | 47479 | | 32.9 | | 68 | | 0 | | United State | | Human | |
| M592_p12 | | 47847 | | 32.1 | | 83 | | 0 | | Syria | | Human | |
| M592_p8 | | 26790 | | 35.7 | | 31 | | 0 | | Syria | | Human | |
| M0684_p4 | | 35042 | | 35.0 | | 54 | | 0 | | United State | | Human | |
| M0715_p5 | | 45446 | | 34.0 | | 66 | | 0 | | United State | | Human | |
| M0745_p3 | | 58947 | | 33.6 | | 87 | | 0 | | United State | | Human | |
| M0821_p2 | | 57863 | | 33.7 | | 85 | | 0 | | United State | | Human | |
| M0896_p3 | | 57863 | | 33.7 | | 85 | | 0 | | United State | | Human | |
| M996_p6 | | 81012 | | 32.0 | | 135 | | 0 | | China | | Human | |
| M1169_p1 | | 59296 | | 33.0 | | 77 | | 0 | | United State | | Human | |
| M1242_p1 | | 56191 | | 32.9 | | 75 | | 0 | | United State | | Human | |
| M17033_p1 | | 41950 | | 33.8 | | 57 | | 0 | | United State | | Human | |
| M35954_p2 | | 12922 | | 37.0 | | 18 | | 0 | | United State | | Human | |
| M64191_p4 | | 60405 | | 32.7 | | 81 | | 0 | | United State | | Human | |
| M64191_p5 | | 46375 | | 32.7 | | 68 | | 0 | | United State | | Human | |
| MAL9_p8 | | 25928 | | 34.8 | | 38 | | 0 | | Malaysia | | Human | |
| MAL9_p9 | | 76364 | | 31.5 | | 113 | | 0 | | Malaysia | | Human | |
| MAL11_p4 | | 110532 | | 33.1 | | 102 | | 0 | | Malaysia | | Human | |
| MAL11_p9 | | 66137 | | 31.7 | | 106 | | 0 | | Malaysia | | Human | |
| MI_p2 | | 69498 | | 33.7 | | 99 | | 0 | | United State | | Human | |
| MI_p4 | | 62741 | | 33.0 | | 87 | | 0 | | United State | | Human | |
| MI_p6 | | 46660 | | 32.8 | | 73 | | 0 | | United State | | Human | |
| Mq2T_p3 | | 37844 | | 34.3 | | 57 | | 0 | | India | | Human | |
| MR1_p1 | | 27907 | | 32.7 | | 36 | | 0 | | Poland | | Human | |
| MRGR3_p8 | | 26621 | | 36.5 | | 31 | | 0 | | Switzerland | | Human | |
| MRSA_041T_B_p2 | | 65581 | | 33.7 | | 92 | | 0 | | Tanzania | | Unknown | |
| MRSA_FGWU_p3 | | 48645 | | 33.9 | | 74 | | 0 | | United Kingdom | | Human | |
| MRSA_FKPC_p2 | | 52554 | | 32.8 | | 80 | | 0 | | United State | | Human | |
| MRSA_FKPC_p4 | | 21508 | | 35.9 | | 28 | | 0 | | United State | | Human | |
| MRSA_FKSE_p1 | | 47305 | | 33.1 | | 72 | | 0 | | United State | | Human | |
| MRSA_FKSE_p2 | | 41603 | | 34.0 | | 50 | | 0 | | United State | | Human | |
| MRSA_FKTK_p4 | | 50783 | | 32.7 | | 75 | | 0 | | United State | | Human | |
| MRSA_FKTN_p1 | | 46876 | | 33.5 | | 67 | | 0 | | United State | | Human | |
| MRSA_FKUV_p3 | | 49067 | | 32.7 | | 69 | | 0 | | United State | | Human | |
| MRSA_FKVP_p1 | | 33851 | | 32.6 | | 42 | | 0 | | United State | | Human | |
| MRSA_FKWH_p4 | | 46669 | | 32.8 | | 70 | | 0 | | United State | | Human | |
| MRSA_FKYJ_p1 | | 69793 | | 33.8 | | 92 | | 0 | | United State | | Human | |
| MRSA_S1_p1 | | 60406 | | 32.7 | | 80 | | 0 | | Switzerland | | Human | |
| MRSA_S1_p5 | | 68815 | | 31.7 | | 93 | | 0 | | Switzerland | | Human | |
| MRSA_S2_p1 | | 60406 | | 32.7 | | 80 | | 0 | | Switzerland | | Human | |
| MRSA_S2_p5 | | 68815 | | 31.7 | | 93 | | 0 | | Switzerland | | Human | |
| MRSA_S4_p4 | | 55451 | | 32.2 | | 78 | | 0 | | Switzerland | | Human | |
| MRSA_S4_p5 | | 38458 | | 33.6 | | 49 | | 0 | | Switzerland | | Human | |
| MRSA_S5_p3 | | 60405 | | 32.7 | | 81 | | 0 | | Switzerland | | Human | |
| MRSA_S5_p5 | | 55451 | | 32.2 | | 78 | | 0 | | Switzerland | | Human | |
| MRSA08_p2 | | 74258 | | 33.3 | | 98 | | 0 | | Tanzania | | Unknown | |
| MRSA08_p3 | | 32860 | | 33.8 | | 39 | | 0 | | Tanzania | | Unknown | |
| MRSA012TA_p2 | | 53088 | | 34.7 | | 74 | | 0 | | Tanzania | | Unknown | |
| MRSA012TB_p2 | | 58119 | | 34.1 | | 79 | | 0 | | Tanzania | | Unknown | |
| MRSA012TB_p3 | | 56694 | | 32.6 | | 82 | | 0 | | Tanzania | | Unknown | |
| MRSA071_p2 | | 64403 | | 33.9 | | 92 | | 0 | | Tanzania | | Unknown | |
| MRSA079A_p1 | | 58119 | | 34.1 | | 79 | | 8 | | Tanzania | | Unknown | |
| MRSA207_p2 | | 30615 | | 35.5 | | 38 | | 0 | | Tanzania | | Unknown | |
| MRSA207_p4 | | 56933 | | 33.5 | | 78 | | 0 | | Tanzania | | Unknown | |
| MRSA252_p2 | | 33849 | | 32.6 | | 42 | | 0 | | United Kingdom | | Human | |
| MRSA252_p3 | | 58086 | | 33.1 | | 82 | | 0 | | United Kingdom | | Human | |
| MRSA252_p5 | | 51693 | | 32.6 | | 73 | | 0 | | United Kingdom | | Human | |
| MRSA035668_p3 | | 68175 | | 32.8 | | 89 | | 0 | | Tanzania | | Unknown | |
| MS4_p3 | | 109056 | | 33.0 | | 178 | | 0 | | China | | Human | |
| MS4_LZD100_p1 | | 37026 | | 33.8 | | 64 | | 0 | | China | | Human | |
| MS4_T10_p1 | | 65360 | | 33.0 | | 98 | | 0 | | China | | Unknown | |
| MS4_T60_p1 | | 42291 | | 33.4 | | 75 | | 0 | | China | | Unknown | |
| MSSA_FKNV_p1 | | 74701 | | 33.5 | | 103 | | 0 | | United State | | Human | |
| MSSA_FKNV_p2 | | 57164 | | 32.2 | | 84 | | 0 | | United State | | Human | |
| MSSA_FKOF_p2 | | 53455 | | 34.0 | | 73 | | 0 | | United State | | Human | |
| MSSA_FKTB_p2 | | 57461 | | 32.3 | | 81 | | 0 | | United State | | Human | |
| MSSA1_p1 | | 31257 | | 35.2 | | 40 | | 0 | | United State | | Human | |
| MSSA476_p1 | | 54898 | | 33.2 | | 70 | | 0 | | Unknown | | Human | |
| MU3_p3 | | 69889 | | 33.6 | | 101 | | 0 | | Japan | | Human | |
| MU4_p4 | | 23012 | | 35.8 | | 29 | | 0 | | Turkey | | Human | |
| MU4_p9 | | 110102 | | 32.8 | | 179 | | 0 | | Turkey | | Human | |
| Mu50_p3 | | 69890 | | 33.5 | | 101 | | 0 | | Japan | | Unknown | |
| MUM270_p1 | | 34648 | | 35.1 | | 60 | | 0 | | India | | Human | |
| MUM270_p2 | | 34218 | | 34.2 | | 58 | | 0 | | India | | Human | |
| MUM475_p1 | | 73979 | | 31.7 | | 95 | | 0 | | India | | Human | |
| MUM475_p2 | | 52861 | | 33.8 | | 82 | | 0 | | India | | Human | |
| MW2_p2 | | 62657 | | 32.6 | | 82 | | 0 | | United State | | Human | |
| N315_p4 | | 47584 | | 32.6 | | 68 | | 0 | | Japan | | Human | |
| Na21_p7 | | 24431 | | 36.3 | | 29 | | 0 | | Sri Lanka | | Human | |
| NA32_p4 | | 50901 | | 33.1 | | 60 | | 0 | | Denmark | | Human | |
| NA32_p5 | | 36189 | | 34.7 | | 48 | | 0 | | Denmark | | Human | |
| NCTC_8325_p3 | | 60343 | | 32.8 | | 81 | | 0 | | Unknown | | Unknown | |
| NCTC_8325_p4 | | 59060 | | 33.9 | | 85 | | 0 | | Unknown | | Unknown | |
| NCTC_8325_p5 | | 46049 | | 33.3 | | 72 | | 0 | | Unknown | | Unknown | |
| Newman_p1 | | 42971 | | 34.7 | | 69 | | 0 | | Unknown | | Unknown | |
| Newman_p3 | | 49576 | | 34.1 | | 72 | | 0 | | Unknown | | Unknown | |
| Newman_p5 | | 44720 | | 34.1 | | 69 | | 0 | | Unknown | | Unknown | |
| Newman_p6 | | 47387 | | 32.9 | | 68 | | 0 | | Unknown | | Unknown | |
| NGS_ED_1006_p2 | | 61767 | | 32.6 | | 87 | | 0 | | United Kingdom | | Human | |
| NML151290_p1 | | 71404 | | 33.0 | | 96 | | 0 | | Canada | | Human | |
| NML151290_p2 | | 55676 | | 32.5 | | 85 | | 0 | | Canada | | Human | |
| NMR05_p5 | | 26435 | | 36.6 | | 31 | | 0 | | India | | Human | |
| NRS2_p2 | | 46652 | | 33.8 | | 65 | | 0 | | Japan | | Human | |
| NRS2_p5 | | 61802 | | 33.0 | | 82 | | 0 | | Japan | | Human | |
| NRS2_p7 | | 39713 | | 33.0 | | 48 | | 0 | | Japan | | Human | |
| NRS158_p1 | | 55675 | | 32.6 | | 86 | | 0 | | France | | Human | |
| NRS158_p2 | | 74217 | | 32.7 | | 95 | | 0 | | France | | Human | |
| O11_p4 | | 25666 | | 34.0 | | 27 | | 0 | | France | | Sheep | |
| O46_p2 | | 25657 | | 31.5 | | 37 | | 0 | | France | | Sheep | |
| O46_p3 | | 32744 | | 35.5 | | 44 | | 0 | | France | | Sheep | |
| OC3_p2 | | 28365 | | 34.8 | | 33 | | 0 | | Russia | | Human | |
| OC3_p5 | | 29829 | | 35.7 | | 40 | | 0 | | Russia | | Human | |
| OCMM6035_p1 | | 43097 | | 34.1 | | 62 | | 0 | | United State | | Human | |
| P32_p6 | | 61448 | | 34.0 | | 72 | | 0 | | Poland | | Human | |
| P151_p6 | | 16391 | | 36.8 | | 19 | | 0 | | Malaysia | | Human | |
| QR502_p1 | | 64935 | | 33.0 | | 90 | | 0 | | China | | Human | |
| 08_02300_p3 | | 46489 | | 33.0 | | 66 | | 0 | | Germany | | Human | |
| R0294_p5 | | 34934 | | 35.6 | | 45 | | 0 | | China | | Other | |
| R0294_p6 | | 21671 | | 35.2 | | 25 | | 0 | | China | | Other | |
| R0353_p2 | | 36148 | | 34.5 | | 39 | | 0 | | China | | Other | |
| R0353_p6 | | 34543 | | 35.5 | | 43 | | 0 | | China | | Other | |
| RA3_p4 | | 28338 | | 35.1 | | 35 | | 0 | | Argentina | | Human | |
| RA3_p5 | | 34366 | | 33.3 | | 37 | | 0 | | Argentina | | Human | |
| Rd_3_p2 | | 57221 | | 34.0 | | 73 | | 0 | | Germany | | Bovine | |
| RH_0600_0125_09_p2 | | 62387 | | 32.1 | | 85 | | 0 | | United Kingdom | | Human | |
| RIVM1295_p2 | | 46926 | | 33.2 | | 74 | | 0 | | Netherlands | | Human | |
| RIVM3897_p1 | | 32083 | | 33.5 | | 36 | | 0 | | Netherlands | | Human | |
| RIVM3897_p3 | | 71652 | | 33.0 | | 92 | | 0 | | Netherlands | | Human | |
| RIVM3897_p4 | | 59285 | | 34.4 | | 83 | | 0 | | Netherlands | | Human | |
| S54F9_p1 | | 52054 | | 34.4 | | 72 | | 0 | | Denmark | | Swine | |
| S56_POEL_p2 | | 28502 | | 36.1 | | 35 | | 0 | | Belgium | | Chicken | |
| S56_POEL_p5 | | 28671 | | 33.9 | | 36 | | 0 | | Belgium | | Chicken | |
| S0385_p1 | | 46969 | | 33.6 | | 67 | | 0 | | Australia | | Human | |
| S0385_p4 | | 50783 | | 33.4 | | 72 | | 0 | | Australia | | Human | |
| S2396_p4 | | 36402 | | 33.1 | | 39 | | 0 | | France | | Human | |
| S2396_p7 | | 30077 | | 32.7 | | 35 | | 0 | | France | | Human | |
| S2397_p6 | | 31024 | | 34.1 | | 39 | | 0 | | France | | Human | |
| S2397_p7 | | 16198 | | 34.3 | | 24 | | 0 | | France | | Human | |
| S2398_p6 | | 30982 | | 34.1 | | 39 | | 0 | | France | | Human | |
| SA_067_p3 | | 34092 | | 33.6 | | 55 | | 0 | | Switzerland | | Other | |
| SA_083_p2 | | 56379 | | 32.6 | | 82 | | 0 | | Switzerland | | Other | |
| SA_083_p3 | | 48966 | | 35.2 | | 75 | | 0 | | Switzerland | | Other | |
| SA_085_p2 | | 45165 | | 33.4 | | 68 | | 0 | | Switzerland | | Other | |
| SA_085_p3 | | 44815 | | 35.1 | | 71 | | 0 | | Switzerland | | Other | |
| SA_120_p4 | | 42488 | | 33.6 | | 45 | | 0 | | Switzerland | | Other | |
| SA_210_p1 | | 61722 | | 31.9 | | 83 | | 0 | | Switzerland | | Other | |
| SA_260_p1 | | 24188 | | 36.0 | | 37 | | 0 | | Switzerland | | Other | |
| SA3_LAU_p2 | | 30753 | | 35.2 | | 38 | | 0 | | Lebanon | | Human | |
| SA8_LAU_p5 | | 16707 | | 35.9 | | 23 | | 0 | | Jordan | | Human | |
| SA11_LAU_p4 | | 33996 | | 35.6 | | 47 | | 0 | | Jordan | | Human | |
| Sa12_001_p1 | | 52025 | | 34.2 | | 70 | | 0 | | Australia | | Bovine | |
| Sa13_005_p4 | | 38325 | | 35.0 | | 42 | | 0 | | Australian | | Sheep | |
| Sa13_006_p2 | | 30113 | | 33.9 | | 33 | | 0 | | Australian | | Bovine | |
| Sa13_006_p4 | | 28142 | | 35.3 | | 32 | | 0 | | Australian | | Bovine | |
| Sa14_002_p2 | | 49806 | | 33.6 | | 65 | | 0 | | Australia | | Bovine | |
| Sa14_003_p1 | | 56846 | | 32.3 | | 84 | | 0 | | Australia | | Bovine | |
| Sa14_004_p3 | | 41854 | | 35.0 | | 62 | | 0 | | Australia | | Bovine | |
| SA268_p3 | | 65271 | | 33.0 | | 97 | | 0 | | China | | Human | |
| SA957_p3 | | 66881 | | 32.9 | | 101 | | 0 | | China | | Human | |
| SAHPchr_p2 | | 52398 | | 33.5 | | 77 | | 0 | | China | | Swine | |
| SARM_C5621_p2 | | 70909 | | 33.2 | | 103 | | 0 | | Spain | | Human | |
| SCCmec1_p2 | | 62771 | | 32.1 | | 82 | | 0 | | Denmark | | Human | |
| SCCmec1_p5 | | 33850 | | 32.6 | | 42 | | 0 | | Denmark | | Human | |
| SCCmec1_p6 | | 57380 | | 33.0 | | 81 | | 0 | | Denmark | | Human | |
| SR434_p4 | | 44240 | | 35.1 | | 69 | | 0 | | China | | Human | |
| st436_p2 | | 46859 | | 34.1 | | 68 | | 0 | | United Kingdom | | Human | |
| st534_p2 | | 56802 | | 33.3 | | 81 | | 0 | | Ireland | | Human | |
| ST772_MRSA_V_p2 | | 56919 | | 32.9 | | 85 | | 0 | | Australia | | Human | |
| st1335_p1 | | 61943 | | 32.9 | | 86 | | 0 | | United Kingdom | | Human | |
| 10S_p1 | | 15565 | | 31.0 | | 25 | | 0 | | Pakistan | | Human | |
| st1358_p1 | | 60355 | | 32.9 | | 77 | | 0 | | Ireland | | Human | |
| st1358_p2 | | 38399 | | 31.1 | | 55 | | 0 | | Ireland | | Human | |
| st1358_p3 | | 27899 | | 31.2 | | 43 | | 0 | | Ireland | | Human | |
| st1424_p1 | | 57466 | | 32.0 | | 83 | | 0 | | United Kingdom | | Human | |
| st1470_p1 | | 69398 | | 33.1 | | 93 | | 0 | | United Kingdom | | Human | |
| st1520_p2 | | 56236 | | 34.4 | | 86 | | 0 | | United Kingdom | | Human | |
| st1607_p1 | | 61943 | | 32.9 | | 86 | | 0 | | Ireland | | Human | |
| st1607_p3 | | 47479 | | 32.9 | | 68 | | 0 | | Ireland | | Human | |
| st1815_p2 | | 65429 | | 32.9 | | 86 | | 0 | | Ireland | | Human | |
| st2436_p3 | | 48645 | | 33.9 | | 74 | | 0 | | United Kingdom | | Human | |
| st2534_p1 | | 58086 | | 33.1 | | 82 | | 0 | | United Kingdom | | Human | |
| st2534_p4 | | 26567 | | 36.1 | | 32 | | 0 | | United Kingdom | | Human | |
| st2543_p1 | | 61942 | | 32.9 | | 86 | | 0 | | United Kingdom | | Human | |
| st2543_p2 | | 49066 | | 32.7 | | 69 | | 0 | | United Kingdom | | Human | |
| st2787_p1 | | 56162 | | 32.3 | | 80 | | 0 | | United Kingdom | | Human | |
| st3047_p2 | | 57207 | | 33.1 | | 88 | | 0 | | United Kingdom | | Human | |
| st3047_p3 | | 44057 | | 34.1 | | 69 | | 0 | | United Kingdom | | Human | |
| ST20130943_p1 | | 47507 | | 33.3 | | 73 | | 0 | | Brazil | | Human | |
| T0131_p4 | | 71346 | | 31.8 | | 94 | | 0 | | China | | Human | |
| T44444_p1 | | 58464 | | 33.0 | | 79 | | 0 | | United State | | Human | |
| T59618_p1 | | 47780 | | 33.1 | | 78 | | 0 | | United State | | Human | |
| Tager_104_p1 | | 57757 | | 32.2 | | 84 | | 0 | | United State | | Human | |
| TCH60_p3 | | 63178 | | 32.8 | | 82 | | 0 | | United State | | Human | |
| TSAR01_p2 | | 61952 | | 32.9 | | 84 | | 0 | | China | | Human | |
| TSAR02_p1 | | 63241 | | 33.2 | | 88 | | 0 | | China | | Human | |
| TSAR04_p3 | | 64039 | | 33.1 | | 90 | | 0 | | China | | Human | |
| TSAR05_p1 | | 31716 | | 35.2 | | 40 | | 0 | | China | | Human | |
| TSAR05_p2 | | 33160 | | 33.7 | | 43 | | 0 | | China | | Human | |
| TSAR08_p2 | | 44650 | | 33.6 | | 75 | | 0 | | China | | Human | |
| Tur_15_p3 | | 30341 | | 33.7 | | 35 | | 0 | | Austria | | Chicken | |
| Tur_20_p2 | | 31777 | | 34.3 | | 45 | | 0 | | Germany | | Chicken | |
| Tur_22_p2 | | 27399 | | 36.4 | | 41 | | 0 | | Italy | | Chicken | |
| TW20_p2 | | 43122 | | 35.1 | | 64 | | 0 | | United Kingdom | | Unknown | |
| TW20_p6 | | 76948 | | 31.8 | | 101 | | 0 | | United Kingdom | | Unknown | |
| TW20_p7 | | 134279 | | 30.9 | | 161 | | 0 | | United Kingdom | | Unknown | |
| UAMS_1_p2 | | 50874 | | 32.6 | | 72 | | 0 | | United State | | Human | |
| USA_1_p2 | | 45164 | | 33.6 | | 64 | | 0 | | United State | | Human | |
| USA_1_p5 | | 26900 | | 36.0 | | 31 | | 0 | | United State | | Human | |
| USA_15_p2 | | 71273 | | 33.0 | | 90 | | 0 | | United State | | Human | |
| USA_15_p3 | | 46336 | | 34.9 | | 70 | | 0 | | United State | | Human | |
| USA300_2014.C01_p1 | | 63254 | | 32.6 | | 83 | | 0 | | United State | | Human | |
| USA300_2014.C01_p5 | | 55449 | | 32.2 | | 78 | | 0 | | United State | | Human | |
| USA300_2014.C01_p6 | | 66345 | | 33.3 | | 90 | | 0 | | United State | | Human | |
| USA300_2014.C02_p2 | | 55448 | | 32.2 | | 79 | | 0 | | United State | | Human | |
| USA300_2014.C02_p3 | | 63254 | | 32.6 | | 83 | | 0 | | United State | | Human | |
| USA300_FPR3757_p4 | | 60405 | | 32.7 | | 81 | | 0 | | United State | | Human | |
| USA300_FPR3757_p5 | | 46376 | | 32.7 | | 68 | | 0 | | United State | | Human | |
| USA300_TCH959_p1 | | 46399 | | 33.0 | | 67 | | 0 | | United State | | Human | |
| USA300_TCH959_p2 | | 72734 | | 33.8 | | 102 | | 0 | | United State | | Human | |
| USA300_TCH1516_p4 | | 60405 | | 32.7 | | 81 | | 0 | | United State | | Human | |
| USA300_TCH1516_p5 | | 46375 | | 32.7 | | 68 | | 0 | | United State | | Human | |
| USFL022_p4 | | 28992 | | 35.9 | | 36 | | 0 | | United State | | Human | |
| USFL022_p7 | | 33375 | | 33.6 | | 37 | | 0 | | United State | | Human | |
| USFL190_p1 | | 48924 | | 33.9 | | 69 | | 0 | | United State | | Human | |
| USFL246_p3 | | 60436 | | 33.0 | | 81 | | 0 | | United State | | Human | |
| USFL246_p5 | | 55450 | | 32.2 | | 79 | | 0 | | United State | | Human | |
| USFL253_p6 | | 26008 | | 36.3 | | 30 | | 0 | | United State | | Human | |
| UTSW_MRSA_55_p2 | | 60405 | | 32.7 | | 81 | | 0 | | United State | | Human | |
| UTSW_MRSA_55_p3 | | 70093 | | 31.6 | | 94 | | 0 | | United State | | Human | |
| V808_p1 | | 31366 | | 36.3 | | 37 | | 0 | | Korea | | Human | |
| V1127_p8 | | 15455 | | 36.9 | | 18 | | 0 | | Korea | | Human | |
| V1142_p2 | | 27539 | | 35.0 | | 34 | | 0 | | Korea | | Human | |
| V1859_p4 | | 60403 | | 32.7 | | 81 | | 0 | | Venezuela | | Human | |
| V1859_p5 | | 55341 | | 32.4 | | 76 | | 0 | | Venezuela | | Human | |
| VB26276_p2 | | 27544 | | 34.7 | | 38 | | 0 | | India | | Human | |
| VET0051R_p1 | | 70721 | | 33.0 | | 97 | | 0 | | Netherlands | | Human | |
| VET0889S_p2 | | 33851 | | 32.6 | | 42 | | 0 | | Netherlands | | Human | |
| VET1518S_p1 | | 33851 | | 32.6 | | 42 | | 0 | | Netherlands | | Human | |
| VH221_p4 | | 29811 | | 35.6 | | 40 | | 0 | | India | | Human | |
| W25799_p1 | | 40384 | | 33.5 | | 60 | | 0 | | United State | | Human | |
| W33563_p2 | | 34042 | | 32.9 | | 39 | | 0 | | United State | | Human | |
| W33563_p3 | | 55361 | | 32.5 | | 73 | | 0 | | United State | | Human | |
| W45755_111412_p6 | | 9424 | | 36.7 | | 15 | | 0 | | United State | | Human | |
| W82303_p3 | | 26835 | | 36.2 | | 32 | | 0 | | United State | | Human | |
| XN108_p5 | | 63611 | | 34.0 | | 91 | | 0 | | China | | Human | |
| XN108_p6 | | 77063 | | 31.8 | | 104 | | 0 | | China | | Human | |
| XN108_p7 | | 132584 | | 30.9 | | 163 | | 0 | | China | | Human | |
| XQ_p1 | | 42468 | | 30.1 | | 55 | | 0 | | China | | Human | |
| XQ_p2 | | 62460 | | 32.9 | | 88 | | 0 | | China | | Human | |
| XQ_p3 | | 35015 | | 32.2 | | 44 | | 0 | | China | | Human | |
| Z172_p4 | | 41481 | | 35.9 | | 62 | | 0 | | China | | Human | |
| Z172_p6 | | 76947 | | 31.8 | | 101 | | 0 | | China | | Human | |
| ZJ5499_p4 | | 62431 | | 32.8 | | 85 | | 0 | | China | | Human | |
| ZJ5499_p5 | | 45962 | | 32.9 | | 68 | | 0 | | China | | Human | |
| **Outgroup** |  | |  | |  | |  | |  | |  | |  |
| Erwinia_phage_phiEa2809 (NC_027340.1) | | |  | |  | |  | |  | |  | |  |

Table S3 **Metadata for** **1389 incomplete phages predicted in the genomes of *S. aureus* and outgroup phage Erwinia_phage_phiEa2809 (NC_027340.1).**

| **Incomplete prophage** | **Genome length (bp)** | **GC content (%)** | **ORFs** | **tRNA** | **Geographic region** | **Host** |
| --- | --- | --- | --- | --- | --- | --- |
| 03_01478_ip2 | 22341 | 31.5 | 24 | 0 | Germany | Human |
| 04_00608_ip2 | 30616 | 32.1 | 32 | 0 | Germany | Human |
| 04_00608_ip3 | 9342 | 26.5 | 11 | 0 | Germany | Human |
| 04_02314_1_ip1 | 15207 | 33.4 | 15 | 0 | United Kingdom | Human |
| 04_02314_1_ip2 | 27389 | 31.3 | 27 | 0 | United Kingdom | Human |
| 04_02314_1_ip3 | 9342 | 26.5 | 11 | 0 | United Kingdom | Human |
| 04_02314_1_ip5 | 8700 | 31.3 | 16 | 0 | United Kingdom | Human |
| 04_02981_ip1 | 22719 | 34.1 | 24 | 0 | Germany | Human |
| 04_02981_ip3 | 6113 | 28.6 | 7 | 0 | Germany | Human |
| 04_02981_ip4 | 18829 | 27.7 | 21 | 0 | Germany | Human |
| 04_03103_ip1 | 36486 | 32 | 51 | 0 | Germany | Human |
| 04_03111_ip3 | 9341 | 26.5 | 11 | 0 | Germany | Human |
| 5R_157_2_ip1 | 41252 | 30.3 | 57 | 0 | Mexico | Human |
| 5R_157_2_ip3 | 29820 | 33.3 | 43 | 0 | Mexico | Human |
| 06_01900_ip1 | 22511 | 32.2 | 37 | 0 | Germany | Human |
| 06_01900_ip2 | 43913 | 31.5 | 56 | 0 | Germany | Human |
| 06_01900_ip3 | 7122 | 30.3 | 9 | 0 | Germany | Human |
| 06_01900_ip5 | 27741 | 33.8 | 33 | 0 | Germany | Human |
| 06_02400_ip1 | 29906 | 32.2 | 43 | 0 | Germany | Human |
| 06_02400_ip3 | 9370 | 26.5 | 12 | 0 | Germany | Human |
| 06BA18369_ip1 | 11496 | 33.1 | 16 | 0 | Canada | Human |
| 06BA18369_ip2 | 18920 | 31.6 | 22 | 0 | Canada | Human |
| 06BA18369_ip3 | 12499 | 32.6 | 19 | 0 | Canada | Human |
| 06BA18369_ip4 | 18830 | 27.7 | 21 | 0 | Canada | Human |
| 06BA18369_ip5 | 17663 | 34.5 | 22 | 0 | Canada | Human |
| 06BA18369_ip6 | 24696 | 34.1 | 40 | 0 | Canada | Human |
| 06BA18369_ip7 | 17022 | 31.5 | 20 | 0 | Canada | Human |
| 07_00058_ip1 | 39801 | 31.5 | 56 | 0 | Germany | Human |
| 07_00058_ip2 | 9341 | 26.5 | 11 | 0 | Germany | Human |
| 07_00058_ip3 | 25485 | 32.1 | 38 | 0 | Germany | Human |
| 07_00059_ip2 | 9341 | 26.5 | 11 | 0 | Germany | Human |
| 07_00059_ip4 | 5323 | 28.5 | 7 | 0 | Germany | Human |
| 07_00309_ip2 | 17241 | 30.5 | 18 | 0 | Germany | Human |
| 07_00309_ip4 | 9342 | 26.6 | 11 | 0 | Germany | Human |
| 07_00655_ip2 | 30027 | 32.3 | 44 | 0 | Germany | Human |
| 07_00655_ip3 | 17246 | 30.4 | 18 | 0 | Germany | Human |
| 07_00655_ip4 | 10569 | 27.2 | 12 | 0 | Germany | Human |
| 07_01497_ip1 | 43616 | 32.1 | 56 | 0 | Germany | Human |
| 07_02789_ip1 | 30066 | 32.3 | 45 | 0 | Germany | Human |
| 07_02789_ip2 | 17250 | 30.4 | 18 | 0 | Germany | Human |
| 07_02997_ip1 | 19351 | 31.3 | 20 | 0 | Australia | Human |
| 07_02997_ip2 | 27010 | 32.4 | 39 | 0 | Australia | Human |
| 07_02997_ip3 | 33911 | 30.9 | 48 | 0 | Australia | Human |
| 07_03339_ip3 | 5066 | 29.9 | 9 | 0 | Czech Republic | Human |
| 07_03339_ip4 | 12402 | 28.2 | 13 | 0 | Czech Republic | Human |
| 07_03345_ip3 | 19011 | 31.6 | 31 | 0 | Czech Republic | Human |
| 07_03346_ip2 | 51086 | 32.4 | 66 | 0 | Czech Republic | Human |
| 07_03346_ip4 | 12237 | 27.7 | 14 | 0 | Czech Republic | Human |
| 07_03349_ip2 | 5827 | 28.1 | 8 | 0 | Denmark | Human |
| 07_03349_ip3 | 16512 | 30.7 | 28 | 0 | Denmark | Human |
| 07_03354_2_ip2 | 9342 | 26.5 | 11 | 0 | Denmark | Human |
| 08_01304_ip1 | 22248 | 32.1 | 36 | 0 | Germany | Human |
| 08_01304_ip3 | 7663 | 26.7 | 9 | 0 | Germany | Human |
| 08_01483_ip1 | 35197 | 30.2 | 38 | 0 | Portugal | Human |
| 08_01483_ip3 | 14937 | 33.3 | 28 | 0 | Portugal | Human |
| 08_01486_ip1 | 17240 | 30.5 | 18 | 0 | Portugal | Human |
| 08_01486_ip2 | 33033 | 31 | 30 | 0 | Portugal | Human |
| 08_01486_ip3 | 9342 | 26.6 | 11 | 0 | Portugal | Human |
| 08_01486_ip4 | 28170 | 31.6 | 43 | 0 | Portugal | Human |
| 08_01667_ip2 | 25807 | 31.9 | 41 | 0 | Germany | Human |
| 08_01667_ip3 | 11615 | 28.7 | 11 | 0 | Germany | Human |
| 08_01668_ip1 | 48074 | 31.3 | 65 | 0 | Germany | Human |
| 08_01668_ip3 | 17240 | 30.5 | 18 | 0 | Germany | Human |
| 08_01668_ip4 | 4252 | 31.6 | 11 | 0 | Germany | Human |
| 08_01668_ip5 | 9341 | 26.5 | 11 | 0 | Germany | Human |
| 08_01669_ip2 | 31823 | 33.2 | 46 | 0 | Denmark | Human |
| 08_01669_ip4 | 24468 | 33 | 20 | 0 | Denmark | Human |
| 08_01669_ip5 | 12551 | 27.9 | 16 | 0 | Denmark | Human |
| 08_01671_ip2 | 29604 | 30.9 | 32 | 0 | Germany | Human |
| 08_01671_ip4 | 9369 | 26.5 | 11 | 0 | Germany | Human |
| 08_02119_ip1 | 16253 | 30.2 | 16 | 0 | Germany | Human |
| 08_02119_ip4 | 19684 | 33.4 | 24 | 0 | Germany | Human |
| 08_02300_ip1 | 7795 | 26.5 | 15 | 0 | Germany | Human |
| 08_02300_ip2 | 31060 | 29.3 | 35 | 0 | Germany | Human |
| 11P4_ip2 | 6760 | 25.5 | 9 | 0 | Netherlands | Swine |
| 11P8_ip1 | 6760 | 25.5 | 9 | 0 | Netherlands | Swine |
| 11P8_ip2 | 13799 | 33 | 20 | 0 | Netherlands | Swine |
| 14_11MN_17_08_66_05_ip1 | 6760 | 25.5 | 9 | 0 | Netherlands | Human |
| 25_2889_ip2 | 16412 | 32 | 16 | 0 | Netherlands | Human |
| 25_2889_ip4 | 6760 | 25.5 | 9 | 0 | Netherlands | Human |
| 25_2889_ip5 | 27806 | 33.5 | 44 | 0 | Netherlands | Human |
| 25_2889_ip6 | 10477 | 34.3 | 25 | 0 | Netherlands | Human |
| 36P1_ip2 | 16412 | 32 | 16 | 0 | Netherlands | Swine |
| 36P1_ip3 | 6760 | 25.4 | 9 | 0 | Netherlands | Swine |
| 40P5_1_1_ip1 | 6760 | 25.5 | 9 | 0 | Netherlands | Swine |
| 40P5_1_1_ip3 | 11060 | 33.1 | 23 | 0 | Netherlands | Swine |
| 42S_ip2 | 28761 | 32.8 | 33 | 0 | Pakistan | Human |
| 043H_ip2 | 18415 | 31.5 | 24 | 0 | Tanzania | Unknown |
| 043H_ip3 | 17917 | 29.1 | 21 | 0 | Tanzania | Unknown |
| 043H_ip5 | 10795 | 31.3 | 18 | 0 | Tanzania | Unknown |
| 043H_ip6 | 29169 | 33.7 | 42 | 0 | Tanzania | Unknown |
| 43P8_ip1 | 16411 | 32 | 16 | 0 | Netherlands | Swine |
| 43P8_ip2 | 6760 | 25.5 | 9 | 0 | Netherlands | Swine |
| 51S_ip1 | 18370 | 32 | 26 | 0 | Pakistan | Human |
| 51S_ip2 | 9222 | 29.7 | 18 | 0 | Pakistan | Human |
| 57_ip1 | 45151 | 30.5 | 48 | 2 | China | Swine |
| 57_ip3 | 24024 | 28.2 | 30 | 0 | China | Swine |
| 065H_ip2 | 17743 | 31.7 | 25 | 0 | Tanzania | Unknown |
| 065H_ip3 | 18830 | 27.7 | 21 | 0 | Tanzania | Unknown |
| 065H_ip4 | 30234 | 34.3 | 45 | 0 | Tanzania | Unknown |
| 065H_ip5 | 8207 | 29.7 | 13 | 0 | Tanzania | Unknown |
| 69_SAUR_ip1 | 18831 | 27.7 | 21 | 0 | United State | Human |
| 69_SAUR_ip2 | 21478 | 31.5 | 28 | 0 | United State | Human |
| 71A_S11_ip1 | 9035 | 28.6 | 17 | 0 | South Africa | Human |
| 71A_S11_ip2 | 32722 | 31.6 | 36 | 0 | South Africa | Human |
| 71A_S11_ip4 | 6133 | 33 | 10 | 0 | South Africa | Human |
| 71A_S11_ip5 | 21574 | 31.8 | 20 | 0 | South Africa | Human |
| 75_ip1 | 10928 | 31.9 | 16 | 0 | Finland | Bovine |
| 75_ip2 | 22987 | 32.2 | 26 | 0 | Finland | Bovine |
| 75_ip4 | 12192 | 26.7 | 13 | 0 | Finland | Bovine |
| 79_S10_ip1 | 21006 | 30.8 | 26 | 0 | South Africa | Human |
| 81_SAUR_ip2 | 29468 | 31.9 | 42 | 0 | United State | Human |
| 81_SAUR_ip3 | 8391 | 30.5 | 15 | 0 | United State | Human |
| 81_SAUR_ip5 | 18831 | 27.7 | 21 | 0 | United State | Human |
| 92_ip1 | 7252 | 31.7 | 11 | 0 | China | Swine |
| 92_ip2 | 7796 | 26.5 | 15 | 0 | China | Swine |
| 92_ip3 | 5844 | 31.6 | 7 | 0 | China | Swine |
| 92_ip4 | 16019 | 30.5 | 17 | 0 | China | Swine |
| 107_ip1 | 45151 | 30.5 | 48 | 2 | China | Swine |
| 107_ip2 | 7796 | 26.5 | 15 | 0 | China | Swine |
| 107_ip4 | 24024 | 28.2 | 30 | 0 | China | Swine |
| 110_ip1 | 10868 | 31.9 | 14 | 0 | Finland | Bovine |
| 110_ip2 | 15670 | 34.4 | 17 | 0 | Finland | Bovine |
| 110_ip3 | 12191 | 26.7 | 13 | 0 | Finland | Bovine |
| 110_ip4 | 22930 | 32.1 | 26 | 0 | Finland | Bovine |
| 112_ip2 | 9235 | 31.8 | 9 | 0 | Finland | Bovine |
| 112_ip3 | 12191 | 26.7 | 13 | 0 | Finland | Bovine |
| 112_ip4 | 10928 | 31.9 | 15 | 0 | Finland | Bovine |
| 118_ip1 | 28761 | 32.8 | 33 | 0 | India | Human |
| 120_ip2 | 22932 | 32.1 | 26 | 0 | Belgium | Bovine |
| 125_ip1 | 45151 | 30.5 | 48 | 2 | China | Swine |
| 125_ip2 | 7796 | 26.5 | 15 | 0 | China | Swine |
| 125_ip4 | 24024 | 28.2 | 30 | 0 | China | Swine |
| 169_SAUR_ip2 | 17811 | 30.5 | 19 | 0 | United State | Human |
| 170_ip1 | 16960 | 32.5 | 23 | 0 | Brazil | Bovine |
| 170_ip3 | 10726 | 32.4 | 16 | 0 | Brazil | Bovine |
| 170_ip4 | 6326 | 35.9 | 6 | 0 | Brazil | Bovine |
| 170_ip5 | 3325 | 34.1 | 14 | 0 | Brazil | Bovine |
| 170_ip6 | 4571 | 34.9 | 16 | 0 | Brazil | Bovine |
| 229_SAUR_ip1 | 18898 | 30 | 32 | 0 | United State | Human |
| 229_SAUR_ip2 | 8100 | 33.8 | 18 | 0 | United State | Human |
| 229_SAUR_ip4 | 26596 | 33.1 | 32 | 0 | United State | Human |
| 299_ip2 | 22913 | 32 | 27 | 0 | Belgium | Bovine |
| 299_ip3 | 11406 | 31.7 | 16 | 0 | Belgium | Bovine |
| 302_ip2 | 4570 | 34.9 | 17 | 0 | Brazil | Bovine |
| 302_ip3 | 6911 | 32.7 | 12 | 0 | Brazil | Bovine |
| 302_ip4 | 3325 | 34.1 | 14 | 0 | Brazil | Bovine |
| 333_ip1 | 28772 | 32.8 | 33 | 0 | India | Human |
| 337_ip2 | 11141 | 31 | 13 | 0 | Malaysia | Human |
| 337_ip3 | 17914 | 30.5 | 20 | 0 | Malaysia | Human |
| 394_SAUR_ip2 | 9510 | 31.7 | 16 | 0 | United State | Human |
| 394_SAUR_ip3 | 15765 | 33.9 | 33 | 0 | United State | Human |
| 394_SAUR_ip5 | 24515 | 31.3 | 36 | 0 | United State | Human |
| 511_SAUR_ip2 | 19684 | 33.4 | 24 | 0 | United State | Human |
| 573_SAUR_ip1 | 19684 | 33.4 | 24 | 0 | United State | Human |
| 573_SAUR_ip3 | 5788 | 32.4 | 11 | 0 | United State | Human |
| 922_SAUR_ip1 | 20088 | 31.1 | 24 | 0 | United State | Human |
| 922_SAUR_ip3 | 20002 | 30.5 | 24 | 0 | United State | Human |
| 922_SAUR_ip4 | 15079 | 30.5 | 27 | 0 | United State | Human |
| 922_SAUR_ip5 | 30124 | 32.6 | 36 | 0 | United State | Human |
| 975_SAUR_ip3 | 18544 | 32.4 | 31 | 0 | United State | Human |
| 975_SAUR_ip4 | 26025 | 33.4 | 33 | 0 | United State | Human |
| 975_SAUR_ip5 | 17055 | 33.3 | 35 | 0 | United State | Human |
| 1089_SAUR_ip1 | 18831 | 27.7 | 21 | 0 | United State | Human |
| 1089_SAUR_ip3 | 22369 | 31.1 | 30 | 0 | United State | Human |
| 1089_SAUR_ip5 | 14060 | 32.4 | 23 | 0 | United State | Human |
| 1158_SAUR_ip2 | 19691 | 33.5 | 24 | 0 | United State | Human |
| 1159_SAUR_ip1 | 19325 | 30.4 | 19 | 0 | United State | Human |
| 1159_SAUR_ip2 | 17106 | 31.8 | 24 | 0 | United State | Human |
| 1159_SAUR_ip5 | 27627 | 33.4 | 36 | 0 | United State | Human |
| 1159_SAUR_ip6 | 8990 | 33.7 | 20 | 0 | United State | Human |
| 1159_SAUR_ip7 | 19684 | 33.3 | 23 | 0 | United State | Human |
| 1322_2_SAUR_ip1 | 16562 | 28.6 | 20 | 0 | United State | Human |
| 1364_ip1 | 8505 | 31.5 | 13 | 0 | Brazil | Bovine |
| 1943STDY5573749_ip1 | 19684 | 33.4 | 24 | 0 | United Kingdom | Unknown |
| 1943STDY5573749_ip3 | 16104 | 30.2 | 16 | 0 | United Kingdom | Unknown |
| 1943STDY5698363_ip1 | 23506 | 34.7 | 28 | 0 | United Kingdom | Other |
| 1943STDY5698363_ip2 | 28414 | 33.9 | 46 | 0 | United Kingdom | Other |
| 1943STDY5698363_ip3 | 7187 | 27.5 | 18 | 0 | United Kingdom | Other |
| 2009_60_561_1_ip1 | 6760 | 25.5 | 9 | 0 | Germany | Chicken |
| 2009_60_561_1_ip3 | 16784 | 31.8 | 20 | 0 | Germany | Chicken |
| 2010_60_6511_5_ip1 | 19862 | 33 | 32 | 0 | Netherlands | Bovine |
| 2010_60_6511_5_ip2 | 16412 | 32 | 16 | 0 | Netherlands | Bovine |
| 2010_60_6511_5_ip5 | 12625 | 32.8 | 25 | 0 | Netherlands | Bovine |
| 2010_60_6511_10_ip2 | 16412 | 32 | 16 | 0 | Netherlands | Bovine |
| 2010_60_6511_10_ip3 | 6760 | 25.5 | 9 | 0 | Netherlands | Bovine |
| 2010_60_6511_10_ip5 | 12627 | 33 | 25 | 0 | Netherlands | Bovine |
| 2010_60_6511_39_ip2 | 19508 | 33.5 | 37 | 0 | Germany | Chicken |
| 2010_60_6511_39_ip3 | 6760 | 25.5 | 9 | 0 | Germany | Chicken |
| 2011_60_1490_31_ip1 | 6760 | 25.4 | 9 | 0 | Germany | Chicken |
| 2011_60_1490_31_ip3 | 16412 | 32 | 16 | 0 | Germany | Chicken |
| 2011_60_2078_5_ip1 | 32824 | 32 | 32 | 0 | Netherlands | Bovine |
| 2011_60_2078_5_ip3 | 6760 | 25.5 | 8 | 0 | Netherlands | Bovine |
| 2011_60_2078_5_ip4 | 16727 | 31.4 | 27 | 0 | Netherlands | Bovine |
| 2011_60_2275_1_ip2 | 15250 | 33.1 | 26 | 0 | Germany | Chicken |
| 2011_60_2275_1_ip3 | 16412 | 32 | 16 | 0 | Germany | Chicken |
| 2011_60_2275_1_ip5 | 6760 | 25.4 | 9 | 0 | Germany | Chicken |
| 2011_60_2275_1_ip6 | 10163 | 35.1 | 22 | 0 | Germany | Chicken |
| 2588STDY5627534_ip2 | 7024 | 26.1 | 14 | 0 | United State | Unknown |
| 2588STDY5627534_ip3 | 19685 | 33.3 | 23 | 0 | United State | Unknown |
| 2588STDY5627534_ip4 | 5504 | 27.8 | 7 | 0 | United State | Unknown |
| 2588STDY5748949_ip4 | 7022 | 26.1 | 14 | 0 | United State | Human |
| 2588STDY5748949_ip5 | 19684 | 33.3 | 23 | 0 | United State | Human |
| 2588STDY5748949_ip6 | 26416 | 31.6 | 41 | 0 | United State | Human |
| 2588STDY5748949_ip8 | 32655 | 33.3 | 46 | 0 | United State | Human |
| 2588STDY5748949_ip9 | 28771 | 30.7 | 32 | 0 | United State | Human |
| 3503_ip1 | 17619 | 31 | 24 | 0 | China | Human |
| 3503_ip2 | 34216 | 34.2 | 48 | 0 | China | Human |
| 3503_ip3 | 10039 | 32 | 10 | 0 | China | Human |
| 3503_ip5 | 18830 | 27.7 | 21 | 0 | China | Human |
| 3503VR10_ip1 | 18830 | 27.7 | 21 | 0 | China | Human |
| 3503VR10_ip2 | 17619 | 31 | 24 | 0 | China | Human |
| 3957_ip1 | 26329 | 31.7 | 36 | 0 | India | Human |
| 3957_ip2 | 20854 | 31 | 27 | 0 | India | Human |
| 3957_ip3 | 13172 | 32.8 | 16 | 0 | India | Human |
| 4126_2_ip1 | 10039 | 32 | 9 | 0 | China | Human |
| 4126_2_ip2 | 18830 | 27.7 | 21 | 0 | China | Human |
| 4126_2_ip4 | 34217 | 34.2 | 47 | 0 | China | Human |
| 4126_2_ip5 | 19892 | 30.8 | 25 | 0 | China | Human |
| 6850_ip1 | 29048 | 32.9 | 29 | 0 | Germany | Human |
| 6850_ip2 | 13398 | 30.5 | 13 | 0 | Germany | Human |
| 08134_6_ip2 | 16412 | 32 | 16 | 0 | Belgium | Other |
| 08134_6_ip3 | 6760 | 25.5 | 9 | 0 | Belgium | Other |
| 08139_6_ip1 | 4937 | 25.8 | 7 | 0 | Netherlands | Other |
| 08139_6_ip2 | 16412 | 32 | 16 | 0 | Netherlands | Other |
| 08142_8_ip1 | 16412 | 32 | 16 | 0 | Netherlands | Other |
| 08142_8_ip2 | 20349 | 33.1 | 33 | 0 | Netherlands | Other |
| 08143_5_ip1 | 16412 | 32 | 16 | 0 | Netherlands | Other |
| 08143_5_ip2 | 20349 | 33.1 | 33 | 0 | Netherlands | Other |
| 10388_ip1 | 6104 | 28.6 | 7 | 0 | Switzerland | Unknown |
| 10388_ip2 | 18826 | 27.7 | 21 | 0 | Switzerland | Unknown |
| 10497_ip1 | 6104 | 28.6 | 7 | 0 | Switzerland | Unknown |
| 10497_ip2 | 18826 | 27.7 | 21 | 0 | Switzerland | Unknown |
| 15532_ip1 | 6104 | 28.6 | 7 | 0 | Switzerland | Unknown |
| 15532_ip2 | 18824 | 27.7 | 21 | 0 | Switzerland | Unknown |
| 16035_ip1 | 6047 | 28.4 | 7 | 0 | Switzerland | Unknown |
| 16035_ip2 | 18826 | 27.7 | 21 | 0 | Switzerland | Unknown |
| 16125_ip1 | 6104 | 28.6 | 7 | 0 | Switzerland | Unknown |
| 16125_ip2 | 18826 | 27.7 | 21 | 0 | Switzerland | Unknown |
| 18341_ip1 | 6104 | 28.6 | 7 | 0 | Switzerland | Unknown |
| 18341_ip2 | 18826 | 27.7 | 21 | 0 | Switzerland | Unknown |
| 18583_ip1 | 6104 | 28.6 | 7 | 0 | Switzerland | Unknown |
| 18583_ip2 | 18826 | 27.7 | 21 | 0 | Switzerland | Unknown |
| 18754_2_ip1 | 28585 | 33.2 | 32 | 0 | Italy | Swine |
| 18754_2_ip2 | 16538 | 31.8 | 16 | 0 | Italy | Swine |
| 18754_2_ip4 | 28515 | 31.6 | 40 | 0 | Italy | Swine |
| 18754_2_ip5 | 26990 | 30.6 | 30 | 0 | Italy | Swine |
| 18754_2_ip6 | 15975 | 31.3 | 21 | 0 | Italy | Swine |
| 71193_ip1 | 8325 | 26.5 | 12 | 0 | United State | Human |
| 91751_ip2 | 25610 | 32.1 | 25 | 0 | Switzerland | Human |
| 91751_ip3 | 15135 | 32.1 | 30 | 0 | Switzerland | Human |
| 91751_ip4 | 11143 | 32.9 | 15 | 0 | Switzerland | Human |
| 91751_ip5 | 24749 | 32 | 33 | 0 | Switzerland | Human |
| 91751_ip6 | 11919 | 34.8 | 19 | 1 | Switzerland | Human |
| 91751_ip7 | 9755 | 30.9 | 15 | 0 | Switzerland | Human |
| 91751_ip9 | 13874 | 32.4 | 22 | 0 | Switzerland | Human |
| 122051_ip1 | 22643 | 32.9 | 24 | 0 | Switzerland | Human |
| 122051_ip3 | 16510 | 28.7 | 21 | 0 | Switzerland | Human |
| 1110700562_ip1 | 6760 | 25.5 | 9 | 0 | Netherlands | Human |
| 1110700610_ip2 | 6760 | 25.5 | 9 | 0 | Netherlands | Human |
| A5_ip1 | 19684 | 33.3 | 24 | 0 | Australia | Human |
| A5_ip2 | 23839 | 34.8 | 27 | 0 | Australia | Human |
| A5_ip4 | 11122 | 33.1 | 16 | 0 | Australia | Human |
| A5_ip5 | 11445 | 34.6 | 28 | 0 | Australia | Human |
| A53_ip1 | 18829 | 27.7 | 21 | 0 | Brazil | Other |
| A69_ip2 | 21279 | 30.1 | 33 | 0 | China | Swine |
| A69_ip3 | 16757 | 31.7 | 22 | 0 | China | Swine |
| A71_ip1 | 16755 | 31.7 | 20 | 0 | China | Swine |
| A71_ip2 | 21299 | 30.1 | 34 | 0 | China | Swine |
| A71_ip3 | 9747 | 35 | 25 | 0 | China | Swine |
| A187_ip1 | 9086 | 26.3 | 10 | 0 | China | Swine |
| A900624_ip1 | 19584 | 31.1 | 26 | 0 | France | Human |
| A900624_ip2 | 16806 | 29.8 | 31 | 0 | France | Human |
| aless_ip2 | 28585 | 33.2 | 33 | 0 | Brazil | Human |
| aless_ip3 | 23247 | 29.9 | 39 | 0 | Brazil | Human |
| APS210_ip1 | 10453 | 32.1 | 20 | 0 | Australia | Human |
| APS210_ip2 | 20399 | 32.4 | 37 | 0 | Australia | Human |
| APS210_ip3 | 17631 | 34.6 | 27 | 0 | Australia | Human |
| APS210_ip4 | 7685 | 32.2 | 15 | 0 | Australia | Human |
| APS210_ip6 | 8072 | 33.5 | 18 | 0 | Australia | Human |
| APS210_ip7 | 26081 | 30.9 | 31 | 0 | Australia | Human |
| APS210_ip8 | 15527 | 34.1 | 22 | 0 | Australia | Human |
| APS211_ip1 | 10504 | 32.1 | 20 | 0 | Australia | Human |
| APS211_ip2 | 27643 | 31.2 | 43 | 0 | Australia | Human |
| APS211_ip3 | 7685 | 32.2 | 15 | 0 | Australia | Human |
| APS211_ip4 | 18019 | 34.7 | 26 | 0 | Australia | Human |
| APS211_ip5 | 17243 | 33.7 | 27 | 0 | Australia | Human |
| APS211_ip6 | 14598 | 34 | 21 | 0 | Australia | Human |
| ARI29_ip1 | 18139 | 33.1 | 30 | 0 | United Kingdom | Human |
| ARI29_ip2 | 43246 | 31.8 | 51 | 0 | United Kingdom | Human |
| ARI29_ip3 | 10055 | 28.3 | 15 | 0 | United Kingdom | Human |
| ARI29_ip4 | 17240 | 30.5 | 18 | 0 | United Kingdom | Human |
| ARI29_ip5 | 6605 | 31 | 7 | 0 | United Kingdom | Human |
| ARI31_ip1 | 7663 | 26.7 | 9 | 0 | United Kingdom | Human |
| ARI31_ip2 | 39534 | 32.3 | 59 | 0 | United Kingdom | Human |
| ARI31_ip5 | 15357 | 31.7 | 16 | 13 | United Kingdom | Human |
| ARI31_ip6 | 20874 | 32.3 | 24 | 18 | United Kingdom | Human |
| ATCC_25923_ip3 | 17917 | 29.1 | 21 | 0 | United State | Human |
| ATCC_25923_ip4 | 17678 | 32 | 24 | 0 | United State | Human |
| ATCC_29213_ip2 | 11014 | 33.7 | 23 | 0 | Unknown | Unknown |
| ATCC_29213_ip3 | 7462 | 30.1 | 9 | 0 | Unknown | Unknown |
| ATCC_29213_ip4 | 16896 | 33.3 | 34 | 0 | Unknown | Unknown |
| AUS0325_ip2 | 8191 | 25.6 | 15 | 0 | Australia | Human |
| BA01611_ip1 | 16949 | 31.6 | 20 | 0 | China | Bovine |
| BA01611_ip3 | 7223 | 28.6 | 8 | 0 | China | Bovine |
| BA01611_ip4 | 25633 | 30.1 | 39 | 0 | China | Bovine |
| BA01611_ip5 | 12878 | 27.4 | 13 | 0 | China | Bovine |
| BSAR77_ip3 | 7663 | 26.7 | 9 | 0 | United Kingdom | Human |
| BSAR77_ip4 | 6554 | 30.4 | 10 | 0 | United Kingdom | Human |
| BSAR111_2_ip3 | 18750 | 30.3 | 18 | 0 | Denmark | sheep |
| BSAR176_2_ip1 | 29595 | 30.9 | 32 | 0 | United Kingdom | canine |
| BSAR176_2_ip2 | 43098 | 32.5 | 57 | 0 | United Kingdom | canine |
| BSAR176_2_ip3 | 7663 | 26.7 | 9 | 0 | United Kingdom | canine |
| BSAR193_2_ip2 | 29595 | 30.9 | 32 | 0 | United Kingdom | canine |
| BSAR193_2_ip4 | 7663 | 26.7 | 9 | 0 | United Kingdom | canine |
| BSAR202_ip2 | 29595 | 30.9 | 32 | 0 | United Kingdom | canine |
| BSAR202_ip4 | 9342 | 26.5 | 11 | 0 | United Kingdom | canine |
| BSAR205_ip2 | 29595 | 30.9 | 32 | 0 | United Kingdom | Other |
| BSAR205_ip3 | 7663 | 26.7 | 9 | 0 | United Kingdom | Other |
| BSAR208_ip1 | 28843 | 32.6 | 40 | 0 | United Kingdom | Human |
| BSAR208_ip2 | 29595 | 30.9 | 32 | 0 | United Kingdom | Human |
| BSAR208_ip5 | 21640 | 33 | 32 | 0 | United Kingdom | Human |
| BSAR486_ip2 | 20551 | 31.1 | 21 | 0 | United Kingdom | Human |
| BSAR486_ip4 | 8197 | 29.1 | 10 | 0 | United Kingdom | Human |
| BSAR729_ip1 | 36299 | 32.2 | 51 | 0 | United Kingdom | Human |
| BSAR729_ip2 | 17240 | 30.5 | 18 | 0 | United Kingdom | Human |
| BSAR729_ip4 | 16321 | 30.2 | 18 | 0 | United Kingdom | Human |
| BSAR729_ip5 | 9341 | 26.5 | 11 | 0 | United Kingdom | Human |
| BSAR729_ip6 | 10918 | 34 | 23 | 0 | United Kingdom | Human |
| BSAR734_ip1 | 26854 | 34.3 | 31 | 0 | United Kingdom | Human |
| BSAR734_ip2 | 14837 | 31.7 | 20 | 1 | United Kingdom | Human |
| BSAR734_ip3 | 26717 | 30.5 | 32 | 0 | United Kingdom | Human |
| BSAR734_ip4 | 30856 | 33 | 43 | 0 | United Kingdom | Human |
| BSAR739_ip3 | 9346 | 28.7 | 10 | 6 | United Kingdom | Human |
| BSAR739_ip4 | 7901 | 27.8 | 10 | 0 | United Kingdom | Human |
| BSAR748_ip2 | 27129 | 31.3 | 28 | 0 | United Kingdom | Human |
| BSAR748_ip3 | 7291 | 26.5 | 10 | 0 | United Kingdom | Human |
| BSAR748_ip4 | 14071 | 33.5 | 29 | 0 | United Kingdom | Human |
| BSAR858_ip2 | 9342 | 26.5 | 11 | 0 | United Kingdom | canine |
| BSAR865_ip2 | 17239 | 30.5 | 19 | 0 | United Kingdom | Other |
| BSAR865_ip4 | 26540 | 33.7 | 30 | 0 | United Kingdom | Other |
| BSAR865_ip5 | 19248 | 32.8 | 32 | 0 | United Kingdom | Other |
| BSAR865_ip6 | 10273 | 27.8 | 12 | 0 | United Kingdom | Other |
| BSAR865_ip7 | 7164 | 41.1 | 10 | 8 | United Kingdom | Other |
| BU_G0201_t8_ip2 | 8196 | 25.6 | 16 | 0 | Ghana | Human |
| BU_G0201_t8_ip4 | 37778 | 33.2 | 55 | 0 | Ghana | Human |
| BU_G0201_t8_ip5 | 11445 | 31.8 | 14 | 0 | Ghana | Human |
| BU_G0201_t8_ip6 | 19652 | 32.8 | 17 | 0 | Ghana | Human |
| BU_W12_t13_ip1 | 15811 | 29.9 | 29 | 0 | Ghana | Human |
| BU_W12_t13_ip2 | 18929 | 31.8 | 23 | 0 | Ghana | Human |
| BU_W12_t13_ip4 | 8833 | 35 | 20 | 0 | Ghana | Human |
| BU_W12_t13_ip5 | 13952 | 33.4 | 31 | 0 | Ghana | Human |
| BU_W22_t4_ip1 | 17128 | 31.2 | 22 | 0 | Ghana | Human |
| BU_W22_t4_ip2 | 6810 | 29.8 | 8 | 0 | Ghana | Human |
| BU_W22_t4_ip3 | 18297 | 31.2 | 24 | 0 | Ghana | Human |
| BU_W22_t4_ip4 | 28950 | 34.3 | 41 | 0 | Ghana | Human |
| BU_W22_t4_ip5 | 18831 | 27.7 | 21 | 0 | Ghana | Human |
| C9_ip1 | 9510 | 31.7 | 16 | 0 | Australia | Human |
| C1655_ip2 | 16412 | 32 | 16 | 0 | Spain | Swine |
| C1655_ip3 | 6760 | 25.5 | 9 | 0 | Spain | Swine |
| CA12_ip1 | 19684 | 33.3 | 23 | 0 | United State | Human |
| CA12_ip2 | 7022 | 26.1 | 14 | 0 | United State | Human |
| CA_347_ip1 | 22722 | 34.1 | 23 | 0 | United State | Human |
| CA_347_ip2 | 7444 | 30.8 | 14 | 0 | United State | Human |
| CA_347_ip3 | 16830 | 29.8 | 32 | 0 | United State | Human |
| CA_347_ip5 | 6092 | 31 | 10 | 0 | United State | Human |
| CC022_ip1 | 18415 | 31.5 | 24 | 0 | Brazil | Human |
| CC022_ip2 | 17917 | 29.1 | 21 | 0 | Brazil | Human |
| CC022_ip4 | 24230 | 33.1 | 37 | 0 | Brazil | Human |
| CC169_ip1 | 18415 | 31.5 | 24 | 0 | Brazil | Human |
| CC169_ip2 | 17917 | 29.1 | 21 | 0 | Brazil | Human |
| CC169_ip3 | 25249 | 33.2 | 38 | 0 | Brazil | Human |
| Chi_4_ip1 | 6760 | 25.5 | 9 | 0 | Germany | Chicken |
| Chi_4_ip3 | 16412 | 32 | 16 | 0 | Germany | Chicken |
| Chi_8_ip2 | 26742 | 33.6 | 46 | 0 | Germany | Chicken |
| Chi_8_ip3 | 6760 | 25.5 | 9 | 0 | Germany | Chicken |
| Chi_8_ip5 | 14841 | 33.5 | 30 | 0 | Germany | Chicken |
| Chi_10_ip1 | 6760 | 25.5 | 9 | 0 | Austria | Chicken |
| Chi_10_ip3 | 16784 | 31.8 | 20 | 0 | Austria | Chicken |
| CIG1242_ip2 | 22718 | 34.1 | 24 | 0 | United State | Human |
| CIG1242_ip5 | 12199 | 27.3 | 16 | 0 | United State | Human |
| CIG1242_ip7 | 26093 | 31.3 | 35 | 0 | United State | Human |
| CIG1524_ip1 | 16632 | 29.8 | 32 | 0 | United State | Human |
| CIG1524_ip2 | 8574 | 26.9 | 12 | 0 | United State | Human |
| CIG1835_ip1 | 17731 | 29.8 | 20 | 0 | United State | Human |
| CN1_ip1 | 19568 | 29 | 24 | 0 | Korea | Unknown |
| CN79_ip1 | 9803 | 32.3 | 10 | 0 | China | Human |
| CN79_ip4 | 21696 | 31.9 | 26 | 0 | China | Human |
| CN79_ip5 | 4824 | 32.9 | 7 | 0 | China | Human |
| COL_ip2 | 19684 | 33.3 | 23 | 0 | United Kingdom | Other |
| COL_ip3 | 7021 | 26.1 | 14 | 0 | United Kingdom | Other |
| CUHK_BJ2002_ip2 | 9803 | 32.3 | 10 | 0 | China | Human |
| CUHK_HK188_ip1 | 7795 | 26.5 | 15 | 0 | China | Human |
| CUHK_HK188_ip2 | 29728 | 32.9 | 48 | 0 | China | Human |
| CUHK_HK188_ip4 | 26287 | 30.5 | 38 | 0 | China | Human |
| CUHK_HK188_ip5 | 24735 | 33.3 | 28 | 0 | China | Human |
| CUHK_HK1997_ip1 | 19684 | 33.3 | 23 | 0 | China | Human |
| CUHK_HK1997_ip10 | 12845 | 33.7 | 20 | 0 | China | Human |
| CUHK_HK1997_ip11 | 9498 | 35.1 | 24 | 0 | China | Human |
| CUHK_HK1997_ip2 | 29617 | 31 | 44 | 0 | China | Human |
| CUHK_HK1997_ip3 | 21664 | 30.7 | 26 | 0 | China | Human |
| CUHK_HK1997_ip4 | 48684 | 31.3 | 36 | 0 | China | Human |
| CUHK_HK1997_ip6 | 9805 | 32.3 | 11 | 0 | China | Human |
| CUHK_HK1997_ip7 | 28217 | 28.9 | 54 | 0 | China | Human |
| CUHK_HK1997_ip8 | 11131 | 32.9 | 16 | 0 | China | Human |
| CUHK_HK1997_ip9 | 18094 | 34.1 | 28 | 0 | China | Human |
| CUHK_HK2007_ip2 | 19684 | 33.3 | 23 | 0 | China | Human |
| CUHK_HK2007_ip3 | 8108 | 31.3 | 8 | 0 | China | Human |
| CUHK_HK2007_ip4 | 69038 | 31.3 | 57 | 0 | China | Human |
| CUHK_HK2007_ip5 | 29616 | 31 | 43 | 0 | China | Human |
| CUHK_HK2007_ip6 | 22303 | 30.5 | 30 | 0 | China | Human |
| CUHK_HK2007_ip7 | 27597 | 33.3 | 34 | 0 | China | Human |
| CUHK_HK2007_ip8 | 23059 | 29.1 | 42 | 0 | China | Human |
| D71_ip1 | 19684 | 33.3 | 23 | 0 | Germany | Human |
| D71_ip2 | 71947 | 32.3 | 56 | 0 | Germany | Human |
| D71_ip3 | 40928 | 31.7 | 54 | 0 | Germany | Human |
| D71_ip4 | 39891 | 30.6 | 68 | 0 | Germany | Human |
| D71_ip6 | 13193 | 35.3 | 24 | 0 | Germany | Human |
| D71_ip7 | 27088 | 30.3 | 42 | 0 | Germany | Human |
| D71_ip8 | 14487 | 30.8 | 30 | 0 | Germany | Human |
| DAR22_ip2 | 6113 | 28.6 | 7 | 0 | Belgium | Human |
| DAR22_ip3 | 15536 | 27.2 | 18 | 0 | Belgium | Human |
| DAR948_ip1 | 6113 | 28.6 | 7 | 0 | United State | Human |
| DAR948_ip3 | 22811 | 28.6 | 24 | 0 | United State | Human |
| DAR948_ip5 | 18339 | 31.4 | 20 | 0 | United State | Human |
| DAR1090_ip1 | 22810 | 28.7 | 23 | 0 | United State | Human |
| DAR1090_ip2 | 47582 | 32.6 | 68 | 0 | United State | Human |
| DAR3179_ip2 | 31684 | 31.5 | 41 | 0 | Argentina | Human |
| DAR3179_ip3 | 19781 | 31.3 | 27 | 0 | Argentina | Human |
| DAR3179_ip4 | 22809 | 28.7 | 24 | 0 | Argentina | Human |
| DAR3179_ip5 | 12500 | 32 | 15 | 0 | Argentina | Human |
| DAR3179_ip6 | 10020 | 33.2 | 19 | 0 | Argentina | Human |
| DAR3236_ip1 | 31817 | 32.7 | 37 | 0 | Argentina | Human |
| DAR3236_ip3 | 7445 | 29.9 | 8 | 0 | Argentina | Human |
| DAR3236_ip4 | 18829 | 27.7 | 22 | 0 | Argentina | Human |
| 07_02088_ip3 | 12403 | 32.4 | 14 | 0 | Germany | Human |
| 07_02088_ip4 | 10457 | 27.4 | 13 | 3 | Germany | Human |
| DAR3534_ip1 | 6057 | 28.3 | 7 | 0 | United State | Human |
| DAR3534_ip2 | 22809 | 28.7 | 24 | 0 | United State | Human |
| DAR3581_ip1 | 6113 | 28.6 | 7 | 0 | United State | Human |
| DAR3581_ip3 | 20480 | 27.9 | 22 | 0 | United State | Human |
| DAR4145_ip2 | 21984 | 31.7 | 26 | 0 | India | Human |
| DAR4145_ip3 | 6404 | 28 | 7 | 0 | India | Human |
| DAR4145_ip4 | 29683 | 32.1 | 32 | 0 | India | Human |
| DAR5798_ip1 | 14542 | 31.3 | 21 | 0 | Colombia | Human |
| DAR5798_ip2 | 18831 | 27.7 | 21 | 0 | Colombia | Human |
| DAR5798_ip3 | 9408 | 34.6 | 23 | 0 | Colombia | Human |
| DAR5843_ip3 | 21148 | 28.9 | 24 | 0 | Peru | Human |
| DAR5854_ip1 | 22193 | 28.7 | 25 | 0 | Ecuador | Human |
| DAR5854_ip2 | 27315 | 33 | 40 | 0 | Ecuador | Human |
| DAR5854_ip4 | 12366 | 32.7 | 25 | 0 | Ecuador | Human |
| DAR5867_ip1 | 12199 | 27.3 | 16 | 0 | Argentina | Human |
| DAR5867_ip3 | 29347 | 34.3 | 42 | 0 | Argentina | Human |
| DAR5867_ip4 | 11367 | 31.4 | 17 | 0 | Argentina | Human |
| DAR5874_ip1 | 7445 | 29.9 | 8 | 0 | Colombia | Human |
| DAR5874_ip2 | 18829 | 27.7 | 22 | 0 | Colombia | Human |
| DAR5877_ip1 | 18703 | 29.2 | 19 | 0 | Peru | Human |
| DAR5877_ip3 | 16707 | 34.4 | 21 | 0 | Peru | Human |
| DAR5877_ip4 | 8618 | 30.4 | 10 | 0 | Peru | Human |
| DAR5877_ip5 | 8705 | 33.6 | 18 | 0 | Peru | Human |
| DAR5889_ip1 | 31817 | 32.7 | 37 | 0 | Peru | Human |
| DAR5889_ip4 | 21492 | 28.7 | 24 | 0 | Peru | Human |
| DAR5890_ip1 | 31817 | 32.7 | 37 | 0 | Venezuela | Human |
| DAR5890_ip2 | 7445 | 29.9 | 8 | 0 | Venezuela | Human |
| DAR5890_ip3 | 22808 | 28.7 | 25 | 0 | Venezuela | Human |
| DAR5898_ip1 | 31817 | 32.7 | 37 | 0 | Venezuela | Human |
| DAR5898_ip2 | 7445 | 29.9 | 8 | 0 | Venezuela | Human |
| DAR5898_ip3 | 22233 | 32.3 | 35 | 0 | Venezuela | Human |
| DAR5898_ip4 | 18829 | 27.7 | 22 | 0 | Venezuela | Human |
| DEU16_ip1 | 15805 | 31.8 | 21 | 0 | Turkey | Human |
| DEU16_ip2 | 23248 | 33.3 | 26 | 0 | Turkey | Human |
| DEU16_ip3 | 9803 | 32.3 | 10 | 0 | Turkey | Human |
| DEU16_ip4 | 21529 | 32.4 | 26 | 0 | Turkey | Human |
| DEU16_ip5 | 25542 | 34 | 33 | 0 | Turkey | Human |
| DICM09_01587_13HST_ip1 | 6760 | 25.5 | 9 | 0 | Spain | Sheep |
| DICM09_01587_13HST_ip3 | 16412 | 32 | 16 | 0 | Spain | Sheep |
| DSM_799_ip1 | 26987 | 30.6 | 29 | 0 | United State | Human |
| DSM_799_ip3 | 23247 | 29.9 | 39 | 0 | United State | Human |
| DSM_799_ip4 | 28585 | 33.2 | 33 | 0 | United State | Human |
| ECT_R_2_ip1 | 6169 | 28.7 | 8 | 0 | Sweden | Human |
| ECT_R_2_ip2 | 22809 | 28.6 | 24 | 0 | Sweden | Human |
| ED98_ip2 | 6169 | 28.8 | 7 | 0 | Ireland | Chicken |
| ES26_ip1 | 21090 | 30.6 | 30 | 0 | Spain | Human |
| ES26_ip2 | 9803 | 32.3 | 10 | 0 | Spain | Human |
| ES26_ip3 | 23016 | 31.3 | 22 | 0 | Spain | Human |
| ES26_ip5 | 26111 | 33.6 | 30 | 0 | Spain | Human |
| ES26_ip6 | 16755 | 34 | 21 | 0 | Spain | Human |
| ES26_ip8 | 26408 | 30.8 | 24 | 0 | Spain | Human |
| ES26_ip9 | 8854 | 31.6 | 17 | 0 | Spain | Human |
| F19490_ip1 | 13030 | 32.2 | 21 | 0 | United State | Unknown |
| F19490_ip2 | 26248 | 31.4 | 41 | 0 | United State | Unknown |
| F26051_ip1 | 9484 | 29.2 | 11 | 0 | United State | Human |
| F48950_ip1 | 19685 | 33.3 | 23 | 0 | United State | Human |
| F48950_ip3 | 7022 | 26.1 | 14 | 0 | United State | Human |
| FDA209P_ip1 | 28585 | 33.2 | 33 | 0 | Unknown | Unknown |
| FDA209P_ip2 | 28608 | 30.1 | 44 | 0 | Unknown | Unknown |
| FDA209P_ip3 | 33113 | 29.3 | 39 | 0 | Unknown | Unknown |
| FKWK_ip2 | 26619 | 31.6 | 37 | 0 | United State | Human |
| FKWK_ip3 | 22721 | 33.3 | 26 | 0 | United State | Human |
| FKWK_ip4 | 22624 | 30.9 | 28 | 0 | United State | Human |
| FKWK_ip7 | 7141 | 32.8 | 16 | 0 | United State | Human |
| FP_N239_ip2 | 6760 | 25.5 | 9 | 0 | Netherlands | Bovine |
| FRI137_ip2 | 21761 | 33.7 | 30 | 0 | United State | Human |
| FRI137_ip3 | 14448 | 31.1 | 16 | 0 | United State | Human |
| FRI137_ip4 | 23984 | 31.7 | 33 | 0 | United State | Human |
| FRI137_ip5 | 10744 | 29.9 | 18 | 0 | United State | Human |
| FVRH6002_ip1 | 29617 | 31 | 44 | 0 | United State | Human |
| FVRH6002_ip3 | 19684 | 33.3 | 23 | 0 | United State | Human |
| FVRH6002_ip4 | 9804 | 32.5 | 10 | 0 | United State | Human |
| FVRH6002_ip5 | 27625 | 33.3 | 34 | 0 | United State | Human |
| FVRH6002_ip6 | 69413 | 31.2 | 59 | 0 | United State | Human |
| FVRH6002_ip7 | 31121 | 29 | 58 | 0 | United State | Human |
| FY20_ip1 | 6760 | 25.4 | 10 | 0 | China | Human |
| FY22_ip1 | 6760 | 25.5 | 9 | 0 | China | Human |
| GD5_ip1 | 6760 | 25.5 | 9 | 0 | China | Human |
| GD705_ip1 | 6760 | 25.5 | 9 | 0 | China | Human |
| GD1539_ip2 | 6755 | 25.5 | 9 | 0 | China | Human |
| GD1677_ip2 | 6760 | 25.5 | 9 | 0 | China | Human |
| GKP136_8_ip1 | 12190 | 26.7 | 12 | 0 | United Kingdom | Bovine |
| GKP136_11_ip2 | 15685 | 33.7 | 33 | 0 | United Kingdom | Bovine |
| GKP136_11_ip5 | 10108 | 34.2 | 21 | 0 | United Kingdom | Bovine |
| GKP136_12_ip1 | 10868 | 31.9 | 14 | 0 | United Kingdom | Bovine |
| GKP136_12_ip2 | 22932 | 32.1 | 26 | 0 | United Kingdom | Bovine |
| GKP136_12_ip3 | 14477 | 28.2 | 15 | 0 | United Kingdom | Bovine |
| GKP136_12_ip4 | 25922 | 36.9 | 31 | 0 | United Kingdom | Bovine |
| GKP136_19_ip1 | 16627 | 31.9 | 17 | 0 | United Kingdom | Bovine |
| GKP136_19_ip2 | 28583 | 33.2 | 32 | 0 | United Kingdom | Bovine |
| GKP136_19_ip3 | 28722 | 31.6 | 40 | 0 | United Kingdom | Bovine |
| GKP136_19_ip4 | 16186 | 33.4 | 22 | 0 | United Kingdom | Bovine |
| GKP136_19_ip6 | 18700 | 30.7 | 25 | 0 | United Kingdom | Bovine |
| GKP136_21_ip2 | 13057 | 28.9 | 13 | 0 | United Kingdom | Bovine |
| GKP136_36_ip2 | 6487 | 34 | 9 | 0 | United Kingdom | Bovine |
| GKP136_36_ip3 | 6126 | 28.6 | 7 | 0 | United Kingdom | Bovine |
| GKP136_64_ip1 | 5622 | 25.5 | 7 | 0 | United Kingdom | Bovine |
| GKP136_67_ip2 | 19907 | 34.1 | 23 | 0 | United Kingdom | Bovine |
| GKP136_68_ip2 | 9236 | 31.8 | 9 | 0 | United Kingdom | Bovine |
| GKP136_68_ip3 | 13820 | 27.5 | 15 | 0 | United Kingdom | Bovine |
| GKP136_68_ip4 | 13006 | 34.4 | 28 | 0 | United Kingdom | Bovine |
| GKP136_74_ip1 | 28585 | 33.2 | 32 | 0 | United Kingdom | Bovine |
| GKP136_74_ip2 | 21972 | 30 | 36 | 0 | United Kingdom | Bovine |
| GKP136_74_ip3 | 18701 | 30.7 | 25 | 0 | United Kingdom | Bovine |
| GKP138_17_ip1 | 9191 | 33.3 | 10 | 0 | United Kingdom | Bovine |
| GKP138_17_ip2 | 12190 | 26.7 | 12 | 0 | United Kingdom | Bovine |
| GKP138_17_ip3 | 22932 | 32.1 | 26 | 0 | United Kingdom | Bovine |
| GKP138_17_ip6 | 16009 | 37.2 | 19 | 0 | United Kingdom | Bovine |
| GKP138_17_ip7 | 10133 | 34.6 | 25 | 0 | United Kingdom | Bovine |
| GKP138_21_ip1 | 9510 | 31.7 | 16 | 0 | United Kingdom | Bovine |
| GKP138_21_ip2 | 9625 | 26.9 | 21 | 0 | United Kingdom | Bovine |
| GKP138_71_ip1 | 16693 | 32.7 | 23 | 0 | United Kingdom | Bovine |
| GKP138_71_ip3 | 22350 | 32.4 | 33 | 0 | United Kingdom | Bovine |
| GKP138_71_ip4 | 9236 | 31.8 | 9 | 0 | United Kingdom | Bovine |
| GKP138_71_ip6 | 22534 | 29.4 | 21 | 0 | United Kingdom | Bovine |
| H_EMRSA_15_ip1 | 17240 | 30.5 | 18 | 0 | Belgium | Human |
| H_EMRSA_15_ip2 | 8722 | 27.7 | 11 | 0 | Belgium | Human |
| H_EMRSA_15_ip4 | 7406 | 30.1 | 12 | 0 | Belgium | Human |
| H_EMRSA_15_ip5 | 9342 | 26.5 | 11 | 0 | Belgium | Human |
| H24_ip1 | 7030 | 26.1 | 14 | 0 | Egypt | Human |
| H24_ip3 | 9803 | 32.4 | 10 | 0 | Egypt | Human |
| H24_ip4 | 16529 | 34.1 | 20 | 0 | Egypt | Human |
| H24_ip6 | 23531 | 31.3 | 30 | 0 | Egypt | Human |
| H24_ip7 | 11572 | 36.2 | 23 | 0 | Egypt | Human |
| H202_ip1 | 52338 | 32.5 | 41 | 0 | Thailand | Human |
| H202_ip10 | 20009 | 31.4 | 28 | 0 | Thailand | Human |
| H202_ip2 | 19448 | 30.9 | 31 | 0 | Thailand | Human |
| H202_ip4 | 18215 | 32.5 | 27 | 0 | Thailand | Human |
| H202_ip5 | 9300 | 27.5 | 21 | 0 | Thailand | Human |
| H202_ip6 | 63389 | 33.2 | 73 | 0 | Thailand | Human |
| H202_ip7 | 29355 | 30.6 | 47 | 0 | Thailand | Human |
| H202_ip8 | 9329 | 29.5 | 18 | 0 | Thailand | Human |
| H202_ip9 | 8477 | 33.2 | 13 | 0 | Thailand | Human |
| H211_ip1 | 24294 | 32 | 34 | 0 | Denmark | Human |
| H211_ip10 | 14711 | 33.6 | 31 | 0 | Denmark | Human |
| H211_ip11 | 35816 | 30.7 | 40 | 0 | Denmark | Human |
| H211_ip12 | 30649 | 33.2 | 45 | 0 | Denmark | Human |
| H211_ip13 | 16572 | 32.6 | 24 | 0 | Denmark | Human |
| H211_ip14 | 19026 | 32 | 36 | 0 | Denmark | Human |
| H211_ip2 | 67875 | 31.5 | 56 | 0 | Denmark | Human |
| H211_ip3 | 25771 | 31.9 | 28 | 0 | Denmark | Human |
| H211_ip4 | 50110 | 31.7 | 65 | 0 | Denmark | Human |
| H211_ip5 | 9759 | 32.4 | 10 | 0 | Denmark | Human |
| H211_ip7 | 17841 | 34.3 | 19 | 0 | Denmark | Human |
| H211_ip8 | 15101 | 28.8 | 41 | 0 | Denmark | Human |
| H211_ip9 | 18038 | 32.2 | 17 | 0 | Denmark | Human |
| H216_ip1 | 60999 | 31.6 | 46 | 0 | Denmark | Human |
| H216_ip10 | 26032 | 31.7 | 25 | 0 | Denmark | Human |
| H216_ip11 | 24416 | 30.4 | 35 | 0 | Denmark | Human |
| H216_ip12 | 42829 | 33.1 | 61 | 0 | Denmark | Human |
| H216_ip13 | 21539 | 31.3 | 42 | 0 | Denmark | Human |
| H216_ip2 | 9804 | 32.3 | 10 | 0 | Denmark | Human |
| H216_ip3 | 19684 | 33.3 | 23 | 0 | Denmark | Human |
| H216_ip4 | 38260 | 31.3 | 55 | 0 | Denmark | Human |
| H216_ip5 | 23412 | 30.8 | 26 | 0 | Denmark | Human |
| H216_ip6 | 7643 | 31.5 | 13 | 0 | Denmark | Human |
| H216_ip8 | 22384 | 34 | 23 | 0 | Denmark | Human |
| H482_ip1 | 8282 | 26.5 | 17 | 0 | Romania | Human |
| H482_ip2 | 19684 | 33.3 | 23 | 0 | Romania | Human |
| H482_ip3 | 9803 | 32.3 | 10 | 0 | Romania | Human |
| H482_ip4 | 29863 | 33.5 | 39 | 0 | Romania | Human |
| H482_ip5 | 38049 | 32.4 | 44 | 0 | Romania | Human |
| H482_ip6 | 46288 | 30.9 | 63 | 0 | Romania | Human |
| H482_ip7 | 32722 | 31.9 | 45 | 0 | Romania | Human |
| H914_91_ip1 | 36879 | 31.9 | 52 | 0 | United Kingdom | Human |
| H914_91_ip2 | 11096 | 28.6 | 11 | 1 | United Kingdom | Human |
| H914_91_ip6 | 8712 | 33.1 | 10 | 0 | United Kingdom | Human |
| HI010_ip1 | 19684 | 33.3 | 23 | 0 | United State | Human |
| HI010_ip2 | 20640 | 30.8 | 24 | 0 | United State | Human |
| HI010_ip3 | 7022 | 26.1 | 14 | 0 | United State | Human |
| HI022_ip1 | 19684 | 33.3 | 23 | 0 | United State | Human |
| HI022_ip2 | 20640 | 30.8 | 24 | 0 | United State | Human |
| HI022_ip3 | 7022 | 26.1 | 14 | 0 | United State | Human |
| HI111_ip1 | 19684 | 33.3 | 23 | 0 | United State | Human |
| HI111_ip2 | 20639 | 30.8 | 24 | 0 | United State | Human |
| HI111_ip3 | 7022 | 26.1 | 14 | 0 | United State | Human |
| HIF003_B2N_C_ip1 | 7022 | 26.1 | 14 | 0 | United State | Human |
| HIF003_B2N_C_ip3 | 19683 | 33.3 | 23 | 0 | United State | Human |
| HOU1445_VS_ip1 | 15010 | 34.4 | 22 | 0 | Brazil | Human |
| HOU1445_VS_ip2 | 17539 | 34.3 | 26 | 0 | Brazil | Human |
| HOU1445_VS_ip3 | 13981 | 30.5 | 20 | 0 | Brazil | Human |
| HOU1445_VS_ip4 | 18332 | 31.9 | 31 | 0 | Brazil | Human |
| HOU1445_VS_ip5 | 9917 | 30.5 | 13 | 0 | Brazil | Human |
| HST_077_ip1 | 19684 | 33.3 | 23 | 0 | Lebanon | Human |
| HST_077_ip2 | 28974 | 30.9 | 43 | 0 | Lebanon | Human |
| HST_077_ip3 | 39678 | 31.7 | 35 | 0 | Lebanon | Human |
| HST_077_ip4 | 27783 | 28.9 | 52 | 0 | Lebanon | Human |
| HST_077_ip5 | 12411 | 32.8 | 17 | 0 | Lebanon | Human |
| HST_077_ip6 | 9804 | 32.4 | 10 | 0 | Lebanon | Human |
| HST_077_ip7 | 16011 | 34.8 | 20 | 0 | Lebanon | Human |
| HST_105_ip1 | 15631 | 30.5 | 20 | 0 | Lebanon | Human |
| HU8_ip1 | 14891 | 32.3 | 16 | 0 | Turkey | Human |
| HU8_ip2 | 9803 | 32.3 | 10 | 0 | Turkey | Human |
| HU8_ip4 | 29860 | 33.5 | 36 | 0 | Turkey | Human |
| HU8_ip5 | 27813 | 32.3 | 40 | 0 | Turkey | Human |
| HU8_ip6 | 18679 | 33.1 | 39 | 0 | Turkey | Human |
| HU8_ip7 | 5440 | 28.1 | 11 | 0 | Turkey | Human |
| HU8_ip8 | 66203 | 31.7 | 83 | 0 | Turkey | Human |
| HUK16_ip1 | 18874 | 30 | 31 | 0 | France | Other |
| HUK16_ip2 | 19583 | 31.1 | 28 | 0 | France | Other |
| HUK16_ip3 | 14042 | 32.2 | 26 | 0 | France | Other |
| HUV05_ip1 | 19684 | 33.3 | 23 | 0 | United State | Human |
| HUV05_ip2 | 20690 | 30.8 | 24 | 0 | United State | Human |
| HUV05_ip3 | 7022 | 26.1 | 14 | 0 | United State | Human |
| HZW450_ip1 | 11720 | 31.4 | 17 | 0 | China | Human |
| HZW450_ip2 | 8159 | 26.7 | 16 | 0 | China | Human |
| HZW450_ip4 | 8314 | 28.3 | 10 | 0 | China | Human |
| IF6SW_P2_ip1 | 26668 | 31.6 | 37 | 0 | Space Station | Other |
| IF6SW_P2_ip4 | 15081 | 34.5 | 18 | 0 | Space Station | Other |
| IF6SW_P2_RA_ip1 | 26668 | 31.6 | 37 | 0 | Space Station | Other |
| IF6SW_P2_RA_ip3 | 41271 | 33.4 | 63 | 0 | Space Station | Other |
| IF7SW_P3_ip2 | 22067 | 28.3 | 23 | 0 | Space Station | Other |
| IF7SW_P3_ip3 | 26216 | 34.8 | 36 | 0 | Space Station | Other |
| IF7SW_P3_ip4 | 10962 | 33.5 | 27 | 0 | Space Station | Other |
| JH1_ip1 | 22719 | 34.1 | 23 | 0 | Unknown | Unknown |
| JH1_ip5 | 6113 | 28.6 | 7 | 0 | Unknown | Unknown |
| JH1_ip6 | 22810 | 28.7 | 24 | 0 | Unknown | Unknown |
| JH9_ip1 | 22719 | 34.1 | 23 | 0 | United State | Unknown |
| JH9_ip5 | 6113 | 28.6 | 7 | 0 | United State | Unknown |
| JH9_ip6 | 22810 | 28.7 | 24 | 0 | United State | Unknown |
| JS395_ip1 | 18772 | 30.3 | 18 | 0 | Switzerland | Human |
| JS395_ip2 | 19871 | 32.6 | 18 | 0 | Switzerland | Human |
| JS395_ip3 | 40601 | 30.8 | 45 | 0 | Switzerland | Human |
| JS395_ip4 | 6428 | 28.5 | 9 | 0 | Switzerland | Human |
| JS395_ip5 | 32511 | 32.8 | 38 | 1 | Switzerland | Human |
| KINW6056_ip1 | 30396 | 32.4 | 51 | 0 | United State | Human |
| KINW6056_ip2 | 12199 | 27.3 | 16 | 0 | United State | Human |
| KINW6056_ip4 | 18098 | 32.2 | 32 | 0 | United State | Human |
| KINW6056_ip5 | 28033 | 33.2 | 32 | 0 | United State | Human |
| KINW6056_ip6 | 10039 | 31.9 | 9 | 0 | United State | Human |
| KINW6056_ip8 | 10465 | 33.6 | 24 | 0 | United State | Human |
| KLT6_ip1 | 7050 | 34.6 | 7 | 0 | Switzerland | Other |
| KLT6_ip2 | 19692 | 33.3 | 23 | 0 | Switzerland | Other |
| KLT6_ip3 | 10839 | 28.6 | 11 | 0 | Switzerland | Other |
| KT_314250_ip1 | 29743 | 32.2 | 44 | 0 | Malaysia | Human |
| KT_314250_ip2 | 11141 | 31 | 12 | 0 | Malaysia | Human |
| KT_314250_ip3 | 29083 | 33.4 | 36 | 0 | Malaysia | Human |
| KT_314250_ip4 | 26835 | 30.7 | 30 | 0 | Malaysia | Human |
| KT_Y21_ip2 | 17961 | 31.6 | 23 | 0 | Malaysia | Human |
| LCT_SAG_ip1 | 21028 | 29.8 | 25 | 0 | China | Human |
| LCT_SAM_ip1 | 9803 | 32.3 | 10 | 0 | China | Human |
| LCT_SAM_ip2 | 21471 | 31.4 | 24 | 0 | China | Human |
| LCT_SAO_ip1 | 21774 | 31.9 | 26 | 0 | China | Human |
| LCT_SAO_ip2 | 4826 | 32.9 | 7 | 0 | China | Human |
| LCT_SAO_ip3 | 9803 | 32.3 | 10 | 0 | China | Human |
| LCT_SAO_ip4 | 38539 | 31.5 | 41 | 0 | China | Human |
| LCT_SAO_ip5 | 8354 | 27.7 | 15 | 0 | China | Human |
| LCT_SAO_ip6 | 15047 | 30.7 | 23 | 0 | China | Human |
| LCT_SAS_ip1 | 21052 | 30.2 | 24 | 0 | China | Human |
| LVP2_ip1 | 26632 | 32.4 | 34 | 0 | India | Human |
| LVP2_ip2 | 25548 | 31.8 | 35 | 0 | India | Human |
| LVP2_ip4 | 12143 | 32.7 | 14 | 0 | India | Human |
| LVP2_ip6 | 23728 | 33.4 | 40 | 0 | India | Human |
| LVP5_ip1 | 28765 | 32.9 | 44 | 0 | India | Human |
| LVP5_ip2 | 26226 | 32 | 35 | 0 | India | Human |
| LVP5_ip4 | 22248 | 30.9 | 31 | 0 | India | Human |
| LVP5_ip5 | 19691 | 33.5 | 24 | 0 | India | Human |
| LVP5_ip6 | 27392 | 33.5 | 35 | 0 | India | Human |
| M1_HF937103_ip2 | 19684 | 33.3 | 23 | 0 | Denmark | Human |
| M1_HF937103_ip3 | 7017 | 26.1 | 14 | 0 | Denmark | Human |
| M3_ip1 | 21299 | 30 | 35 | 0 | China | Swine |
| M3_ip2 | 27602 | 33.2 | 29 | 0 | China | Swine |
| M6_ip1 | 21299 | 30 | 34 | 0 | China | Swine |
| M6_ip2 | 16183 | 32 | 21 | 0 | China | Swine |
| M013_ip1 | 11719 | 31.4 | 16 | 0 | China | Human |
| M013_ip2 | 8155 | 26.7 | 16 | 0 | China | Human |
| M013_ip4 | 8123 | 28.4 | 9 | 0 | China | Human |
| M48_ip2 | 19684 | 33.3 | 23 | 0 | China | Swine |
| M48_ip3 | 9804 | 32.5 | 10 | 0 | China | Swine |
| M48_ip4 | 8353 | 27.7 | 15 | 0 | China | Swine |
| M48_ip8 | 7064 | 32.1 | 11 | 0 | China | Swine |
| M51_ip2 | 7022 | 26.1 | 14 | 0 | China | Swine |
| M51_ip4 | 19684 | 33.3 | 23 | 0 | China | Swine |
| M116_ip1 | 19684 | 33.3 | 23 | 0 | Viet Nam | Human |
| M116_ip10 | 20653 | 31.1 | 28 | 0 | Viet Nam | Human |
| M116_ip11 | 24870 | 33.7 | 30 | 0 | Viet Nam | Human |
| M116_ip2 | 9804 | 32.4 | 10 | 0 | Viet Nam | Human |
| M116_ip3 | 78019 | 31.6 | 63 | 0 | Viet Nam | Human |
| M116_ip4 | 35397 | 31.8 | 50 | 0 | Viet Nam | Human |
| M116_ip5 | 7539 | 31.5 | 12 | 0 | Viet Nam | Human |
| M116_ip7 | 35723 | 29.9 | 62 | 0 | Viet Nam | Human |
| M116_ip9 | 24265 | 32.3 | 24 | 0 | Viet Nam | Human |
| M0239_ip1 | 7796 | 26.5 | 15 | 0 | United State | Human |
| M278_ip1 | 9804 | 32.3 | 10 | 0 | Portugal | Human |
| M278_ip2 | 29617 | 33 | 35 | 0 | Portugal | Human |
| M278_ip4 | 41962 | 32.5 | 66 | 0 | Portugal | Human |
| M278_ip5 | 10366 | 30.9 | 15 | 0 | Portugal | Human |
| M0359_ip1 | 12199 | 27.3 | 16 | 0 | United State | Human |
| M0359_ip2 | 29641 | 33.3 | 38 | 0 | United State | Human |
| M0359_ip3 | 17745 | 32.1 | 29 | 0 | United State | Human |
| M0359_ip4 | 9204 | 33.4 | 20 | 0 | United State | Human |
| M0396_ip1 | 19684 | 33.4 | 24 | 0 | United State | Human |
| M0396_ip3 | 16254 | 30.1 | 16 | 0 | United State | Human |
| M0406_ip1 | 23926 | 32.4 | 38 | 0 | United State | Human |
| M0406_ip2 | 27356 | 33.4 | 36 | 0 | United State | Human |
| M0406_ip3 | 26114 | 32.1 | 38 | 0 | United State | Human |
| M0406_ip4 | 22719 | 34.1 | 23 | 0 | United State | Human |
| M0406_ip5 | 21318 | 33.5 | 38 | 0 | United State | Human |
| M0406_ip8 | 21267 | 28.6 | 23 | 0 | United State | Human |
| M0426_ip1 | 12199 | 27.3 | 16 | 0 | United State | Human |
| M0426_ip2 | 39013 | 33.3 | 57 | 0 | United State | Human |
| M0426_ip3 | 22718 | 34.1 | 24 | 0 | United State | Human |
| M0426_ip5 | 12031 | 30.4 | 15 | 0 | United State | Human |
| M0426_ip6 | 12520 | 33.6 | 30 | 0 | United State | Human |
| M0426_ip7 | 12021 | 34.8 | 29 | 0 | United State | Human |
| M0513_ip2 | 9630 | 27.7 | 14 | 0 | United State | Human |
| M0513_ip4 | 10030 | 31.9 | 9 | 0 | United State | Human |
| M0513_ip5 | 16694 | 31.7 | 22 | 0 | United State | Human |
| M0513_ip7 | 12199 | 27.3 | 16 | 0 | United State | Human |
| M592_ip1 | 86192 | 32.3 | 73 | 0 | Syria | Human |
| M592_ip10 | 31104 | 32.6 | 33 | 0 | Syria | Human |
| M592_ip11 | 23840 | 34 | 33 | 0 | Syria | Human |
| M592_ip2 | 19683 | 33.3 | 23 | 0 | Syria | Human |
| M592_ip3 | 35358 | 31.8 | 50 | 0 | Syria | Human |
| M592_ip4 | 28551 | 31 | 33 | 0 | Syria | Human |
| M592_ip5 | 14249 | 30.9 | 15 | 0 | Syria | Human |
| M592_ip6 | 38616 | 30 | 68 | 0 | Syria | Human |
| M592_ip7 | 9804 | 32.3 | 10 | 0 | Syria | Human |
| M592_ip9 | 18808 | 34.2 | 21 | 0 | Syria | Human |
| M0684_ip1 | 26641 | 31.6 | 37 | 0 | United State | Human |
| M0684_ip2 | 22724 | 33.2 | 28 | 0 | United State | Human |
| M0684_ip3 | 29286 | 32 | 41 | 0 | United State | Human |
| M0690_ip1 | 15943 | 32.1 | 24 | 0 | United State | Human |
| M0690_ip2 | 13600 | 32.8 | 27 | 0 | United State | Human |
| M0690_ip3 | 22724 | 33.2 | 28 | 0 | United State | Human |
| M0690_ip4 | 29017 | 31.9 | 40 | 0 | United State | Human |
| M0690_ip5 | 22719 | 34.1 | 23 | 0 | United State | Human |
| M0715_ip1 | 26641 | 31.6 | 37 | 0 | United State | Human |
| M0715_ip2 | 22719 | 34.1 | 23 | 0 | United State | Human |
| M0715_ip3 | 22724 | 33.2 | 28 | 0 | United State | Human |
| M0715_ip4 | 33214 | 32.5 | 52 | 0 | United State | Human |
| M0745_ip1 | 15811 | 30 | 30 | 0 | United State | Human |
| M0745_ip2 | 22719 | 34.1 | 23 | 0 | United State | Human |
| M0745_ip4 | 8628 | 26.9 | 11 | 0 | United State | Human |
| M0821_ip1 | 26641 | 31.6 | 37 | 0 | United State | Human |
| M0821_ip3 | 22724 | 33.2 | 28 | 0 | United State | Human |
| M0821_ip4 | 33214 | 32.5 | 52 | 0 | United State | Human |
| M0896_ip1 | 16830 | 29.8 | 32 | 0 | United State | Human |
| M0896_ip2 | 8628 | 26.9 | 11 | 0 | United State | Human |
| M0896_ip4 | 29017 | 31.9 | 40 | 0 | United State | Human |
| M0896_ip5 | 22719 | 34.1 | 23 | 0 | United State | Human |
| M996_ip1 | 9804 | 32.4 | 10 | 0 | China | Human |
| M996_ip2 | 19684 | 33.3 | 23 | 0 | China | Human |
| M996_ip3 | 33958 | 31.1 | 50 | 0 | China | Human |
| M996_ip4 | 23477 | 31.2 | 16 | 0 | China | Human |
| M996_ip5 | 50070 | 31.6 | 44 | 0 | China | Human |
| M1169_ip2 | 16830 | 31.7 | 19 | 0 | United State | Human |
| M1169_ip3 | 16564 | 31.4 | 30 | 0 | United State | Human |
| M1216_ip1 | 35889 | 30.5 | 55 | 0 | United State | Human |
| M1216_ip2 | 31737 | 30.5 | 32 | 3 | United State | Human |
| M1216_ip3 | 13922 | 31.8 | 18 | 0 | United State | Human |
| M1216_ip4 | 19685 | 33.3 | 23 | 0 | United State | Human |
| M1242_ip2 | 17240 | 30.5 | 18 | 0 | United State | Human |
| M1311_ip1 | 28951 | 33.7 | 29 | 0 | United State | Human |
| M1311_ip2 | 21037 | 31.9 | 26 | 0 | United State | Human |
| M1311_ip3 | 22842 | 32.7 | 40 | 0 | United State | Human |
| M1311_ip4 | 8628 | 26.9 | 11 | 0 | United State | Human |
| M1311_ip5 | 15931 | 32.1 | 24 | 0 | United State | Human |
| M35954_ip1 | 19846 | 34.5 | 22 | 0 | United State | Human |
| M64191_ip1 | 19684 | 33.3 | 23 | 0 | United State | Human |
| M64191_ip2 | 20640 | 30.8 | 24 | 0 | United State | Human |
| M64191_ip3 | 7022 | 26.1 | 14 | 0 | United State | Human |
| MAL9_ip1 | 9804 | 32.5 | 10 | 0 | Malaysia | Human |
| MAL9_ip2 | 19684 | 33.3 | 23 | 0 | Malaysia | Human |
| MAL9_ip3 | 69909 | 31.2 | 60 | 0 | Malaysia | Human |
| MAL9_ip4 | 43559 | 31.3 | 58 | 0 | Malaysia | Human |
| MAL9_ip5 | 49838 | 30.7 | 76 | 0 | Malaysia | Human |
| MAL9_ip6 | 20978 | 34.6 | 20 | 0 | Malaysia | Human |
| MAL9_ip7 | 26990 | 32.3 | 43 | 0 | Malaysia | Human |
| MAL11_ip1 | 7021 | 26.1 | 14 | 0 | Malaysia | Human |
| MAL11_ip2 | 9804 | 32.3 | 10 | 0 | Malaysia | Human |
| MAL11_ip3 | 19684 | 33.3 | 23 | 0 | Malaysia | Human |
| MAL11_ip5 | 33980 | 32.2 | 48 | 0 | Malaysia | Human |
| MAL11_ip6 | 34594 | 31.2 | 48 | 0 | Malaysia | Human |
| MAL11_ip7 | 36003 | 30.4 | 61 | 0 | Malaysia | Human |
| MAL11_ip8 | 21861 | 30.4 | 32 | 0 | Malaysia | Human |
| MCRF184_ip1 | 27820 | 31.5 | 40 | 0 | United State | Human |
| MCRF184_ip2 | 28576 | 32.9 | 33 | 0 | United State | Human |
| MI_ip1 | 22719 | 34.1 | 23 | 0 | United State | Human |
| MI_ip3 | 6113 | 28.6 | 7 | 0 | United State | Human |
| MI_ip5 | 18831 | 27.7 | 21 | 0 | United State | Human |
| Mq2T_ip1 | 17010 | 30 | 33 | 0 | India | Human |
| Mq2T_ip2 | 10851 | 32.6 | 17 | 0 | India | Human |
| MR1_ip2 | 20159 | 34.2 | 21 | 0 | Poland | Human |
| MR1_ip3 | 19897 | 36.5 | 22 | 0 | Poland | Human |
| MR1_ip4 | 14208 | 33.1 | 19 | 0 | Poland | Human |
| MRGR3_ip1 | 35098 | 29.5 | 59 | 0 | Switzerland | Human |
| MRGR3_ip10 | 18025 | 33.7 | 20 | 0 | Switzerland | Human |
| MRGR3_ip11 | 28351 | 30.5 | 34 | 0 | Switzerland | Human |
| MRGR3_ip12 | 26453 | 30.9 | 35 | 0 | Switzerland | Human |
| MRGR3_ip13 | 19885 | 31.7 | 33 | 0 | Switzerland | Human |
| MRGR3_ip2 | 15217 | 32.4 | 18 | 0 | Switzerland | Human |
| MRGR3_ip3 | 11750 | 29.8 | 13 | 0 | Switzerland | Human |
| MRGR3_ip4 | 80287 | 31.4 | 66 | 0 | Switzerland | Human |
| MRGR3_ip5 | 34263 | 33.6 | 52 | 0 | Switzerland | Human |
| MRGR3_ip6 | 9804 | 32.4 | 10 | 0 | Switzerland | Human |
| MRGR3_ip7 | 19684 | 33.3 | 23 | 0 | Switzerland | Human |
| MRGR3_ip9 | 42865 | 31.7 | 61 | 0 | Switzerland | Human |
| MRSA_041T_B_ip1 | 6754 | 27.7 | 8 | 0 | Tanzania | Unknown |
| MRSA_CVM43477_ip1 | 28790 | 33.2 | 50 | 0 | United State | Other |
| MRSA_FGWU_ip1 | 15811 | 30 | 30 | 0 | United Kingdom | Human |
| MRSA_FGWU_ip2 | 22520 | 33.2 | 26 | 0 | United Kingdom | Human |
| MRSA_FGWU_ip4 | 33214 | 32.4 | 54 | 0 | United Kingdom | Human |
| MRSA_FGWU_ip5 | 10039 | 32 | 10 | 0 | United Kingdom | Human |
| MRSA_FKPC_ip1 | 14027 | 28.8 | 18 | 2 | United State | Human |
| MRSA_FKPC_ip3 | 29150 | 32.7 | 46 | 0 | United State | Human |
| MRSA_FKPC_ip5 | 12734 | 32.2 | 23 | 0 | United State | Human |
| MRSA_FKSE_ip3 | 14676 | 30.1 | 26 | 0 | United State | Human |
| MRSA_FKSE_ip4 | 29018 | 31.9 | 39 | 0 | United State | Human |
| MRSA_FKTK_ip1 | 12124 | 31.3 | 15 | 0 | United State | Human |
| MRSA_FKTK_ip3 | 8196 | 25.6 | 15 | 0 | United State | Human |
| MRSA_FKTN_ip2 | 15811 | 30 | 29 | 0 | United State | Human |
| MRSA_FKTN_ip3 | 28370 | 33 | 46 | 0 | United State | Human |
| MRSA_FKTN_ip4 | 27240 | 33.2 | 34 | 0 | United State | Human |
| MRSA_FKTN_ip5 | 14321 | 32.7 | 21 | 0 | United State | Human |
| MRSA_FKUV_ip1 | 12199 | 27.3 | 16 | 0 | United State | Human |
| MRSA_FKUV_ip2 | 5397 | 27.2 | 15 | 0 | United State | Human |
| MRSA_FKVP_ip3 | 15030 | 31.1 | 19 | 0 | United State | Human |
| MRSA_FKVP_ip4 | 15036 | 34.9 | 18 | 0 | United State | Human |
| MRSA_FKVP_ip5 | 10146 | 34.4 | 29 | 0 | United State | Human |
| MRSA_FKWA_ip1 | 19684 | 33.4 | 24 | 0 | United State | Human |
| MRSA_FKWA_ip2 | 16253 | 30.2 | 16 | 0 | United State | Human |
| MRSA_FKWH_ip1 | 12199 | 27.3 | 16 | 0 | United State | Human |
| MRSA_FKWH_ip2 | 34499 | 31.9 | 48 | 0 | United State | Human |
| MRSA_FKYJ_ip2 | 7796 | 26.5 | 15 | 0 | United State | Human |
| MRSA_FKYJ_ip3 | 5844 | 31.7 | 7 | 0 | United State | Human |
| MRSA_FKYJ_ip4 | 16019 | 30.5 | 17 | 0 | United State | Human |
| MRSA_PR1_ip1 | 29571 | 31 | 43 | 0 | Malaysia | Human |
| MRSA_PR1_ip2 | 21110 | 32 | 34 | 0 | Malaysia | Human |
| MRSA_PR1_ip3 | 7065 | 32.1 | 10 | 0 | Malaysia | Human |
| MRSA_PR1_ip4 | 19684 | 33.3 | 23 | 0 | Malaysia | Human |
| MRSA_PR1_ip5 | 9819 | 32.3 | 10 | 0 | Malaysia | Human |
| MRSA_PR1_ip6 | 27856 | 33.3 | 35 | 0 | Malaysia | Human |
| MRSA_S1_ip2 | 7022 | 26.1 | 14 | 0 | Switzerland | Human |
| MRSA_S1_ip3 | 16516 | 30.6 | 20 | 0 | Switzerland | Human |
| MRSA_S1_ip4 | 19684 | 33.3 | 23 | 0 | Switzerland | Human |
| MRSA_S2_ip2 | 20640 | 30.8 | 24 | 0 | Switzerland | Human |
| MRSA_S2_ip3 | 7022 | 26.1 | 14 | 0 | Switzerland | Human |
| MRSA_S2_ip4 | 19684 | 33.3 | 23 | 0 | Switzerland | Human |
| MRSA_S4_ip1 | 16311 | 30.5 | 20 | 0 | Switzerland | Human |
| MRSA_S4_ip2 | 19684 | 33.3 | 23 | 0 | Switzerland | Human |
| MRSA_S4_ip3 | 7022 | 26.1 | 14 | 0 | Switzerland | Human |
| MRSA_S4_ip6 | 19542 | 31.4 | 29 | 0 | Switzerland | Human |
| MRSA_S5_ip1 | 20640 | 30.8 | 24 | 0 | Switzerland | Human |
| MRSA_S5_ip2 | 7022 | 26.1 | 14 | 0 | Switzerland | Human |
| MRSA_S5_ip4 | 19684 | 33.3 | 23 | 0 | Switzerland | Human |
| MRSA08_ip1 | 18830 | 27.7 | 21 | 0 | Tanzania | Unknown |
| MRSA08_ip4 | 21082 | 33.1 | 35 | 0 | Tanzania | Unknown |
| MRSA012TA_ip3 | 19012 | 31.7 | 24 | 0 | Tanzania | Unknown |
| MRSA012TA_ip4 | 39539 | 33.3 | 53 | 0 | Tanzania | Unknown |
| MRSA012TA_ip5 | 20124 | 30.4 | 20 | 0 | Tanzania | Unknown |
| MRSA012TB_ip1 | 19672 | 33.4 | 24 | 0 | Tanzania | Unknown |
| MRSA071_ip1 | 8238 | 24.7 | 16 | 0 | Tanzania | Unknown |
| MRSA071_ip3 | 15631 | 30.5 | 20 | 0 | Tanzania | Unknown |
| MRSA079A_ip2 | 19672 | 33.4 | 23 | 0 | Tanzania | Unknown |
| MRSA140_ip1 | 8196 | 25.6 | 16 | 0 | Tanzania | Unknown |
| MRSA140_ip2 | 11445 | 31.8 | 14 | 0 | Tanzania | Unknown |
| MRSA140_ip3 | 23855 | 31.9 | 27 | 0 | Tanzania | Unknown |
| MRSA140_ip4 | 48122 | 33.1 | 66 | 0 | Tanzania | Unknown |
| MRSA207_ip1 | 36531 | 32.2 | 44 | 0 | Tanzania | Unknown |
| MRSA207_ip5 | 19684 | 33.3 | 23 | 0 | Tanzania | Unknown |
| MRSA252_ip1 | 22718 | 34.1 | 24 | 0 | United Kingdom | Human |
| MRSA252_ip4 | 12199 | 27.3 | 16 | 0 | United Kingdom | Human |
| MRSA035668_ip1 | 19684 | 33.4 | 24 | 0 | Tanzania | Unknown |
| MRSA035668_ip2 | 16296 | 29.7 | 16 | 0 | Tanzania | Unknown |
| MRSA041776_938686_ip1 | 18999 | 31.6 | 24 | 0 | Tanzania | Unknown |
| MRSA041776_938686_ip3 | 18830 | 27.7 | 21 | 0 | Tanzania | Unknown |
| MRSA041776_938686_ip4 | 36009 | 33.4 | 52 | 0 | Tanzania | Unknown |
| MRSA041776_938686_ip5 | 19018 | 31.6 | 23 | 0 | Tanzania | Unknown |
| MS4_ip1 | 11719 | 31.4 | 18 | 0 | China | Human |
| MS4_ip2 | 8159 | 26.7 | 16 | 0 | China | Human |
| MS4_ip4 | 8314 | 28.3 | 10 | 0 | China | Human |
| MS4_LZD100_ip2 | 4887 | 30 | 10 | 0 | China | Human |
| MS4_T60_ip2 | 8159 | 26.7 | 16 | 0 | China | Unknown |
| MS4_T60_ip3 | 14005 | 31.2 | 25 | 0 | China | Unknown |
| MSSA_FKNV_ip3 | 18829 | 27.7 | 21 | 0 | United State | Human |
| MSSA_FKNV_ip4 | 13649 | 26.9 | 21 | 0 | United State | Human |
| MSSA_FKOF_ip1 | 21742 | 30.6 | 29 | 0 | United State | Human |
| MSSA_FKOF_ip3 | 25760 | 31.5 | 35 | 0 | United State | Human |
| MSSA_FKTB_ip1 | 9510 | 31.7 | 16 | 0 | United State | Human |
| MSSA_FKTY_ip1 | 23709 | 33.6 | 24 | 0 | United State | Human |
| MSSA_T188_ip1 | 18874 | 30 | 33 | 0 | Tanzania | Unknown |
| MSSA_T188_ip2 | 22387 | 31.4 | 29 | 0 | Tanzania | Unknown |
| MSSA1_ip2 | 36046 | 30.5 | 50 | 0 | United State | Human |
| MSSA1_ip3 | 16058 | 33.9 | 33 | 0 | United State | Human |
| MSSA1_ip4 | 29799 | 33.3 | 42 | 0 | United State | Human |
| MSSAT120_ip1 | 7022 | 26.1 | 14 | 0 | Tanzania | Unknown |
| MSSAT120_ip4 | 19684 | 33.3 | 23 | 0 | Tanzania | Unknown |
| MSSAT120_ip5 | 15725 | 30.4 | 18 | 0 | Tanzania | Unknown |
| MU3_ip1 | 22719 | 34.1 | 22 | 0 | Japan | Human |
| MU3_ip2 | 40942 | 32.5 | 45 | 0 | Japan | Human |
| MU3_ip4 | 6113 | 28.6 | 7 | 0 | Japan | Human |
| MU3_ip5 | 18830 | 27.7 | 21 | 0 | Japan | Human |
| MU3_ip6 | 19982 | 30.9 | 26 | 0 | Japan | Human |
| MU4_ip1 | 9803 | 32.3 | 10 | 0 | Turkey | Human |
| MU4_ip2 | 18546 | 34.4 | 14 | 0 | Turkey | Human |
| MU4_ip3 | 34064 | 33.9 | 38 | 0 | Turkey | Human |
| MU4_ip5 | 22023 | 33.6 | 25 | 0 | Turkey | Human |
| MU4_ip6 | 29332 | 31.1 | 30 | 0 | Turkey | Human |
| MU4_ip7 | 7039 | 28.5 | 8 | 0 | Turkey | Human |
| MU4_ip8 | 16546 | 31.4 | 18 | 0 | Turkey | Human |
| Mu50_ip1 | 22719 | 34.1 | 22 | 0 | Japan | Unknown |
| Mu50_ip2 | 40942 | 32.5 | 45 | 0 | Japan | Unknown |
| Mu50_ip4 | 6113 | 28.6 | 7 | 0 | Japan | Unknown |
| Mu50_ip5 | 18830 | 27.7 | 21 | 0 | Japan | Unknown |
| Mu50_ip6 | 25826 | 31 | 32 | 0 | Japan | Unknown |
| MUM270_ip3 | 15946 | 34.1 | 23 | 0 | India | Human |
| MUM270_ip4 | 14299 | 31.7 | 21 | 0 | India | Human |
| MUM475_ip3 | 4382 | 28.7 | 5 | 0 | India | Human |
| MW2_ip1 | 17731 | 29.8 | 20 | 0 | United State | Human |
| 08_01672_ip1 | 39813 | 32.1 | 53 | 0 | Germany | Human |
| 08_01672_ip5 | 7234 | 26.6 | 9 | 0 | Germany | Human |
| N315_ip1 | 22719 | 34.1 | 23 | 0 | Japan | Human |
| N315_ip2 | 6113 | 28.6 | 7 | 0 | Japan | Human |
| N315_ip3 | 18830 | 27.7 | 21 | 0 | Japan | Human |
| N315_ip5 | 19972 | 30.9 | 26 | 0 | Japan | Human |
| Na21_ip1 | 9792 | 32.3 | 10 | 0 | Sri Lanka | Human |
| Na21_ip10 | 42584 | 30.2 | 44 | 0 | Sri Lanka | Human |
| Na21_ip11 | 9490 | 33.2 | 18 | 0 | Sri Lanka | Human |
| Na21_ip12 | 16597 | 33.7 | 30 | 0 | Sri Lanka | Human |
| Na21_ip13 | 5872 | 34.5 | 9 | 0 | Sri Lanka | Human |
| Na21_ip14 | 21427 | 31.6 | 38 | 0 | Sri Lanka | Human |
| Na21_ip2 | 19684 | 33.3 | 23 | 0 | Sri Lanka | Human |
| Na21_ip3 | 70230 | 31.6 | 57 | 0 | Sri Lanka | Human |
| Na21_ip4 | 20774 | 33.5 | 21 | 0 | Sri Lanka | Human |
| Na21_ip5 | 40736 | 31.8 | 53 | 0 | Sri Lanka | Human |
| Na21_ip6 | 32917 | 31 | 33 | 0 | Sri Lanka | Human |
| Na21_ip8 | 22679 | 33.9 | 26 | 0 | Sri Lanka | Human |
| Na21_ip9 | 14578 | 29.1 | 36 | 0 | Sri Lanka | Human |
| NA32_ip1 | 7021 | 26.1 | 14 | 0 | Denmark | Human |
| NA32_ip2 | 9804 | 32.3 | 10 | 0 | Denmark | Human |
| NA32_ip3 | 19684 | 33.3 | 23 | 0 | Denmark | Human |
| NA32_ip6 | 32194 | 32.3 | 30 | 0 | Denmark | Human |
| NA32_ip7 | 35878 | 30.4 | 43 | 0 | Denmark | Human |
| NA32_ip8 | 28448 | 34.7 | 34 | 0 | Denmark | Human |
| NA32_ip9 | 14426 | 32.9 | 25 | 0 | Denmark | Human |
| NCTC_8325_ip1 | 19684 | 33.3 | 23 | 0 | Unknown | Unknown |
| NCTC_8325_ip2 | 7021 | 26.1 | 14 | 0 | Unknown | Unknown |
| Newman_ip2 | 19684 | 33.3 | 23 | 0 | Unknown | Unknown |
| Newman_ip4 | 7021 | 26.1 | 14 | 0 | Unknown | Unknown |
| NGS_ED_1006_ip1 | 9055 | 26.8 | 17 | 0 | United Kingdom | Human |
| NGS_ED_1006_ip3 | 12198 | 27.3 | 15 | 0 | United Kingdom | Human |
| NMR02_ip1 | 58243 | 31.4 | 46 | 0 | India | Human |
| NMR02_ip2 | 21936 | 30.7 | 26 | 0 | India | Human |
| NMR02_ip3 | 38453 | 31 | 51 | 0 | India | Human |
| NMR02_ip4 | 17631 | 28.6 | 20 | 0 | India | Human |
| NMR02_ip5 | 14577 | 29.1 | 36 | 0 | India | Human |
| NMR02_ip6 | 11479 | 33.5 | 23 | 0 | India | Human |
| NMR02_ip7 | 10955 | 35 | 27 | 0 | India | Human |
| NMR02_ip8 | 19684 | 33.3 | 23 | 0 | India | Human |
| NMR02_ip9 | 9803 | 32.3 | 10 | 0 | India | Human |
| NMR05_ip1 | 23893 | 30.7 | 27 | 0 | India | Human |
| NMR05_ip10 | 9847 | 33.2 | 20 | 0 | India | Human |
| NMR05_ip11 | 19684 | 33.3 | 23 | 0 | India | Human |
| NMR05_ip12 | 9804 | 32.3 | 10 | 0 | India | Human |
| NMR05_ip2 | 34365 | 31.1 | 47 | 0 | India | Human |
| NMR05_ip3 | 49745 | 32.4 | 34 | 0 | India | Human |
| NMR05_ip4 | 19073 | 31.8 | 20 | 0 | India | Human |
| NMR05_ip6 | 20228 | 34.4 | 21 | 0 | India | Human |
| NMR05_ip7 | 13189 | 28.5 | 34 | 0 | India | Human |
| NMR05_ip8 | 7017 | 26.1 | 14 | 0 | India | Human |
| NMR05_ip9 | 15446 | 29 | 17 | 0 | India | Human |
| NRS2_ip1 | 6113 | 28.6 | 7 | 0 | Japan | Human |
| NRS2_ip3 | 28949 | 33.7 | 31 | 0 | Japan | Human |
| NRS2_ip4 | 18831 | 27.7 | 21 | 0 | Japan | Human |
| NRS2_ip6 | 12788 | 32.4 | 21 | 0 | Japan | Human |
| NRS158_ip3 | 16357 | 30.4 | 19 | 0 | France | Human |
| O11_ip1 | 35034 | 33 | 41 | 0 | France | Sheep |
| O11_ip2 | 31194 | 33 | 51 | 0 | France | Sheep |
| O11_ip3 | 28919 | 33.9 | 32 | 0 | France | Sheep |
| O11_ip5 | 13905 | 29.2 | 14 | 0 | France | Sheep |
| O46_ip1 | 16270 | 33.8 | 27 | 0 | France | Sheep |
| O46_ip4 | 13066 | 28.9 | 13 | 0 | France | Sheep |
| O46_ip5 | 15559 | 33.4 | 23 | 0 | France | Sheep |
| O46_ip6 | 10356 | 33.5 | 19 | 0 | France | Sheep |
| OC3_ip1 | 10252 | 33.8 | 15 | 0 | Russia | Human |
| OC3_ip3 | 27862 | 33.5 | 38 | 0 | Russia | Human |
| OC3_ip4 | 9804 | 32.4 | 10 | 0 | Russia | Human |
| OC3_ip6 | 11059 | 30.6 | 18 | 0 | Russia | Human |
| OCMM6035_ip2 | 21990 | 30.6 | 23 | 0 | United State | Human |
| OCMM6035_ip3 | 37202 | 33.2 | 50 | 0 | United State | Human |
| OCMM6035_ip4 | 17732 | 29.8 | 20 | 0 | United State | Human |
| OCMM6035_ip5 | 31970 | 33.5 | 35 | 0 | United State | Human |
| OCMM6035_ip6 | 7305 | 35.2 | 18 | 0 | United State | Human |
| P32_ip1 | 7020 | 26.1 | 14 | 0 | Poland | Human |
| P32_ip10 | 11956 | 32.5 | 24 | 0 | Poland | Human |
| P32_ip2 | 19684 | 33.3 | 23 | 0 | Poland | Human |
| P32_ip3 | 9803 | 32.3 | 10 | 0 | Poland | Human |
| P32_ip4 | 59391 | 31.9 | 43 | 0 | Poland | Human |
| P32_ip8 | 29713 | 34 | 35 | 0 | Poland | Human |
| P32_ip9 | 17408 | 33.7 | 32 | 0 | Poland | Human |
| P151_ip1 | 22107 | 31.6 | 26 | 0 | Malaysia | Human |
| P151_ip2 | 22718 | 34.1 | 23 | 0 | Malaysia | Human |
| P151_ip3 | 24923 | 32.9 | 37 | 0 | Malaysia | Human |
| P151_ip4 | 17619 | 31 | 24 | 0 | Malaysia | Human |
| P151_ip5 | 18829 | 27.7 | 21 | 0 | Malaysia | Human |
| P151_ip7 | 9894 | 34.3 | 24 | 0 | Malaysia | Human |
| P151_ip8 | 1325 | 32.5 | 6 | 0 | Malaysia | Human |
| PB1_1_ip1 | 13977 | 31 | 21 | 0 | United Kingdom | Human |
| PB1_1_ip2 | 8744 | 34 | 19 | 0 | United Kingdom | Human |
| PB1_1_ip3 | 17079 | 34.5 | 21 | 0 | United Kingdom | Human |
| PE009_ip1 | 7022 | 26.1 | 14 | 0 | United State | Human |
| PE009_ip2 | 16516 | 30.6 | 20 | 0 | United State | Human |
| PE009_ip3 | 19684 | 33.3 | 23 | 0 | United State | Human |
| QR502_ip2 | 16781 | 31.7 | 20 | 0 | China | Human |
| QR502_ip3 | 6125 | 27.1 | 7 | 0 | China | Human |
| QR502_ip4 | 35030 | 31.1 | 46 | 0 | China | Human |
| R0294_ip1 | 11394 | 31.6 | 20 | 0 | China | Other |
| R0294_ip2 | 10053 | 30.7 | 14 | 0 | China | Other |
| R0294_ip3 | 6760 | 25.5 | 9 | 0 | China | Other |
| R0294_ip4 | 21725 | 32.8 | 28 | 0 | China | Other |
| R0294_ip7 | 13736 | 35.7 | 17 | 0 | China | Other |
| R0294_ip8 | 9888 | 33.1 | 17 | 0 | China | Other |
| R0353_ip1 | 11395 | 31.6 | 20 | 0 | China | Other |
| R0353_ip3 | 18998 | 31.3 | 27 | 0 | China | Other |
| R0353_ip4 | 24821 | 35.3 | 29 | 0 | China | Other |
| R0353_ip5 | 6760 | 25.5 | 9 | 0 | China | Other |
| R0353_ip7 | 28993 | 32.9 | 30 | 0 | China | Other |
| R0615_ip1 | 13889 | 33.1 | 15 | 0 | China | Other |
| R0615_ip2 | 23139 | 31.6 | 30 | 0 | China | Other |
| R0615_ip3 | 28317 | 32.5 | 39 | 0 | China | Other |
| R0615_ip4 | 9321 | 31.9 | 15 | 0 | China | Other |
| R0615_ip5 | 6760 | 25.5 | 9 | 0 | China | Other |
| R0615_ip6 | 22815 | 34.9 | 29 | 0 | China | Other |
| RA3_ip1 | 7022 | 26.1 | 14 | 0 | Argentina | Human |
| RA3_ip2 | 19684 | 33.3 | 23 | 0 | Argentina | Human |
| RA3_ip3 | 9804 | 32.4 | 10 | 0 | Argentina | Human |
| RA3_ip6 | 22202 | 34.1 | 27 | 0 | Argentina | Human |
| RA3_ip7 | 20348 | 30.7 | 25 | 0 | Argentina | Human |
| RA3_ip8 | 23356 | 32 | 33 | 0 | Argentina | Human |
| Rd_3_ip1 | 16412 | 32 | 16 | 0 | Germany | Bovine |
| RF122_ip1 | 10868 | 31.9 | 14 | 0 | Ireland | Bovine |
| RF122_ip2 | 9236 | 31.8 | 9 | 0 | Ireland | Bovine |
| RF122_ip3 | 55566 | 33.1 | 77 | 0 | Ireland | Bovine |
| RH_0600_0125_09_ip1 | 17259 | 30.5 | 18 | 0 | United Kingdom | Human |
| RH_0600_0125_09_ip3 | 7733 | 29.6 | 8 | 0 | United Kingdom | Human |
| RIVM1295_ip1 | 6765 | 25.5 | 9 | 0 | Netherlands | Human |
| RIVM1607_ip1 | 16412 | 32 | 16 | 0 | Netherlands | Human |
| RIVM1607_ip2 | 6760 | 25.5 | 9 | 0 | Netherlands | Human |
| RIVM3897_ip2 | 6760 | 25.5 | 9 | 0 | Netherlands | Human |
| 08BA02176_ip2 | 16412 | 32 | 16 | 0 | Canada | Human |
| 08BA02176_ip3 | 7785 | 26.5 | 10 | 0 | Canada | Human |
| RKI4_ip1 | 35951 | 30.4 | 50 | 0 | Germeny | Human |
| RKI4_ip2 | 21007 | 30.1 | 25 | 0 | Germeny | Human |
| S54F9_ip2 | 7188 | 27.5 | 18 | 0 | Denmark | Swine |
| S54F9_ip3 | 17941 | 29.1 | 21 | 0 | Denmark | Swine |
| S56_POEL_ip1 | 16412 | 32 | 16 | 0 | Belgium | Chicken |
| S56_POEL_ip3 | 6760 | 25.5 | 9 | 0 | Belgium | Chicken |
| S56_POEL_ip4 | 16049 | 32.3 | 19 | 0 | Belgium | Chicken |
| S56_POEL_ip6 | 9810 | 34.4 | 25 | 0 | Belgium | Chicken |
| S56_POEL_ip7 | 9454 | 33.2 | 16 | 0 | Belgium | Chicken |
| S0385_ip2 | 16412 | 32 | 16 | 0 | Australia | Human |
| S0385_ip3 | 6760 | 25.5 | 9 | 0 | Australia | Human |
| S2396_ip1 | 17626 | 32.4 | 25 | 0 | France | Human |
| S2396_ip10 | 34308 | 31.6 | 40 | 0 | France | Human |
| S2396_ip11 | 19510 | 30.6 | 20 | 0 | France | Human |
| S2396_ip12 | 16756 | 31.6 | 27 | 0 | France | Human |
| S2396_ip2 | 13277 | 31.4 | 14 | 0 | France | Human |
| S2396_ip3 | 25114 | 32.6 | 36 | 0 | France | Human |
| S2396_ip6 | 18801 | 35.9 | 21 | 0 | France | Human |
| S2396_ip8 | 14956 | 32.7 | 21 | 0 | France | Human |
| S2396_ip9 | 12256 | 32.9 | 17 | 0 | France | Human |
| S2397_ip1 | 20297 | 33 | 37 | 0 | France | Human |
| S2397_ip2 | 12782 | 30.7 | 15 | 0 | France | Human |
| S2397_ip3 | 17504 | 34.3 | 29 | 0 | France | Human |
| S2397_ip4 | 16947 | 30.9 | 24 | 0 | France | Human |
| S2397_ip5 | 20927 | 29.9 | 28 | 1 | France | Human |
| S2398_ip1 | 13970 | 27.8 | 19 | 0 | France | Human |
| S2398_ip3 | 6272 | 30.1 | 11 | 0 | France | Human |
| S2398_ip4 | 11368 | 33.7 | 23 | 0 | France | Human |
| S2398_ip5 | 17725 | 31.4 | 22 | 0 | France | Human |
| S2398_ip7 | 18358 | 32.5 | 26 | 0 | France | Human |
| SA_067_ip1 | 9340 | 31.5 | 18 | 0 | Switzerland | Other |
| SA_067_ip2 | 19456 | 33.4 | 23 | 0 | Switzerland | Other |
| SA_083_ip1 | 17911 | 30.6 | 18 | 0 | Switzerland | Other |
| SA_083_ip4 | 19672 | 33.4 | 24 | 0 | Switzerland | Other |
| SA_085_ip1 | 11921 | 32.8 | 13 | 0 | Switzerland | Other |
| 09_01244_ip2 | 29594 | 30.9 | 33 | 0 | Singapore | Human |
| 09_01244_ip3 | 9342 | 26.5 | 11 | 0 | Singapore | Human |
| SA_120_ip1 | 19843 | 32.8 | 35 | 0 | Switzerland | Other |
| SA_120_ip2 | 27278 | 32.1 | 37 | 0 | Switzerland | Other |
| SA_120_ip3 | 27141 | 33.8 | 30 | 0 | Switzerland | Other |
| SA_210_ip2 | 19684 | 33.3 | 23 | 0 | Switzerland | Other |
| SA_210_ip3 | 7022 | 26.1 | 14 | 0 | Switzerland | Other |
| SA_210_ip4 | 16515 | 30.6 | 20 | 0 | Switzerland | Other |
| SA_260_ip2 | 26792 | 34.6 | 42 | 0 | Switzerland | Other |
| SA_260_ip3 | 28706 | 34.4 | 47 | 0 | Switzerland | Other |
| SA_260_ip4 | 11835 | 27.4 | 16 | 0 | Switzerland | Other |
| SA_260_ip5 | 7190 | 27.5 | 18 | 0 | Switzerland | Other |
| SA_260_ip6 | 7999 | 33.1 | 13 | 0 | Switzerland | Other |
| SA_ST125_MupR_ip1 | 18830 | 27.7 | 21 | 0 | Spain | Human |
| SA_ST125_MupR_ip2 | 13529 | 30.8 | 16 | 0 | Spain | Human |
| SA_ST125_MupR_ip3 | 31893 | 32.8 | 37 | 0 | Spain | Human |
| SA_ST125_MupR_ip4 | 65667 | 33.7 | 92 | 0 | Spain | Human |
| SA3_LAU_ip1 | 17094 | 33.5 | 33 | 0 | Lebanon | Human |
| SA3_LAU_ip3 | 28267 | 33.9 | 37 | 0 | Lebanon | Human |
| SA3_LAU_ip4 | 22795 | 33.2 | 33 | 0 | Lebanon | Human |
| SA3_LAU_ip5 | 17609 | 32 | 25 | 0 | Lebanon | Human |
| SA3_LAU_ip6 | 9053 | 29.9 | 16 | 0 | Lebanon | Human |
| SA8_LAU_ip1 | 15835 | 30 | 30 | 0 | Jordan | Human |
| SA8_LAU_ip2 | 27814 | 33.1 | 36 | 0 | Jordan | Human |
| SA8_LAU_ip3 | 16354 | 31.8 | 18 | 0 | Jordan | Human |
| SA8_LAU_ip4 | 30188 | 34.3 | 55 | 0 | Jordan | Human |
| SA11_LAU_ip1 | 12199 | 27.3 | 16 | 0 | Jordan | Human |
| SA11_LAU_ip2 | 31715 | 34.2 | 46 | 0 | Jordan | Human |
| SA11_LAU_ip3 | 7188 | 27.5 | 18 | 0 | Jordan | Human |
| SA11_LAU_ip5 | 29159 | 31.9 | 39 | 0 | Jordan | Human |
| Sa12_001_ip2 | 10868 | 31.9 | 14 | 0 | Australia | Bovine |
| Sa12_001_ip3 | 9236 | 31.8 | 9 | 0 | Australia | Bovine |
| Sa12_001_ip4 | 12191 | 26.7 | 12 | 0 | Australia | Bovine |
| Sa12_002_ip1 | 7061 | 33.3 | 7 | 0 | Australia | Bovine |
| Sa12_002_ip2 | 25335 | 30.9 | 31 | 0 | Australia | Bovine |
| Sa12_002_ip3 | 7877 | 30.1 | 8 | 0 | Australia | Bovine |
| Sa12_002_ip4 | 9866 | 31.9 | 14 | 0 | Australia | Bovine |
| Sa13_005_ip1 | 11029 | 31.6 | 18 | 2 | Australian | Sheep |
| Sa13_005_ip3 | 18299 | 31.1 | 22 | 0 | Australian | Sheep |
| Sa13_005_ip5 | 6126 | 28.6 | 7 | 0 | Australian | Sheep |
| 09_01245_ip1 | 29906 | 32.2 | 43 | 0 | Singapore | Human |
| 09_01245_ip2 | 15641 | 30.4 | 17 | 0 | Singapore | Human |
| 09_01245_ip3 | 33746 | 31.1 | 49 | 0 | Singapore | Human |
| 09_01245_ip4 | 8473 | 30.5 | 15 | 0 | Singapore | Human |
| 09_01245_ip5 | 9390 | 26 | 11 | 0 | Singapore | Human |
| 09_01245_ip7 | 24706 | 34.4 | 32 | 0 | Singapore | Human |
| Sa13_006_ip1 | 28740 | 33.8 | 30 | 0 | Australian | Bovine |
| Sa13_006_ip3 | 16167 | 31.3 | 18 | 0 | Australian | Bovine |
| Sa13_006_ip5 | 6270 | 32.1 | 10 | 0 | Australian | Bovine |
| Sa14_002_ip1 | 7021 | 26.1 | 14 | 0 | Australia | Bovine |
| Sa14_002_ip3 | 19684 | 33.3 | 23 | 0 | Australia | Bovine |
| Sa14_003_ip2 | 18831 | 27.7 | 21 | 0 | Australia | Bovine |
| Sa14_003_ip3 | 22306 | 31 | 29 | 0 | Australia | Bovine |
| Sa14_004_ip1 | 8656 | 32.6 | 12 | 0 | Australia | Bovine |
| Sa14_004_ip2 | 7784 | 34.2 | 9 | 0 | Australia | Bovine |
| SA40_ip1 | 11720 | 31.4 | 17 | 0 | China | Human |
| SA40_ip2 | 8156 | 26.7 | 16 | 0 | China | Human |
| SA40_ip3 | 7597 | 28.5 | 7 | 0 | China | Human |
| SA40TW_ip1 | 11719 | 31.4 | 16 | 0 | China | Human |
| SA268_ip1 | 11720 | 31.4 | 17 | 0 | China | Human |
| SA268_ip2 | 8159 | 26.7 | 16 | 0 | China | Human |
| SA268_ip4 | 8314 | 28.3 | 10 | 0 | China | Human |
| SA957_ip1 | 11720 | 31.4 | 17 | 0 | China | Human |
| SA957_ip2 | 8158 | 26.7 | 16 | 0 | China | Human |
| SA957_ip4 | 8315 | 28.3 | 9 | 0 | China | Human |
| SAHPchr_ip1 | 15684 | 31.4 | 19 | 0 | China | Swine |
| SAHPchr_ip3 | 9086 | 26.2 | 10 | 0 | China | Swine |
| SAHPchr_ip4 | 21299 | 30 | 34 | 0 | China | Swine |
| SAHPchr_ip5 | 7223 | 28.6 | 8 | 0 | China | Swine |
| SAHPchr_ip6 | 16755 | 31.7 | 20 | 0 | China | Swine |
| SARM_C5621_ip1 | 6760 | 25.5 | 9 | 0 | Spain | Human |
| SARM_C5621_ip3 | 28023 | 32.6 | 29 | 0 | Spain | Human |
| 9_ip1 | 6682 | 26.4 | 11 | 0 | Finland | Bovine |
| SCCmec1_ip1 | 12199 | 27.3 | 16 | 0 | Denmark | Human |
| SCCmec1_ip3 | 26459 | 31.3 | 34 | 0 | Denmark | Human |
| SCCmec1_ip7 | 9515 | 32 | 10 | 0 | Denmark | Human |
| SR411_ip1 | 6760 | 25.5 | 9 | 0 | China | Human |
| SR434_ip1 | 9449 | 32.2 | 14 | 0 | China | Human |
| SR434_ip2 | 8196 | 25.6 | 16 | 0 | China | Human |
| SR434_ip3 | 15631 | 30.5 | 20 | 0 | China | Human |
| st424_ip1 | 18870 | 30 | 32 | 0 | United Kingdom | Human |
| st424_ip2 | 6111 | 26.3 | 7 | 0 | United Kingdom | Human |
| st436_ip1 | 18850 | 30 | 30 | 0 | United Kingdom | Human |
| st436_ip3 | 24113 | 33.8 | 30 | 0 | United Kingdom | Human |
| st436_ip4 | 26273 | 33.2 | 31 | 0 | United Kingdom | Human |
| st436_ip5 | 29301 | 32 | 40 | 0 | United Kingdom | Human |
| st534_ip1 | 15787 | 29.9 | 29 | 0 | Ireland | Human |
| st534_ip3 | 19598 | 31.1 | 26 | 0 | Ireland | Human |
| ST772_MRSA_V_ip1 | 29954 | 32.6 | 34 | 0 | Australia | Human |
| st1332_ip1 | 15589 | 29.7 | 23 | 0 | United Kingdom | Human |
| st1332_ip2 | 16289 | 30.5 | 20 | 0 | United Kingdom | Human |
| st1335_ip2 | 14344 | 32.3 | 21 | 0 | United Kingdom | Human |
| st1335_ip3 | 12199 | 27.3 | 16 | 0 | United Kingdom | Human |
| st1335_ip4 | 7188 | 27.5 | 18 | 0 | United Kingdom | Human |
| st1335_ip5 | 10040 | 31.9 | 10 | 0 | United Kingdom | Human |
| 10S_ip2 | 27360 | 34.4 | 43 | 0 | Pakistan | Human |
| 10S_ip3 | 17567 | 35.9 | 37 | 0 | Pakistan | Human |
| 10S_ip4 | 14234 | 31.4 | 18 | 0 | Pakistan | Human |
| st1424_ip2 | 18831 | 27.7 | 21 | 0 | United Kingdom | Human |
| st1520_ip1 | 6683 | 26.4 | 11 | 0 | United Kingdom | Human |
| st1520_ip3 | 28408 | 33.3 | 36 | 0 | United Kingdom | Human |
| st1520_ip4 | 18509 | 32.3 | 34 | 0 | United Kingdom | Human |
| st1607_ip2 | 12199 | 27.3 | 16 | 0 | Ireland | Human |
| st1607_ip4 | 7188 | 27.5 | 18 | 0 | Ireland | Human |
| st1607_ip5 | 22718 | 34.1 | 24 | 0 | Ireland | Human |
| st1624_ip1 | 16847 | 30.5 | 19 | 0 | United Kingdom | Human |
| st1815_ip1 | 20192 | 28.5 | 21 | 7 | Ireland | Human |
| st1815_ip3 | 19810 | 33 | 21 | 0 | Ireland | Human |
| st1815_ip4 | 31836 | 32.8 | 37 | 0 | Ireland | Human |
| st1831_ip1 | 17579 | 30.9 | 18 | 0 | Ireland | Human |
| st1831_ip2 | 24222 | 31.2 | 37 | 0 | Ireland | Human |
| st1831_ip3 | 17291 | 33.8 | 34 | 0 | Ireland | Human |
| st1831_ip4 | 16551 | 33.5 | 26 | 0 | Ireland | Human |
| st1831_ip5 | 14507 | 32 | 25 | 0 | Ireland | Human |
| st2436_ip1 | 15811 | 30 | 30 | 0 | United Kingdom | Human |
| st2436_ip2 | 22520 | 33.2 | 26 | 0 | United Kingdom | Human |
| st2436_ip4 | 33214 | 32.4 | 54 | 0 | United Kingdom | Human |
| st2436_ip5 | 10039 | 32 | 10 | 0 | United Kingdom | Human |
| st2534_ip2 | 12199 | 27.3 | 16 | 0 | United Kingdom | Human |
| st2534_ip3 | 12172 | 32.2 | 18 | 0 | United Kingdom | Human |
| st2534_ip5 | 7188 | 27.5 | 18 | 0 | United Kingdom | Human |
| st2534_ip6 | 28003 | 33.2 | 33 | 0 | United Kingdom | Human |
| st2534_ip7 | 10040 | 31.9 | 10 | 0 | United Kingdom | Human |
| st2534_ip8 | 22337 | 30.5 | 31 | 0 | United Kingdom | Human |
| st2543_ip3 | 10040 | 31.9 | 10 | 0 | United Kingdom | Human |
| st2543_ip4 | 5342 | 27.5 | 13 | 0 | United Kingdom | Human |
| st2761_ip1 | 18828 | 27.7 | 22 | 0 | United Kingdom | Human |
| st2761_ip2 | 18915 | 31.6 | 23 | 0 | United Kingdom | Human |
| st2787_ip2 | 31987 | 31.1 | 36 | 0 | United Kingdom | Human |
| st2787_ip3 | 20820 | 32.5 | 38 | 0 | United Kingdom | Human |
| st3046_ip1 | 9804 | 32.5 | 10 | 0 | United Kingdom | Human |
| st3046_ip2 | 19684 | 33.3 | 23 | 0 | United Kingdom | Human |
| st3046_ip3 | 28258 | 33.3 | 35 | 0 | United Kingdom | Human |
| st3046_ip4 | 69417 | 31.2 | 58 | 0 | United Kingdom | Human |
| st3046_ip5 | 29617 | 31 | 44 | 0 | United Kingdom | Human |
| st3046_ip6 | 21479 | 30.7 | 25 | 0 | United Kingdom | Human |
| st3046_ip7 | 29732 | 28.8 | 56 | 0 | United Kingdom | Human |
| st3046_ip8 | 4689 | 34.3 | 8 | 0 | United Kingdom | Human |
| st3046_ip9 | 4527 | 34.6 | 7 | 0 | United Kingdom | Human |
| st3047_ip1 | 8159 | 26.7 | 16 | 0 | United Kingdom | Human |
| T0131_ip1 | 9803 | 32.3 | 10 | 0 | China | Human |
| T0131_ip2 | 5981 | 30 | 7 | 0 | China | Human |
| T0131_ip3 | 8354 | 27.7 | 15 | 0 | China | Human |
| T0131_ip5 | 24666 | 30.5 | 32 | 0 | China | Human |
| T44444_ip2 | 22644 | 32.9 | 24 | 0 | United State | Human |
| T44444_ip3 | 13962 | 27.8 | 18 | 0 | United State | Human |
| T59618_ip2 | 18831 | 27.7 | 21 | 0 | United State | Human |
| Tager_104_ip2 | 17057 | 30.3 | 16 | 0 | United State | Human |
| TCH60_ip1 | 24512 | 32.8 | 24 | 0 | United State | Human |
| TCH60_ip2 | 17233 | 29.1 | 22 | 0 | United State | Human |
| TSAR01_ip1 | 35992 | 30.4 | 50 | 0 | China | Human |
| TSAR01_ip3 | 36699 | 31.4 | 52 | 0 | China | Human |
| TSAR01_ip4 | 26715 | 33.4 | 32 | 0 | China | Human |
| TSAR02_ip2 | 16782 | 31.7 | 20 | 0 | China | Human |
| TSAR02_ip3 | 21299 | 30.1 | 34 | 0 | China | Human |
| TSAR03_ip1 | 35030 | 31.1 | 46 | 0 | China | Human |
| TSAR04_ip1 | 21299 | 30.1 | 34 | 0 | China | Human |
| TSAR04_ip2 | 6125 | 27.1 | 7 | 0 | China | Human |
| TSAR05_ip3 | 29118 | 32.2 | 40 | 0 | China | Human |
| TSAR05_ip4 | 36543 | 30.3 | 52 | 0 | China | Human |
| TSAR05_ip5 | 27432 | 30.9 | 39 | 0 | China | Human |
| TSAR05_ip6 | 29226 | 33.3 | 39 | 0 | China | Human |
| TSAR05_ip7 | 15664 | 34 | 31 | 0 | China | Human |
| TSAR06_ip1 | 21299 | 30.1 | 34 | 0 | China | Human |
| TSAR07_ip1 | 16643 | 30.3 | 29 | 0 | China | Human |
| TSAR07_ip2 | 27670 | 33.4 | 56 | 0 | China | Human |
| TSAR08_ip1 | 21299 | 30.1 | 34 | 0 | China | Human |
| Tur_15_ip1 | 6760 | 25.5 | 8 | 0 | Austria | Chicken |
| Tur_15_ip2 | 17869 | 34 | 38 | 0 | Austria | Chicken |
| Tur_15_ip4 | 16255 | 33.7 | 33 | 0 | Austria | Chicken |
| Tur_20_ip1 | 6761 | 25.4 | 9 | 0 | Germany | Chicken |
| Tur_22_ip1 | 6760 | 25.5 | 9 | 0 | Italy | Chicken |
| Tur_22_ip3 | 23244 | 34.5 | 33 | 0 | Italy | Chicken |
| TW20_ip1 | 8647 | 33 | 11 | 0 | United Kingdom | Unknown |
| TW20_ip3 | 19684 | 33.3 | 23 | 0 | United Kingdom | Unknown |
| TW20_ip4 | 9804 | 32.5 | 10 | 0 | United Kingdom | Unknown |
| TW20_ip5 | 8353 | 27.7 | 15 | 0 | United Kingdom | Unknown |
| TW20_ip8 | 7065 | 32.1 | 10 | 0 | United Kingdom | Unknown |
| UAMS_1_ip1 | 12219 | 27.3 | 16 | 0 | United State | Human |
| UAMS_1_ip3 | 26501 | 31.1 | 34 | 0 | United State | Human |
| USA_1_ip1 | 6760 | 25.5 | 9 | 0 | United State | Human |
| USA_1_ip3 | 16412 | 32 | 16 | 0 | United State | Human |
| USA_1_ip4 | 36170 | 33.2 | 50 | 0 | United State | Human |
| USA_15_ip1 | 6760 | 25.5 | 9 | 0 | United State | Human |
| USA300_2014.C01_ip2 | 7015 | 26.1 | 14 | 0 | United State | Human |
| USA300_2014.C01_ip3 | 16516 | 30.6 | 20 | 0 | United State | Human |
| USA300_2014.C01_ip4 | 19683 | 33.3 | 23 | 0 | United State | Human |
| USA300_2014.C02_ip1 | 19684 | 33.3 | 23 | 0 | United State | Human |
| USA300_2014.C02_ip4 | 7020 | 26.1 | 14 | 0 | United State | Human |
| USA300_FPR3757_ip1 | 19684 | 33.3 | 23 | 0 | United State | Human |
| USA300_FPR3757_ip2 | 20640 | 30.8 | 24 | 0 | United State | Human |
| USA300_FPR3757_ip3 | 7022 | 26.1 | 14 | 0 | United State | Human |
| USA300_TCH959_ip3 | 7796 | 26.5 | 15 | 0 | United State | Human |
| USA300_TCH959_ip4 | 14256 | 30.7 | 15 | 0 | United State | Human |
| USA300_TCH1516_ip1 | 19684 | 33.3 | 23 | 0 | United State | Human |
| USA300_TCH1516_ip2 | 20640 | 30.8 | 24 | 0 | United State | Human |
| USA300_TCH1516_ip3 | 7022 | 26.1 | 14 | 0 | United State | Human |
| USFL022_ip2 | 16820 | 33.7 | 35 | 0 | United State | Human |
| USFL022_ip3 | 7022 | 26.1 | 14 | 0 | United State | Human |
| USFL022_ip5 | 19684 | 33.3 | 23 | 0 | United State | Human |
| USFL022_ip6 | 49315 | 32.2 | 59 | 0 | United State | Human |
| USFL190_ip2 | 7021 | 26 | 14 | 0 | United State | Human |
| USFL246_ip1 | 7022 | 26.1 | 14 | 0 | United State | Human |
| USFL246_ip2 | 22342 | 31.2 | 29 | 0 | United State | Human |
| USFL246_ip4 | 20388 | 33.2 | 24 | 0 | United State | Human |
| USFL253_ip1 | 19684 | 33.3 | 23 | 0 | United State | Human |
| USFL253_ip2 | 7022 | 26.1 | 14 | 0 | United State | Human |
| USFL253_ip3 | 26026 | 33 | 35 | 0 | United State | Human |
| USFL253_ip4 | 23598 | 33.2 | 44 | 0 | United State | Human |
| UTSW_MRSA_55_ip1 | 7022 | 26.1 | 14 | 0 | United State | Human |
| UTSW_MRSA_55_ip4 | 19684 | 33.3 | 23 | 0 | United State | Human |
| UTSW_MRSA_55_ip5 | 20640 | 30.8 | 24 | 0 | United State | Human |
| V808_ip2 | 19684 | 33.3 | 23 | 0 | Korea | Human |
| V808_ip3 | 9804 | 32.5 | 10 | 0 | Korea | Human |
| V808_ip4 | 27503 | 33.2 | 33 | 0 | Korea | Human |
| V808_ip5 | 22290 | 30.6 | 30 | 0 | Korea | Human |
| V808_ip6 | 39528 | 31 | 53 | 0 | Korea | Human |
| V808_ip7 | 69407 | 31.2 | 58 | 0 | Korea | Human |
| V808_ip8 | 31185 | 29 | 58 | 0 | Korea | Human |
| V1127_ip1 | 22718 | 34.1 | 23 | 0 | Korea | Human |
| V1127_ip2 | 25569 | 32.5 | 38 | 0 | Korea | Human |
| V1127_ip3 | 22265 | 31.6 | 27 | 0 | Korea | Human |
| V1127_ip4 | 18830 | 27.7 | 21 | 0 | Korea | Human |
| V1127_ip5 | 19972 | 30.9 | 26 | 0 | Korea | Human |
| V1127_ip6 | 1396 | 32.9 | 6 | 0 | Korea | Human |
| V1127_ip7 | 9999 | 34.4 | 24 | 0 | Korea | Human |
| V1142_ip1 | 24717 | 32.5 | 34 | 0 | Korea | Human |
| V1859_ip1 | 19684 | 33.3 | 23 | 0 | Venezuela | Human |
| V1859_ip2 | 20690 | 30.8 | 25 | 0 | Venezuela | Human |
| V1859_ip3 | 7022 | 26.1 | 14 | 0 | Venezuela | Human |
| VB26276_ip1 | 28771 | 32.8 | 34 | 0 | India | Human |
| VB26276_ip3 | 24621 | 34.3 | 35 | 0 | India | Human |
| VB26276_ip4 | 18376 | 31.4 | 30 | 0 | India | Human |
| VC40_ip1 | 19684 | 33.3 | 23 | 0 | Germany | Other |
| VC40_ip2 | 7023 | 26.1 | 14 | 0 | Germany | Other |
| VET0051R_ip2 | 6760 | 25.5 | 9 | 0 | Netherlands | Human |
| VET0889S_ip3 | 5172 | 27.8 | 12 | 0 | Netherlands | Human |
| VET0889S_ip5 | 12199 | 27.3 | 16 | 0 | Netherlands | Human |
| VET1518S_ip2 | 5172 | 27.8 | 12 | 0 | Netherlands | Human |
| VET1518S_ip3 | 12199 | 27.3 | 16 | 0 | Netherlands | Human |
| VH221_ip1 | 27322 | 33.5 | 36 | 0 | India | Human |
| VH221_ip2 | 19691 | 33.5 | 23 | 0 | India | Human |
| VH221_ip3 | 22262 | 30.9 | 31 | 0 | India | Human |
| VH221_ip5 | 11492 | 33.5 | 23 | 0 | India | Human |
| W33563_ip1 | 35603 | 32.7 | 42 | 0 | United State | Human |
| W33563_ip4 | 13566 | 33.2 | 29 | 0 | United State | Human |
| W45755_111412_ip1 | 7061 | 33.3 | 7 | 0 | United State | Human |
| W45755_111412_ip10 | 4144 | 30.7 | 8 | 0 | United State | Human |
| W45755_111412_ip11 | 2668 | 35.7 | 7 | 0 | United State | Human |
| W45755_111412_ip12 | 3037 | 35.8 | 8 | 0 | United State | Human |
| W45755_111412_ip13 | 2147 | 33.3 | 6 | 0 | United State | Human |
| W45755_111412_ip2 | 9390 | 32.1 | 13 | 0 | United State | Human |
| W45755_111412_ip3 | 4540 | 32.3 | 6 | 0 | United State | Human |
| W45755_111412_ip4 | 13946 | 34.3 | 12 | 0 | United State | Human |
| W45755_111412_ip5 | 14083 | 35 | 13 | 0 | United State | Human |
| W45755_111412_ip7 | 4471 | 28.5 | 5 | 0 | United State | Human |
| W45755_111412_ip8 | 4863 | 33 | 8 | 0 | United State | Human |
| W45755_111412_ip9 | 4666 | 32.3 | 8 | 0 | United State | Human |
| W82303_ip1 | 18831 | 27.7 | 21 | 0 | United State | Human |
| W82303_ip2 | 16901 | 31.4 | 22 | 0 | United State | Human |
| W82303_ip4 | 7100 | 34.6 | 16 | 0 | United State | Human |
| XN108_ip1 | 8505 | 33.1 | 11 | 0 | China | Human |
| XN108_ip2 | 19683 | 33.3 | 23 | 0 | China | Human |
| XN108_ip3 | 9798 | 32.5 | 10 | 0 | China | Human |
| XN108_ip4 | 5981 | 29.9 | 7 | 0 | China | Human |
| XN108_ip8 | 7063 | 32.1 | 10 | 0 | China | Human |
| Z172_ip1 | 8649 | 32.9 | 11 | 0 | China | Human |
| Z172_ip2 | 19685 | 33.3 | 23 | 0 | China | Human |
| Z172_ip3 | 9804 | 32.5 | 10 | 0 | China | Human |
| Z172_ip5 | 5980 | 29.9 | 7 | 0 | China | Human |
| Z172_ip7 | 7065 | 32.1 | 10 | 0 | China | Human |
| ZJ5499_ip1 | 22719 | 34.1 | 23 | 0 | China | Human |
| ZJ5499_ip2 | 35200 | 33 | 40 | 0 | China | Human |
| ZJ5499_ip3 | 6113 | 28.6 | 7 | 0 | China | Human |
| ZJ5499_ip6 | 19972 | 30.9 | 25 | 0 | China | Human |
| **Outgroup** |  |  |  |  |  |  |
| Erwinia_phage_phiEa2809 (NC_027340.1) | |  |  |  |  |  |

Table S4. Summary of the gene content of functional modules present in the *S. aureus* intact prophages.

| **Groups** | **Phage morphogenesis** | **Host cell lysis** | **DNA metabolism** | **DNA packaging** | **Lysogeny** | **Virulence** | **Resistance** | **No. (%)** | **Represent Phage** |
| --- | --- | --- | --- | --- | --- | --- | --- | --- | --- |
| 1 | Phage tail tube protein; Phage head-tail joining protein; Phage portal protein | lysin | Type I restriction modification system; Single-strand binding protein; DNA binding protein | Terminase small subunit | Clp protease; Integrase | dUTPase; hlb; lukG; lukH; hld | - | 34 | 04_03111_p2 |
| 2 | Phage portal protein; Phage capsid protein; Phage tail protein | lysin (2) | - | DNA packaging protein (2) | - | dUTPase | - | 27 | 08142_8_p3 |
| 3 | Phage tail protein; Phage tail tube protein; Phage portal protein | lysin ; Holin | DNA binding protein; DNA polymerase | terminase small subunit | Clp protease; Integrase; Recombinase | Virulence-associated protein E; dUTPase; lukH; lukG; hlb; scn; sak | - | 27 | CA12_p3 |
| 4 | Phage portal protein; Phage head-tail connector protein; Phage head-tail joining protein; Phage tail protein | lysin | DNA binding protein (3); Single-strand binding protein | Phage terminase small subunit | Integrase; Recombinase | lukH; lukG; hlb; scn; sak; dUTPase; | - | 24 | 147_SAUR_p2 |
| 5 | Phage portal protein; Phage tail tube protein; Phage tail protein | lysin ; Holin | DNA binding protein (2); DNA polymerase | terminase small subunit | Integrase; Clp protease; | Virulence-associated protein E | - | 21 | 08BA02176_p1 |
| 6 | - | - | - | Terminase small subunit | Integrase | seb | - | 18 | 93b_S9_p2 |
| 7 | Phage portal protein; Phage capsid protein; Phage head-tail joining protein; Phage tail tube protein; Phage tail tape measure protein; Phage tail protein | lysin (3) | DNA binding protein; Single-strand binding protein | Terminase small subunit | - | dUTPase; lukH; lukG; hlb; scn; sak; chp | - | 17 | MRSA_S1_p5 |
| 8 | Phage portal protein; Phage capsid protein; Phage head-tail joining protein; Phage tail tube protein; Phage tail protein | lysin | Single-strand binding protein; DNA binding protein | DNA packaging protein | Integrase | dUTPase; lukH; lukG; hlb; scn; sak; chp; selp; eap/map | - | 17 | 107_p3 |
| 9 | Phage tail protein; Phage tail tube protein; Phage portal protein; | - | DNA binding protein; DNA polymerase | terminase small subunit | Clp protease; Integrase; Recombinase | Virulence-associated protein E; dUTPase | - | 17 | 03_01478_p1 |
| 10 | Phage portal protein; Phage head-tail connector protein; Phage tail assembly chaperone protein; Phage tail protein | lysin (2); Holin | DNA binding protein (3); Single-strand binding protein | Terminase small subunit; terminase large subunit; | Transposase | dUTPase; PVL | - | 15 | 04_02981_p2 |
| 11 | Phage tail protein (2); Phage tail tube protein; Phage head-tail joining protein; Phage capsid protein; Phage portal protein | lysin (2) | Single-strand binding protein; DNA binding protein | DNA packaging protein | Transposase; Integrase | dUTPase; lukH; lukG; hlb; scn; sak; chp; eap/map | - | 14 | CIG1242_p6 |
| 12 | Phage tail protein; Phage tail tape measure protein; Phage tail tube protein; Phage head-tail joining protein; Phage capsid Phage portal protein | lysin | Single-strand binding protein | Terminase small subunit | Clp protease; Integrase | dUTPase; lukH; lukG; hlb; sak; eap/map; sea | - | 14 | Z172_p6 |
| 13 | Phage tail protein; Phage tail tube protein; Phage portal protein; | lysin; Holin | DNA helicase; DNA polymerase; DNA binding protein (2) | terminase small subunit | Clp protease; Integrase; recombinase | dUTPase; ebp; Virulence-associated protein E | - | 13 | 11P4_p1 |
| 14 | Phage tail protein; Phage tail tape measure protein; Phage tail assembly chaperone protein; Phage capsid protein; Phage portal protein | lysin (2); Holin | Single-strand binding protein; DNA binding protein (2) | DNA packaging protein (2) | - | dUTPase; eta | - | 12 | 08_02119_p3 |
| 15 | Phage portal protein; Phage capsid family; Phage head-tail joining protein; Phage tail tube protein; Phage tail protein | holin | DNA helicase | DNA packaging protein | recombinase (2); integrase; Clp protease | lukS-PV; lukF-PV; dUTPase; sea | - | 12 | 118_p2 |
| 16 | Phage tail protein; Phage tail tape measure protein | lysin | - | - | - | - | - | 10 | MRGR3_p8 |
| 17 | Phage portal protein; Phage head-tail connector protein; Phage tail assembly chaperone protein; Phage tail protein | lysin | sequence-specific DNA binding; DNA binding protein (3) | Terminase small subunit | Integrase | dUTPase; lukE; lukD; splA; splB; splC; splD; splE; splF | - | 10 | 82_SAUR_p1 |
| 18 | Phage tail protein; Phage tail tube protein; Phage portal protein | holin | DNA helicase; DNA polymerase; DNA binding protein | terminase small subunit | Clp protease; Integrase; recombinase | Virulence-associated protein E; dUTPase; | - | 10 | DAR3581_p2 |
| 19 | Phage tail protein; Phage tail tube protein; Phage portal protein | lysin; Holin | DNA polymerase | terminase small subunit | Clp protease; Integrase; Recombinase | Virulence-associated protein E; dUTPase | - | 9 | CIG1242_p4 |
| 20 | Phage portal protein; Phage head-tail connector protein; Phage tail assembly chaperone protein; Phage tail protein | lysin | sequence-specific DNA binding; DNA binding protein (4) | Terminase small subunit | - | dUTPase | - | 8 | st3047_p3 |
| 21 | Phage portal protein; Phagehead-tail connector protein; Phage tail assembly chaperone protein; Phage tail protein | lysin (2); Holin | - | Terminase small subunit; terminase large subunit; | - | - | - | 8 | S2398_p6 |
| 22 | Phage portal protein; Phage tail tube protein; Phage tail protein | - | DNA binding protein (2); DNA polymerase | terminase small subunit | Integrase; Clp protease | dUTPase; geh; Virulence-associated protein E | - | 8 | 2010_60_6511_39_p1 |
| 23 | Phage portal protein; Phage tail tube protein; Phage tail protein | lysin; Holin | DNA binding protein (2); DNA polymerase | terminase small subunit | Integrase; Clp protease | dUTPase; lukS-PV; lukF-PV; Virulence-associated protein E | dfrG | 8 | 04Hi_p1 |
| 24 | Phage tail protein; Phage tail tube protein; Phage head-tail joining protein; Phage capsid protein; Phage portal protein | holin | DNA binding protein (3) | DNA packaging protein | recombinase; integrase | hlb; lukG; lukH; dUTPase; | - | 7 | GKP136_64_p2 |
| 25 | Phage portal protein; Phage head-tail connector protein; Phage tail assembly chaperone protein; Phage tail protein | lysin | RNA methyltransferase | Terminase small subunit | - | dUTPase | - | 7 | 3957_p4 |
| 26 | Phage portal protein; Phage capsid protein; Phage head-tail joining protein; Phage tail tube protein; Phage tail tape measure protein; Phage tail protein | lysin | DNA binding protein | DNA packaging protein | Integrase; Recombinase | dUTPase; lukH; lukG; hlb; sak; chp; scn; eap/map | - | 6 | 92_p6 |
| 27 | Phage tail protein; Phage tail assembly chaperone protein; Phage major tail protein; Phage head-tail connector protein; Phage portal protein | lysin | DNA helicase; Single-strand binding protein; DNA binding protein (2) | terminase large subunit; Terminase small subunit | integrase | dUTPase | - | 6 | 120_p1 |
| 28 | Phage tail protein; Phage tail assembly chaperone protein; Phage head-tail connector protein; Phage portal protein | lysin | DNA binding protein | Terminase small subunit; terminase large subunit | - | dUTPase; | - | 6 | IF6SW_P2_p2 |
| 29 | Phage portal protein; Phage tail tube protein; Phage tail protein | lysin; Holin | - | terminase small subunit | Clp protease | PVL; Virulence-associated protein E | - | 6 | 2010_60_6511_10_p4 |
| 30 | Phage tail protein; Phage tail tube protein; Phage portal protein | - | DNA binding protein | terminase small subunit | Clp protease | Virulence-associated protein E | - | 6 | 2011_60_2275_1_p4 |
| 31 | Phage portal protein; Phage tail tube protein; Phage tail protein | lysin ; Holin | DNA binding protein (2); DNA polymerase | terminase small subunit | Recombinase; Integrase; Clp protease | dUTPase; ebp; Virulence-associated protein E | - | 6 | 08_02119_p2 |
| 32 | Phage portal protein; Phage head-tail connector protein; Phage head-tail joining protein; Phage tail protein | lysin | DNA binding protein; Single-strand binding protein; | Phage terminase small subunit (2) | Integrase; Recombinase | lukH; lukG; hlb; selk; selq; sea; sak; scn; eap/map | - | 5 | MUM475_p1 |
| 33 | Phage portal protein; Phage capsid protein; Phage tail assembly chaperone protein; Phage tail tape measure protein; Phage tail protein | - | - | DNA packaging (2) | - | - | - | 5 | DAR5877_p2 |
| 34 | Phage tail protein; Phage tail assembly chaperone protein; Phage head-tail connector protein; Phage portal protein | lysin | Single-strand binding protein; DNA binding protein (2) | Terminase small subunit; terminase large subunit | - | dUTPase | Arsenical Resistance | 5 | DAR3236_p5 |
| 35 | Phage portal protein; Phage tail tube protein; Phage tail protein | lysin ; Holin | - | terminase small subunit | Clp protease | Virulence-associated protein E | - | 5 | DAR5843_p1 |
| 36 | head morphogenesis protein (3); Phage portal protein; Phage tail tube protein; Phage tail protein | - | - | terminase small subunit | Clp protease | - | - | 5 | 07_03345_p2 |
| 37 | Phage portal protein; Phage head-tail connector protein; Phage tail assembly chaperone protein; Phage tail protein | lysin; Holin | - | Terminase small subunit | - | - | - | 4 | DAR5867_p2 |
| 38 | Phage tail protein; Phage tail assembly chaperone protein; Phage head-tail connector protein; Phage portal protein | lysin | Single-strand binding protein; DNA binding protein (2) | Terminase small subunit; terminase large subunit | - | dUTPase | - | 4 | BB155_p2 |
| 39 | Phage portal protein; Phage head-tail connector protein; Phage tail assembly chaperone protein; Phage tail protein | lysin | - | Terminase small subunit; terminase large subunit | - | - | - | 4 | DAR5843_p2 |
| 40 | Phage portal protein; Phage tail tube protein; Phage tail protein | holin | - | terminase small subunit | Clp protease | Virulence-associated protein E; dUTPase | - | 4 | MRSA08_p3 |
| 41 | Phage tail protein; Phage tail tube protein; Phage portal protein | - | DNA helicase; DNA binding protein (2); Single-strand binding protein | terminase small subunit | Clp protease; Integrase | Virulence-associated protein E (2); dUTPase | - | 4 | BA01611_p6 |
| 42 | Phage tail protein (2); Phage tail assembly chaperone protein; Phage tail-component; Phage head-tail connector protein; Phage portal protein | - | - | DNA packaging protein | - | - | - | 3 | H24_p5 |
| 43 | Phage tail protein; Phage tail assembly chaperone protein; Phage tail-component; Phage capsid protein; Phage portal protein | lysin | Single-strand binding protein; DNA binding protein (4) | DNA packaging protein (2) | - | dUTPase | - | 3 | CA_347_p7 |
| 44 | Phage tail protein (3); Phage minor structural protein | lysin | DNA primase; DNA helicase; RNA helicase; DNA-binding protein | - | Recombinase; Transposase (5); Integrase | - | AAC6_Ie_APH2_Ia; APH(3')-IIIa; aad(6); Bla | 3 | M48_p7 |
| 45 | Phage tail protein; Phage tail tube protein; Phage portal protein; | lysin | Type I restriction modification system; DNA polymerase; DNA binding protein | terminase small subunit | Clp protease | Virulence-associated protein E; dUTPase | - | 3 | BSAR748_p1 |
| 46 | Phage portal protein; Phage capsid protein; Bacteriophage tail-component; Phage tail assembly chaperone protein; Phage tail protein | lysin | Single-strand binding protein; DNA binding protein (3) | DNA packaging protein (2) | - | dUTPase | - | 2 | GD1539_p1 |
| 47 | Phage tail protein; Phage tail tape measure protein; Phage tail assembly chaperone protein; Phage tail-component; Phage capsid protein; Phage portal protein | lysin | DNA polymerase; DNA binding protein | DNA packaging protein (2) | integrase; recombinase | dUTPase | - | 2 | DAR5889_p3 |
| 48 | - | - | - | - | - | splA; splB; splC; lukD; lukE; seg; sei; sem; sen; yent1; yent2; | - | 2 | 4126_1_p1 |
| 49 | Phage tail protein; Phage tail tape measure protein; Phage tail assembly chaperone protein; Phage capsid protein; Phage portal protein | lysin (2) | DNA helicase; DNA binding protein (2) | DNA packaging protein (2) | - | dUTPase | FemAB | 2 | BU_G0201_t8_p3 |
| 50 | Phage portal protein; head morphogenesis protein; Phage head-tail connector protein; Phage tail-component; Phage tail tube protein; Phage tail assembly chaperone protein; Phage tail tape measure protein; Phage tail protein | - | DNA binding protein (3) | Terminase small subunit | integrase (2) | toxin-antitoxin module; dUTPase | aluminum resistance | 2 | BSAR58_p2 |
| 51 | Phage tail-component; Phage head-tail connector protein; Phage portal protein | - | - | DNA packaging protein | - | - | - | 2 | M35954_p2 |
| 52 | head-tail connector protein; Phage minor structural protein; Phage portal protein | - | - | DNA packaging protein | - | - | - | 2 | CUHK_HK2007_p1 |
| 53 | - | - | - | - | Integrase (2) | - | - | 2 | O46_p2 |
| 54 | head morphogenesis; tail tape measure protein; capsid protein; portal protein | holin | - | - | Transposase; Clp protease | dUTPase; | - | 2 | BU_N22_t6_p2 |
| 55 | Phage tail protein; Phage head-tail joining protein; Phage capsid protein; Phage portal protein | holin; recombinase | - | DNA packaging protein | Clp protease | lukF-PV; lukS-PV; dUTPase; sea | - | 2 | 3957_p5 |
| 56 | Phage portal protein; Phage capsid protein; Phage tail-component; Phage tail assembly chaperone protein; Phage tail tape measure protein; Phage tail protein | - | - | DNA packaging protein (2) | - | - | - | 2 | ES26_p7 |
| 57 | Phage portal protein; Phage tail tube protein; Phage tail protein | - | - | terminase small subunit | Clp protease | dUTPase; Virulence-associated protein E | - | 2 | GKP136_8_p2 |
| 58 | Phage tail protein; Phage tail tube protein; Phage portal protein | Holin | DNA polymerase | terminase small subunit | Clp protease; Integrase; recombinase | Virulence-associated protein E; dUTPase | - | 2 | M0396_p2 |
| 59 | Phage tail protein; Phage tail tube protein; Phage portal protein | lysin | - | terminase small subunit | Clp protease | dUTPase | - | 2 | FRI137_p1 |
| 60 | head morphogenesis protein; Phage tail protein; Phage tail tube protein; Phage portal protein | - | - | terminase small subunit | Clp protease | Virulence-associated protein E; dUTPase | - | 2 | 04_03103_p3 |
| 61 | - | - | - | - | Transposase | hla | - | 1 | 18412_p1 |
| 62 | - | - | - | - | Recombinase | - | - | 1 | 4126_1_p4 |
| 63 | head morphogenesis protein | lysin | - | - | Integrase; Transposase (5) | - | - | 1 | BSAR486_p5 |
| 64 | - | lysin | Single-stranded DNA-binding protein; type IV secretory system | - | Transposase (4) | - | qacJ;AAC6_Ie_APH2_Ia; ANT(6)-Ia | 1 | CIG1242_p1 |
| 65 | - | lysin (2) | Single-strand binding protein | Terminase small subunit | Transposase | sdrE; sdrD | ErmC | 1 | DEU16_p6 |
| 66 | head morphogenesis protein (2) | - | DNA binding protein; type I restriction-modification system | - | recombinase; Transposase (5) | dUTPase; set32; set19; fnbA; sdrD | mepA | 1 | ES26_p10 |
| 67 | - | lysin | - | terminase small subunit | Transposase (4) | splA; splB; splC; splD(3); splE; splF (2); fnbA (4); fnbB; dUTPase (2); sdrC; sdrD; PVL | dfrC; APH(2'')-Ia; ANT(6)-Ia; AAC6_Ie_APH2_Ia | 1 | H24_p8 |
| 68 | portal protein; head morphogenesis protein | lysin (2) | Single-strand binding protein (4); type I restriction-modification system | Terminase small subunit | Transposase (4) | sdrC; sdrD (2); sdrE; vWbp (2); ebh; set32; Virulence-associated protein E (2); dUTPase | ANT(6)-Ia; ErmA; ANT(9)-Ia; AAC6_Ie_APH2_Ia | 1 | H482_p8 |
| 69 | - | - | Type I restriction modification DNA | - | Recombinase (2); Transposase (2) | adsA | mecA; Arsenical Resistance | 1 | M1_HF937103_p1 |
| 70 | tail tape measure protein | - | DNA binding protein (2) | Terminase large subunit | Transposase (4) | sdrC; sdrD (2); sdrE; fnbB; fnbA; dUTPase; splD; splE; splF (2) | tet(K); ANT(9)-Ia; ErmA; qacJ; AAC6_Ie_APH2_Ia; ANT(6)-Ia; Arsenical Resistance; Cadmium resistance | 1 | MAL9_p9 |
| 71 | - | lysin (3) | Single-strand binding protein (2); type I restriction-modification system (2) | Terminase small subunit | Transposase (2) | ebh; sdrC (2); sdrD (3); sdrC; Virulence-associated protein E (2); fibrinogen-binding adhesin; dUTPase; Lipoprotein; PVL (3); ebh (2); set32; set38 (2); set18 | ErmA; ErmC; ANT(9)-Ia | 1 | MU4_p9 |
| 72 | - | lysin | - | - | Transposase (7) | type II toxin-antitoxin system | dfrC; ANT(6)-Ia; AAC6_Ie_APH2_Ia | 1 | NRS2_p7 |
| 73 | head morphogenesis protein (2) | - | type I restriction-modification system | - | Transposase (3) | fnbA (2); fnbB; dUTPase; set19; sdrD | AAC6_Ie_APH2_Ia; ANT(6)-Ia | 1 | M592_p12 |
| 74 | portal protein; tail tape measure protein; head morphogenesis protein | hoin | - | - | Integrase; Transposase; Clp protease | - | - | 1 | XQ_p1 |
| 75 | - | - | - | Terminase small subunit | Integrase | type II toxin-antitoxin system | - | 1 | BSAR865_p1 |
| 76 | - | - | - | Terminase small subunit | Integrase | sell; sec; tsst-1 | - | 1 | 4126_1_p3 |
| 77 | - | - | - | Terminase small subunit | - | seb | - | 1 | 10S_p1 |
| 78 | - | - | - | Terminase small subunit | Integrase | Virulence-associated protein E | - | 1 | H24_p2 |
| 79 | - | - | - | Terminase small subunit | Integrase | - | - | 1 | DAR3236_p2 |
| 80 | - | - | - | Terminase small subunit | Integrase | - | - | 1 | GKP136_11_p1 |
| 81 | Phage portal protein; Phage capsid protein; Phage tail-component; tail tape measure protein (2); Phage tail protein (2); Phage tail tube protein; Phage head-tail joining protein; Phage capsid protein; Phage portal protein | - | - | DNA packaging protein (2); Terminase large subunit | Clp protease | - | norC | 1 | D71_p5 |
| 82 | Phage tail-component; Phage head-tail connector protein; Phage minor structural protein; Phage portal protein | - | DNA helicase; Single-strand binding protein; DNA binding protein | DNA packaging protein (2) | - | dUTPase | - | 1 | H202_p3 |
| 83 | head morphogenesis protein; Phage tail protein; Phage head-tail joining protein; Phage head-tail connector protein; Phage portal protein | - | DNA helicase (2); Single-strand binding protein; DNA primase | DNA packaging protein; Phage small terminase subunit | - | dUTPase | aad(6); APH(3')-IIIa | 1 | M996_p6 |
| 84 | Phage portal protein; Phage minor structural protein; head-tail connector protein; Phage tail assembly chaperone protein; Phage tail tape measure protein; Phage tail protein | lysin | Single-strand binding protein | terminase small subunit; DNA packaging protein | integrase; Exonuclease | lukG; lukH; hlb; hld; dUTPase | - | 1 | FVRH6002_p2 |
| 85 | Phage tail protein; Phage tail tape measure protein; Phage tail tube protein; Phage head-tail joining protein; Phage capsid family; Phage portal protein | lysin | Single-strand binding protein; DNA binding protein (3) | DNA packaging protein; Phage small terminase subunit | Transposase; recombinase; integrase | eap/map; hlb; scn; sak; sea; dUTPase; lukG; lukH; | - | 1 | CN79_p3 |
| 86 | Phage portal protein; Phage head-tail joining protein; head morphogenesis protein | - | - | DNA packaging protein | Clp protease; Integrase; recombinase | sdrC (2); sdrD (3); sdrF (2); dUTPase (2); splD; splE; splF(2); set19; set32; | - | 1 | M116_p12 |
| 87 | Phage tail protein (3); Phage tail tape measure; Phage tail assembly chaperone protein; Phage tail-component; Phage head-tail connector protein; Phage portal protein; Phage minor structural protein | lysin (2) | RNA helicase | DNA packaging protein | Integrase | - | MepA; mepR | 1 | MAL11_p4 |
| 88 | Phage tail tape measure protein; Phage tail assembly chaperone protein (2); Phage tail-component (2); Phage capsid protein; Phage minor structural protein; Phage head-tail connector protein; Phage portal protein | - | type I restriction-modification system | DNA packaging protein | - | - | qacA; | 1 | MAL9_p8 |
| 89 | Phage portal protein; Phage tail tube protein (2) | - | - | Phage terminase large subunit | Clp protease; Transposase (3); Recombinase | fnbA; set32; set33; set34; set35; set38; splB; splC; splD; map (3); hlb; dUTPase; Lipoprotein; splD; splE; splF(2); | ANT(6)-Ia; AAC6_Ie_APH2_Ia | 1 | MAL11_p9 |
| 90 | Phage portal protein; Phage minor structural protein; Phage head-tail connector protein; Phage tail-component; Phage tail assembly chaperone protein; Phage tail tape measure protein; Phage tail protein | - | - | DNA packaging protein | - | sfaA; sfaB; sfaC | LmrS; SepA; sdrM | 1 | H211_p6 |
| 91 | Phage tail protein; Phage tail tape measure protein; Phage tail tube protein; Phage head-tail joining protein (2); Phage capsid protein; Phage portal protein; Phage tail protein; Phage head-tail connector protein; Phage portal protein | - | Single-strand binding protein; DNA binding protein | Phage terminase small subunit | CI repressor; Transposase; Integrase | dUTPase; eap/map; hlb | arsenical resistance | 1 | 04_02314_1_p4 |
| 92 | Phage head-tail joining protein; Phage head-tail connector protein; Phage portal protein | lysin | - | Phage terminase small subunit | - | - | - | 1 | S2397_p7 |
| 93 | Phage tail protein; Phage head-tail joining protein; Phage head-tail connector protein; Phage portal protein | - | DNA binding protein | Phage terminase small subunit | - | dUTPase; | - | 1 | MUM270_p2 |
| 94 | Phage portal protein; Phage head-tail connector protein; Phage head-tail joining protein; Phage tail protein | - | - | Phage terminase small subunit | - | - | - | 1 | H216_p9 |
| 95 | Phage portal protein | - | DNA binding protein (3); Single-strand binding protein | Terminase small subunit | - | lukH; lukG; dUTPase; hlb | - | 1 | IF6SW_P2_p3 |
| 96 | Phage tail protein (2); Phage tail tube protein (2); Phage head-tail joining protein (2); Phage capsid protein (2); Phage portal protein | Lysin; holin (2) | DNA binding protein (6); Single-strand binding protein (2) | DNA packaging protein | integrase (2); recombinase | ebp; lukF-PVL(2); lukS-PV(2); dUTPase (2); | - | 1 | MS4_p3 |
| 97 | Phage portal protein; Phage capsid family; Phage head-tail joining protein; Phage tail tube protein; Phage tail protein | lysin | DNA binding protein; Single-strand binding protein | DNA packaging protein | integrase | lukG; lukH; hlb; dUTPase; sea; sak; chp; scn | - | 1 | 1943STDY5573748_p2 |
| 98 | Phage portal protein; Phage capsid protein; Phage head-tail joining protein; Phage tail tube protein; Phage tail protein | lysin | Single-strand binding protein; type I restriction-modification system | DNA packaging protein | Transposase; integrase | atl; seg; selo; selm; seln; set15; yent1; yent2; lukE; lukD; splA; splB; splC; splF; dUTPase; Virulence-associated protein E; lipoprotein (5) | - | 1 | 91751_p8 |
| 99 | Phage portal protein; Phage capsid family; Phage head-tail joining protein; Phage tail tube protein; Phage tail tape measure protein; Phage tail protein | lysin | DNA binding protein (3) | DNA packaging protein | recombinase; integrase | dUTPase; sak; scn | - | 1 | MRSA_FKPC_p2 |
| 100 | Phage tail protein; Phage tail assembly chaperone protein; Phage major tail protein; Phage head-tail connector protein; Phage portal protein | - | - | terminase large subunit; Terminase small subunit | - | dUTPase | - | 1 | GKP138_17_p5 |
| 101 | Phage tail protein; Phage tail assembly chaperone protein; Phage major tail protein; Phage head-tail connector protein; Phage portal protein | lysin | - | terminase large subunit; Terminase small subunit | - | dUTPase | - | 1 | GKP136_8_p3 |
| 102 | Phage portal protein; Phage head-tail connector protein; Phage tail assembly chaperone protein; Phage tail protein | lysin (2); Holin | DNA binding protein; DNA polymerase | terminase large subunit; Terminase small subunit | recombinase; integrase | dUTPase | - | 1 | MRSA08_p2 |
| 103 | Phage tail protein; Phage capsid protein; Phage portal protein | - | - | DNA packaging protein (2) | - | - | - | 1 | 170_p2 |
| 104 | Phage tail protein; Phage capsid protein; Phage portal protein | lysin | DNA binding protein (3); Single-strand binding protein; Type I restriction modification protein | DNA packaging protein (2) | integrase | splA; splB; splC; splD; splE; splF; dUTPase; lukD; lukE | - | 1 | BSAR57_p2 |
| 105 | Phage portal protein; head morphogenesis protein; Phage head-tail connector protein; Phage tail-component; Phage tail tube protein; Phage tail assembly chaperone protein; Phage tail tape measure protein; Phage tail protein | - | Type I restriction modification protein; Single-strand binding protein; DNA helicase | Terminase small subunit | - | - | - | 1 | BSAR57_p3 |
| 106 | Phage tail protein; Phage capsid protein; Phage portal protein | lysin | DNA binding protein; type I restriction-modification system | DNA packaging protein (2) | - | splA; splB (2); splC; splD; splE; lukD; lukE | - | 1 | BSAR58_p3 |
| 107 | Phage portal protein; Phage minor structural protein; Phage head-tail connector protein; Phage tail-component; Phage tail protein | - | DNA binding protein (2) | DNA packaging protein | integrase | dUTPase | - | 1 | BU_G0301_t8_p2 |
| 108 | Phage tail protein; Phage tail assembly chaperone protein; Phage tail-component; Phage head-tail connector protein; Phage minor structural protein; Phage portal protein | lysin | DNA binding protein (2) | DNA packaging protein | integrase | dUTPase | - | 1 | BU_G1074_t4_p1 |
| 109 | Phage portal protein; Phage capsid protein; Phage tail assembly chaperone protein; Phage tail tape measure protein; Phage tail protein | - | - | DNA packaging protein (2) | - | dUTPase | - | 1 | SA8_LAU_p5 |
| 110 | Phage portal protein; Phage capsid protein; Phage tail assembly chaperone protein; Phage tail tape measure protein; Phage tail protein | lysin (2); Holin | Single-strand binding protein; DNA binding protein (2) | DNA packaging protein (2) | - | dUTPase | - | 1 | USA300_TCH959_p2 |
| 111 | Phage portal protein; head morphogenesis protein; Phage head-tail connector protein; Phage tail-component; Phage tail tube protein; Phage tail assembly chaperone protein; Phage tail tape measure protein; Phage tail protein | - | - | Terminase small subunit | - | splA; splB; splC; lukD; lukE | - | 1 | GKP136_11_p3 |
| 112 | Phage portal protein; Phage capsid protein; Phage tail-component; Phage tail assembly chaperone protein; Phage tail tape measure protein; Phage tail protein | lysin; Holin | Single-strand binding protein; DNA binding protein (2) | DNA packaging protein (2); terminase small subunit (2) | Transposase; Integrase | dUTPase; eno | - | 1 | AUS0325_p1 |
| 113 | Phage tail protein; Phage tail assembly chaperone protein; Phage tail-component; Phage head-tail connector protein; Phage minor structural protein | lysin | Single-strand binding protein; DNA binding protein (3) | DNA packaging protein | - | dUTPase | - | 1 | M51_p3 |
| 114 | Phage tail protein; Phage capsid protein; Phage portal protein | lysin | DNA helicase; DNA binding protein (2) | DNA packaging protein (2) | Integrase (2) | dUTPase | - | 1 | RIVM3897_p4 |
| 115 | Phage portal protein; Phage capsid protein; Phage tail protein | lysin | - | DNA packaging protein (2) | - | - | - | 1 | GKP136_11_p4 |
| 116 | Phage portal protein; Phage capsid protein; Phage tail protein | lysin | - | DNA packaging protein | recombinase | splA (2); splB; splC; splD; splE; splF | - | 1 | Sa13_005_p4 |
| 117 | Phage tail protein; Phage tail assembly chaperone protein; Phage capsid protein; Phage portal protein | - | - | DNA packaging protein (2) | - | - | - | 1 | FKWK_p6 |
| 118 | Phage portal protein; Phage capsid protein; Phage tail-component; Phage tail assembly chaperone protein; Phage tail tape measure protein; Phage tail protein | lysin | - | DNA packaging protein; terminase large subunit | - | - | - | 1 | CUHK_HK1997_p5 |
| 119 | Phage portal protein; Phage capsid protein; Phage tail-component; Phage tail assembly chaperone protein; Phage tail tape measure protein; Phage tail protein | lysin | DNA binding protein (3) | DNA packaging protein (2) | Integrase | geh | - | 1 | P32_p6 |
| 120 | Phage portal protein; head-tail connector protein; Phage tail assembly chaperone protein; Phage tail protein | lysin; Holin | DNA binding protein (3) | Terminase small subunit; terminase large subunit | Integrase | dUTPase | - | 1 | USA300_2014.C01_p6 |
| 121 | Phage portal protein; Phage capsid protein; Phage tail-component; Phage tail assembly chaperone protein; Phage tail tape measure protein; Phage tail protein | lysin | - | DNA packaging protein; terminase large subunit | - | - | - | 1 | M592_p8 |
| 122 | Phage tail protein; Phage tail assembly chaperone protein; Phage tail-component; Phage portal protein; Phage capsid protein; | lysin | Single-strand binding protein; DNA binding protein | DNA packaging protein (2) | Recombinase | dUTPase | - | 1 | SR434_p4 |
| 123 | Phage portal protein; Phage head-tail connector protein; Phage major tail protein; Phage tail protein | lysin (2); Holin | Single-strand binding protein; DNA binding protein (2) | Terminase small subunit | - | dUTPase; eno; clfA; nuc | - | 1 | MRSA_FKYJ_p1 |
| 124 | Phage portal protein; Phage minor structural protein; Phage head-tail connector protein; Phage tail-component; Phage tail protein; Phage tail assembly chaperone protein | lysin (2); Holin | - | Terminase small subunit; terminase large subunit | - | - | - | 1 | APS210_p5 |
| 125 | Phage tail protein; Phage tail assembly chaperone protein; Phage head-tail connector protein; Phage portal protein | lysin | Single-strand binding protein; DNA binding protein (2) | Terminase small subunit; terminase large subunit | - | dUTPase | - | 1 | DAR5874_p5 |
| 126 | Phage portal protein; Phage head-tail connector protein; Phage tail assembly chaperone protein; Phage tail protein | - | - | Terminase small subunit; terminase large subunit | - | - | - | 1 | LVP2_p5 |
| 127 | Phage portal protein; Phagehead-tail connector protein; Phage tail assembly chaperone protein; Phage tail protein | lysin | - | Terminase small subunit; terminase large subunit | - | - | - | 1 | BU_W12_t13_p3 |
| 128 | Phage portal protein; Phage head-tail connector protein; Phage tail assembly chaperone protein; Phage tail protein | lysin | DNA binding protein; | Terminase small subunit | Recombinase; | dUTPase | - | 1 | DAR4145_p1 |
| 129 | Phage portal protein; Phage head-tail connector protein; Phage head-tail joining protein; Phage tail protein | lysin; Holin | Single-strand binding protein; DNA binding protein (2); DNA-methyltransferase | Terminase small subunit (2) | Integrase | dUTPase | - | 1 | ED98_p1 |
| 130 | Phage portal protein; Single-strand binding protein | - | - | Terminase small subunit (3) | - | - | - | 1 | S2396_p7 |
| 131 | Phage portal protein; Phage minor structural protein; Phage head-tail connector protein; Phage tail-component; Phage tail assembly chaperone protein; Phage tail protein | lysin; Holin | - | DNA packaging protein | - | - | - | 1 | BSAR208_p4 |
| 132 | Phage portal protein; Phage head-tail connector protein; Phage head-tail joining protein; Phage tail protein | - | - | Phage terminase, small subunit | - | - | - | 1 | R0294_p6 |
| 133 | Phage portal protein; Phage tail tube protein; Phage tail protein | - | DNA binding protein; DNA polymerase | terminase small subunit | Integrase; Clp protease | Virulence-associated protein E; dUTPase | - | 1 | 1110700562_p2 |
| 134 | Phage portal protein; Phage tail tube protein; Phage tail protein | - | DNA binding protein (2); DNA polymerase | terminase small subunit | Integrase; Clp protease | dUTPase; Virulence-associated protein E | - | 1 | 43P8_p3 |
| 135 | Phage tail protein; Phage tail tube protein; Phage portal protein | Lysin (2) | DNA helicase; DNA binding protein (2) | terminase small subunit | transposase; Clp protease | dUTPase; ebp; Virulence-associated protein E | - | 1 | GD705_p2 |
| 136 | Phage portal protein; Phage tail tube protein; Phage tail protein; Type I restriction modification protein | lysin (2); Holin | - | terminase small subunit | Clp protease | dUTPase; Virulence-associated protein E | - | 1 | GKP138_71_p2 |
| 137 | Phage portal protein; Phage tail tube protein; Phage tail protein | lysin (2); Holin | DNA binding protein; Single-strand binding protein; DNA helicase | terminase small subunit | Recombinase; Clp protease | dUTPase; ebp; hla; Virulence-associated protein E | - | 1 | DAR5889_p2 |
| 138 | Phage portal protein; Phage tail tube protein; Phage tail protein | - | DNA binding protein (2); Single-strand binding protein; DNA helicase; | terminase small subunit | Integrase (2); Clp protease | dUTPase; lukH; lukG; hlb; Virulence-associated protein E | - | 1 | GKP138_41_p1 |
| 139 | Phage portal protein; Phage tail tube protein; Phage tail protein | - | DNA binding protein (3); | terminase small subunit | Clp protease; Transposase | dUTPase; eno; Virulence-associated protein E (2) | AAC6_Ie_APH2_Ia | 1 | BA01611_p2 |
| 140 | Phage portal protein; Phage tail tube protein; Phage tail protein | lysin | DNA binding protein (2); DNA polymerase | terminase small subunit | Integrase; Clp protease | Virulence-associated protein E | arsenical resistance | 1 | 1943STDY5573749_p2 |
| 141 | Phage portal protein; Phage tail tube protein; Phage tail protein | lysin ; Holin | - | terminase small subunit | Clp protease | Virulence-associated protein E | - | 1 | M116_p6 |
| 142 | Phage tail protein; Phage tail tube protein; Phage portal protein | Holin | Single-strand binding protein; DNA binding protein (3) | terminase small subunit | Clp protease; Recombinase | Virulence-associated protein E; dUTPase | - | 1 | MRSA207_p4 |
| 143 | Phage portal protein; Phage tail tube protein; Phage tail protein | Holin | - | DNA packaging protein | Recombinase; Clp protease | Virulence-associated protein E; dUTPase; isdA; isdB; isdC; isdD; isdE; isdF; isdG; srtB | - | 1 | NA32_p4 |
| 144 | head morphogenesis protein; Phage tail protein; Phage tail tube protein; Phage portal protein; Phage head-tail joining protein; Phage tail tube protein; Phage tail protein | lysin (2) | Type I restriction modification system; | terminase small subunit | Clp protease (2); Integrase | Virulence-associated protein E | blaZ | 1 | BSAR729_p3 |
| 145 | Phage portal protein; Phage tail tube protein; Phage tail protein; | - | DNA binding protein (3); | terminase small subunit | Clp protease | dUTPase; Virulence-associated protein E | tet(38) | 1 | H914_91_p5 |
| 146 | Phage tail protein; Phage tail tube protein; Phage portal protein | lysin (2) | DNA helicase; Type I restriction modification system; DNA polymerase | terminase small subunit | Clp protease; Integrase; Recombinase | Virulence-associated protein E; dUTPase; ebp | - | 1 | H_EMRSA_15_p3 |
| 147 | Phage tail protein; Phage tail tube protein; Phage portal protein (2); Phage head-tail joining protein; Phage tail tube protein; Phage tail protein | lysin | DNA repair exonuclease; | terminase small subunit | Clp protease (2); | dUTPase | - | 1 | 07_02997_p4 |
| 148 | Phage portal protein; Phage tail tube protein; Phage tail protein | - | - | terminase small subunit | Clp protease; Transposase | Virulence-associated protein E; dUTPase | aluminum resistance | 1 | 06_01900_p4 |

Table S5. Summary of the gene content of functional modules present in the *S. aureus* incomplete prophages.

| Groups | Phage morphogenesis | Host cell lysis | DNA metabolism | DNA packaging | Lysogeny | Virulence | Antimicrobial resitance | No. (%) | Represent Phage |
| --- | --- | --- | --- | --- | --- | --- | --- | --- | --- |
| 1 | head morphogenesis protein | - | - | - | Integrase | - | - | 47 | 08BA02176_ip3 |
| 2 | head morphogenesis protein (4) | - | - | - | - | - | - | 45 | 2588STDY5627534_ip2 |
| 3 | - | - | - | - | - | SplA; SplB; SplC; lukD; lukE; seg; seln; yent1; yent2; sei; selm; selo | - | 44 | 06BA18369_ip4 |
| 4 | - | - | - | - | Transposase (2) | - | - | 38 | CN79_ip1 |
| 5 | - | - | DNA-binding protein | - | Integrase (2) | - | - | 35 | CA12_ip1 |
| 6 | - | - | - | - | - | lukE; seg; sei; selm (2); selo; selu | - | 30 | 110_ip3 |
| 7 | - | - | - | - | Transposase | hla | - | 26 | 04_02981_ip3 |
| 8 | - | - | DNA-binding protein | - | Integrase (2) | - | - | 26 | 1159_SAUR_ip7 |
| 9 | - | - | DNA-binding protein | - | Integrase | - | - | 22 | 08BA02176_ip2 |
| 10 | - | - | DNA-binding protein (2) | - | Recombinase | - | - | 20 | 04_02981_ip1 |
| 11 | - | - | - | - | Integrase | selk; selq | - | 18 | HI010_ip2 |
| 12 | - | - | - | - | Integrase | sec; sell | - | 15 | 07_00309_ip2 |
| 13 | - | - | DNA-binding protein | - | Integrase | - | - | 15 | 08_02119_ip4 |
| 14 | - | - | type I restriction-modification system | - | - | seg; sei; selm; selo; seln | - | 15 | 04_03111_ip3 |
| 15 | - | - | DNA-binding protein | - | Integrase; Transposase | - | - | 14 | HZW450_ip1 |
| 16 | Phage tail tape measure protein; Phage tail tube protein; Phage head-tail joining protein; Phage capsid protein; Phage portal protein; Phage tail protein | Lysin | - | DNA packaging protein | - | sak; chp; scn; hlb | - | 14 | 229_SAUR_ip4 |
| 17 | - | - | DNA binding protein (2); Single-strand binding protein | - | Integrase | hlb; lukG; lukH; dUTPase | - | 13 | APS210_ip2 |
| 18 | - | - | - | terminase small subunit | Integrase | toxin-antitoxin (TA) module; hlb; lukG; lukH | - | 13 | 1159_SAUR_ip1 |
| 19 | - | - | DNA polymerase; DNA ligase; DNA methylase; DNA binding protein | - | Recombinase | - | - | 12 | CUHK_HK1997_ip2 |
| 20 | Phage-related minor tail protein; Phage tail protein; Phage minor structural protein | Holin; Lysin | RNA helicase; DNA-binding protein | - | Integrase | - | - | 12 | CUHK_HK2007_ip4 |
| 21 | - | - | DNA modification protein; DNA helicase; | - | Integrase | - | - | 11 | CUHK_HK1997_ip7 |
| 22 | - | - | - | Terminase small subunit | Integrase | Virulence-associated protein E; eno; clfA; emp | - | 11 | MU3_ip2 |
| 23 | Phage tail tape measure protein; Phage tail tube protein; Phage head-tail joining protein; Phage capsid protein; Phage portal protein; Phage tail protein | Lysin | - | DNA packaging protein | Clp protease; Recombinase | sea; sak; scn; hlb | - | 11 | CUHK_HK2007_ip7 |
| 24 | head morphogenesis protein (3) | - | - | - | - | - | - | 11 | 08_02300_ip1 |
| 25 | head morphogenesis protein (2); Phage portal protein | - | - | DNA packaging protein | Integrase; Transposase | - | - | 11 | 229_SAUR_ip1 |
| 26 | head morphogenesis protein; Phage portal protein | - | DNA-binding protein | DNA packaging protein | Integrase | - | - | 11 | A69_ip2 |
| 27 | - | - | - | Terminase small subunit (2) | Integrase | Virulence-associated protein E | - | 10 | 1159_SAUR_ip2 |
| 28 | - | - | - | - | Recombinase | - | - | 10 | 3503_ip3 |
| 29 | - | - | - | - | Integrase | - | - | 9 | 2009_60_561_1_ip3 |
| 30 | Phage tail protein; Phage head-tail joining protein; Phage head-tail connector protein; Phage portal protein | - | - | Terminase small subunit | - | - | - | 9 | 06BA18369_ip5 |
| 31 | head morphogenesis protein (5) | - | - | - | - | - | - | 9 | HZW450_ip2 |
| 32 | - | - | - | - | - | SplA; SplB; SplC; lukD; lukE; seg; seln; yent1; yent2; sei; selm; selo | - | 9 | 04_02981_ip4 |
| 33 | Phage portal protein; head morphogenesis protein | - | DNA binding protein; | DNA packaging protein | Transposase; Integrase; Transposase (2) | efb; hla | AAC6_Ie_APH2_Ia | 8 | BA01611_ip3 |
| 34 | - | - | - | - | Integrase (2); Transposase | - | - | 8 | 71A_S11_ip4 |
| 35 | - | Lysin (2); Holin | Type I restriction modification system (2) | - | Integrase | - | - | 8 | 75_ip1 |
| 36 | - | - | Single-strand binding protein; DNA binding protein (3) | - | - | dUTPase | - | 8 | 394_SAUR_ip3 |
| 37 | - | - | DNA binding protein | terminase small subunit | Integrase | tsst-1; sec; sell | - | 8 | 3503_ip1 |
| 38 | - | - | - | Terminase small subunit | Integrase | Virulence-associated protein E; tsst-1; sec; sell | - | 8 | 06BA18369_ip2 |
| 39 | - | - | - | - | Integrase | set24; set1; set10; set8; set31; set16; sell | - | 8 | 08_01671_ip2 |
| 40 | Phage tail tube protein; Phage tail protein | Lysin (2) | - | - | - | lukH; lukG; hlb (2); scn; sak; chp | - | 8 | 122051_ip1 |
| 41 | Phage tail protein; Phage tail tube protein; Phage head-tail joining protein; Phage capsid protein; Phage portal protein | Lysin | - | DNA packaging protein | - | hlb; scn; sak; chp; dUTPase | - | 8 | 5R_157_2_ip3 |
| 42 | Phage portal protein | - | - | DNA packaging protein | Transposase | - | aluminum resistance | 8 | FKWK_ip2 |
| 43 | - | - | type I restriction-modification system | - | - | SplB; SplC; SplD; SplE; SplF; seg; seln; selu; sei; selm; selo | - | 7 | 043H_ip3 |
| 44 | - | - | DNA binding protein; DNA polymerase | - | Integrase; Recombinase | - | - | 7 | 07_02789_ip1 |
| 45 | - | - | Single-strand binding protein; DNA binding protein | - | Integrase | lukH; lukG; hlb | - | 7 | 81_SAUR_ip2 |
| 46 | - | - | - | Terminase small subunit | Integrase | seb | - | 7 | 169_SAUR_ip2 |
| 47 | - | - | - | - | - | seg; seln; sei; selm; selo | - | 7 | A187_ip1 |
| 48 | - | - | type I restriction-modification system | - | Transposase | seg; seln; sei; selm; selo | - | 7 | 04_00608_ip3 |
| 49 | head morphogenesis protein | - | - | - | Integrase | - | - | 7 | AUS0325_ip2 |
| 50 | - | holin | DNA binding protein | - | - | lukF; lukM | - | 6 | 75_ip2 |
| 51 | - | - | DNA binding protein (2); DNA polymerase | - | - | - | - | 6 | 40P5_1_1_ip3 |
| 52 | - | - | DNA binding protein; DNA polymerase (2) | - | Integrase; Recombinase | - | - | 6 | KT_314250_ip1 |
| 53 | - | - | - | - | Integrase | lukH; lukG; hlb | - | 6 | 065H_ip2 |
| 54 | - | - | - | Terminase small subunit (2) | Integrase; Transposase | eno | - | 6 | HST_105_ip1 |
| 55 | - | - | - | Terminase small subunit | Integrase | sell; sec; eno; Virulence-associated protein E | - | 6 | 42S_ip2 |
| 56 | - | - | Type I restriction modification system | - | Transposase (2) | scn; chp | - | 6 | HZW450_ip4 |
| 57 | head morphogenesis protein (3); Phage portal protein | - | - | DNA packaging protein | Integrase; Transposase | - | - | 6 | CA_347_ip3 |
| 58 | Phage portal protein | - | DNA-binding protein | DNA packaging protein | Integrase; Transposase | - | aluminum resistance | 6 | 18754_2_ip4 |
| 59 | - | - | DNA binding protein; Single-strand binding protein | - | Integrase | lukH; lukG; hlb | - | 5 | KINW6056_ip4 |
| 60 | - | - | DNA binding protein | - | Integrase | Virulence-associated protein E | - | 5 | CN79_ip4 |
| 61 | - | - | DNA-binding protein | - | Integrase | - | - | 5 | 18754_2_ip1 |
| 62 | Phage tail protein; Phage head-tail joining protein; Phage head-tail connector protein; Phage portal protein | Lysin; Holin | Single-strand binding protein | Terminase small subunit | Recombinase | hlb; scn; sak; sea | - | 5 | OCMM6035_ip3 |
| 63 | Phage head-tail joining protein; Phage head-tail connector protein; Phage portal protein; Phage tail protein | Holin | - | Terminase small subunit | - | dUTPase; lukF; lukM | - | 5 | O11_ip3 |
| 64 | - | - | type I restriction-modification system | - | - | SplA; SplB; SplC; SplD; SplE; SplF; lukD; lukE | - | 5 | 08_02119_ip1 |
| 65 | - | - | type I restriction-modification system | - | Integrase | hysA; SplA; SplB; SplC; SplD; SplE; SplF; lukD; lukE | - | 5 | 08_02300_ip2 |
| 66 | head morphogenesis protein (5) | - | - | - | Integrase (2) | - | - | 5 | S54F9_ip2 |
| 67 | Phage tail protein; Phage tail assembly chaperone protein; Phage tail-component; Phage head-tail connector protein; head morphogenesis protein; Phage portal protein | Holin | DNA-binding protein | DNA packaging protein | Integrase | - | - | 5 | 5R_157_2_ip1 |
| 68 | - | - | - | - | - | - | - | 4 | 229_SAUR_ip2 |
| 69 | - | - | DNA binding protein (2); Single-strand binding protein | - | - | - | - | 4 | 2010_60_6511_5_ip1 |
| 70 | - | - | DNA binding protein | - | - | lukF; lukM | - | 4 | GKP136_68_ip2 |
| 71 | - | - | DNA binding protein; Single-strand binding protein | terminase small subunit | Integrase | toxin-antitoxin (TA) module; lukH; lukG; hlb | - | 4 | 07_00058_ip1 |
| 72 | - | - | - | - | Integrase; Recombinase | - | - | 4 | V1127_ip3 |
| 73 | Phage-related minor tail protein (2); Phage tail protein; Phage minor structural protein | Holin; Lysin | RNA helicase; DNA-binding protein | - | - | - | - | 4 | FVRH6002_ip6 |
| 74 | - | - | DNA-binding protein | - | Integrase | - | - | 4 | 394_SAUR_ip2 |
| 75 | Phage tail tape measure protein; Phage tail tube protein; Phage head-tail joining protein; Phage capsid protein (2); Phage portal protein; Phage tail protein | Lysin | - | DNA packaging protein | Clp protease | scn; sak; chp | - | 4 | 975_SAUR_ip4 |
| 76 | Phage portal protein; Phage head-tail joining protein; Phage tail tube protein; Phage tail protein | Lysin | - | DNA packaging protein | Clp protease | dUTPase | - | 4 | 06_01900_ip5 |
| 77 | Phage tail protein; Phage tail assembly chaperone protein; Phage tail-component; Phage head-tail connector protein; head morphogenesis protein | Holin | - | - | - | - | - | 4 | 922_SAUR_ip3 |
| 78 | - | - | - | - | - | - | aad(6) | 3 | H211_ip8 |
| 79 | - | - | DNA modification protein; DNA helicase; DNA primase | - | Transposase | - | AAC6_Ie_APH2_Ia | 3 | H211_ip9 |
| 80 | - | - | - | - | Recombinase; Transposase | - | - | 3 | TW20_ip1 |
| 81 | - | - | DNA-methyltransferase; DNA binding protein | - | - | - | - | 3 | S56_POEL_ip4 |
| 82 | - | - | Single-strand binding protein; DNA binding protein (3) | - | - | - | - | 3 | M0406_ip5 |
| 83 | Phage portal protein; Phage head-tail connector protein; Phage tail assembly chaperone protein; Phage tail protein | - | - | Terminase small subunit; terminase large subunit | - | - | - | 3 | DAR5877_ip3 |
| 84 | - | - | - | terminase small subunit | Integrase (2) | lukH; lukG; hlb; tsst-1 | - | 3 | CIG1242_ip7 |
| 85 | - | - | DNA binding protein | - | Integrase | lukH; lukG; hlb | - | 3 | DAR5867_ip4 |
| 86 | - | - | DNA binding protein; Single-strand binding protein | - | - | dUTPase | - | 3 | BSAR748_ip4 |
| 87 | - | - | DNA binding protein | - | Integrase; Recombinase | - | - | 3 | 3957_ip1 |
| 88 | - | - | DNA polymerase | - | - | - | - | 3 | KINW6056_ip8 |
| 89 | Phage-related minor tail protein; Phage tail protein; Phage minor structural protein | - | RNA helicase; DNA-binding protein | - | - | - | - | 3 | CUHK_HK1997_ip4 |
| 90 | Phage tail protein; Phage tail tube protein; Phage head-tail joining protein; Phage capsid protein; Phage portal protein | Lysin | Single-strand binding protein | DNA packaging protein | - | dUTPase | - | 3 | 06BA18369_ip6 |
| 91 | Phage head-tail joining protein; Phage tail tube protein; Phage tail protein (2) | Lysin | - | - | - | scn; sak; chp (2) | - | 3 | 6850_ip1 |
| 92 | Phage tail tube protein; Phage head-tail joining protein; Phage capsid protein; Phage portal protein | - | - | DNA packaging protein | Clp protease | - | - | 3 | CUHK_HK1997_ip10 |
| 93 | - | - | - | - | - | SplA; SplB (2); SplC; lukD; lukE | - | 3 | GKP136_21_ip2 |
| 94 | - | - | type I restriction-modification system | - | - | SplA; SplB; SplC; SplD; SplE; SplF; lukD | - | 3 | 6850_ip2 |
| 95 | - | - | DNA-binding protein | - | Transposase; Integrase | hysA; SplA; SplB; SplC; SplD; SplE; SplF; lukD; lukE | - | 3 | 57_ip1 |
| 96 | - | - | - | - | Transposase | seg; seln; sei; selm; selo | - | 3 | 08_01304_ip3 |
| 97 | head morphogenesis protein (2) | - | - | - | Integrase (2) | - | - | 3 | VET0889S_ip3 |
| 98 | - | - | DNA methyltransferase; DNA helicase; Single-strand binding protein | - | - | - | - | 2 | CUHK_HK1997_ip11 |
| 99 | - | - | DNA binding protein; Single-strand binding protein | - | - | dUTPase | - | 2 | P151_ip7 |
| 100 | - | - | - | - | Recombinase (3) | - | fusC | 2 | 337_ip2 |
| 101 | - | - | - | - | Transposase (2) | - | AAC6_Ie_APH2_Ia | 2 | CN79_ip5 |
| 102 | - | - | - | - | Transposase | - | dfrC | 2 | Sa12_002_ip4 |
| 103 | - | - | Single-strand binding protein; DNA binding protein (3) | - | - | - | - | 2 | M1311_ip3 |
| 104 | - | - | DNA binding protein | - | Recombinase | - | - | 2 | 3957_ip3 |
| 105 | - | - | DNA binding protein (2) | - | Recombinase | - | - | 2 | BU_G0201_t8_ip6 |
| 106 | - | - | DNA binding protein (3) | - | Recombinase | - | - | 2 | 06BA18369_ip3 |
| 107 | - | - | DNA binding protein (3); Single-strand binding protein | - | Recombinase | PVL | - | 2 | P151_ip3 |
| 108 | - | - | - | - | - | - | - | 2 | Sa12_002_ip1 |
| 109 | - | - | - | terminase small subunit | Clp protease | Virulence-associated protein E; dUTPase | - | 2 | 11P8_ip2 |
| 110 | - | - | - | terminase small subunit | Integrase (2) | lukH; lukG; hlb; tsst-1 | - | 2 | SCCmec1_ip3 |
| 111 | - | - | DNA binding protein; DNA polymerase | - | Recombinase | - | - | 2 | D71_ip3 |
| 112 | - | - | DNA polymerase; DNA ligase; DNA binding protein | - | Recombinase | - | - | 2 | M116_ip4 |
| 113 | - | - | DNA binding protein (2); DNA helicase | - | - | - | - | 2 | A71_ip3 |
| 114 | - | - | DNA binding protein; DNA polymerase | - | Integrase; Recombinase | - | - | 2 | DAR5898_ip3 |
| 115 | - | - | Single-strand binding protein | - | - | - | - | 2 | Na21_ip11 |
| 116 | - | - | - | - | Integrase | lukH; lukG; hlb | - | 2 | ATCC_29213_ip3 |
| 117 | - | - | DNA binding protein | - | Integrase | lukH; lukG; hlb | - | 2 | HOU1445_VS_ip4 |
| 118 | - | - | - | - | - | type II toxin-antitoxin system; sec; sell | - | 2 | GKP136_36_ip3 |
| 119 | - | - | - | - | Integrase | - | - | 2 | 07_02088_ip3 |
| 120 | - | - | - | - | Integrase | - | - | 2 | R0353_ip7 |
| 121 | head morphogenesis protein (4) | - | - | - | - | - | blaZ | 2 | 57_ip3 |
| 122 | Phage portal protein; Phage capsid protein (2); Phage head-tail joining protein; Phage tail tube protein; Phage tail protein | Lysin | DNA-binding protein (3); Single-strand binding protein | DNA packaging protein | Recombinase (2) | dUTPase | - | 2 | 3503_ip2 |
| 123 | Phage tail protein; Phage head-tail joining protein; Phage head-tail connector protein; Phage portal protein | - | Single-strand binding protein | Terminase small subunit | - | dUTPase | - | 2 | 10S_ip2 |
| 124 | - | - | - | - | - | - | - | 2 | P151_ip8 |
| 125 | - | - | - | - | Recombinase | - | ANT(9)-Ia; ErmA | 2 | M1311_ip1 |
| 126 | - | - | - | - | - | dUTPase | - | 2 | 170_ip6 |
| 127 | - | - | DNA-binding protein (2) | - | Integrase | - | - | 2 | R0294_ip1 |
| 128 | Phage portal protein; Phage capsid protein; Phage tail tube protein; Phage head-tail joining protein; Phage tail tape measure protein | - | DNA-binding protein (2) | DNA packaging protein | Integrase | lukH; lukG; hlb; dUTPase | - | 2 | CC022_ip4 |
| 129 | Phage tail tape measure protein; Phage tail tube protein; Phage head-tail joining protein; Phage capsid protein; Phage portal protein; Phage tail protein | Lysin | - | DNA packaging protein | - | dUTPase; scn; sak; hlb | - | 2 | LVP5_ip6 |
| 130 | Phage tail tape measure protein; Phage tail tube protein; Phage head-tail joining protein; Phage capsid protein; Phage portal protein; Phage tail protein | - | - | DNA packaging protein | Recombinase | - | - | 2 | H211_ip7 |
| 131 | Phage tail protein; Phage tail tube protein; Phage head-tail joining protein; Phage portal protein | Lysin (2) | - | DNA packaging protein | Clp protease | hlb; scn; sak; chp; dUTPase | - | 2 | 1159_SAUR_ip5 |
| 132 | - | - | type I restriction-modification system | - | - | SplA; SplB; SplC; SplD; SplE; SplF; lukD; lukE; lukH; lukG; hlb | - | 2 | 92_ip4 |
| 133 | - | - | - | - | Integrase | - | aluminum resistance | 2 | M0690_ip1 |
| 134 | - | - | - | - | Integrase | - | - | 2 | APS210_ip1 |
| 135 | - | - | - | - | Integrase | - | aluminum resistance | 2 | 51S_ip1 |
| 136 | head morphogenesis protein (3); Phage portal protein | - | - | Phage terminase large subunit | Transposase | - | - | 2 | 51S_ip2 |
| 137 | head morphogenesis protein; Phage portal protein | - | DNA-binding protein | DNA packaging protein | Integrase | - | aluminum resistance | 2 | QR502_ip4 |
| 138 | - | - | - | - | - | efb; hla | - | 1 | F26051_ip1 |
| 139 | - | - | DNA binding protein (2); | - | Recombinase | - | - | 1 | APS211_ip5 |
| 140 | - | - | DNA helicase; Single-strand binding protein; DNA binding protein | - | - | - | - | 1 | GKP138_17_ip7 |
| 141 | - | - | - | terminase small subunit | - | - | - | 1 | FRI137_ip5 |
| 142 | - | - | - | - | Transposase | hla | - | 1 | DAR4145_ip3 |
| 143 | - | - | - | - | - | fnbB; SplB; SplC; SplD | AAC6_Ie_APH2_Ia; ANT(4')-Ib (2) | 1 | D71_ip7 |
| 144 | - | - | DNA binding protein; | - | Transposase | lipoprotein;spa; set19; splF; esaG; dUTPase | ANT(6)-Ia; AAC6_Ie_APH2_Ia | 1 | D71_ip8 |
| 145 | - | - | - | - | - | - | - | 1 | H202_ip8 |
| 146 | - | - | DNA polymerase; DNA helicase; Single-strand binding protein | - | - | PVL | - | 1 | H211_ip10 |
| 147 | - | - | Single-strand binding protein; type I restriction-modification system | - | Recombinase; Transposase | spa; fnbA; fnbB; SplB; SplC; SplD; dUTPase; PVL | AAC6_Ie_APH2_Ia; ANT(6)-Ia | 1 | H211_ip12 |
| 148 | - | - | type I restriction-modification system | - | Transposase | SplB; SplC; SplD; SplE; set19; dUTPase | AAC6_Ie_APH2_Ia | 1 | H211_ip13 |
| 149 | - | - | - | - | Recombinase | set19; set34; set32; dUTPase (2); lipoprotein (3) | - | 1 | H211_ip14 |
| 150 | - | - | Single-strand binding protein; DNA helicase; type I restriction-modification system | - | Transposase; | - | dfrG | 1 | H216_ip11 |
| 151 | - | holin | - | - | Transposase | fnbA; SplD; SplE; SplF (2); hlb; dUTPase (3) | AAC6_Ie_APH2_Ia; ANT(6)-Ia | 1 | H216_ip13 |
| 152 | - | - | type I restriction-modification system | - | Transposase | dUTPase | - | 1 | Na21_ip13 |
| 153 | - | Lysin | type I restriction-modification system (2) | - | Transposase | sak; set19; fnbA; SplB; SplC; SplF; Lipoprotein | - | 1 | Na21_ip14 |
| 154 | - | - | type I restriction-modification system | - | Transposase (3) | SplD; SplE; SplF (2) | AAC6_Ie_APH2_Ia | 1 | NA32_ip9 |
| 155 | - | - | - | - | Transposase | SplD; SplE; SplF (2); set32; dUTPase | arlR | 1 | P32_ip10 |
| 156 | - | - | RNA helicase; DNA binding protein; DNA modification protein; | - | - | Virulence-associated protein E; lip; icaC; icaB; icaA | - | 1 | H202_ip7 |
| 157 | - | - | DNA helicase; DNA N-6-adenine-methyltransferase; DNA binding protein | - | - | - | aad(6) | 1 | H24_ip7 |
| 158 | - | - | DNA binding protein; Single-strand binding protein | Terminase small subunit | Transposase ; Integrase | sasA | tet(38) | 1 | H202_ip6 |
| 159 | - | Lysin | - | - | Integrase | sak; clfA; vWbp | - | 1 | H202_ip10 |
| 160 | - | Lysin (2) | - | - | Integrase | ebh; sdrC; sdrD (2); sdrE; sak | - | 1 | H211_ip11 |
| 161 | - | - | Single-strand binding protein | - | Transposase (2); Integrase | - | - | 1 | H216_ip12 |
| 162 | Phage portal protein | - | DNA helicase; DNA binding protein (3) | terminase small subunit (2); Terminase large subunit | - | sdrC; sdrD; emp | - | 1 | H482_ip7 |
| 163 | head morphogenesis protein | - | DNA binding protein (2); Single-strand binding protein | - | Integrase | geh; emp; Lipoprotein (2); Virulence-associated protein E (2); vWbp; clfA; sdrC; sdrD (2); sdrE | qacJ | 1 | HU8_ip8 |
| 164 | - | - | Single-strand binding protein | - | Transposase | - | - | 1 | MAL9_ip7 |
| 165 | Phage portal protein; Phage head-tail connector protein; Phage tail-component; Phage tail assembly chaperone protein; Phage tail protein | - | - | DNA packaging protein | - | - | - | 1 | MR1_ip3 |
| 166 | Phage portal protein; Phage head-tail connector protein; Phage major tail protein; Phage tail assembly chaperone protein; Phage tail protein | - | - | Terminase small subunit | - | - | - | 1 | R0294_ip7 |
| 167 | - | - | DNA binding protein (3); | - | Transposase | - | cat-TC | 1 | RA3_ip8 |
| 168 | - | - | DNA binding protein (2); | - | - | - | - | 1 | S2396_ip9 |
| 169 | Phage portal protein | - | - | DNA packaging protein | - | - | - | 1 | st3046_ip8 |
| 170 | - | - | single-stranded DNA exodeoxyribonuclease; DNA primase; DNA helicase; DNA modification protein | - | Integrase | - | APH(3')-IIIa; aad(6) | 1 | MAL9_ip5 |
| 171 | Phage tail protein; Phage tail tape measure protein; Phage tail tube protein; Phage head-tail joining protein; Phage capsid protein; Phage portal protein | - | - | DNA packaging protein | Clp protease | scn; hlb; eap/map; set35 | dfrC; ANT(6)-Ia; AAC6_Ie_APH2_Ia | 1 | MRGR3_ip1 |
| 172 | - | - | single-stranded DNA exodeoxyribonuclease; DNA primase; DNA helicase; DNA modification protein | - | - | - | - | 1 | H216_ip10 |
| 173 | - | - | single-stranded DNA exodeoxyribonuclease; DNA primase; DNA helicase; DNA modification protein | - | Integrase | sdrD; sdrE; lukD; lukE | bla | 1 | Na21_ip10 |
| 174 | - | Lysin; Holin | - | - | - | - | - | 1 | 91751_ip6 |
| 175 | - | Holin | DNA polymerase | - | - | scn; hlb; eap/map | - | 1 | MRGR3_ip12 |
| 176 | - | - | - | - | Integrase | selk; selq; Virulence-associated protein E | - | 1 | F19490_ip2 |
| 177 | - | - | DNA polymerase; DNA binding protein (3); Type I restriction modification | - | Integrase | type II toxin-antitoxin system; lukH; lukG; hlb; set40; selq; selk; Lipoprotein (6) | - | 1 | H482_ip6 |
| 178 | - | - | - | - | Transposase (3); Integrase (2) | - | blaZ | 1 | CN1_ip1 |
| 179 | - | - | Single-strand binding protein; DNA helicase | - | - | PVL; dUTPase | - | 1 | HU8_ip6 |
| 180 | - | - | DNA-binding protein | Terminase small subunit | - | selq; selk | - | 1 | LCT_SAO_ip6 |
| 181 | - | - | - | Terminase small subunit | - | seb; selk; Virulence-associated protein E | - | 1 | MS4_T60_ip3 |
| 182 | - | - | - | - | Integrase | toxin-antitoxin (TA) module; efb; seh; sdrE; sdrD | arlR; arlS; dfrC | 1 | S2396_ip1 |
| 183 | - | - | Single-strand binding protein; DNA helicase | - | - | dUTPase | - | 1 | NMR02_ip7 |
| 184 | - | - | - | - | - | dUTPase (2) | - | 1 | 302_ip4 |
| 185 | - | - | - | - | Integrase | geh | - | 1 | st1831_ip3 |
| 186 | - | - | DNA binding protein; DNA helicase | - | - | toxin-antitoxin module; geh | - | 1 | CUHK_HK1997_ip8 |
| 187 | - | - | DNA binding protein (3) | - | Integrase | geh | - | 1 | A5_ip4 |
| 188 | - | - | DNA binding protein (2) | - | Integrase | geh | - | 1 | 06BA18369_ip1 |
| 189 | - | - | DNA binding protein; Single-strand binding protein; DNA helicase; | - | - | dUTPase | - | 1 | A5_ip5 |
| 190 | - | - | DNA methyltransferase; DNA binding protein; | - | Integrase | dUTPase; geh | - | 1 | 1943STDY5698363_ip2 |
| 191 | - | - | DNA polymerase; DNA binding protein; | - | Integrase | geh | - | 1 | O11_ip1 |
| 192 | Phage tail tube protein | - | - | - | Integrase | geh | - | 1 | 91751_ip4 |
| 193 | - | - | - | - | Integrase | geh | - | 1 | GKP138_17_ip1 |
| 194 | - | - | - | - | Integrase | geh (2) | - | 1 | HST_077_ip5 |
| 195 | - | - | DNA binding protein (2) | - | Integrase | geh | - | 1 | FRI137_ip2 |
| 196 | - | - | DNA binding protein (3) | - | Recombinase (2); Integrase | lukH; lukG; hlb | - | 1 | H24_ip6 |
| 197 | - | - | - | - | Recombinase (2); | - | dfrC | 1 | HU8_ip5 |
| 198 | - | - | - | - | Recombinase | - | dfrC | 1 | M592_ip10 |
| 199 | head morphogenesis protein | - | DNA helicase | - | - | - | LmrS; SepA; sdrM | 1 | MRGR3_ip3 |
| 200 | - | - | - | terminase small subunit | Integrase | lukH; lukG; hlb | dfrC | 1 | MU4_ip6 |
| 201 | - | - | - | - | Transposase (3) | ebh | ANT(6)-Ia; AAC6_Ie_APH2_Ia; dfrC | 1 | MUM270_ip4 |
| 202 | - | - | - | - | Transposase (2) | seh | arlR; arlS | 1 | S2396_ip11 |
| 203 | Phage head-tail connector protein; head morphogenesis protein (3) | - | - | - | Transposase | sdrD; sdrE | dfrC | 1 | S2396_ip12 |
| 204 | - | - | DNA binding protein (2); Single-strand binding protein; DNA helicase; DNA methylation | - | - | dUTPase | - | 1 | Chi_8_ip2 |
| 205 | - | - | DNA-methyltransferase; DNA binding protein (4); Single-strand binding protein; DNA helicase | - | - | - | - | 1 | 25_2889_ip5 |
| 206 | - | - | DNA binding protein | Terminase small subunit | - | Virulence-associated protein E; selq; selk | - | 1 | LVP5_ip2 |
| 207 | - | holin | DNA binding protein | - | - | lukM; lukF | - | 1 | 112_ip2 |
| 208 | - | - | DNA helicase; DNA binding protein; Single-strand binding protein | - | - | - | - | 1 | 2011_60_2275_1_ip6 |
| 209 | - | - | - | Terminase small subunit | Integrase | Virulence-associated protein E | - | 1 | ATCC_25923_ip4 |
| 210 | - | - | - | Terminase small subunit | Integrase | - | - | 1 | ES26_ip1 |
| 211 | - | - | DNA binding protein (3); DNA helicase | - | - | selq; selk | - | 1 | S2397_ip1 |
| 212 | - | - | DNA binding protein (2); Single-strand binding protein | - | Recombinase | - | arlR | 1 | USFL022_ip2 |
| 213 | - | - | Single-strand binding protein | - | - | - | - | 1 | W45755_111412_ip11 |
| 214 | - | - | Single-strand binding protein | - | - | - | - | 1 | W82303_ip4 |
| 215 | - | - | DNA binding protein (2); Single-strand binding protein; DNA helicase; DNA methylation | - | - | dUTPase | - | 1 | Tur_15_ip2 |
| 216 | - | - | Single-strand binding protein; DNA binding protein (2) | Terminase small subunit | - | Virulence-associated protein E | - | 1 | LVP5_ip1 |
| 217 | - | - | Single-strand binding protein; DNA binding protein (2) | - | - | - | - | 1 | CUHK_HK188_ip2 |
| 218 | - | - | Single-strand binding protein; DNA binding protein (2) | - | Recombinase; Integrase (2) | - | Arsenical Resistance | 1 | DAR5854_ip2 |
| 219 | Phage tail protein; Phage tail assembly chaperone protein; Phage head-tail connector protein | - | DNA binding protein | - | Recombinase (2) | hysA | mercury resistance | 1 | H482_ip5 |
| 220 | - | - | Single-strand binding protein; DNA binding protein (2) | Terminase small subunit; terminase large subunit | - | dUTPase | - | 1 | SA8_LAU_ip4 |
| 221 | Phage tail protein; Phage tail assembly chaperone protein; Phage head-tail connector protein; Phage portal protein | Lysin (2); holin | Single-strand binding protein; DNA binding protein (3) | Terminase small subunit | - | dUTPase | - | 1 | SA_ST125_MupR_ip4 |
| 222 | Phage portal protein; Phage head-tail connector protein; Phage tail assembly chaperone protein; Phage tail protein | - | - | Terminase small subunit; terminase large subunit | - | dUTPase | - | 1 | SA_260_ip2 |
| 223 | Phage portal protein; Phage head-tail connector protein; Phage tail assembly chaperone protein; Phage tail protein | - | - | Terminase small subunit | - | - | - | 1 | MU4_ip5 |
| 224 | Phage tail assembly chaperone protein; Phage tail-component; Phage head-tail connector protein; Phage minor structural protein; Phage portal protein | - | - | DNA packaging protein | - | dUTPase | - | 1 | 10S_ip3 |
| 225 | - | - | Single-strand binding protein; DNA binding protein | - | Integrase | - | - | 1 | GKP136_11_ip5 |
| 226 | - | - | DNA binding protein (3) | - | - | - | LmrS; SepA; sdrM | 1 | BSAR208_ip1 |
| 227 | - | - | Single-strand binding protein; DNA binding protein (3) | - | Integrase | - | - | 1 | P32_ip9 |
| 228 | - | - | DNA binding protein | - | Integrase | - | - | 1 | R0294_ip8 |
| 229 | - | - | Single-stranded DNA-binding protein; DNA helicase; DNA binding protein | - | Integrase | - | - | 1 | S2396_ip3 |
| 230 | - | - | DNA polymerase | - | - | - | - | 1 | S56_POEL_ip7 |
| 231 | - | - | Single-strand binding protein; DNA binding protein (3) | - | - | - | - | 1 | O11_ip2 |
| 232 | - | - | Single-strand binding protein; DNA binding protein (3) | - | Integrase | dUTPase | - | 1 | USFL253_ip4 |
| 233 | - | - | DNA binding protein | - | Recombinase | - | - | 1 | APS210_ip4 |
| 234 | - | - | DNA binding protein | - | Recombinase | - | - | 1 | DEU16_ip1 |
| 235 | - | - | DNA binding protein (3) | - | Recombinase | - | - | 1 | H216_ip6 |
| 236 | - | Lysin | - | - | Recombinase | - | - | 1 | HU8_ip1 |
| 237 | - | - | DNA binding protein (2) | - | Integrase | - | - | 1 | R0353_ip3 |
| 238 | - | - | DNA binding protein | - | Integrase | toxin-antitoxin (TA) module | - | 1 | R0615_ip4 |
| 239 | - | - | Single-strand binding protein; DNA binding protein (3) | - | Recombinase | dUTPase | - | 1 | USA_1_ip4 |
| 240 | - | - | DNA binding protein (2) | - | Integrase | - | - | 1 | R0294_ip2 |
| 241 | - | - | DNA binding protein; type I restriction-modification system | - | Recombinase | - | - | 1 | 91751_ip9 |
| 242 | - | - | DNA binding protein (2); Single-strand binding protein | - | Recombinase | - | - | 1 | SA_120_ip1 |
| 243 | - | - | DNA binding protein (3) | - | Recombinase | - | - | 1 | NRS2_ip6 |
| 244 | - | - | DNA binding protein (2) | - | Integrase | Virulence-associated protein E | - | 1 | O46_ip5 |
| 245 | - | - | DNA binding protein | - | - | - | - | 1 | SA_260_ip6 |
| 246 | - | - | DNA binding protein (3) | - | Recombinase | - | - | 1 | GKP138_71_ip3 |
| 247 | - | - | DNA binding protein (3) | - | Recombinase | dUTPase | - | 1 | MRSA_FKTN_ip3 |
| 248 | head morphogenesis protein (4) | - | DNA binding protein | - | Integrase | - | - | 1 | Sa13_006_ip5 |
| 249 | - | - | DNA binding protein (2); Single-strand binding protein | - | Recombinase | - | - | 1 | V1142_ip1 |
| 250 | - | - | DNA polymerase | - | Transposase (4) | - | - | 1 | 92_ip3 |
| 251 | - | - | - | - | Integrase; Transposase (2) | lukS-PV; lukF-PV | - | 1 | 91751_ip7 |
| 252 | - | - | DNA binding protein (2); | - | Recombinase | ebh; sdrC; sdrD (2); sdrE (2); sak; spa; fnbB; splB; splC; splD; splF; set19; set32; set34 | AAC6_Ie_APH2_Ia; ANT(6)-Ia; ErmA; ANT(9)-Ia | 1 | H211_ip1 |
| 253 | - | - | - | - | Transposase (4) | - | - | 1 | JS395_ip4 |
| 254 | - | - | - | - | - | - | - | 1 | KLT6_ip1 |
| 255 | - | - | DNA binding protein (2) | - | Recombinase | toxin-antitoxin (TA) module | - | 1 | M592_ip5 |
| 256 | - | - | DNA binding protein (2) | - | Recombinase (2); Transposase | - | - | 1 | MRGR3_ip11 |
| 257 | - | - | DNA polymerase | - | Transposase (4) | - | - | 1 | MRSA_FKYJ_ip3 |
| 258 | - | - | - | - | Transposase (2); Integrase | - | - | 1 | MSSA_FKNV_ip4 |
| 259 | - | - | - | - | Recombinase | - | - | 1 | Na21_ip4 |
| 260 | - | - | - | - | Transposase | - | - | 1 | W45755_111412_ip3 |
| 261 | - | - | DNA polymerase | - | Recombinase | - | - | 1 | NA32_ip6 |
| 262 | - | - | - | - | Recombinase | toxin-antitoxin (TA) module | - | 1 | NMR05_ip4 |
| 263 | - | - | DNA binding protein (2); DNA helicase | - | Integrase | - | - | 1 | GKP136_68_ip4 |
| 264 | - | - | type I restriction-modification system | - | - | seg; seln; selu; sei; selm; selo; lukD; SplA; SplB; SplC; SplF | - | 1 | IF7SW_P3_ip2 |
| 265 | - | - | DNA binding protein | - | Recombinase | toxin-antitoxin (TA) module | - | 1 | M116_ip5 |
| 266 | Phage tail protein; Phage tail assembly chaperone protein; Phage major tail protein; Phage head-tail connector protein; Phage portal protein | Lysin | DNA helicase; DNA binding protein (2); Single-strand binding protein | terminase large subunit; Terminase small subunit | Integrase | lukE; seg; seln; selu; sei; selo; selm (2); dUTPase | - | 1 | RF122_ip3 |
| 267 | - | - | DNA helicase; DNA binding protein (2); Single-strand binding protein | - | Integrase | toxin-antitoxin (TA) module; dUTPase | - | 1 | GKP136_11_ip2 |
| 268 | - | - | DNA binding protein | - | Integrase | lukD; lukE; seg; seln; selu; sei; selo (2); selm | - | 1 | GKP138_71_ip6 |
| 269 | - | - | DNA binding protein | - | - | seg; seln; selu; sei; selo; selm (2); SplB; SplC; SplD; SplE | - | 1 | S2397_ip5 |
| 270 | - | - | DNA binding protein (2) | - | Integrase | ebh | - | 1 | SA3_LAU_ip1 |
| 271 | - | - | DNA binding protein (2) | - | Integrase | lukH; lukG; hlb | - | 1 | st1831_ip2 |
| 272 | - | - | DNA binding protein (2); Single-strand binding protein | - | Recombinase; Integrase | dUTPase | - | 1 | 975_SAUR_ip3 |
| 273 | - | - | DNA binding protein (3); Single-strand binding protein | - | Integrase | type II toxin-antitoxin system; ebh | - | 1 | F19490_ip1 |
| 274 | - | - | DNA binding protein (2) | - | - | - | - | 1 | W45755_111412_ip13 |
| 275 | - | - | DNA helicase; DNA binding protein; Single-strand binding protein; DNA methyltransferase | terminase small subunit | Recombinase; | toxin-antitoxin (TA) module | - | 1 | MAL11_ip5 |
| 276 | - | - | DNA binding protein (2) | - | Integrase | ebh | - | 1 | Mq2T_ip2 |
| 277 | Phage portal protein | - | - | terminase, large subunit; DNA packaging protein (2) | - | - | - | 1 | st3046_ip9 |
| 278 | Phage tail tube protein; Phage portal protein | - | - | - | Clp protease | - | - | 1 | W45755_111412_ip9 |
| 279 | Phage tail protein | holin | - | - | - | - | - | 1 | W45755_111412_ip4 |
| 280 | Phage tail protein; Phage tail tube protein; Phage portal protein | holin | - | terminase small subunit | Clp protease | Virulence-associated protein E | - | 1 | OCMM6035_ip5 |
| 281 | - | - | DNA binding protein | - | - | - | - | 1 | W45755_111412_ip10 |
| 282 | - | - | - | - | Integrase | lukH; lukG; hlb | - | 1 | S2398_ip5 |
| 283 | - | - | DNA binding protein | - | Integrase | type II toxin-antitoxin system; lukH; lukG; hlb | - | 1 | 043H_ip5 |
| 284 | - | - | - | terminase small subunit | Integrase | tsst-1 | - | 1 | st1335_ip2 |
| 285 | - | - | - | - | Integrase | lukH; lukG; hlb | - | 1 | SA8_LAU_ip3 |
| 286 | - | - | DNA binding protein (2); Single-strand binding protein | - | - | - | - | 1 | ES26_ip9 |
| 287 | - | - | - | terminase small subunit | - | hlgB; hlgC | - | 1 | ES26_ip3 |
| 288 | - | - | DNA binding protein (3); Single-strand binding protein | - | Integrase | lukH; lukG; hlb | LmrS | 1 | M278_ip4 |
| 289 | - | - | DNA binding protein | - | Integrase | toxin-antitoxin (TA) module; lukH; lukG; hlb | - | 1 | OC3_ip6 |
| 290 | - | - | DNA polymerase; DNA ligase | - | Recombinase | - | APH(3')-IIIa | 1 | MRGR3_ip9 |
| 291 | - | - | - | - | - | - | - | 1 | H202_ip2 |
| 292 | - | - | DNA polymerase; DNA ligase; DNA methylase; DNA binding protein | - | Recombinase | type II toxin-antitoxin system; sbi | - | 1 | MAL9_ip4 |
| 293 | - | - | DNA polymerase; DNA ligase; DNA binding protein | - | Recombinase | hld | - | 1 | H211_ip4 |
| 294 | - | - | - | terminase small subunit | Integrase | toxin-antitoxin (TA) module; lukH; lukG; hlb | - | 1 | 08_01486_ip2 |
| 295 | - | - | - | terminase small subunit | Integrase | toxin-antitoxin (TA) module; lukH; lukG; hlb | - | 1 | BSAR748_ip2 |
| 296 | - | - | - | terminase small subunit | Integrase | lukH; lukG; hlb | - | 1 | 04_02314_1_ip2 |
| 297 | - | - | - | - | Integrase | toxin-antitoxin (TA) module; lukH; lukG; hlb | - | 1 | PB1_1_ip1 |
| 298 | - | - | DNA binding protein; Single-strand binding protein | terminase small subunit | Integrase | toxin-antitoxin (TA) module (2); lukH; lukG; hlb | - | 1 | 06_01900_ip2 |
| 299 | - | - | DNA binding protein; DNA polymerase | - | Integrase; Transposase | dUTPase; Virulence-associated protein E | - | 1 | st2787_ip3 |
| 300 | - | - | - | - | Integrase; Recombinase | - | - | 1 | 04_00608_ip2 |
| 301 | - | - | DNA binding protein; DNA polymerase | - | Integrase; Transposase | - | - | 1 | 07_00058_ip3 |
| 302 | - | - | DNA binding protein; DNA polymerase | - | Recombinase (2) | - | - | 1 | 07_03345_ip3 |
| 303 | - | - | DNA binding protein; DNA polymerase | - | Transposase (2) | - | arsenical resistance | 1 | 08_01486_ip4 |
| 304 | - | - | DNA binding protein; DNA polymerase | - | Integrase | dUTPase; Virulence-associated protein E | - | 1 | 08_01669_ip2 |
| 305 | - | Lysin; Holin | DNA helicase | - | Integrase; Recombinase | ebp; lukF-PV; lukS-PV | - | 1 | 71A_S11_ip2 |
| 306 | - | - | DNA binding protein; DNA polymerase | - | Integrase; Transposase; Recombinase | dUTPase; Virulence-associated protein E | - | 1 | ARI31_ip2 |
| 307 | - | - | DNA binding protein; DNA polymerase | - | Integrase | - | - | 1 | BSAR208_ip5 |
| 308 | - | - | DNA binding protein; | - | Integrase; Recombinase | PVL; dUTPase | - | 1 | BSAR729_ip1 |
| 309 | - | - | DNA binding protein; DNA polymerase | - | - | - | - | 1 | BSAR729_ip6 |
| 310 | - | Lysin | DNA binding protein; | - | Integrase | hla; efb | - | 1 | BSAR734_ip3 |
| 311 | - | - | DNA binding protein; DNA polymerase | - | Integrase | cna | - | 1 | BSAR865_ip5 |
| 312 | - | - | DNA binding protein; DNA helicase | - | - | - | - | 1 | O46_ip6 |
| 313 | - | - | DNA polymerase | - | - | - | - | 1 | PB1_1_ip2 |
| 314 | - | - | DNA binding protein; DNA polymerase | - | Integrase; Recombinase | - | - | 1 | 08_01304_ip1 |
| 315 | - | - | DNA binding protein; DNA polymerase | - | Integrase; Recombinase | hlgA (2); hlgB; hlgC | - | 1 | 04_03103_ip1 |
| 316 | - | - | DNA binding protein; DNA polymerase | - | Integrase; Recombinase | - | - | 1 | 07_00655_ip2 |
| 317 | - | Lysin | DNA helicase; DNA polymerase; DNA binding protein | - | Integrase | dUTPase; ebp | - | 1 | 07_02997_ip2 |
| 318 | - | - | DNA binding protein; DNA polymerase | - | Integrase; Recombinase | sdrE | - | 1 | 07_03346_ip2 |
| 319 | - | Lysin | DNA binding protein; DNA polymerase; Type I restriction modification | - | - | - | - | 1 | 08_01483_ip3 |
| 320 | - | - | DNA binding protein; DNA polymerase | - | Integrase; Recombinase | - | - | 1 | 08_01667_ip2 |
| 321 | - | - | DNA binding protein; DNA polymerase | - | Integrase; Recombinase | - | - | 1 | BSAR176_2_ip2 |
| 322 | - | - | DNA binding protein; DNA polymerase | - | Integrase; Recombinase | sfaA; sfaB; sfaC; sfaD | LmrS; SepA; sdrM | 1 | 07_01497_ip1 |
| 323 | - | Lysin | DNA binding protein; DNA polymerase | - | Integrase; Recombinase; Transposase; | - | - | 1 | 08_01668_ip1 |
| 324 | - | - | DNA polymerase | - | - | dUTPase | - | 1 | 25_2889_ip6 |
| 325 | - | - | DNA binding protein | - | Integrase | - | - | 1 | 065H_ip5 |
| 326 | - | - | - | - | - | dUTPase (2) | - | 1 | 170_ip5 |
| 327 | - | - | DNA binding protein (3); DNA polymerase; DNA-methyltransferase | - | - | - | - | 1 | 2011_60_2275_1_ip2 |
| 328 | - | - | DNA binding protein; DNA polymerase | - | Integrase | dUTPase | - | 1 | Chi_8_ip5 |
| 329 | - | - | DNA binding protein (2) | - | Integrase | - | - | 1 | MS4_LZD100_ip2 |
| 330 | - | - | DNA binding protein; Single-strand binding protein; DNA helicase | - | Integrase | dUTPase | - | 1 | TSAR07_ip2 |
| 331 | - | - | DNA binding protein (2); DNA polymerase | - | Integrase; Recombinase | - | - | 1 | TSAR05_ip3 |
| 332 | - | - | DNA binding protein (3); DNA polymerase | - | Integrase | - | - | 1 | st1831_ip5 |
| 333 | - | - | DNA binding protein (2) | - | Integrase; Recombinase | - | - | 1 | FRI137_ip4 |
| 334 | - | - | Type I restriction modification system (3) | - | Integrase (2) | - | - | 1 | 1364_ip1 |
| 335 | - | Lysin | - | - | Integrase; Transposase | - | - | 1 | 170_ip4 |
| 336 | - | - | DNA binding protein; Type I restriction modification system (2) | - | Integrase (2) | - | - | 1 | 170_ip3 |
| 337 | - | - | DNA binding protein | - | Integrase | - | - | 1 | 302_ip3 |
| 338 | Phage tail protein; Phage tail tube protein; Phage portal protein | Holin | DNA polymerase | Terminase, small subunit | Clp protease; Integrase | Virulence-associated protein E; lukF-PV; lukS-PV; dUTPase | - | 1 | MRSA140_ip4 |
| 339 | head morphogenesis protein | - | DNA binding protein (2) | - | Integrase | - | - | 1 | S2397_ip4 |
| 340 | - | Lysin | DNA polymerase; DNA binding protein | - | Transposase; Integrase; Recombinase | - | tet(K) | 1 | USFL022_ip6 |
| 341 | - | - | DNA binding protein (2) | - | Integrase; Recombinase | - | - | 1 | 2011_60_2078_5_ip4 |
| 342 | - | - | DNA polymerase | - | - | dUTPase | - | 1 | 1159_SAUR_ip6 |
| 343 | - | - | DNA polymerase; DNA binding protein | - | Integrase; Recombinase | - | - | 1 | GKP138_71_ip1 |
| 344 | - | - | DNA binding protein (2) | - | - | - | - | 1 | IF7SW_P3_ip4 |
| 345 | - | - | Single-strand binding protein; DNA binding protein (2) | - | - | dUTPase | - | 1 | M0426_ip7 |
| 346 | - | - | Single-strand binding protein; DNA polymerase | - | - | - | - | 1 | Na21_ip12 |
| 347 | - | - | DNA binding protein; DNA polymerase (2) | - | Integrase | - | - | 1 | O46_ip1 |
| 348 | - | - | Single-strand binding protein; DNA binding protein | - | - | - | - | 1 | OCMM6035_ip6 |
| 349 | - | - | DNA binding protein (2) | - | Integrase | - | - | 1 | S2398_ip3 |
| 350 | - | - | DNA binding protein (2) | - | Integrase | - | - | 1 | SA_067_ip1 |
| 351 | - | - | DNA polymerase | - | - | dUTPase | - | 1 | W45755_111412_ip12 |
| 352 | - | - | DNA binding protein (3) | - | Integrase | - | - | 1 | 81_SAUR_ip3 |
| 353 | - | - | DNA binding protein; DNA polymerase | - | Integrase | - | - | 1 | BU_W12_t13_ip5 |
| 354 | - | - | Single-strand binding protein; DNA binding protein (2) | - | Integrase; Recombinase | - | - | 1 | KINW6056_ip1 |
| 355 | - | - | DNA polymerase; DNA binding protein | - | Integrase; Recombinase | - | - | 1 | 2588STDY5748949_ip6 |
| 356 | - | - | DNA binding protein (2) | - | Integrase; Recombinase | - | - | 1 | MRSA_FKVP_ip3 |
| 357 | - | - | DNA binding protein | - | Integrase; Recombinase | - | - | 1 | MRSA_S4_ip6 |
| 358 | - | - | - | terminase small subunit | Integrase | lukH; lukG; hlb | - | 1 | 2588STDY5748949_ip9 |
| 359 | - | - | Single-strand binding protein | - | - | - | - | 1 | 91751_ip3 |
| 360 | - | - | DNA binding protein | - | Integrase | lukH; lukG; hlb | - | 1 | CUHK_HK188_ip4 |
| 361 | - | - | - | - | Integrase | lukH; lukG; hlb | arlS; arlR | 1 | ES26_ip8 |
| 362 | - | - | - | - | Integrase | lukH; lukG; hlb | - | 1 | M116_ip9 |
| 363 | - | - | - | - | Integrase | lukH; lukG; hlb | ANT(9)-Ia; ErmA | 1 | MAL11_ip8 |
| 364 | - | - | Single-strand binding protein | - | Integrase | lukH; lukG; hlb | - | 1 | MRSA_PR1_ip2 |
| 365 | - | - | - | terminase small subunit | Integrase | lukH; lukG; hlb | - | 1 | NA32_ip7 |
| 366 | - | - | DNA binding protein | - | Integrase | selq; selk; lukH; lukG; hlb | tet(M); | 1 | S2396_ip6 |
| 367 | - | - | DNA binding protein | terminase small subunit | Integrase (2) | - | - | 1 | Sa12_002_ip2 |
| 368 | - | - | Single-strand binding protein; DNA binding protein | - | Integrase | dUTPase | arlR; arlS | 1 | USFL253_ip3 |
| 369 | - | - | Single-strand binding protein | - | - | dUTPase | - | 1 | NMR02_ip6 |
| 370 | - | - | - | terminase small subunit | Integrase | lukH; lukG; hlb | - | 1 | Na21_ip6 |
| 371 | - | - | - | - | Integrase | lukH; lukG; hlb | - | 1 | DAR5877_ip4 |
| 372 | - | - | DNA binding protein (2) | - | Integrase; Transposase | adsA; lukH; lukG; hlb (2) | - | 1 | 91751_ip2 |
| 373 | - | - | Single-strand binding protein | - | - | - | - | 1 | DAR5877_ip5 |
| 374 | - | - | - | terminase small subunit (3) | Integrase | eno | - | 1 | LCT_SAO_ip4 |
| 375 | - | - | DNA binding protein | terminase small subunit (2) | Integrase | Virulence-associated protein E | - | 1 | T0131_ip5 |
| 376 | - | - | DNA binding protein | terminase small subunit | Integrase (2) | lukH; lukG; hlb; sell; sec; tsst-1 | - | 1 | Mu50_ip6 |
| 377 | - | - | DNA binding protein | Terminase small subunit | Integrase | Virulence-associated protein E | - | 1 | BU_W22_t4_ip3 |
| 378 | - | - | - | Terminase small subunit | - | Virulence-associated protein E; tsst-1 | - | 1 | SR434_ip1 |
| 379 | - | - | - | Terminase small subunit | Integrase | eno | - | 1 | 170_ip1 |
| 380 | - | - | - | Terminase small subunit | Integrase | eno; seb | - | 1 | 69_SAUR_ip2 |
| 381 | - | - | - | Terminase small subunit | Integrase | Virulence-associated protein E; eno | - | 1 | JS395_ip5 |
| 382 | - | - | - | - | Transposase (3) | eno | - | 1 | Sa14_004_ip2 |
| 383 | - | - | - | Terminase small subunit | Integrase | Virulence-associated protein E | - | 1 | LVP2_ip1 |
| 384 | - | - | - | Terminase small subunit | Integrase | Virulence-associated protein E | - | 1 | SAHPchr_ip1 |
| 385 | - | - | - | - | Integrase | sell; sec | - | 1 | 08_01483_ip1 |
| 386 | - | - | - | - | Integrase; Transposase | sspA; sspB | - | 1 | 08_01669_ip4 |
| 387 | - | - | DNA binding protein | - | Integrase | - | - | 1 | W33563_ip1 |
| 388 | - | - | - | - | Integrase | sell; sec | - | 1 | 07_02789_ip2 |
| 389 | - | - | - | - | Integrase | - | - | 1 | 04_02314_1_ip1 |
| 390 | - | - | - | - | Integrase | sec | - | 1 | BSAR486_ip2 |
| 391 | - | - | - | Terminase small subunit | Integrase | tsst-1; seb; Virulence-associated protein E | - | 1 | HOU1445_VS_ip3 |
| 392 | - | - | - | - | Integrase | - | - | 1 | M3_ip2 |
| 393 | - | - | DNA-binding protein | Terminase small subunit | - | efb; Virulence-associated protein E; | - | 1 | S2396_ip10 |
| 394 | - | - | - | - | Integrase | - | - | 1 | Sa13_006_ip3 |
| 395 | - | - | DNA-binding protein | - | Integrase (2) | - | - | 1 | GKP136_67_ip2 |
| 396 | - | - | DNA-binding protein | - | Integrase | - | - | 1 | 18754_2_ip2 |
| 397 | - | - | DNA-binding protein | - | Integrase (2) | - | - | 1 | GKP136_36_ip2 |
| 398 | - | - | DNA-binding protein | - | Integrase | eap/map | - | 1 | Sa13_005_ip3 |
| 399 | - | - | DNA-binding protein | - | Integrase | - | - | 1 | 110_ip2 |
| 400 | - | - | DNA-binding protein (2) | - | Integrase (2) | - | - | 1 | 2011_60_2078_5_ip1 |
| 401 | Phage minor structural protein | Holin; Lysin | - | - | - | - | - | 1 | M996_ip4 |
| 402 | head morphogenesis protein | - | DNA-binding protein (2) | - | Transposase (2) | - | - | 1 | 04_02314_1_ip5 |
| 403 | - | - | Type I restriction modification system | - | Transposase (2) | - | - | 1 | 06_01900_ip3 |
| 404 | - | - | DNA-binding protein (2) | - | Recombinase | - | - | 1 | APS210_ip6 |
| 405 | - | - | Type I restriction modification system | - | Transposase (2) | - | - | 1 | ARI29_ip5 |
| 406 | - | - | - | Terminase small subunit | - | eap/map; hlb; sak; scn; chp | - | 1 | BSAR729_ip4 |
| 407 | - | - | DNA-binding protein | Terminase small subunit | - | eap/map; hlb; sak; scn; chp | arsenical resistance | 1 | BSAR734_ip4 |
| 408 | head morphogenesis protein | - | - | - | - | - | - | 1 | BSAR865_ip7 |
| 409 | - | Lysin (2) | - | - | - | eap/map; hlb (2); sak; scn; chp; lukH; lukG | - | 1 | JS395_ip2 |
| 410 | - | Lysin | - | - | - | hlb; sak; scn; chp | - | 1 | RA3_ip7 |
| 411 | - | - | Type I restriction modification system | - | Transposase (2) | - | - | 1 | RH_0600_0125_09_ip3 |
| 412 | - | - | Type I restriction modification system | - | Recombinase | - | - | 1 | SA_ST125_MupR_ip2 |
| 413 | - | - | Type I restriction modification system | - | Recombinase; Transposase | - | mecA | 1 | 71A_S11_ip5 |
| 414 | - | - | - | - | Recombinase (2); Transposase (2) | - | blaZ; Cadmium resistance; Arsenical Resistance | 1 | 125_ip4 |
| 415 | - | - | Type I restriction modification system | - | Recombinase (2) | - | - | 1 | BSAR739_ip4 |
| 416 | - | - | - | - | Recombinase; Transposase | - | mecA | 1 | DAR948_ip5 |
| 417 | - | - | - | - | Transposase (3); | - | APH(3')-IIIa; aad(6); msrA; mphC; blaZ | 1 | DAR4145_ip4 |
| 418 | - | - | - | - | Recombinase | - | - | 1 | H914_91_ip6 |
| 419 | - | - | type I restriction-modification system | - | Recombinase (3); Transposase | - | arsenical resistance | 1 | JS395_ip3 |
| 420 | - | - | DNA helicase | - | Recombinase (2); Transposase (3) | - | mecA; dfrC | 1 | MRSA140_ip3 |
| 421 | - | - | Type I restriction modification system | - | Recombinase | adsA | - | 1 | st1815_ip3 |
| 422 | - | - | Type I restriction modification system | - | Recombinase (2) | - | - | 1 | st1831_ip1 |
| 423 | - | - | Type I restriction modification system | - | Recombinase | - | arsenical resistance | 1 | M1216_ip3 |
| 424 | - | - | - | - | Transposase (2) | - | - | 1 | 07_03339_ip3 |
| 425 | - | - | - | - | Transposase | efb; hla | - | 1 | 07_03349_ip2 |
| 426 | head morphogenesis protein | - | - | - | Integrase; Transposase (3) | - | - | 1 | BSAR77_ip4 |
| 427 | - | - | DNA-binding protein | - | Integrase; Transposase | - | - | 1 | CA_347_ip2 |
| 428 | - | - | - | - | Transposase (2) | - | - | 1 | CA_347_ip5 |
| 429 | - | - | - | - | Integrase | efb (2); hla | - | 1 | H_EMRSA_15_ip2 |
| 430 | - | - | - | - | Integrase; Transposase | - | - | 1 | H_EMRSA_15_ip4 |
| 431 | - | - | - | - | Integrase | - | - | 1 | R0294_ip4 |
| 432 | - | - | DNA-binding protein | - | Integrase | - | - | 1 | GKP136_74_ip1 |
| 433 | - | - | DNA-binding protein | - | Integrase (2) | - | - | 1 | DEU16_ip2 |
| 434 | - | - | - | - | Integrase | ebh | - | 1 | MU4_ip2 |
| 435 | - | - | - | - | Integrase (2) | - | - | 1 | OC3_ip1 |
| 436 | - | - | - | - | Integrase | - | tet(38) | 1 | DEU16_ip4 |
| 437 | - | - | DNA-binding protein | - | Integrase (2) | - | - | 1 | M278_ip2 |
| 438 | Phage tail protein; Phage capsid protein; Phage head-tail joining protein; Phage tail tube protein | Lysin | - | - | Recombinase | - | - | 1 | W45755_111412_ip5 |
| 439 | Phage tail protein; Phage capsid protein; Phage head-tail joining protein; Phage tail tube protein; Phage portal protein | Lysin | DNA-binding protein | DNA packaging protein | Recombinase; Integrase | hlb (2); sak; scn; chp; selp; lukH; lukG; dUTPase | - | 1 | DAR1090_ip2 |
| 440 | Phage capsid protein; Phage head-tail joining protein; Phage tail tube protein; Phage portal protein | Lysin | - | DNA packaging protein | - | dUTPase | - | 1 | HOU1445_VS_ip1 |
| 441 | - | Lysin | - | - | Recombinase | selp; sak; scn; chp; hlb | - | 1 | HOU1445_VS_ip5 |
| 442 | - | - | - | - | - | sak; scn; chp; hlb | - | 1 | W45755_111412_ip7 |
| 443 | Phage portal protein | - | - | DNA packaging protein | - | - | - | 1 | W45755_111412_ip8 |
| 444 | Phage tail protein; Phage tail tube protein; Phage head-tail joining protein; Phage capsid protein; Phage portal protein | Lysin | - | DNA packaging protein | Recombinase | - | - | 1 | M35954_ip1 |
| 445 | - | Lysin | - | - | - | clfA; sak; sea | - | 1 | MRGR3_ip2 |
| 446 | Phage head-tail joining protein; Phage head-tail connector protein; Phage portal protein | - | - | Terminase small subunit | - | - | - | 1 | MRSA_FKVP_ip4 |
| 447 | Phage portal protein;Phage minor structural protein; Phage head-tail connector protein; Phage tail-component; Phage tail assembly chaperone protein; Phage tail protein | Lysin | - | DNA packaging protein; Terminase small subunit | - | dUTPase | - | 1 | GKP136_12_ip4 |
| 448 | Phage tail protein; Phage tail assembly chaperone protein; Phage tail-component; Phage head-tail connector protein; Phage minor structural protein; Phage portal protein | - | - | DNA packaging protein; Terminase small subunit | - | - | - | 1 | GKP138_17_ip6 |
| 449 | Phage tail tape measure protein; Phage tail protein | Lysin | - | - | - | - | - | 1 | MAL9_ip6 |
| 450 | Phage portal protein; head morphogenesis protein; Phage tail-component; Phage head-tail connector protein; Phage tail tube protein; Phage tail assembly chaperone protein; Phage tail tape measure protein; Phage tail protein | - | - | Terminase small subunit | - | isdA; isdB; isdC; isdD; isdE | - | 1 | SA_120_ip3 |
| 451 | - | Lysin; Holin | DNA helicase | - | Transposase | dUTPase | tet(45) | 1 | M592_ip11 |
| 452 | - | - | - | - | Transposase | sak; scn; eap/map; hlb | - | 1 | MU4_ip7 |
| 453 | - | - | - | - | Transposase (2) | eap/map; hlb | - | 1 | MU4_ip8 |
| 454 | - | Lysin | - | - | - | - | tet(45); tet(K) | 1 | S2396_ip8 |
| 455 | - | - | - | - | Integrase; Transposase (2) | - | - | 1 | R0615_ip3 |
| 456 | Phage portal protein; Phage head-tail joining protein; Phage tail tube protein | - | - | DNA packaging protein | - | dUTPase | - | 1 | st1831_ip4 |
| 457 | Phage tail protein; Phage tail tube protein; Phage head-tail joining protein; Phage capsid protein; Phage portal protein | Lysin | - | DNA packaging protein | - | dUTPase | - | 1 | Tur_22_ip3 |
| 458 | Phage portal protein; Phage capsid protein; Phage head-tail joining protein; Phage tail tube protein; Phage tail protein | Lysin (2) | DNA-binding protein (2); Single-strand binding protein | DNA packaging protein | Integrase | lukS-PV; lukF-PV; dUTPase; sak | - | 1 | M1_LIDQ_ip1 |
| 459 | Phage tail tube protein; Phage head-tail joining protein; Phage capsid protein; Phage portal protein | - | - | DNA packaging protein | - | dUTPase | - | 1 | S2398_ip4 |
| 460 | Phage portal protein; Phage capsid protein; Phage head-tail joining protein; Phage tail tube protein | Lysin | DNA polymerase | DNA packaging protein | - | dUTPase | - | 1 | S2397_ip3 |
| 461 | Phage tail protein; Phage tail tube protein; Phage head-tail joining protein; Phage capsid protein; Phage portal protein | Lysin | - | DNA packaging protein | Recombinase; Clp protease | - | - | 1 | P32_ip8 |
| 462 | Phage tail protein | Lysin | - | - | Recombinase | sak; scn; chp; hlb | - | 1 | DAR3179_ip5 |
| 463 | Phage tail tube protein; Phage head-tail joining protein; Phage capsid protein; Phage portal protein | - | - | DNA packaging protein | - | - | - | 1 | DAR3179_ip6 |
| 464 | - | Lysin | type I restriction-modification system; DNA-binding protein (3) | - | - | sak; sdrD (2); sdrE; sdrF | Arsenical Resistance; Cadmium resistance | 1 | 91751_ip5 |
| 465 | Phage tail protein; Phage tail tape measure protein; Phage tail tube protein; Phage head-tail joining protein; Phage capsid protein; Phage portal protein | Lysin | - | DNA packaging protein | - | sak; scn; chp; dUTPase | - | 1 | 043H_ip6 |
| 466 | Phage portal protein | - | - | DNA packaging protein | - | dUTPase | - | 1 | FKWK_ip7 |
| 467 | Phage tail tape measure protein; Phage tail tube protein; Phage head-tail joining protein; Phage capsid protein; Phage portal protein | - | - | DNA packaging protein | Integrase | dUTPase | - | 1 | MRSA_CVM43477_ip1 |
| 468 | Phage tail tape measure protein; Phage tail tube protein; Phage head-tail joining protein; Phage capsid protein; Phage portal protein | - | DNA-binding protein (3); Single-strand binding protein | DNA packaging protein | Integrase | hlb; lukH; lukG; dUTPase | - | 1 | IF6SW_P2_RA_ip3 |
| 469 | Phage tail tape measure protein; Phage tail tube protein; Phage head-tail joining protein; Phage capsid protein; Phage portal protein; Phage tail protein | Lysin | DNA-binding protein; Single-strand binding protein | DNA packaging protein | - | dUTPase; sak; scn; chp; hlb | - | 1 | BU_G0201_t8_ip4 |
| 470 | Phage tail tape measure protein; Phage tail tube protein; Phage head-tail joining protein; Phage capsid protein; Phage portal protein | - | - | DNA packaging protein | - | - | - | 1 | MUM270_ip3 |
| 471 | Phage tail tape measure protein; Phage tail tube protein; Phage head-tail joining protein; Phage capsid protein; Phage portal protein | - | DNA-binding protein (2); Single-strand binding protein | DNA packaging protein | Recombinase | - | - | 1 | H202_ip4 |
| 472 | Phage tail tape measure protein; Phage tail tube protein; Phage head-tail joining protein; Phage capsid protein; Phage portal protein; Phage tail protein | Lysin | - | DNA packaging protein | Recombinase | sak; scn; chp; hlb | - | 1 | CUHK_HK188_ip5 |
| 473 | Phage tail tape measure protein; Phage tail tube protein; Phage head-tail joining protein; Phage capsid protein; Phage portal protein; Phage tail protein | Lysin | Single-strand binding protein | DNA packaging protein | Clp protease | sak; scn; chp; hlb; dUTPase | - | 1 | 2588STDY5748949_ip8 |
| 474 | Phage tail tape measure protein; Phage tail tube protein; Phage head-tail joining protein; Phage capsid protein; Phage portal protein; Phage tail protein | - | type I restriction-modification system | DNA packaging protein | Clp protease; Recombinase | Type IV toxin-antitoxin system; SplA; SplB; SplC; SplD; sea | - | 1 | MU4_ip3 |
| 475 | Phage tail tube protein; Phage tail protein | - | DNA methyltransferase; DNA helicase; Single-strand binding protein; Type I restriction modification | - | - | set40 | - | 1 | M116_ip11 |
| 476 | head morphogenesis protein (2) | Lysin | type I restriction-modification system | - | - | hlb | - | 1 | M278_ip5 |
| 477 | head morphogenesis protein (3) | - | - | - | - | sak; scn; chp; hlb | - | 1 | 09_01245_ip4 |
| 478 | - | - | - | - | - | sak; scn; chp; hlb | - | 1 | 07_00059_ip4 |
| 479 | Phage portal protein; Phage head-tail joining protein; Phage tail tube protein; Phage tail protein | Lysin | - | DNA packaging protein | Clp protease | - | - | 1 | PB1_1_ip3 |
| 480 | Phage tail tape measure protein; Phage tail protein | - | - | - | Transposase | - | - | 1 | H202_ip9 |
| 481 | - | - | type I restriction-modification system | - | Transposase | SplA; SplB; SplC; SplD; SplE; SplF; lukD; lukE | - | 1 | JS395_ip1 |
| 482 | - | - | - | - | Transposase (2) | - | - | 1 | 2588STDY5627534_ip4 |
| 483 | - | - | - | - | - | SplA; SplB; SplC; lukD; lukE | - | 1 | KLT6_ip3 |
| 484 | - | - | DNA-binding protein | - | Integrase | Type IV Toxin-Antitoxin system; SplA; SplB; SplC | - | 1 | M116_ip10 |
| 485 | - | - | - | - | - | lukD; lukE | - | 1 | MUM475_ip3 |
| 486 | - | Lysin; Holin | - | - | - | lukD; lukE | - | 1 | S2396_ip2 |
| 487 | - | - | - | - | - | Type IV Toxin-Antitoxin system; SplA; SplB; SplC | - | 1 | Sa12_002_ip3 |
| 488 | - | - | type I restriction-modification system | - | - | SplA; SplB; SplC; SplD; SplE; SplF; lukD; lukE | - | 1 | SA_083_ip1 |
| 489 | - | - | DNA-binding protein | - | Integrase | lukD; lukE | - | 1 | Sa13_005_ip1 |
| 490 | - | - | - | - | Integrase | SplA; SplB; SplC; SplD; lukD; lukE | - | 1 | 1322_2_SAUR_ip1 |
| 491 | - | - | type I restriction-modification system | - | - | SplA; SplB; SplC; SplD; SplE; SplF; lukD | - | 1 | USA300_TCH959_ip4 |
| 492 | - | - | - | - | Transposase | seg; sen; sei; sem; seo; seu | AAC6_Ie_APH2_Ia | 1 | BA01611_ip5 |
| 493 | - | - | - | - | Transposase (2) | SplA; SplB; SplC; lukD; lukE; seg; sen; yent1; yent2; sei; sem (2); seo | AAC6_Ie_APH2_Ia (2) | 1 | DAR5889_ip4 |
| 494 | - | - | DNA polymerase | - | - | seg; sen; sei; sem; seo; seu | - | 1 | BSAR734_ip2 |
| 495 | - | - | - | - | Transposase | seg; sen; sei; sem; seo; seu | - | 1 | 08_01667_ip3 |
| 496 | - | - | type I restriction-modification system | - | Transposase (2) | seg; sen; sei; sem; seo; seu | - | 1 | 07_02088_ip4 |
| 497 | - | - | type I restriction-modification system | - | Transposase | seg; sen; sei; sem; seo; seu | - | 1 | 07_03339_ip4 |
| 498 | - | - | DNA polymerase; DNA-binding protein | - | - | seg; sen; sei; sem; seo; seu | - | 1 | 07_03349_ip3 |
| 499 | - | - | type I restriction-modification system | - | Transposase | seg; sen; sei; sem; seo; seu | - | 1 | 07_00655_ip4 |
| 500 | - | - | - | - | Transposase (3) | seg; sen; sei; sem; seo; seu | - | 1 | 08_01669_ip5 |
| 501 | - | - | DNA polymerases | - | Transposase | seg; sen; sei; sem; seo; seu | - | 1 | H914_91_ip2 |
| 502 | head morphogenesis protein (3) | - | - | - | - | seg; sen; sei; sem; seo; seu | - | 1 | ARI29_ip3 |
| 503 | head morphogenesis protein (3) | - | - | - | Transposase | - | - | 1 | 08_01668_ip4 |
| 504 | head morphogenesis protein | - | - | - | Transposase | - | - | 1 | BSAR486_ip4 |
| 505 | head morphogenesis protein | - | type I restriction-modification system | - | Transposase | - | - | 1 | BSAR748_ip3 |
| 506 | head morphogenesis protein (2) | - | - | - | - | - | - | 1 | H202_ip5 |
| 507 | head morphogenesis protein (3) | - | - | - | Transposase | - | AAC6_Ie_APH2_Ia; ANT(6)-Ia | 1 | HU8_ip7 |
| 508 | - | - | DNA-binding protein | - | - | set35; dUTPase | dfrC; ANT(6)-Ia; AAC6_Ie_APH2_Ia | 1 | MRGR3_ip13 |
| 509 | head morphogenesis protein (5) | - | - | - | - | - | - | 1 | 71A_S11_ip1 |
| 510 | - | - | - | - | Integrase (2) | - | - | 1 | M0513_ip2 |
| 511 | head morphogenesis protein (3) | - | - | - | Integrase (2) | - | - | 1 | NGS_ED_1006_ip1 |
| 512 | head morphogenesis protein (5) | - | - | - | Integrase | - | - | 1 | 1943STDY5698363_ip3 |
| 513 | head morphogenesis protein (5) | - | - | - | Integrase (2) | - | - | 1 | SA_260_ip5 |
| 514 | head morphogenesis protein (3) | - | - | - | Integrase (2) | - | - | 1 | st2543_ip4 |
| 515 | - | - | - | - | Integrase | dUTPase | - | 1 | M1169_ip3 |
| 516 | head morphogenesis protein (2); Phage portal protein; Phage head-tail connector protein; Phage tail-component; Phage tail tube protein; Phage tail assembly chaperone protein; Phage tail protein | Holin | DNA-binding protein | Phage terminase large subunit | Integrase; Transposase | - | - | 1 | M1216_ip1 |
| 517 | head morphogenesis protein (3); Phage portal protein | - | - | Phage terminase large subunit | Integrase; Transposase | - | - | 1 | Mq2T_ip1 |
| 518 | Phage tail tape measure protein; Phage capsid protein (2); portal protein | - | - | - | Clp protease | - | - | 1 | 10S_ip4 |
| 519 | head morphogenesis protein; Phage portal protein | - | DNA-binding protein | DNA packaging protein | Transposase | - | - | 1 | 922_SAUR_ip4 |
| 520 | portal protein; Phage capsid protein; Phage tail tape measure protein | Holin | - | - | Clp protease | dUTPase | - | 1 | APS210_ip7 |
| 521 | portal protein; Phage capsid protein; Phage tail tape measure protein | Holin | - | - | Clp protease | - | - | 1 | M1169_ip2 |
| 522 | - | - | DNA-binding protein | - | Integrase | - | - | 1 | st1332_ip1 |

Table S6. Comparison of the antibiotic resistance genes and virulence factors among the S. aureus isolates, intact prophages and incomplete prophages.

| **Genes** | ***S. aureus*** | **Intact prophages** | **Incomplete prophages** |
| --- | --- | --- | --- |
| **Antibiotic resistance genes** |  |  |  |
| *blaZ* | + | + | + |
| *mecA* | + | + | + |
| *mecC* | + | - | - |
| *TEM-206* | + | - | - |
| *TEM-183* | + | - | - |
| *CTX-M-105* | + | - | - |
| *SHV-155* | + | - | - |
| *AAC(3)-IV* | + | - | - |
| *AAC(6')-Ib10* | + | - | - |
| *AAC(6')-Ib8* | + | - | - |
| *aadA* | + | - | - |
| *aadA8b* | + | - | - |
| *aadA23* | + | - | - |
| *aadA24* | + | - | - |
| *aadA25* | + | - | - |
| *AAC6_Ie_APH2_Ia* | + | + | + |
| *aad(6)* | + | + | + |
| *APH(3')-IIa* | + | - | - |
| *ANT(4')-Ib* | + | + | + |
| *ANT(6)-Ia* | + | + | + |
| *ANT(9)-Ia* | + | + | + |
| *APH(2'')-If* | + | - | - |
| *APH(3')-IIIa* | + | + | + |
| *APH(6)-Id* | + | - | - |
| *spd* | + | - | - |
| *arlR* | + | - | + |
| *arlS* | + | - | + |
| *qacA* | + | + | - |
| *qacB* | + | + | - |
| *qacJ* | + | + | + |
| *dfrC* | + | + | + |
| *dfrG* | + | + | + |
| *dfrK* | + | - | - |
| *fexA* | + | - | - |
| *fexB* | + | - | - |
| *floR* | + | - | - |
| *mexN* | + | - | - |
| *catA8* | + | - | - |
| *cat-TC* | + | - | + |
| *Sint_ACT_CHL* | + | - | - |
| *Efae_ACT_CHL* | + | - | - |
| *FosB* | + | - | - |
| *FosD* | + | - | - |
| *FosY* | + | - | - |
| *fusB* | + | - | - |
| *fusC* | + | - | + |
| *lnuA* | + | - | - |
| *lnuB* | + | - | - |
| *lnuG* | + | - | - |
| *tet(38)* | + | + | + |
| *tet(45)* | + | + | + |
| *tet(A)* | + | - | - |
| *tet(K)* | + | + | + |
| *tet(L)* | + | - | - |
| *tet(M)* | + | + | + |
| *emrY* | + | - | - |
| *mphC* | + | - | + |
| *sul2* | + | - | - |
| *apmA* | + | - | - |
| *mef(D)* | + | - | - |
| *SAT-4* | + | + | - |
| *ErmB* | + | - | - |
| *Erm(33)* | + | + | - |
| *ErmA* | + | + | + |
| *ErmC* | + | + | - |
| *ErmT* | + | + | - |
| *lsaE* | + | - | - |
| *mepA* | + | + | + |
| *mepR* | + | + | - |
| *mgrA* | + | - | - |
| *msrA* | + | - | + |
| *msrE* | + | - | - |
| *msrF* | + | - | - |
| *oqxB* | + | - | - |
| *norA* | + | - | - |
| *norC* | + | + | - |
| *LmrS* | + | + | + |
| *sdrM* | + | + | + |
| *sepA* | + | + | + |
| *vgaALC* | + | - | - |
| *vgaE* | + | - | - |
| *acrB* | + | - | - |
| *adeF* | + | - | - |
| *mdtM* | + | - | - |
| *Ecol_acrA* | + | - | - |
| **Virulence factors** |  |  |  |
| *aaa* | + | - | - |
| *cna* | + | - | + |
| *eap* | + | + | + |
| *ebp* | + | + | + |
| *efaA* | + | - | - |
| *emp* | + | + | + |
| *map* | + | + | + |
| *clfA* | + | + | + |
| *clfB* | + | + | - |
| *fnbA* | + | + | + |
| *fnbB* | + | + | + |
| *atl* | + | + | - |
| *eap/map* | + | + | + |
| *flp* | + | - | - |
| *sasA* | + | - | + |
| *sdrC* | + | + | + |
| *sdrD* | + | + | + |
| *sdrE* | + | + | + |
| *sdrG* | + | - | - |
| *tuf* | + | - | - |
| *cap8* | + | - | - |
| *capA* | + | - | - |
| *capN* | + | - | - |
| *chp* | + | + | + |
| *ebh* | + | + | + |
| *efb* | + | - | + |
| *SAOUHSC_00129* | + | - | - |
| *SACOL_RS00730* | + | + | + |
| *SAV_RS00940* | + | - | - |
| *SAUSA300_RS00855* | + | - | - |
| *sbi* | + | + | + |
| *sbnA* | + | - | - |
| *sbnB* | + | - | - |
| *sbnC* | + | - | - |
| *sbnD* | + | - | - |
| *sbnE* | + | - | - |
| *sbnF* | + | - | - |
| *sbnG* | + | - | - |
| *sbnH* | + | - | - |
| *sbnI* | + | - | - |
| *scn* | + | + | + |
| *SH_RS01820* | + | - | - |
| *SSP_RS00305* | + | - | - |
| *wecB* | + | - | - |
| *adsA* | + | + | + |
| *coa* | + | - | - |
| *eno* | + | + | + |
| *eta* | + | + | - |
| *geh* | + | + | + |
| *hysA* | + | + | + |
| *sak* | + | + | + |
| *lip* | + | - | + |
| *splA* | + | + | + |
| *splB* | + | + | + |
| *splC* | + | + | + |
| *splD* | + | + | + |
| *splE* | + | + | + |
| *splF* | + | + | + |
| *sspA* | + | + | + |
| *sspB* | + | + | + |
| *sspC* | + | + | - |
| *vWbp* | + | + | + |
| *aur* | + | + | - |
| *esaA* | + | - | - |
| *esaB* | + | - | - |
| *esaD* | + | - | - |
| *esaE* | + | - | - |
| *esaG* | + | - | + |
| *essA* | + | - | - |
| *essB* | + | - | - |
| *essC* | + | - | - |
| *esxA* | + | - | - |
| *esxB* | + | - | - |
| *esxC* | + | - | - |
| *esxD* | + | - | - |
| *tssL* | + | - | - |
| *harA* | + | - | - |
| *isdA* | + | + | + |
| *isdB* | + | + | + |
| *isdC* | + | + | + |
| *isdD* | + | + | + |
| *isdE* | + | + | + |
| *isdF* | + | + | - |
| *isdG* | + | + | - |
| *isdI* | + | - | - |
| *sfaA* | + | + | + |
| *sfaB* | + | + | + |
| *sfaC* | + | + | + |
| *sfaD* | + | - | + |
| *sirA* | + | - | - |
| *sirB* | + | - | - |
| *sirC* | + | - | - |
| *srtB* | + | + | - |
| *hlb* | + | + | + |
| *hld* | + | + | + |
| *hlgA* | + | + | + |
| *hlgC* | + | + | + |
| *hlgB* | + | + | + |
| *hla* | + | + | + |
| *lukD* | + | + | + |
| *lukE* | + | + | + |
| *lukF* | + | + | + |
| *lukG* | + | + | + |
| *lukH* | + | + | + |
| *lukM* | + | + | + |
| *lukS-PV* | + | + | + |
| *lukS-R* | + | + | + |
| *edin-B* | + | - | - |
| *yent1* | + | + | + |
| *yent2* | + | + | + |
| *nuc* | + | + | + |
| *sea* | + | + | + |
| *seb* | + | + | + |
| *sec* | + | + | + |
| *seg* | + | + | + |
| *seh* | + | - | + |
| *sei* | + | + | + |
| *sej* | + | - | - |
| *selk* | + | + | + |
| *sell* | + | + | + |
| *selm* | + | + | + |
| *seln* | + | + | + |
| *selo* | + | + | + |
| *selp* | + | + | + |
| *selq* | + | + | + |
| *selu* | + | + | + |
| *set3* | + | + | + |
| *set4* | + | - | + |
| *set5* | + | - | + |
| *set6* | + | - | - |
| *set7* | + | - | + |
| *set8* | + | + | + |
| *set9* | + | + | + |
| *set10* | + | + | + |
| *set20* | + | + | + |
| *spa* | + | - | + |
| *tsst-1* | + | + | + |
| *icaR* | + | + | - |
| *icaA* | + | + | + |
| *icaB* | + | + | + |
| *icaC* | + | + | + |
| *icaD* | + | + | - |
| *cupB5* | + | - | - |
| *sasC* | + | - | - |
| *clpP* | + | - | - |
| *acrB* | + | - | - |

Table S7. Comparison of the distribution of antibiotic resistance genes and virulence factors among the 16 lytic phages, 148 intact prophages and 522 incomplete prophages.

| **Genes** | **Lytic phages (n=16)** | | **Intact prophages (n=148)** | | **Incomplete prophages (n=522)** | | ***p*-Value** | ***rec*+ (n=115)** | | ***rec*- (n=571)** | | ***p*-Value** | ***tnp*+ (n=119)** | | ***tnp*- (n=567)** | | ***p*-Value** | ***int*+ (n=292)** | | ***int*- (n=394)** | | ***p*-Value** |
| --- | --- | --- | --- | --- | --- | --- | --- | --- | --- | --- | --- | --- | --- | --- | --- | --- | --- | --- | --- | --- | --- | --- |
|  | **No.** | **Percentage** | **No.** | **Percentage** | **No.** | **Percentage** |  | **No.** | **Percentage** | **No.** | **Percentage** |  | **No.** | **Percentage** | **No.** | **Percentage** |  | **No.** | **Percentage** | **No.** | **Percentage** |  |
| **Virulence factors** | | | |  |  |  |  |  |  |  |  |  |  |  |  |  |  |  |  |  |  |  |
| *cna* | 0 | 0.0% | 0 | 0.0% | 1 | 0.2% | 0.593 | 0 | 0.0% | 1 | 0.2% | 0.654 | 0 | 0.0% | 1 | 0.2% | 0.647 | 1 | 0.3% | 0 | 0.0% | 0.246 |
| *ebp* | 0 | 0.0% | 6 | 4.1% | 2 | 0.4% | 0.005 | 4 | 3.5% | 4 | 0.7% | 0.011 | 1 | 0.8% | 7 | 1.2% | 0.716 | 6 | 2.1% | 2 | 0.5% | 0.062 |
| *emp* | 0 | 0.0% | 0 | 0.0% | 3 | 0.6% | 0.353 | 0 | 0.0% | 3 | 0.5% | 0.437 | 0 | 0.0% | 3 | 0.5% | 0.427 | 2 | 0.7% | 1 | 0.3% | 0.398 |
| *map* | 0 | 0.0% | 1 | 0.7% | 0 | 0.0% | 0.132 | 1 | 0.9% | 0 | 0.0% | 0.026 | 1 | 0.8% | 0 | 0.0% | 0.029 | 0 | 0.0% | 1 | 0.3% | 0.390 |
| *clfA* | 0 | 0.0% | 1 | 0.7% | 4 | 0.8% | 0.775 | 0 | 0.0% | 5 | 0.9% | 0.315 | 0 | 0.0% | 5 | 0.9% | 0.305 | 3 | 1.0% | 2 | 0.5% | 0.429 |
| *fnbA* | 0 | 0.0% | 5 | 3.4% | 3 | 0.6% | 0.035 | 2 | 1.7% | 6 | 1.1% | 0.531 | 8 | 6.7% | 0 | 0.0% | 0.000 | 0 | 0.0% | 8 | 2.0% | 0.014 |
| *fnbB* | 0 | 0.0% | 3 | 2.0% | 3 | 0.6% | 0.234 | 2 | 1.7% | 4 | 0.7% | 0.276 | 4 | 3.4% | 2 | 0.4% | 0.001 | 0 | 0.0% | 6 | 1.5% | 0.034 |
| *atl* | 0 | 0.0% | 1 | 0.7% | 0 | 0.0% | 0.132 | 0 | 0.0% | 1 | 0.2% | 0.654 | 1 | 0.8% | 0 | 0.0% | 0.029 | 1 | 0.3% | 0 | 0.0% | 0.246 |
| *eap/map* | 0 | 0.0% | 7 | 4.7% | 8 | 1.5% | 0.103 | 2 | 1.7% | 13 | 2.3% | 0.720 | 5 | 4.2% | 10 | 1.8% | 0.099 | 8 | 2.7% | 7 | 1.8% | 0.394 |
| *sasA* | 0 | 0.0% | 0 | 0.0% | 1 | 0.2% | 0.593 | 0 | 0.0% | 1 | 0.2% | 0.654 | 1 | 0.8% | 0 | 0.0% | 0.029 | 1 | 0.3% | 0 | 0.0% | 0.246 |
| *sdrC* | 0 | 0.0% | 5 | 3.4% | 4 | 0.8% | 0.071 | 1 | 0.9% | 8 | 1.4% | 0.648 | 4 | 3.4% | 5 | 0.9% | 0.031 | 3 | 1.0% | 6 | 1.5% | 0.573 |
| *sdrD* | 0 | 0.0% | 8 | 5.4% | 8 | 1.5% | 0.050 | 1 | 0.9% | 15 | 2.6% | 0.255 | 8 | 6.7% | 8 | 1.4% | 0.000 | 5 | 1.7% | 11 | 2.8% | 0.355 |
| *sdrE* | 0 | 0.0% | 1 | 0.7% | 1 | 0.2% | 0.493 | 0 | 0.0% | 2 | 0.4% | 0.526 | 0 | 0.0% | 2 | 0.4% | 0.517 | 1 | 0.3% | 1 | 0.3% | 0.832 |
| *chp* | 0 | 0.0% | 5 | 3.4% | 21 | 4.0% | 0.458 | 5 | 4.3% | 21 | 3.7% | 0.732 | 2 | 1.7% | 24 | 4.2% | 0.186 | 5 | 1.7% | 21 | 5.3% | 0.014 |
| *ebh* | 0 | 0.0% | 2 | 1.4% | 7 | 1.3% | 0.805 | 1 | 0.9% | 8 | 1.4% | 0.648 | 3 | 2.5% | 6 | 1.1% | 0.203 | 5 | 1.7% | 4 | 1.0% | 0.428 |
| *sbi* | 0 | 0.0% | 0 | 0.0% | 1 | 0.2% | 0.593 | 1 | 0.9% | 0 | 0.0% | 0.026 | 0 | 0.0% | 1 | 0.2% | 0.647 | 0 | 0.0% | 1 | 0.3% | 0.390 |
| *scn* | 0 | 0.0% | 10 | 6.8% | 27 | 5.2% | 0.920 | 10 | 8.7% | 27 | 4.7% | 0.086 | 4 | 3.4% | 33 | 5.8% | 0.281 | 10 | 3.4% | 27 | 6.9% | 0.049 |
| *adsA* | 0 | 0.0% | 1 | 0.7% | 2 | 0.4% | 0.802 | 2 | 1.7% | 1 | 0.2% | 0.020 | 2 | 1.7% | 1 | 0.2% | 0.024 | 1 | 0.3% | 2 | 0.5% | 0.746 |
| *eno* | 0 | 0.0% | 3 | 2.0% | 8 | 1.5% | 0.944 | 0 | 0.0% | 11 | 1.9% | 0.134 | 4 | 3.4% | 7 | 1.2% | 0.093 | 8 | 2.7% | 3 | 0.8% | 0.041 |
| *eta* | 0 | 0.0% | 1 | 0.7% | 0 | 0.0% | 0.132 | 0 | 0.0% | 1 | 0.2% | 0.654 | 0 | 0.0% | 1 | 0.2% | 0.647 | 0 | 0.0% | 1 | 0.3% | 0.390 |
| *geh* | 0 | 0.0% | 2 | 1.4% | 11 | 2.1% | 0.421 | 0 | 0.0% | 13 | 2.3% | 0.103 | 0 | 0.0% | 13 | 2.3% | 0.096 | 12 | 4.1% | 1 | 0.3% | 0.000 |
| *hysA* | 0 | 0.0% | 0 | 0.0% | 3 | 0.6% | 0.353 | 1 | 0.9% | 2 | 0.4% | 0.442 | 1 | 0.8% | 2 | 0.4% | 0.464 | 2 | 0.7% | 1 | 0.3% | 0.398 |
| *sak* | 0 | 0.0% | 11 | 7.4% | 31 | 5.9% | 0.995 | 11 | 9.6% | 31 | 5.4% | 0.092 | 4 | 3.4% | 38 | 6.7% | 0.167 | 14 | 4.8% | 28 | 7.1% | 0.212 |
| *lip* | 0 | 0.0% | 0 | 0.0% | 1 | 0.2% | 0.593 | 0 | 0.0% | 1 | 0.2% | 0.654 | 0 | 0.0% | 1 | 0.2% | 0.647 | 0 | 0.0% | 1 | 0.3% | 0.390 |
| *splA* | 0 | 0.0% | 8 | 5.4% | 18 | 3.4% | 0.631 | 1 | 0.9% | 25 | 4.4% | 0.072 | 5 | 4.2% | 21 | 3.7% | 0.796 | 7 | 2.4% | 19 | 4.8% | 0.100 |
| *splB* | 0 | 0.0% | 9 | 6.1% | 25 | 4.8% | 0.978 | 4 | 3.5% | 30 | 5.3% | 0.424 | 9 | 7.6% | 25 | 4.4% | 0.150 | 7 | 2.4% | 27 | 6.9% | 0.008 |
| *splC* | 0 | 0.0% | 9 | 6.1% | 25 | 4.8% | 0.978 | 4 | 3.5% | 30 | 5.3% | 0.424 | 9 | 7.6% | 25 | 4.4% | 0.150 | 7 | 2.4% | 27 | 6.9% | 0.008 |
| *splD* | 0 | 0.0% | 8 | 5.4% | 19 | 3.6% | 0.714 | 4 | 3.5% | 23 | 4.0% | 0.782 | 10 | 8.4% | 17 | 3.0% | 0.006 | 6 | 2.1% | 21 | 5.3% | 0.029 |
| *splE* | 0 | 0.0% | 8 | 5.4% | 14 | 2.7% | 0.325 | 1 | 0.9% | 21 | 3.7% | 0.119 | 9 | 7.6% | 13 | 2.3% | 0.003 | 5 | 1.7% | 17 | 4.3% | 0.056 |
| *splF* | 0 | 0.0% | 8 | 5.4% | 16 | 3.1% | 0.471 | 2 | 1.7% | 22 | 3.9% | 0.261 | 11 | 9.2% | 13 | 2.3% | 0.000 | 6 | 2.1% | 18 | 4.6% | 0.077 |
| *sspA* | 0 | 0.0% | 0 | 0.0% | 1 | 0.2% | 0.593 | 0 | 0.0% | 1 | 0.2% | 0.654 | 1 | 0.8% | 0 | 0.0% | 0.029 | 1 | 0.3% | 0 | 0.0% | 0.246 |
| *sspB* | 0 | 0.0% | 0 | 0.0% | 1 | 0.2% | 0.593 | 0 | 0.0% | 1 | 0.2% | 0.654 | 1 | 0.8% | 0 | 0.0% | 0.029 | 1 | 0.3% | 0 | 0.0% | 0.246 |
| *vwb* | 0 | 0.0% | 1 | 0.7% | 2 | 0.4% | 0.802 | 0 | 0.0% | 3 | 0.5% | 0.437 | 1 | 0.8% | 2 | 0.4% | 0.464 | 2 | 0.7% | 1 | 0.3% | 0.398 |
| *esaG* | 0 | 0.0% | 1 | 0.7% | 0 | 0.0% | 0.132 | 0 | 0.0% | 1 | 0.2% | 0.654 | 1 | 0.8% | 0 | 0.0% | 0.029 | 0 | 0.0% | 1 | 0.3% | 0.390 |
| *isdA* | 0 | 0.0% | 1 | 0.7% | 0 | 0.0% | 0.132 | 1 | 0.9% | 0 | 0.0% | 0.026 | 0 | 0.0% | 1 | 0.2% | 0.647 | 0 | 0.0% | 1 | 0.3% | 0.390 |
| *isdB* | 0 | 0.0% | 1 | 0.7% | 1 | 0.2% | 0.493 | 1 | 0.9% | 1 | 0.2% | 0.208 | 0 | 0.0% | 2 | 0.4% | 0.517 | 0 | 0.0% | 2 | 0.5% | 0.223 |
| *isdC* | 0 | 0.0% | 1 | 0.7% | 1 | 0.2% | 0.493 | 1 | 0.9% | 1 | 0.2% | 0.208 | 0 | 0.0% | 2 | 0.4% | 0.517 | 0 | 0.0% | 2 | 0.5% | 0.223 |
| *isdD* | 0 | 0.0% | 1 | 0.7% | 1 | 0.2% | 0.493 | 1 | 0.9% | 1 | 0.2% | 0.208 | 0 | 0.0% | 2 | 0.4% | 0.517 | 0 | 0.0% | 2 | 0.5% | 0.223 |
| *isdE* | 0 | 0.0% | 1 | 0.7% | 1 | 0.2% | 0.493 | 1 | 0.9% | 1 | 0.2% | 0.208 | 0 | 0.0% | 2 | 0.4% | 0.517 | 0 | 0.0% | 2 | 0.5% | 0.223 |
| *isdF* | 0 | 0.0% | 1 | 0.7% | 0 | 0.0% | 0.132 | 1 | 0.9% | 0 | 0.0% | 0.026 | 0 | 0.0% | 1 | 0.2% | 0.647 | 0 | 0.0% | 1 | 0.3% | 0.390 |
| *isdG* | 0 | 0.0% | 1 | 0.7% | 0 | 0.0% | 0.132 | 1 | 0.9% | 0 | 0.0% | 0.026 | 0 | 0.0% | 1 | 0.2% | 0.647 | 0 | 0.0% | 1 | 0.3% | 0.390 |
| *sfaA* | 0 | 0.0% | 1 | 0.7% | 1 | 0.2% | 0.493 | 1 | 0.9% | 1 | 0.2% | 0.208 | 0 | 0.0% | 2 | 0.4% | 0.517 | 1 | 0.3% | 1 | 0.3% | 0.832 |
| *sfaB* | 0 | 0.0% | 1 | 0.7% | 1 | 0.2% | 0.493 | 1 | 0.9% | 1 | 0.2% | 0.208 | 0 | 0.0% | 2 | 0.4% | 0.517 | 1 | 0.3% | 1 | 0.3% | 0.832 |
| *sfaC* | 0 | 0.0% | 1 | 0.7% | 1 | 0.2% | 0.493 | 1 | 0.9% | 1 | 0.2% | 0.208 | 0 | 0.0% | 2 | 0.4% | 0.517 | 1 | 0.3% | 1 | 0.3% | 0.832 |
| *sfaD* | 0 | 0.0% | 0 | 0.0% | 1 | 0.2% | 0.593 | 1 | 0.9% | 0 | 0.0% | 0.026 | 0 | 0.0% | 1 | 0.2% | 0.647 | 1 | 0.3% | 0 | 0.0% | 0.246 |
| *srtB* | 0 | 0.0% | 1 | 0.7% | 0 | 0.0% | 0.132 | 1 | 0.9% | 0 | 0.0% | 0.026 | 0 | 0.0% | 1 | 0.2% | 0.647 | 0 | 0.0% | 1 | 0.3% | 0.390 |
| *hla* | 0 | 0.0% | 2 | 1.4% | 7 | 1.3% | 0.805 | 1 | 0.9% | 8 | 1.4% | 0.648 | 5 | 4.2% | 4 | 0.7% | 0.002 | 3 | 1.0% | 6 | 1.5% | 0.573 |
| *hlb* | 0 | 0.0% | 17 | 11.5% | 66 | 12.6% | 0.254 | 12 | 10.4% | 71 | 12.4% | 0.549 | 8 | 6.7% | 75 | 13.2% | 0.048 | 54 | 18.5% | 29 | 7.4% | 0.000 |
| *hlgA* | 0 | 0.0% | 0 | 0.0% | 1 | 0.2% | 0.593 | 1 | 0.9% | 0 | 0.0% | 0.026 | 0 | 0.0% | 1 | 0.2% | 0.647 | 1 | 0.3% | 0 | 0.0% | 0.246 |
| *hlgC* | 0 | 0.0% | 0 | 0.0% | 2 | 0.4% | 0.449 | 1 | 0.9% | 1 | 0.2% | 0.208 | 0 | 0.0% | 2 | 0.4% | 0.517 | 1 | 0.3% | 1 | 0.3% | 0.832 |
| *hlgB* | 0 | 0.0% | 0 | 0.0% | 2 | 0.4% | 0.449 | 1 | 0.9% | 1 | 0.2% | 0.208 | 0 | 0.0% | 2 | 0.4% | 0.517 | 1 | 0.3% | 1 | 0.3% | 0.832 |
| *hld* | 0 | 0.0% | 2 | 1.4% | 1 | 0.2% | 0.153 | 1 | 0.9% | 2 | 0.4% | 0.442 | 0 | 0.0% | 3 | 0.5% | 0.427 | 2 | 0.7% | 1 | 0.3% | 0.398 |
| *lukS-PV* | 0 | 0.0% | 4 | 2.7% | 4 | 0.8% | 0.168 | 1 | 0.9% | 7 | 1.2% | 0.746 | 1 | 0.8% | 7 | 1.2% | 0.716 | 7 | 2.4% | 1 | 0.3% | 0.010 |
| *lukF-PV* | 0 | 0.0% | 4 | 2.7% | 4 | 0.8% | 0.168 | 1 | 0.9% | 7 | 1.2% | 0.746 | 1 | 0.8% | 7 | 1.2% | 0.716 | 7 | 2.4% | 1 | 0.3% | 0.010 |
| *lukE* | 0 | 0.0% | 6 | 4.1% | 19 | 3.6% | 0.816 | 0 | 0.0% | 25 | 4.4% | 0.022 | 4 | 3.4% | 21 | 3.7% | 0.856 | 10 | 3.4% | 15 | 3.8% | 0.792 |
| *lukM* | 0 | 0.0% | 0 | 0.0% | 4 | 0.8% | 0.284 | 0 | 0.0% | 4 | 0.7% | 0.369 | 0 | 0.0% | 4 | 0.7% | 0.359 | 0 | 0.0% | 4 | 1.0% | 0.084 |
| *lukD* | 0 | 0.0% | 6 | 4.1% | 20 | 3.8% | 0.738 | 0 | 0.0% | 26 | 4.6% | 0.020 | 4 | 3.4% | 22 | 3.9% | 0.788 | 9 | 3.1% | 17 | 4.3% | 0.404 |
| *lukG* | 0 | 0.0% | 15 | 10.1% | 43 | 8.2% | 0.951 | 6 | 5.2% | 52 | 9.1% | 0.172 | 3 | 2.5% | 55 | 9.7% | 0.010 | 53 | 18.2% | 5 | 1.3% | 0.000 |
| *lukH* | 0 | 0.0% | 15 | 10.1% | 43 | 8.2% | 0.951 | 6 | 5.2% | 52 | 9.1% | 0.172 | 3 | 2.5% | 55 | 9.7% | 0.010 | 53 | 18.2% | 5 | 1.3% | 0.000 |
| *sea* | 0 | 0.0% | 6 | 4.1% | 4 | 0.8% | 0.028 | 4 | 3.5% | 6 | 1.1% | 0.048 | 1 | 0.8% | 9 | 1.6% | 0.537 | 5 | 1.7% | 5 | 1.3% | 0.633 |
| *seb* | 0 | 0.0% | 2 | 1.4% | 4 | 0.8% | 0.722 | 0 | 0.0% | 6 | 1.1% | 0.270 | 0 | 0.0% | 6 | 1.1% | 0.260 | 4 | 1.4% | 2 | 0.5% | 0.231 |
| *sec* | 0 | 0.0% | 1 | 0.7% | 9 | 1.7% | 0.292 | 0 | 0.0% | 10 | 1.8% | 0.153 | 0 | 0.0% | 10 | 1.8% | 0.145 | 9 | 3.1% | 1 | 0.3% | 0.002 |
| *seg* | 0 | 0.0% | 2 | 1.4% | 23 | 4.4% | 0.058 | 0 | 0.0% | 25 | 4.4% | 0.022 | 11 | 9.2% | 14 | 2.5% | 0.000 | 3 | 1.0% | 22 | 5.6% | 0.002 |
| *seh* | 0 | 0.0% | 2 | 1.4% | 0 | 0.0% | 0.033 | 0 | 0.0% | 2 | 0.4% | 0.526 | 0 | 0.0% | 2 | 0.4% | 0.517 | 1 | 0.3% | 1 | 0.3% | 0.832 |
| *sei* | 0 | 0.0% | 1 | 0.7% | 23 | 4.4% | 0.025 | 0 | 0.0% | 24 | 4.2% | 0.025 | 10 | 8.4% | 14 | 2.5% | 0.001 | 2 | 0.7% | 22 | 5.6% | 0.001 |
| *selk* | 0 | 0.0% | 1 | 0.7% | 8 | 1.5% | 0.352 | 1 | 0.9% | 8 | 1.4% | 0.648 | 0 | 0.0% | 9 | 1.6% | 0.167 | 5 | 1.7% | 4 | 1.0% | 0.428 |
| *sell* | 0 | 0.0% | 1 | 0.7% | 9 | 1.7% | 0.292 | 0 | 0.0% | 10 | 1.8% | 0.153 | 0 | 0.0% | 10 | 1.8% | 0.145 | 9 | 3.1% | 1 | 0.3% | 0.002 |
| *selm* | 0 | 0.0% | 1 | 0.7% | 12 | 2.3% | 0.169 | 0 | 0.0% | 13 | 2.3% | 0.103 | 3 | 2.5% | 10 | 1.8% | 0.582 | 3 | 1.0% | 10 | 2.5% | 0.152 |
| *seln* | 0 | 0.0% | 1 | 0.7% | 0 | 0.0% | 0.132 | 0 | 0.0% | 1 | 0.2% | 0.654 | 1 | 0.8% | 0 | 0.0% | 0.029 | 1 | 0.3% | 0 | 0.0% | 0.246 |
| *selo* | 0 | 0.0% | 1 | 0.7% | 12 | 2.3% | 0.169 | 0 | 0.0% | 13 | 2.3% | 0.103 | 3 | 2.5% | 10 | 1.8% | 0.582 | 3 | 1.0% | 10 | 2.5% | 0.152 |
| *selp* | 0 | 0.0% | 1 | 0.7% | 2 | 0.4% | 0.802 | 2 | 1.7% | 1 | 0.2% | 0.020 | 0 | 0.0% | 3 | 0.5% | 0.427 | 2 | 0.7% | 1 | 0.3% | 0.398 |
| *selq* | 0 | 0.0% | 1 | 0.7% | 7 | 1.3% | 0.426 | 1 | 0.9% | 7 | 1.2% | 0.746 | 0 | 0.0% | 8 | 1.4% | 0.193 | 5 | 1.7% | 3 | 0.8% | 0.252 |
| *selu* | 0 | 0.0% | 0 | 0.0% | 6 | 1.1% | 0.188 | 0 | 0.0% | 6 | 1.1% | 0.270 | 0 | 0.0% | 6 | 1.1% | 0.260 | 2 | 0.7% | 4 | 1.0% | 0.647 |
| *set3* | 0 | 0.0% | 0 | 0.0% | 1 | 0.2% | 0.593 | 0 | 0.0% | 1 | 0.2% | 0.654 | 0 | 0.0% | 1 | 0.2% | 0.647 | 1 | 0.3% | 0 | 0.0% | 0.246 |
| *set4* | 0 | 0.0% | 1 | 0.7% | 0 | 0.0% | 0.132 | 0 | 0.0% | 1 | 0.2% | 0.654 | 1 | 0.8% | 0 | 0.0% | 0.029 | 1 | 0.3% | 0 | 0.0% | 0.246 |
| *set5* | 0 | 0.0% | 1 | 0.7% | 0 | 0.0% | 0.132 | 0 | 0.0% | 1 | 0.2% | 0.654 | 1 | 0.8% | 0 | 0.0% | 0.029 | 0 | 0.0% | 1 | 0.3% | 0.390 |
| *set7* | 0 | 0.0% | 3 | 2.0% | 5 | 1.0% | 0.514 | 2 | 1.7% | 6 | 1.1% | 0.531 | 5 | 4.2% | 3 | 0.5% | 0.001 | 1 | 0.3% | 7 | 1.8% | 0.084 |
| *set8* | 0 | 0.0% | 5 | 3.4% | 3 | 0.6% | 0.035 | 3 | 2.6% | 5 | 0.9% | 0.115 | 5 | 4.2% | 3 | 0.5% | 0.001 | 1 | 0.3% | 7 | 1.8% | 0.084 |
| *set9* | 0 | 0.0% | 1 | 0.7% | 0 | 0.0% | 0.132 | 1 | 0.9% | 0 | 0.0% | 0.026 | 1 | 0.8% | 0 | 0.0% | 0.029 | 0 | 0.0% | 1 | 0.3% | 0.390 |
| *set10* | 0 | 0.0% | 2 | 1.4% | 0 | 0.0% | 0.033 | 1 | 0.9% | 1 | 0.2% | 0.208 | 2 | 1.7% | 0 | 0.0% | 0.002 | 0 | 0.0% | 2 | 0.5% | 0.223 |
| *set20* | 0 | 0.0% | 1 | 0.7% | 1 | 0.2% | 0.493 | 0 | 0.0% | 2 | 0.4% | 0.526 | 0 | 0.0% | 2 | 0.4% | 0.517 | 1 | 0.3% | 1 | 0.3% | 0.832 |
| *yent1* | 0 | 0.0% | 2 | 1.4% | 3 | 0.6% | 0.529 | 0 | 0.0% | 5 | 0.9% | 0.315 | 2 | 1.7% | 3 | 0.5% | 0.180 | 1 | 0.3% | 4 | 1.0% | 0.306 |
| *yent2* | 0 | 0.0% | 2 | 1.4% | 3 | 0.6% | 0.529 | 0 | 0.0% | 5 | 0.9% | 0.315 | 2 | 1.7% | 3 | 0.5% | 0.180 | 1 | 0.3% | 4 | 1.0% | 0.306 |
| *tsst-1* | 0 | 0.0% | 1 | 0.7% | 8 | 1.5% | 0.352 | 0 | 0.0% | 9 | 1.6% | 0.176 | 0 | 0.0% | 9 | 1.6% | 0.167 | 8 | 2.7% | 1 | 0.3% | 0.005 |
| *nuc* | 0 | 0.0% | 1 | 0.7% | 0 | 0.0% | 0.132 | 0 | 0.0% | 1 | 0.2% | 0.654 | 0 | 0.0% | 1 | 0.2% | 0.647 | 0 | 0.0% | 1 | 0.3% | 0.390 |
| *icaA* | 0 | 0.0% | 0 | 0.0% | 1 | 0.2% | 0.593 | 0 | 0.0% | 1 | 0.2% | 0.654 | 0 | 0.0% | 1 | 0.2% | 0.647 | 0 | 0.0% | 1 | 0.3% | 0.390 |
| *icaB* | 0 | 0.0% | 0 | 0.0% | 1 | 0.2% | 0.593 | 0 | 0.0% | 1 | 0.2% | 0.654 | 0 | 0.0% | 1 | 0.2% | 0.647 | 0 | 0.0% | 1 | 0.3% | 0.390 |
| *icaC* | 0 | 0.0% | 0 | 0.0% | 1 | 0.2% | 0.593 | 0 | 0.0% | 1 | 0.2% | 0.654 | 0 | 0.0% | 1 | 0.2% | 0.647 | 0 | 0.0% | 1 | 0.3% | 0.390 |
| **Antibiotic resistance genes** | | |  |  |  |  |  |  |  |  |  |  |  |  |  |  |  |  |  |  |  |  |
| *blaZ* | 0 | 0.0% | 2 | 1.4% | 5 | 1.0% | 0.899 | 2 | 1.7% | 5 | 0.9% | 0.401 | 4 | 3.4% | 3 | 0.5% | 0.005 | 4 | 1.4% | 3 | 0.8% | 0.434 |
| *mecA* | 0 | 0.0% | 1 | 0.7% | 3 | 0.6% | 0.960 | 4 | 3.5% | 0 | 0.0% | 0.000 | 4 | 3.4% | 0 | 0.0% | 0.000 | 0 | 0.0% | 4 | 1.0% | 0.084 |
| *aad(6)* | 0 | 0.0% | 2 | 1.4% | 4 | 0.8% | 0.722 | 1 | 0.9% | 5 | 0.9% | 0.995 | 2 | 1.7% | 4 | 0.7% | 0.300 | 2 | 0.7% | 4 | 1.0% | 0.647 |
| *aac6_Ie_aph2_Ia* | 0 | 0.0% | 9 | 6.1% | 16 | 3.1% | 0.311 | 4 | 3.5% | 21 | 3.7% | 0.917 | 21 | 17.6% | 4 | 0.7% | 0.000 | 2 | 0.7% | 23 | 5.8% | 0.000 |
| *ant(4')-Ib* | 0 | 0.0% | 0 | 0.0% | 1 | 0.2% | 0.593 | 0 | 0.0% | 1 | 0.2% | 0.654 | 0 | 0.0% | 1 | 0.2% | 0.647 | 0 | 0.0% | 1 | 0.3% | 0.390 |
| *ant(6)-Ia* | 0 | 0.0% | 7 | 4.7% | 8 | 1.5% | 0.103 | 3 | 2.6% | 12 | 2.1% | 0.735 | 12 | 10.1% | 3 | 0.5% | 0.000 | 0 | 0.0% | 15 | 3.8% | 0.001 |
| *ant(9)-Ia* | 0 | 0.0% | 3 | 2.0% | 3 | 0.6% | 0.234 | 2 | 1.7% | 4 | 0.7% | 0.276 | 3 | 2.5% | 3 | 0.5% | 0.034 | 1 | 0.3% | 5 | 1.3% | 0.198 |
| *aph(3')-IIIa* | 0 | 0.0% | 2 | 1.4% | 3 | 0.6% | 0.529 | 2 | 1.7% | 3 | 0.5% | 0.163 | 2 | 1.7% | 3 | 0.5% | 0.180 | 2 | 0.7% | 3 | 0.8% | 0.907 |
| *aph(2'')-Ia* | 0 | 0.0% | 1 | 0.7% | 0 | 0.0% | 0.132 | 0 | 0.0% | 1 | 0.2% | 0.654 | 1 | 0.8% | 0 | 0.0% | 0.029 | 0 | 0.0% | 1 | 0.3% | 0.390 |
| *arlR* | 0 | 0.0% | 0 | 0.0% | 6 | 1.1% | 0.188 | 1 | 0.9% | 5 | 0.9% | 0.995 | 2 | 1.7% | 4 | 0.7% | 0.300 | 3 | 1.0% | 3 | 0.8% | 0.712 |
| *arlS* | 0 | 0.0% | 0 | 0.0% | 4 | 0.8% | 0.284 | 0 | 0.0% | 4 | 0.7% | 0.369 | 1 | 0.8% | 3 | 0.5% | 0.686 | 3 | 1.0% | 1 | 0.3% | 0.189 |
| *qacA* | 0 | 0.0% | 1 | 0.7% | 0 | 0.0% | 0.132 | 0 | 0.0% | 1 | 0.2% | 0.654 | 0 | 0.0% | 1 | 0.2% | 0.647 | 0 | 0.0% | 1 | 0.3% | 0.390 |
| *qacJ* | 0 | 0.0% | 2 | 1.4% | 1 | 0.2% | 0.153 | 0 | 0.0% | 3 | 0.5% | 0.437 | 2 | 1.7% | 1 | 0.2% | 0.024 | 1 | 0.3% | 2 | 0.5% | 0.746 |
| *dfrC* | 0 | 0.0% | 2 | 1.4% | 10 | 1.9% | 0.496 | 3 | 2.6% | 9 | 1.6% | 0.442 | 6 | 5.0% | 6 | 1.1% | 0.003 | 2 | 0.7% | 10 | 2.5% | 0.067 |
| *dfrG* | 0 | 0.0% | 1 | 0.7% | 1 | 0.2% | 0.493 | 0 | 0.0% | 2 | 0.4% | 0.526 | 1 | 0.8% | 1 | 0.2% | 0.223 | 1 | 0.3% | 1 | 0.3% | 0.832 |
| *cat-TC* | 0 | 0.0% | 0 | 0.0% | 1 | 0.2% | 0.593 | 0 | 0.0% | 1 | 0.2% | 0.654 | 1 | 0.8% | 0 | 0.0% | 0.029 | 0 | 0.0% | 1 | 0.3% | 0.390 |
| *tet(*38*)* | 0 | 0.0% | 1 | 0.7% | 4 | 0.8% | 0.775 | 0 | 0.0% | 5 | 0.9% | 0.315 | 2 | 1.7% | 3 | 0.5% | 0.180 | 2 | 0.7% | 3 | 0.8% | 0.907 |
| *tet(*K*)* | 0 | 0.0% | 1 | 0.7% | 2 | 0.4% | 0.802 | 1 | 0.9% | 2 | 0.4% | 0.442 | 2 | 1.7% | 1 | 0.2% | 0.024 | 1 | 0.3% | 2 | 0.5% | 0.746 |
| *tet(*M*)* | 0 | 0.0% | 0 | 0.0% | 1 | 0.2% | 0.593 | 0 | 0.0% | 1 | 0.2% | 0.654 | 0 | 0.0% | 1 | 0.2% | 0.647 | 1 | 0.3% | 0 | 0.0% | 0.246 |
| *mphC* | 0 | 0.0% | 0 | 0.0% | 1 | 0.2% | 0.593 | 0 | 0.0% | 1 | 0.2% | 0.654 | 1 | 0.8% | 0 | 0.0% | 0.029 | 0 | 0.0% | 1 | 0.3% | 0.390 |
| *ermA* | 0 | 0.0% | 3 | 2.0% | 3 | 0.6% | 0.234 | 2 | 1.7% | 4 | 0.7% | 0.276 | 3 | 2.5% | 3 | 0.5% | 0.034 | 1 | 0.3% | 5 | 1.3% | 0.198 |
| *ermC* | 0 | 0.0% | 2 | 1.4% | 0 | 0.0% | 0.033 | 0 | 0.0% | 2 | 0.4% | 0.526 | 2 | 1.7% | 0 | 0.0% | 0.002 | 0 | 0.0% | 2 | 0.5% | 0.223 |
| *mepA* | 0 | 0.0% | 2 | 1.4% | 0 | 0.0% | 0.033 | 0 | 0.0% | 2 | 0.4% | 0.526 | 1 | 0.8% | 1 | 0.2% | 0.223 | 1 | 0.3% | 1 | 0.3% | 0.832 |
| *mepR* | 0 | 0.0% | 1 | 0.7% | 0 | 0.0% | 0.132 | 0 | 0.0% | 1 | 0.2% | 0.654 | 0 | 0.0% | 1 | 0.2% | 0.647 | 1 | 0.3% | 0 | 0.0% | 0.246 |
| *msrA* | 0 | 0.0% | 0 | 0.0% | 1 | 0.2% | 0.593 | 0 | 0.0% | 1 | 0.2% | 0.654 | 1 | 0.8% | 0 | 0.0% | 0.029 | 0 | 0.0% | 1 | 0.3% | 0.390 |
| *norC* | 0 | 0.0% | 1 | 0.7% | 0 | 0.0% | 0.132 | 0 | 0.0% | 1 | 0.2% | 0.654 | 0 | 0.0% | 1 | 0.2% | 0.647 | 0 | 0.0% | 1 | 0.3% | 0.390 |
| *LmrS* | 0 | 0.0% | 1 | 0.7% | 4 | 0.8% | 0.775 | 1 | 0.9% | 4 | 0.7% | 0.846 | 0 | 0.0% | 5 | 0.9% | 0.305 | 2 | 0.7% | 3 | 0.8% | 0.907 |
| *sdrM* | 0 | 0.0% | 1 | 0.7% | 3 | 0.6% | 0.960 | 1 | 0.9% | 3 | 0.5% | 0.659 | 0 | 0.0% | 4 | 0.7% | 0.359 | 1 | 0.3% | 3 | 0.8% | 0.477 |
| *sepA* | 0 | 0.0% | 1 | 0.7% | 3 | 0.6% | 0.960 | 1 | 0.9% | 3 | 0.5% | 0.659 | 0 | 0.0% | 4 | 0.7% | 0.359 | 1 | 0.3% | 3 | 0.8% | 0.477 |
